# Supplementary figures and images for: Collateral deletion of the mitochondrial AAA+ ATPase ATAD1 sensitizes cancer cells to proteasome dysfunction (part 1 of 2)
Source: eLife. 2022 Nov 21;11:e82860. doi: 10.7554/eLife.82860 (PMC9815822; doi:10.7554/eLife.82860)

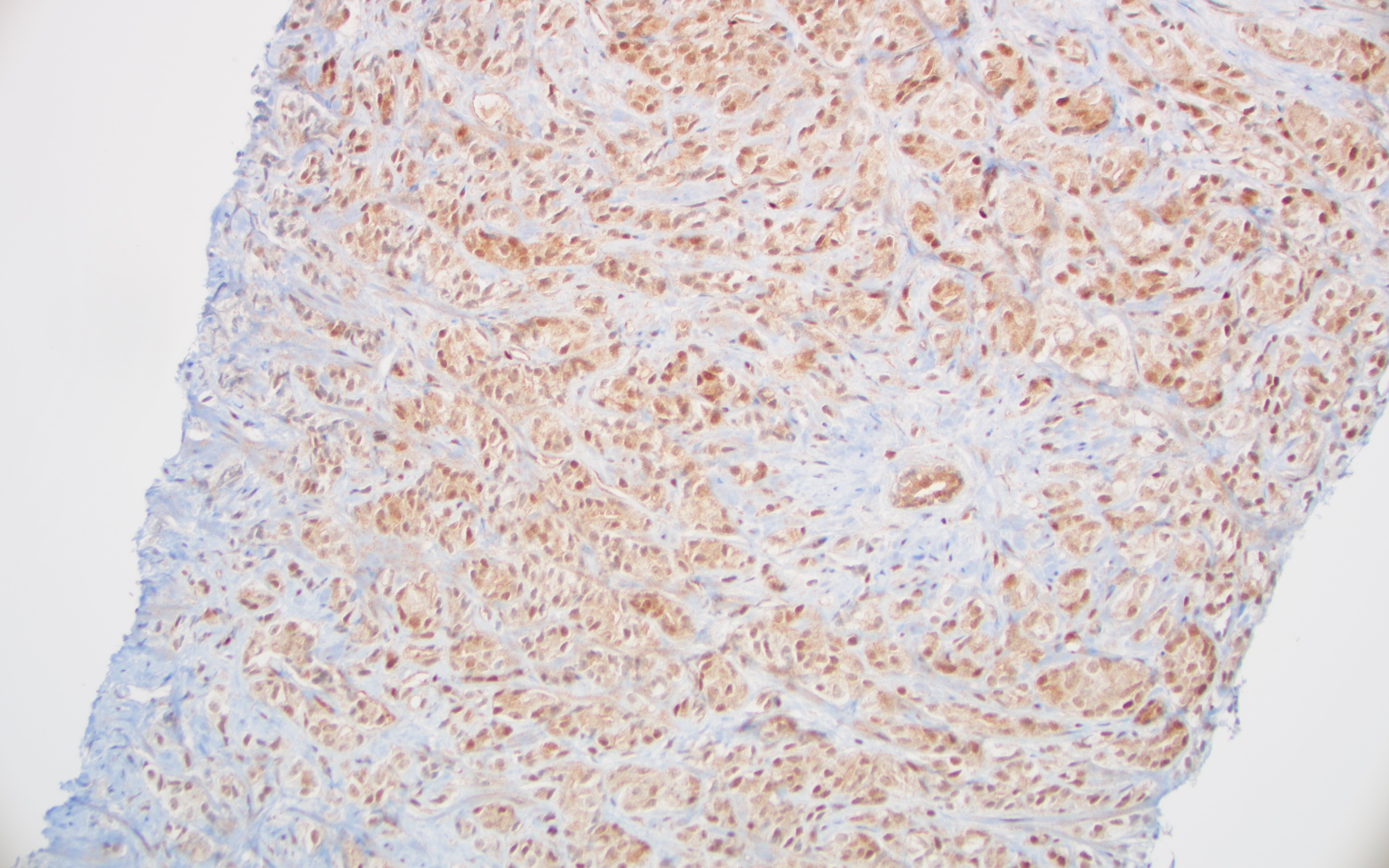

Supplement: Figure 1—figure supplement 1—source data 1. [file elife-82860-fig1-figsupp1-data1.zip › elife_Figure 1 Supplement 1/Figure 1 supplement 1 source data 2/Case 1_PTEN 20x.jpg]

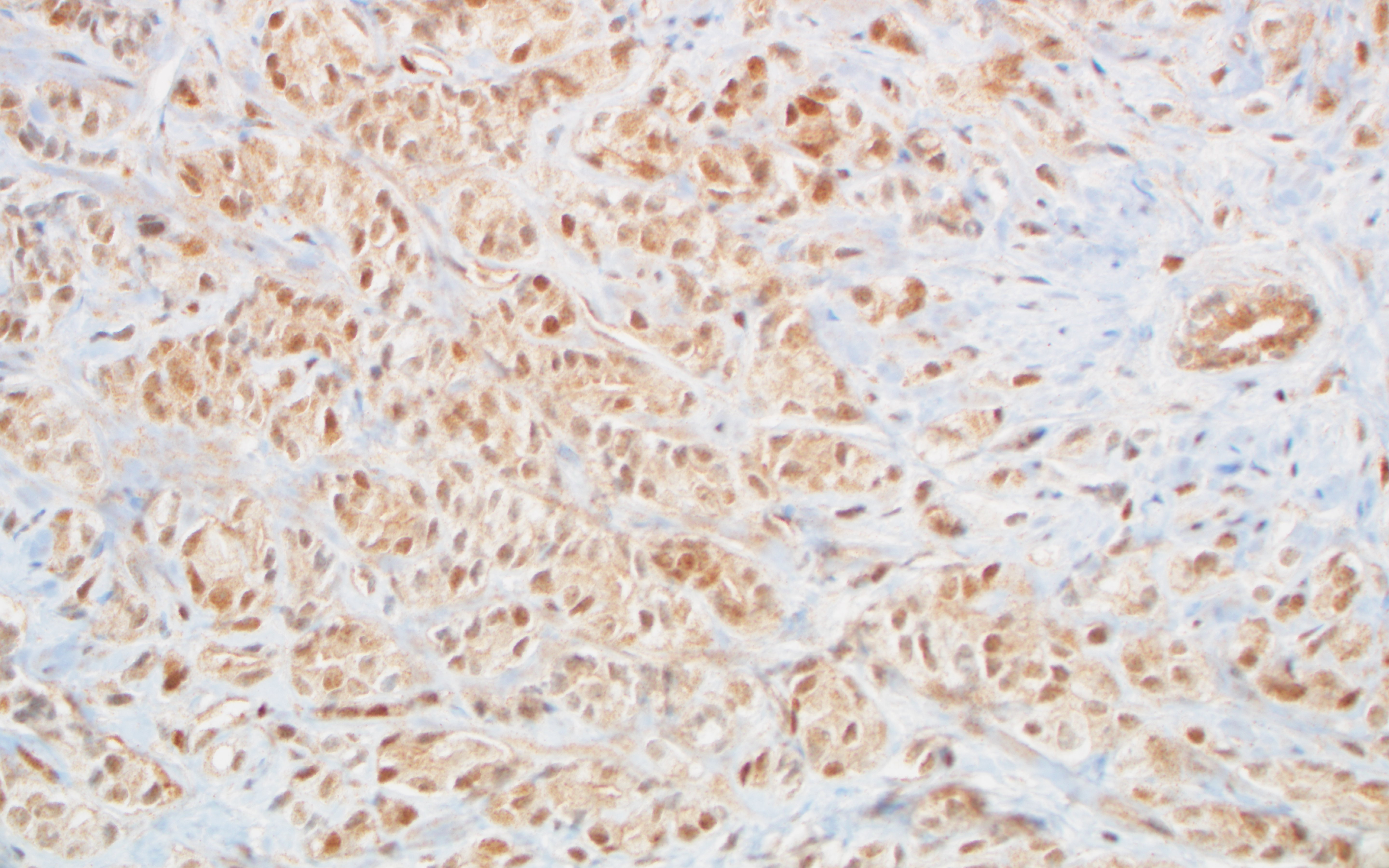

Supplement: Figure 1—figure supplement 1—source data 1. [file elife-82860-fig1-figsupp1-data1.zip › elife_Figure 1 Supplement 1/Figure 1 supplement 1 source data 2/Case 1_PTEN 40x.jpg]

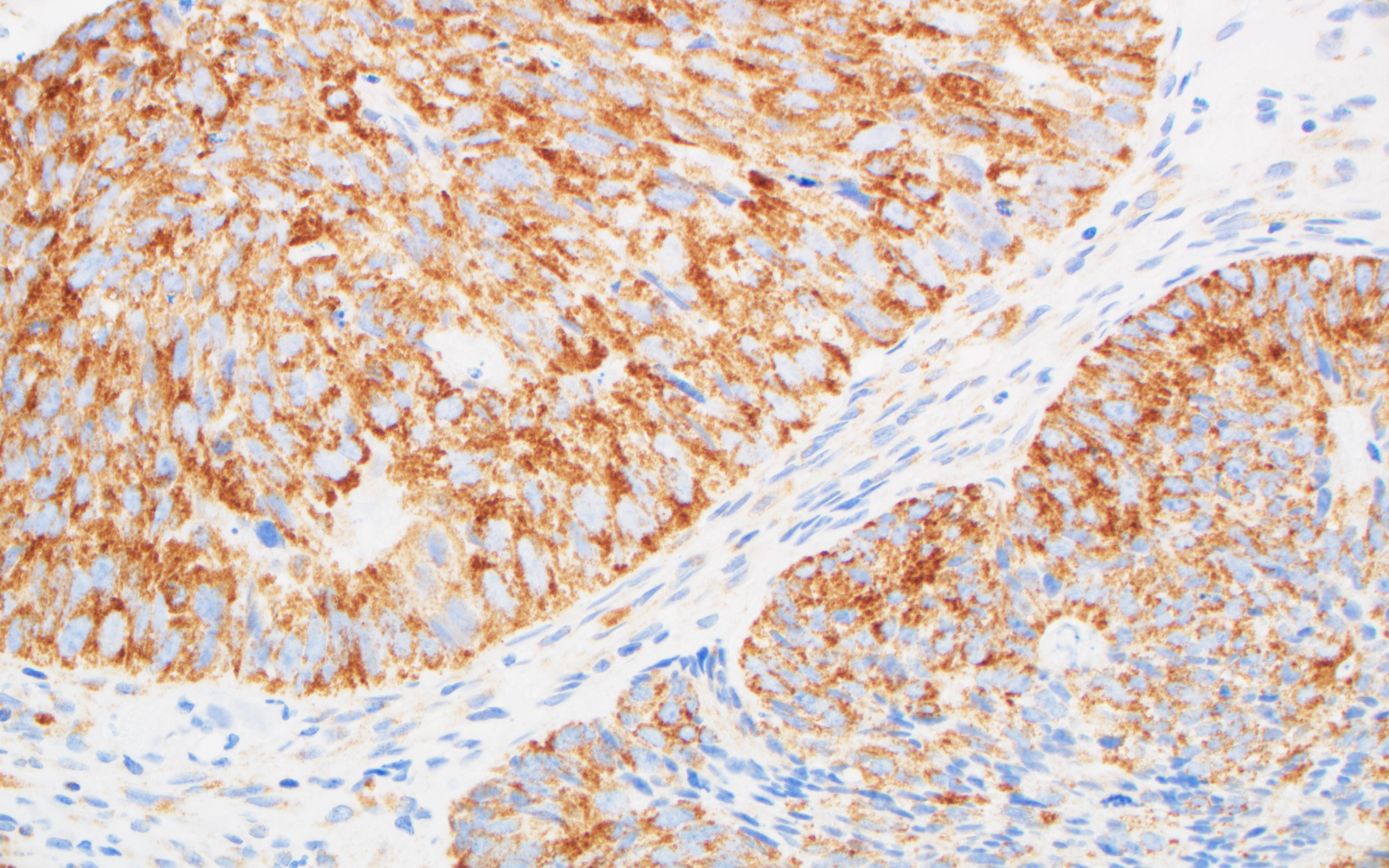

Supplement: Figure 1—figure supplement 1—source data 1. [file elife-82860-fig1-figsupp1-data1.zip › elife_Figure 1 Supplement 1/Figure 1 supplement 1 source data 2/Case 2_ATAD1 40x.jpg]

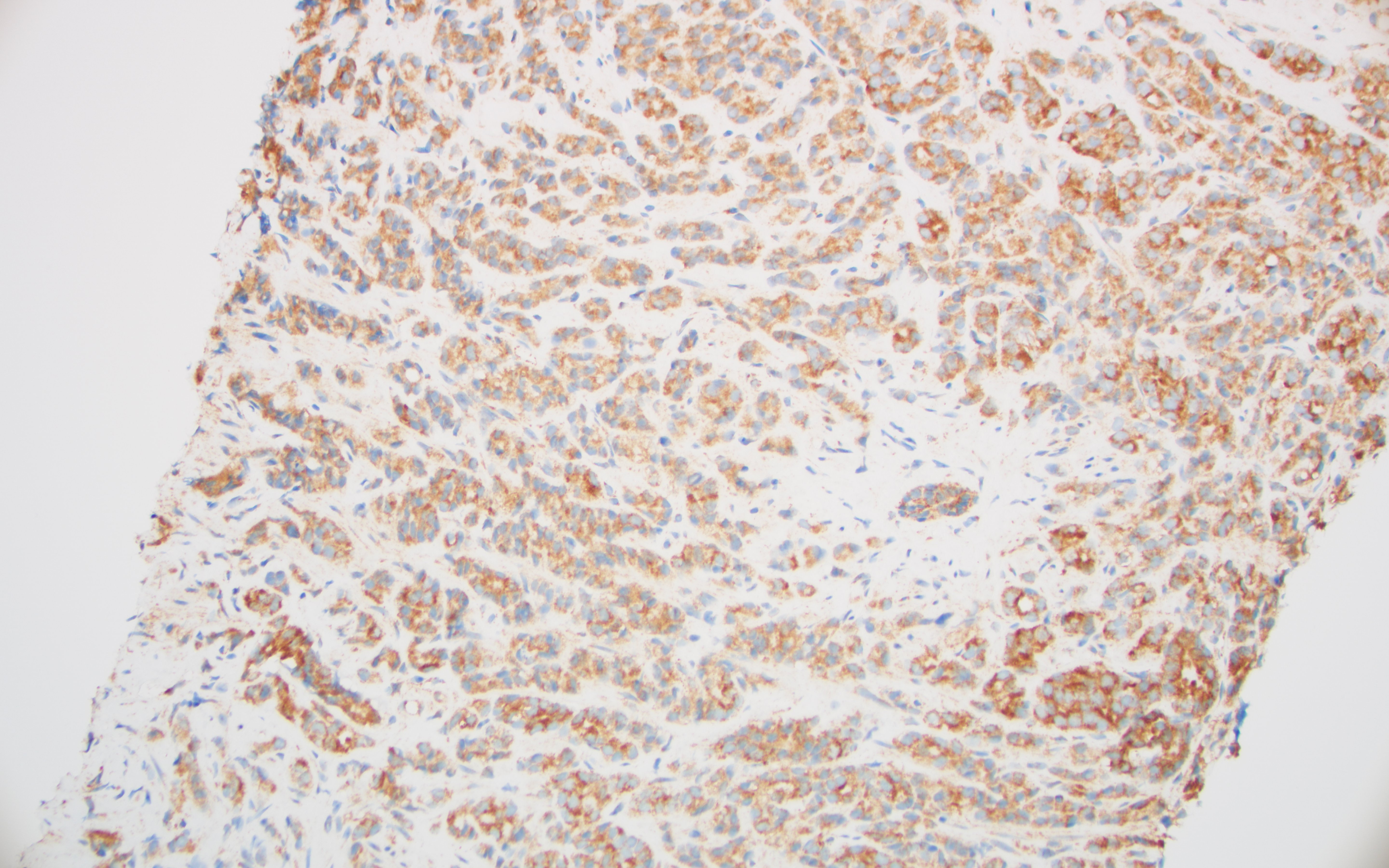

Supplement: Figure 1—figure supplement 1—source data 1. [file elife-82860-fig1-figsupp1-data1.zip › elife_Figure 1 Supplement 1/Figure 1 supplement 1 source data 2/Case 1_ATAD1 20x.jpg]

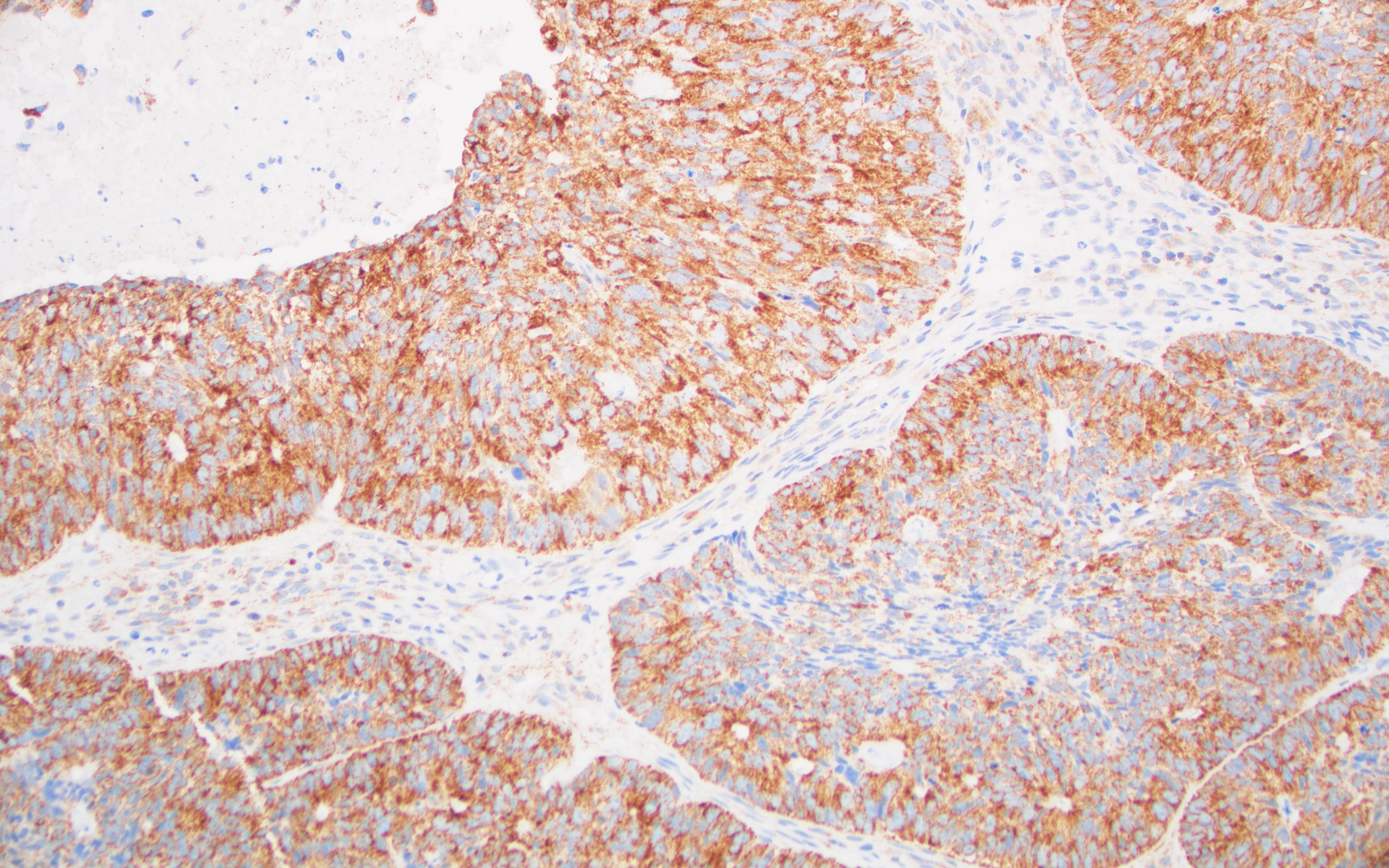

Supplement: Figure 1—figure supplement 1—source data 1. [file elife-82860-fig1-figsupp1-data1.zip › elife_Figure 1 Supplement 1/Figure 1 supplement 1 source data 2/Case 2_ATAD1 20x.jpg]

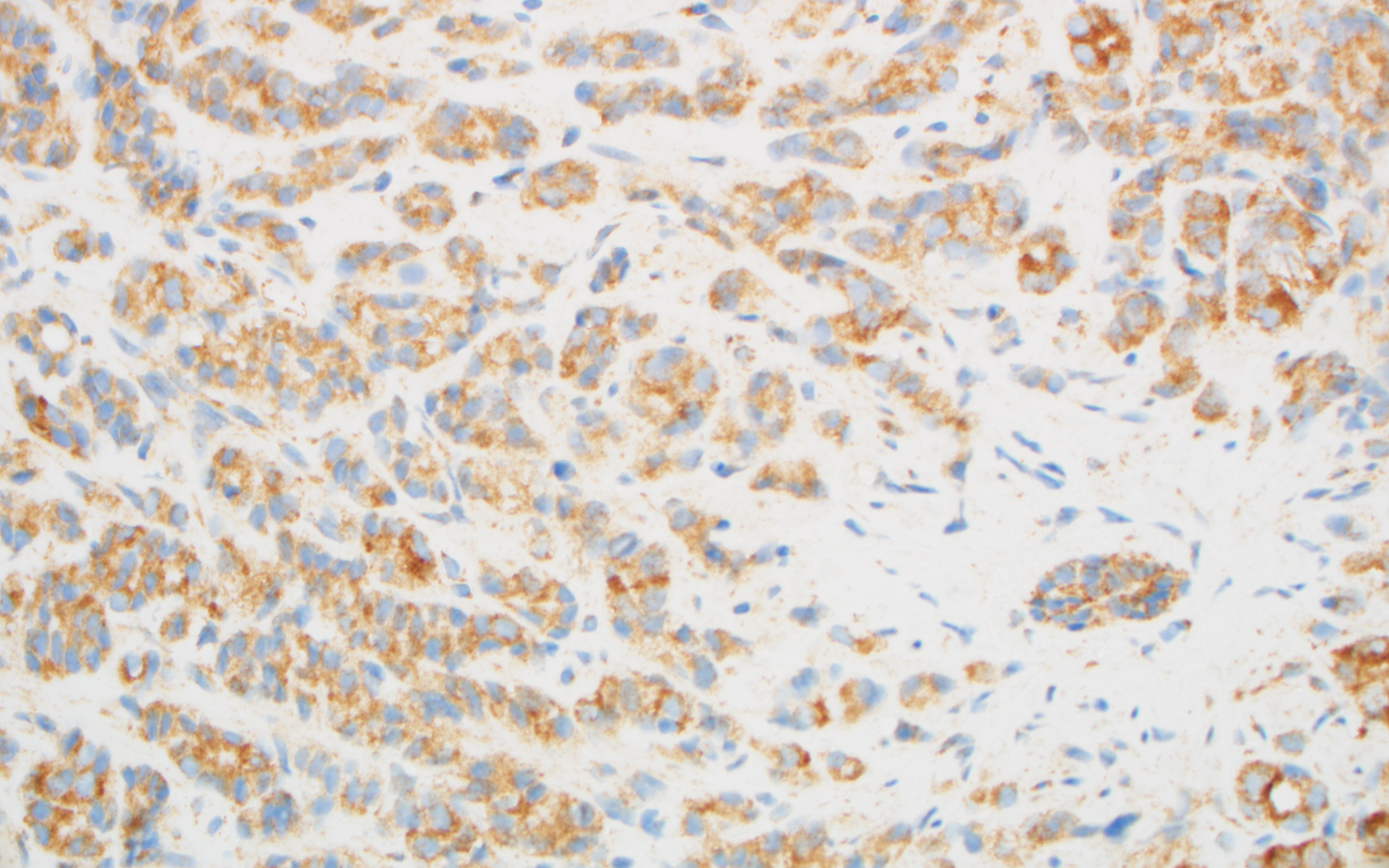

Supplement: Figure 1—figure supplement 1—source data 1. [file elife-82860-fig1-figsupp1-data1.zip › elife_Figure 1 Supplement 1/Figure 1 supplement 1 source data 2/Case 1_ATAD1 40x.jpg]

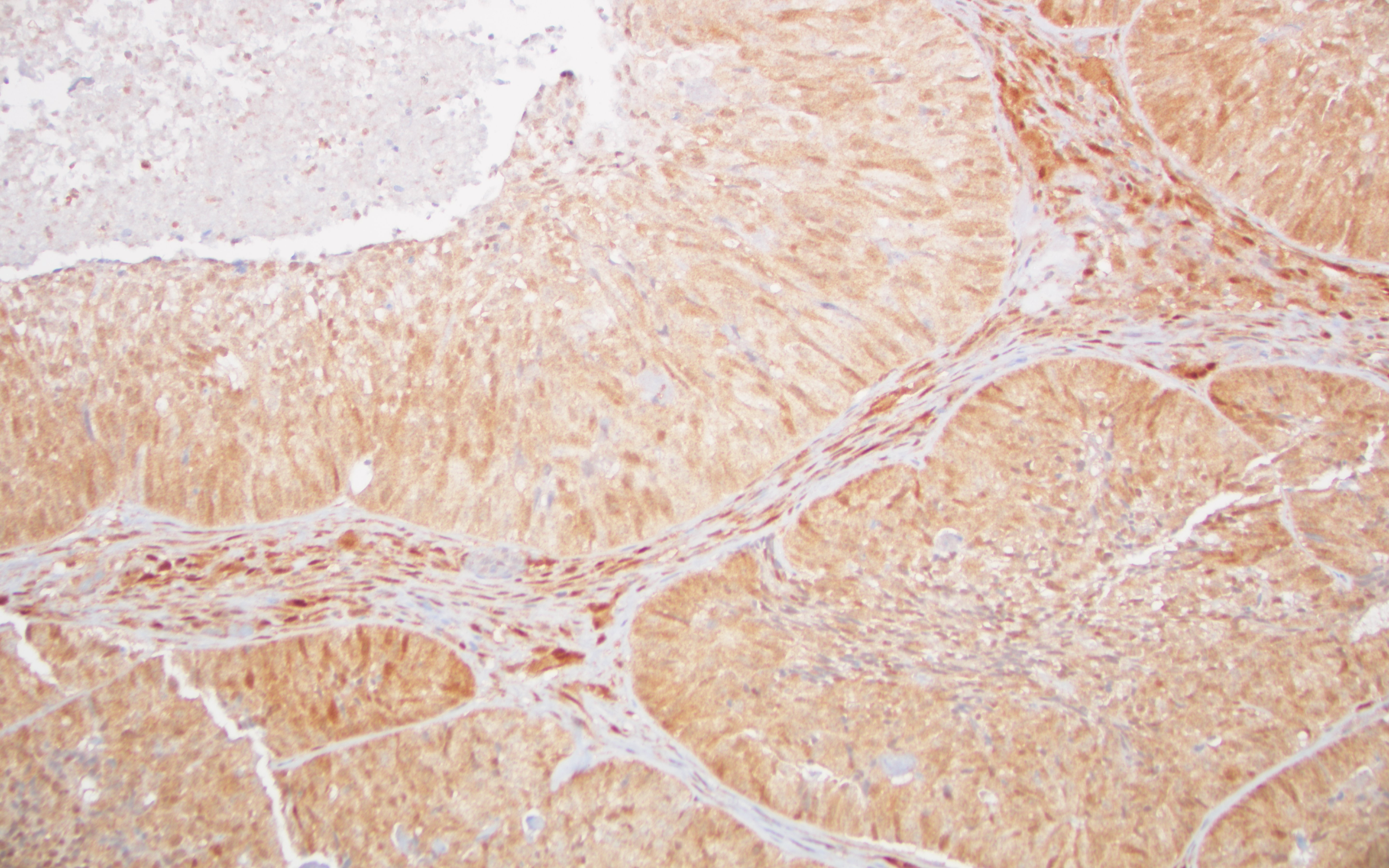

Supplement: Figure 1—figure supplement 1—source data 1. [file elife-82860-fig1-figsupp1-data1.zip › elife_Figure 1 Supplement 1/Figure 1 supplement 1 source data 2/Case 2_PTEN 20x.jpg]

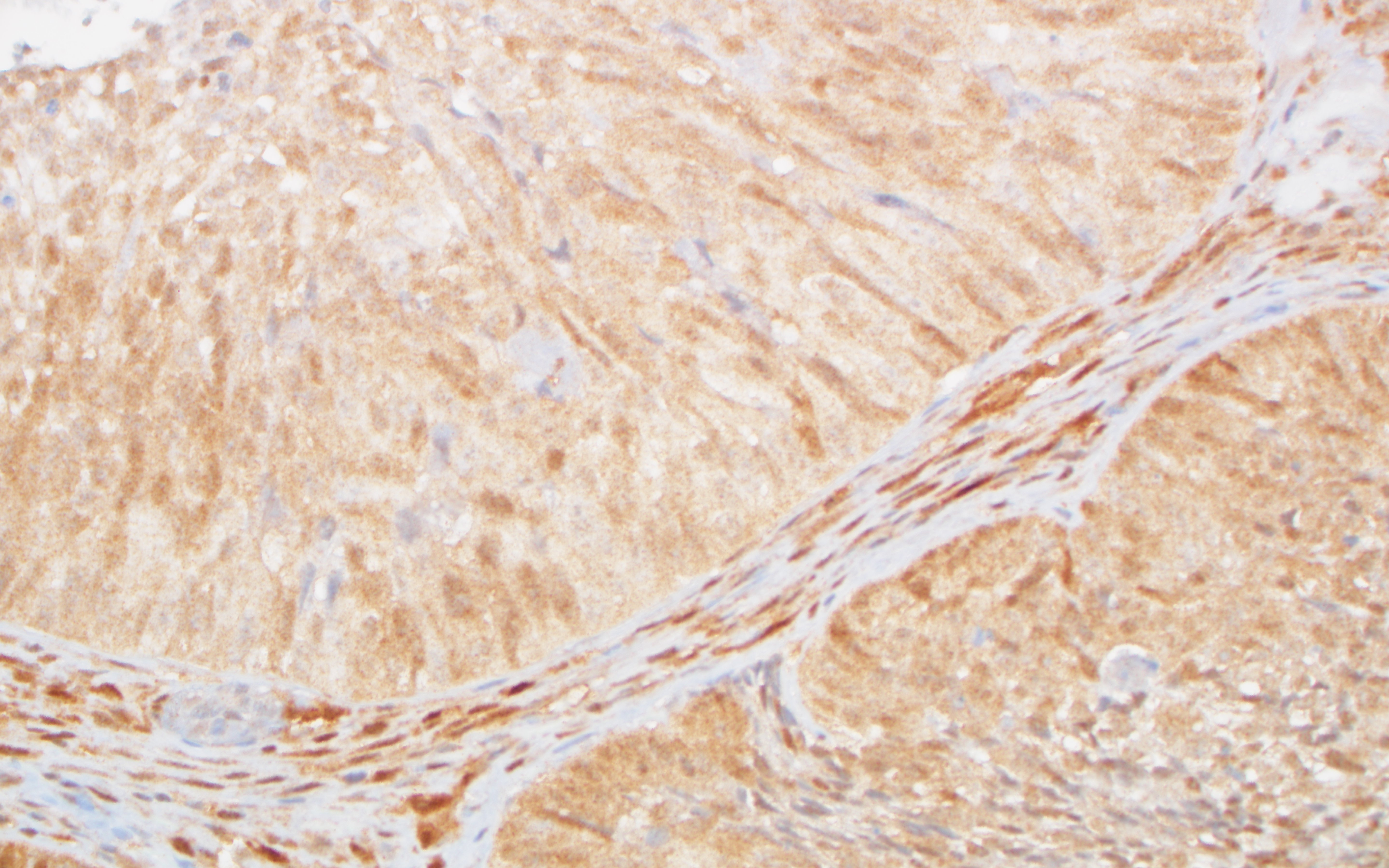

Supplement: Figure 1—figure supplement 1—source data 1. [file elife-82860-fig1-figsupp1-data1.zip › elife_Figure 1 Supplement 1/Figure 1 supplement 1 source data 2/Case 2_PTEN 40x.jpg]

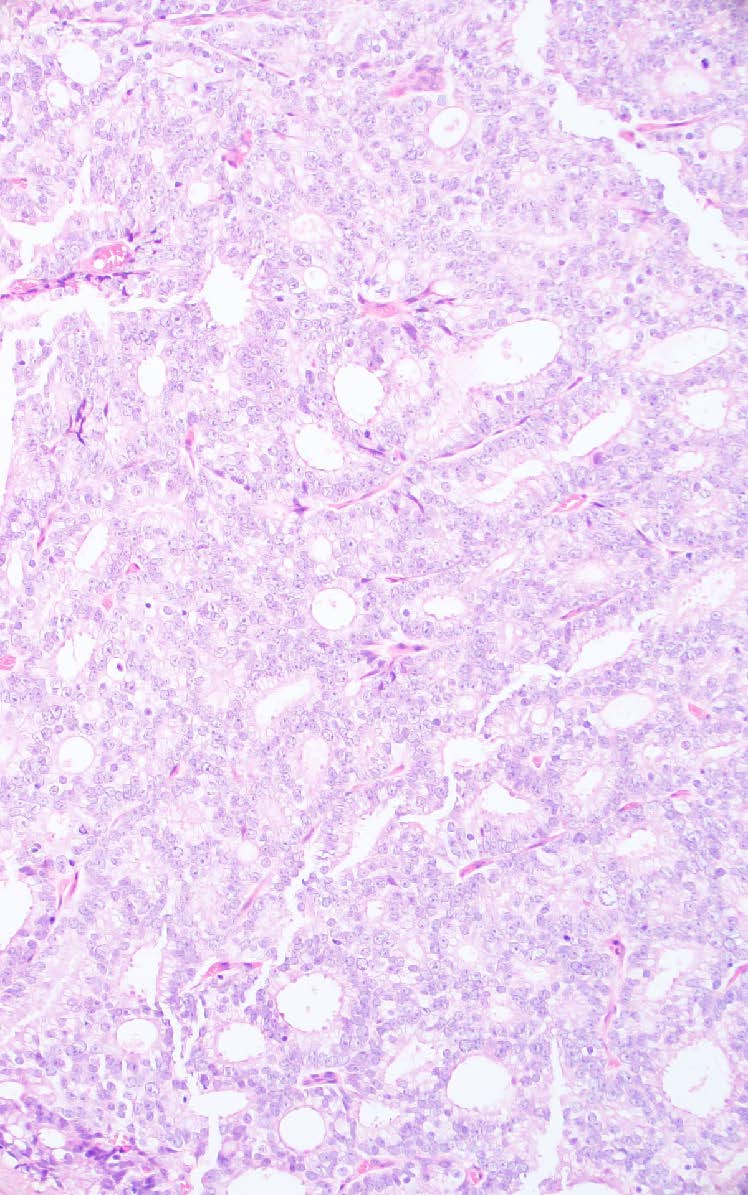

Supplement: Figure 1—figure supplement 1—source data 1. [file elife-82860-fig1-figsupp1-data1.zip › elife_Figure 1 Supplement 1/Figure 1 supplement 1 source data 1/ATAD1 Null HE.jpg]

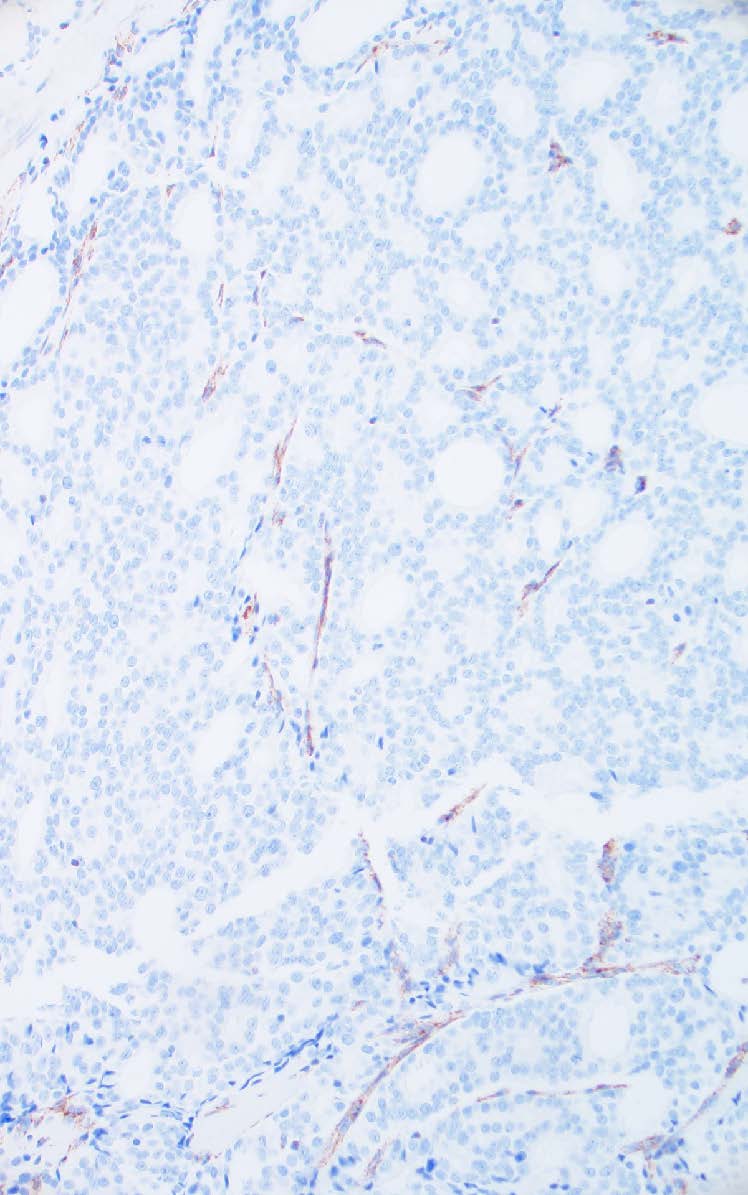

Supplement: Figure 1—figure supplement 1—source data 1. [file elife-82860-fig1-figsupp1-data1.zip › elife_Figure 1 Supplement 1/Figure 1 supplement 1 source data 1/ATAD1 Null IHC.jpg]

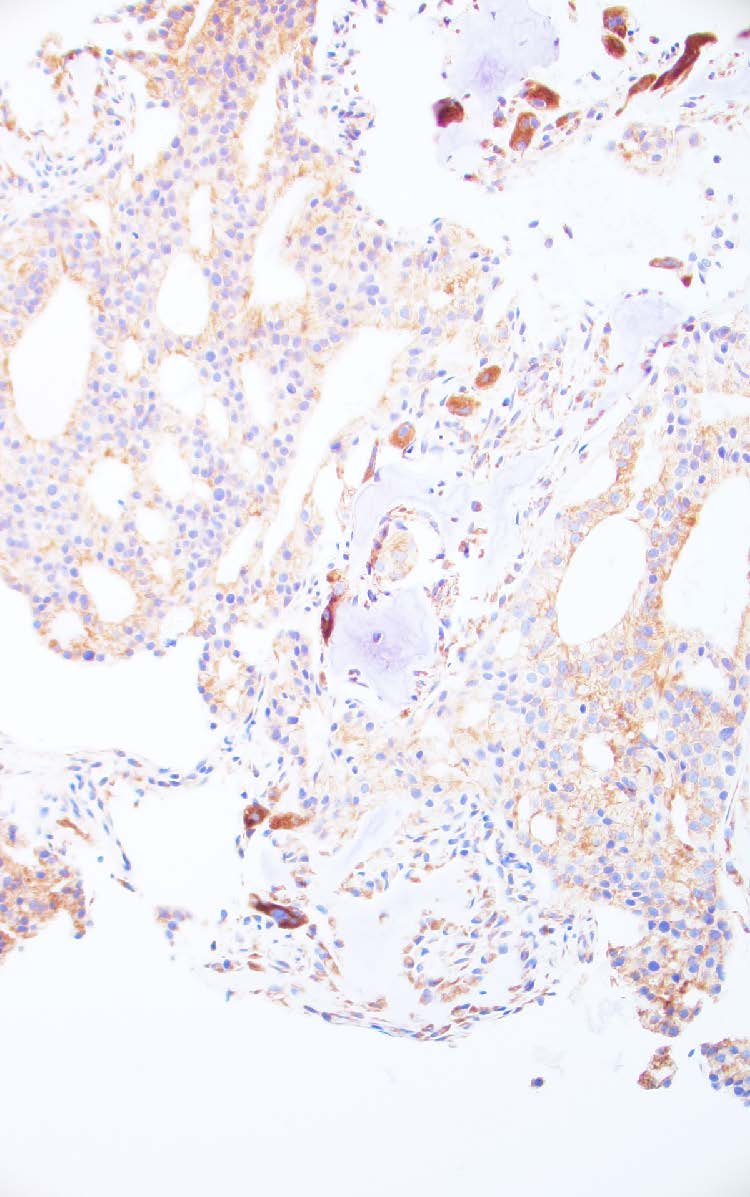

Supplement: Figure 1—figure supplement 1—source data 1. [file elife-82860-fig1-figsupp1-data1.zip › elife_Figure 1 Supplement 1/Figure 1 supplement 1 source data 1/ATAD1 Positive IHC.jpg]

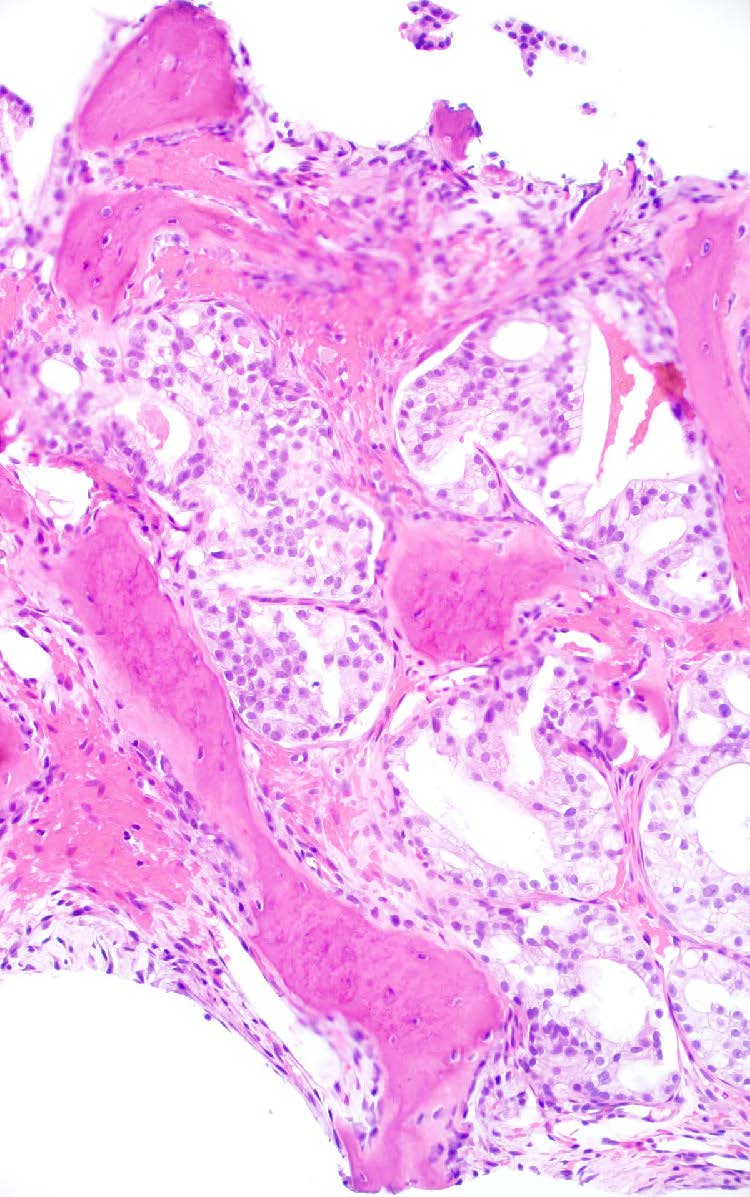

Supplement: Figure 1—figure supplement 1—source data 1. [file elife-82860-fig1-figsupp1-data1.zip › elife_Figure 1 Supplement 1/Figure 1 supplement 1 source data 1/ATAD1 Positive HE.jpg]

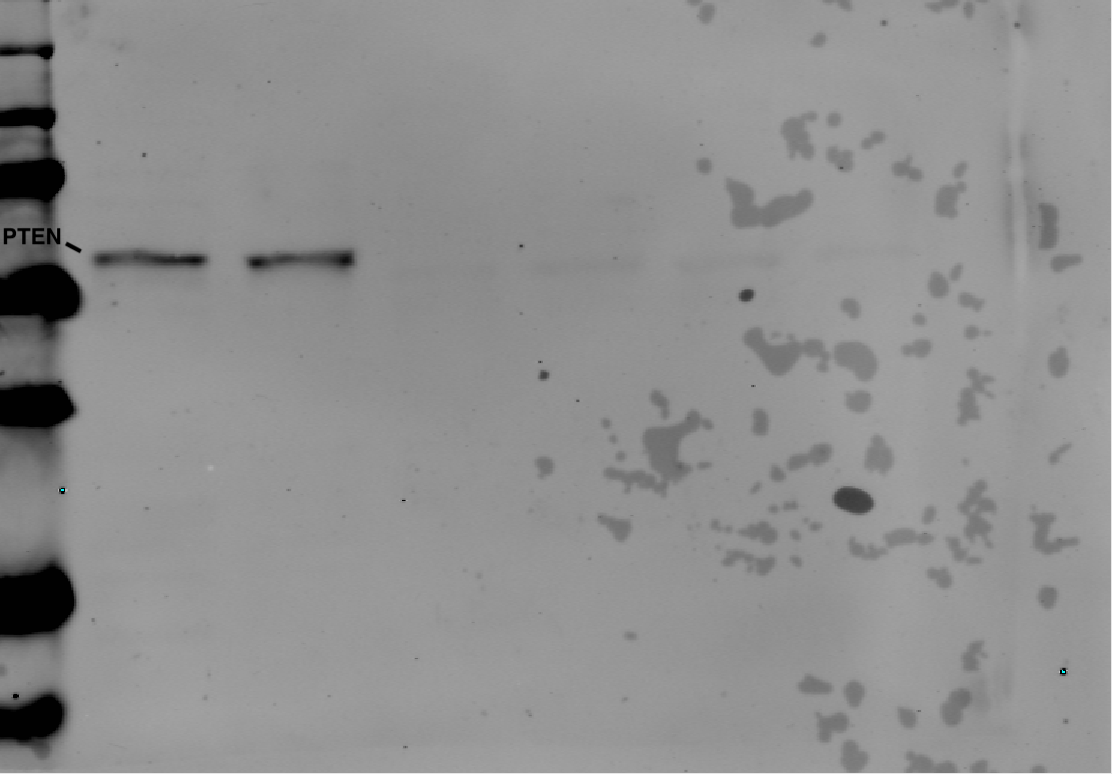

Supplement: Figure 1—figure supplement 3—source data 1. [file elife-82860-fig1-figsupp3-data1.zip › elife_Figure 1 supplement 3/Fig_1_F1S3A_Source Data/Fig_1_Supp_3A_Labeled/Fig_1_F1S3A PTEN labeled.tif]

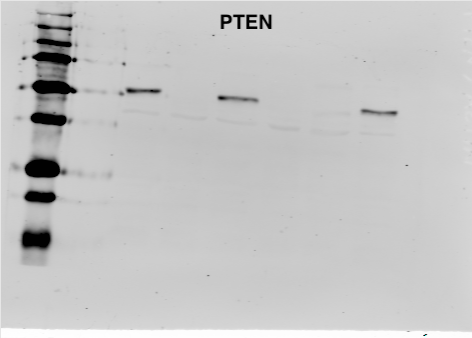

Supplement: Figure 1—figure supplement 3—source data 1. [file elife-82860-fig1-figsupp3-data1.zip › elife_Figure 1 supplement 3/Fig_1_F1S3A_Source Data/Fig_1_Supp_3A_Labeled/Fig_1_F1S3A Source Data PTEN labeled.tif]

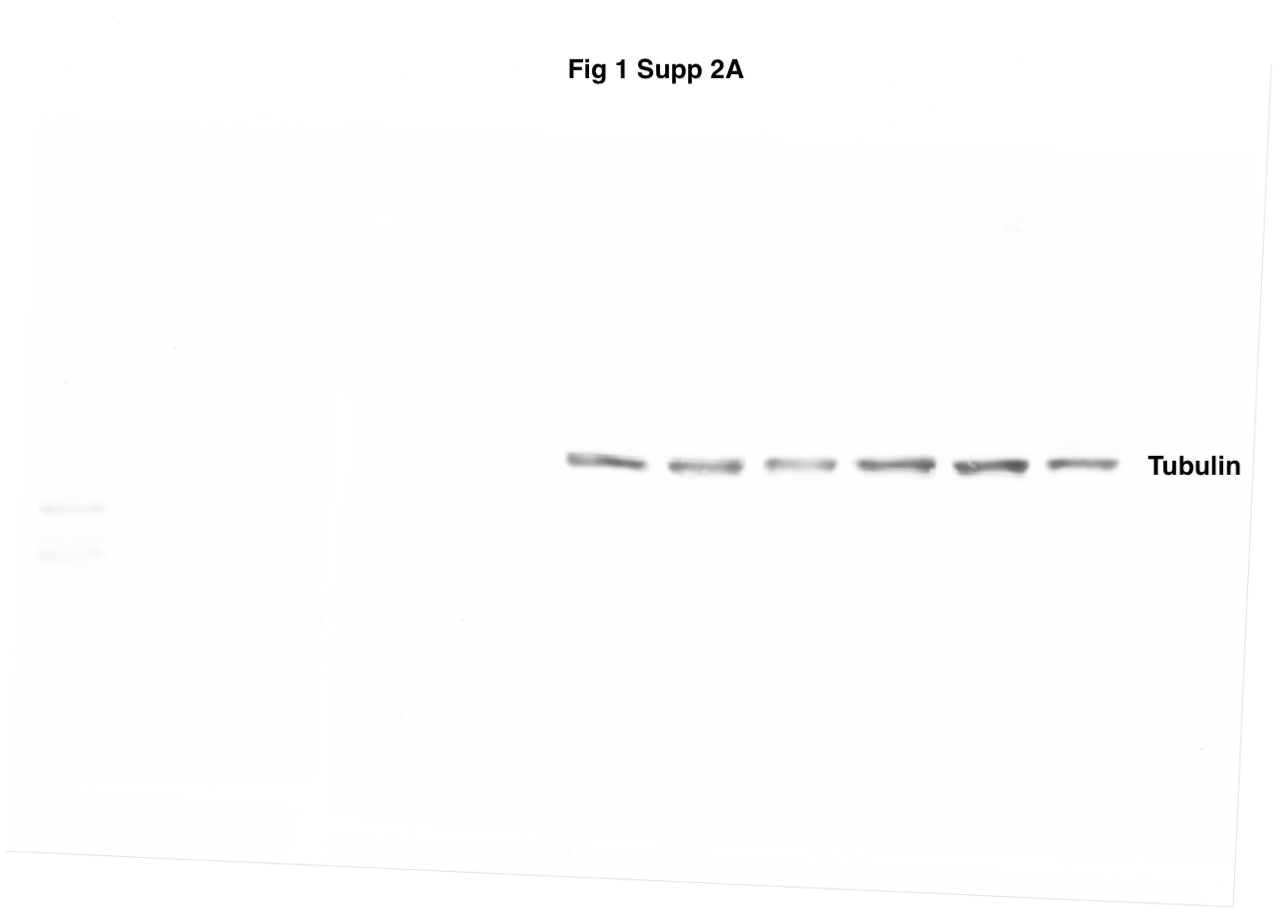

Supplement: Figure 1—figure supplement 3—source data 1. [file elife-82860-fig1-figsupp3-data1.zip › elife_Figure 1 supplement 3/Fig_1_F1S3A_Source Data/Fig_1_Supp_3A_Labeled/Fig_1_F1S3A tubulin labeled.tif]

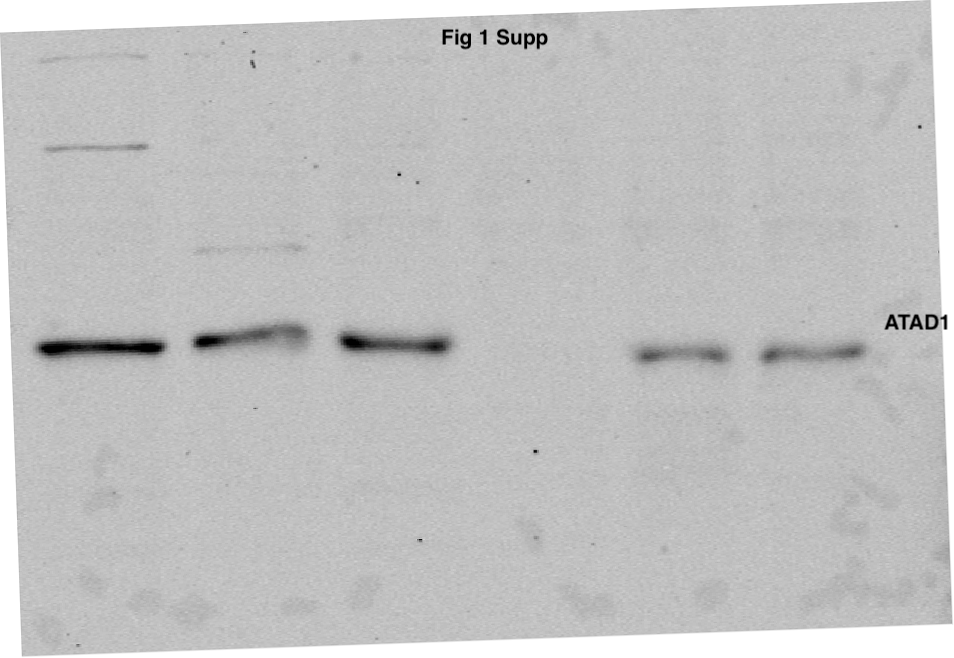

Supplement: Figure 1—figure supplement 3—source data 1. [file elife-82860-fig1-figsupp3-data1.zip › elife_Figure 1 supplement 3/Fig_1_F1S3A_Source Data/Fig_1_Supp_3A_Labeled/Fig_1_F1S3A atad1 labeled.tif]

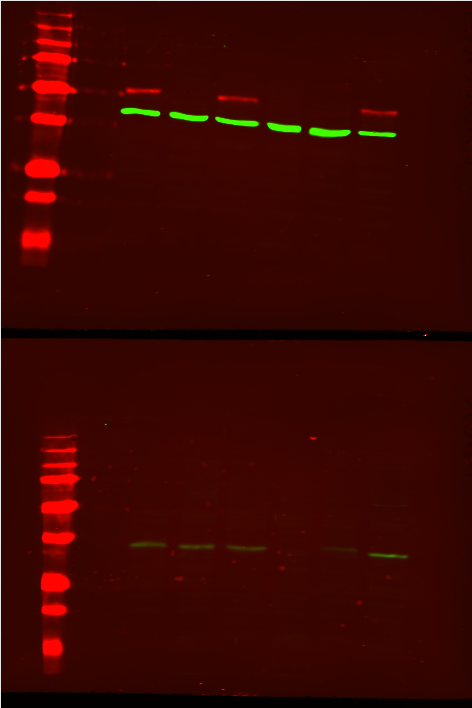

Supplement: Figure 1—figure supplement 3—source data 1. [file elife-82860-fig1-figsupp3-data1.zip › elife_Figure 1 supplement 3/Fig_1_F1S3A_Source Data/Fig_1_Supp_3A_Unlabeled/Fig_1_F1S3B Source Data PTEN ATAD1 Actin.tif]

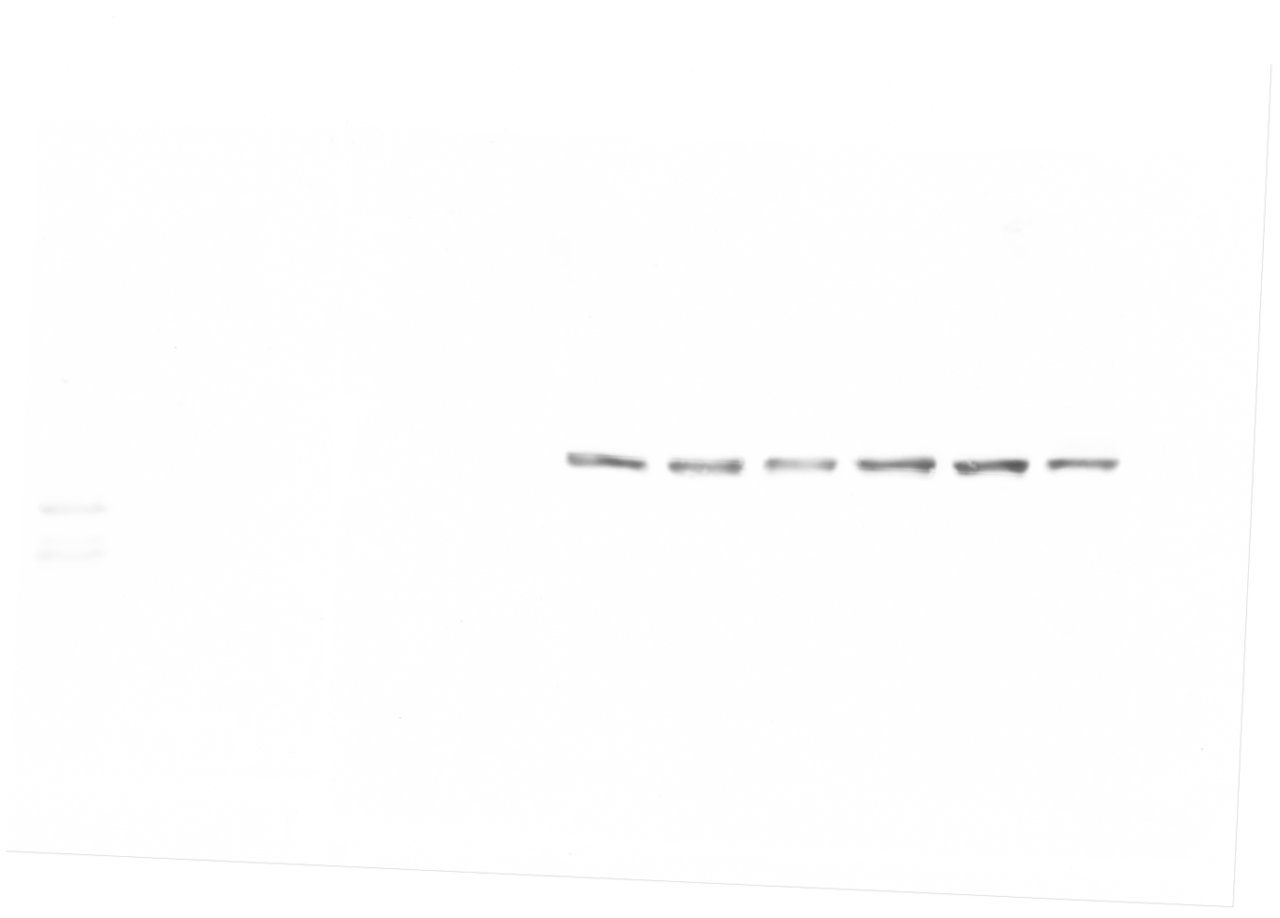

Supplement: Figure 1—figure supplement 3—source data 1. [file elife-82860-fig1-figsupp3-data1.zip › elife_Figure 1 supplement 3/Fig_1_F1S3A_Source Data/Fig_1_Supp_3A_Unlabeled/Fig_1_F1S3A tubulin.tif]

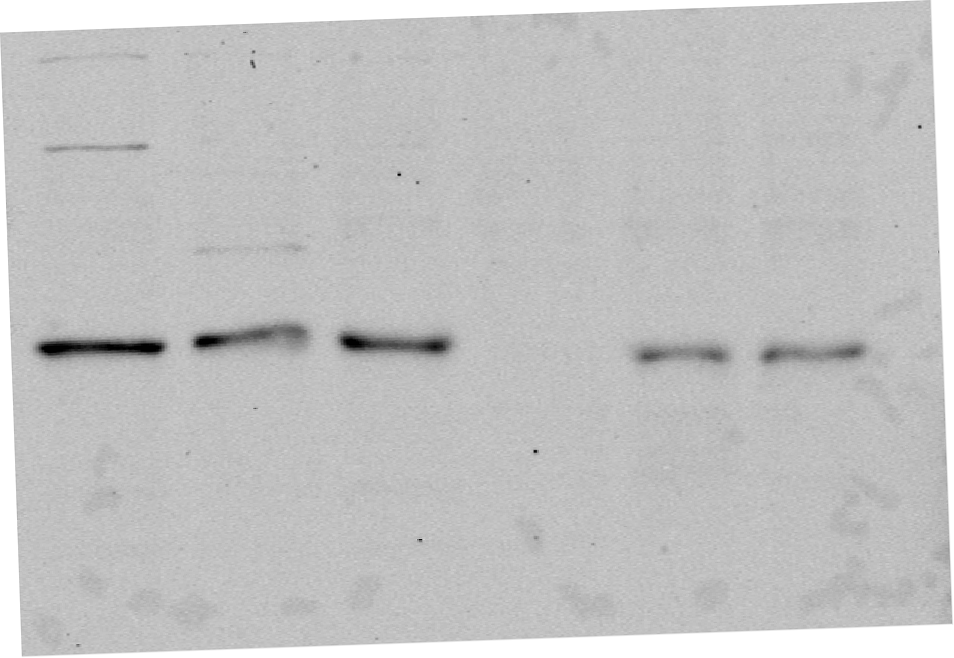

Supplement: Figure 1—figure supplement 3—source data 1. [file elife-82860-fig1-figsupp3-data1.zip › elife_Figure 1 supplement 3/Fig_1_F1S3A_Source Data/Fig_1_Supp_3A_Unlabeled/Fig_1_F1S3A atad1.tif]

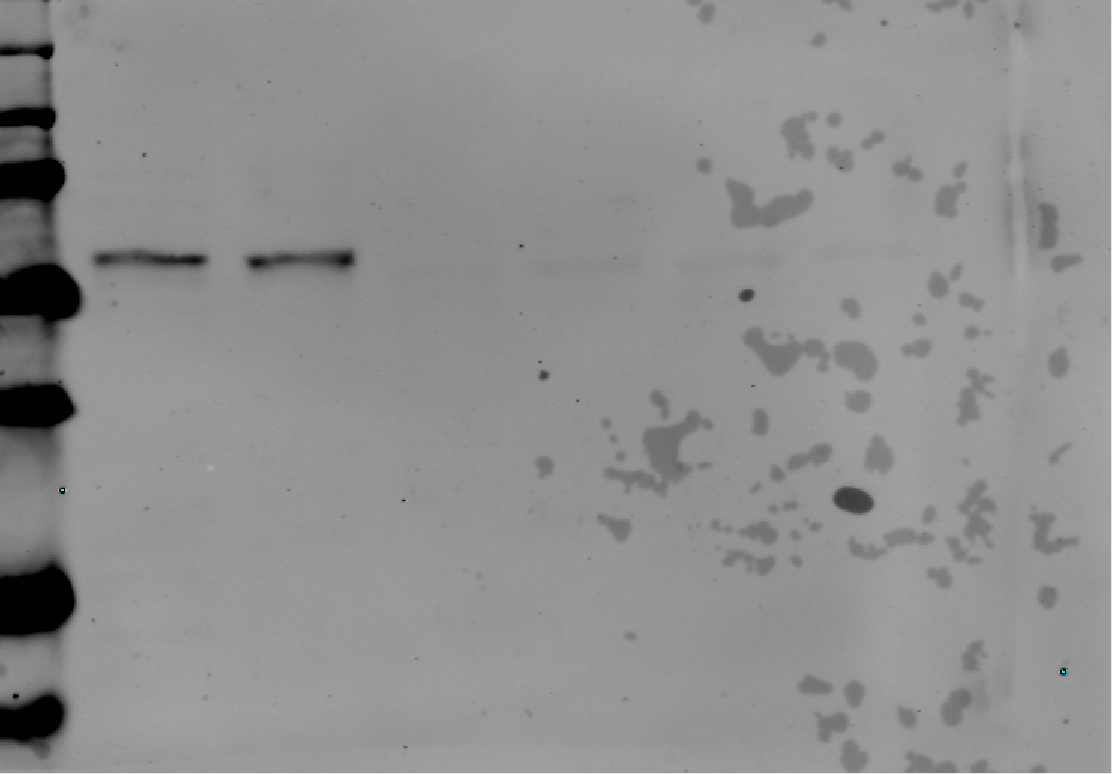

Supplement: Figure 1—figure supplement 3—source data 1. [file elife-82860-fig1-figsupp3-data1.zip › elife_Figure 1 supplement 3/Fig_1_F1S3A_Source Data/Fig_1_Supp_3A_Unlabeled/Fig_1_F1S3A PTEN unlabeled.tif]

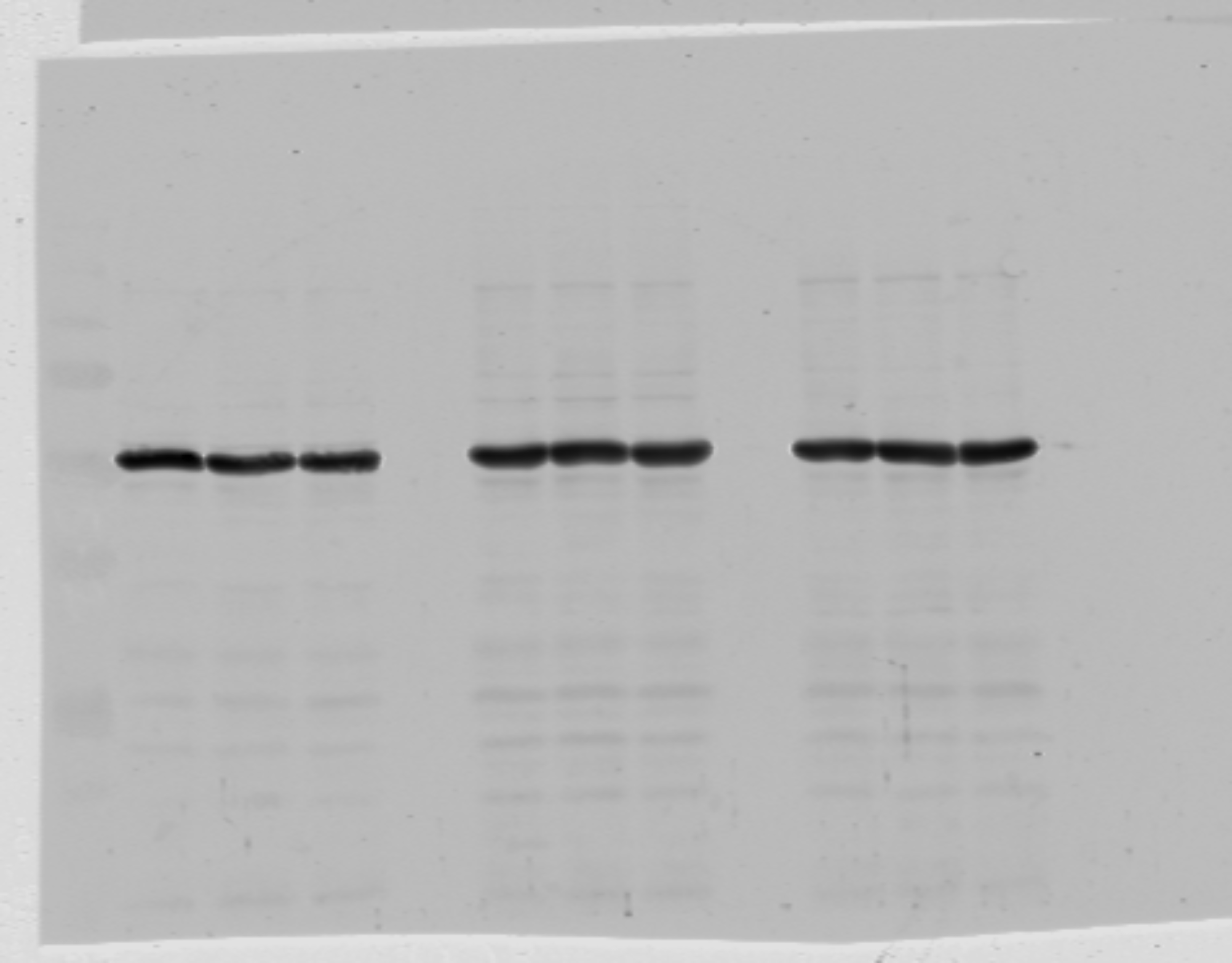

Supplement: Figure 1—figure supplement 3—source data 1. [file elife-82860-fig1-figsupp3-data1.zip › elife_Figure 1 supplement 3/Fig_1_F1S3B Source Data/Fig_1_Supp_2B_Unlabeled/Fig_1_F1S3B Source Data 1 tubulin unlabeled.tif.tif]

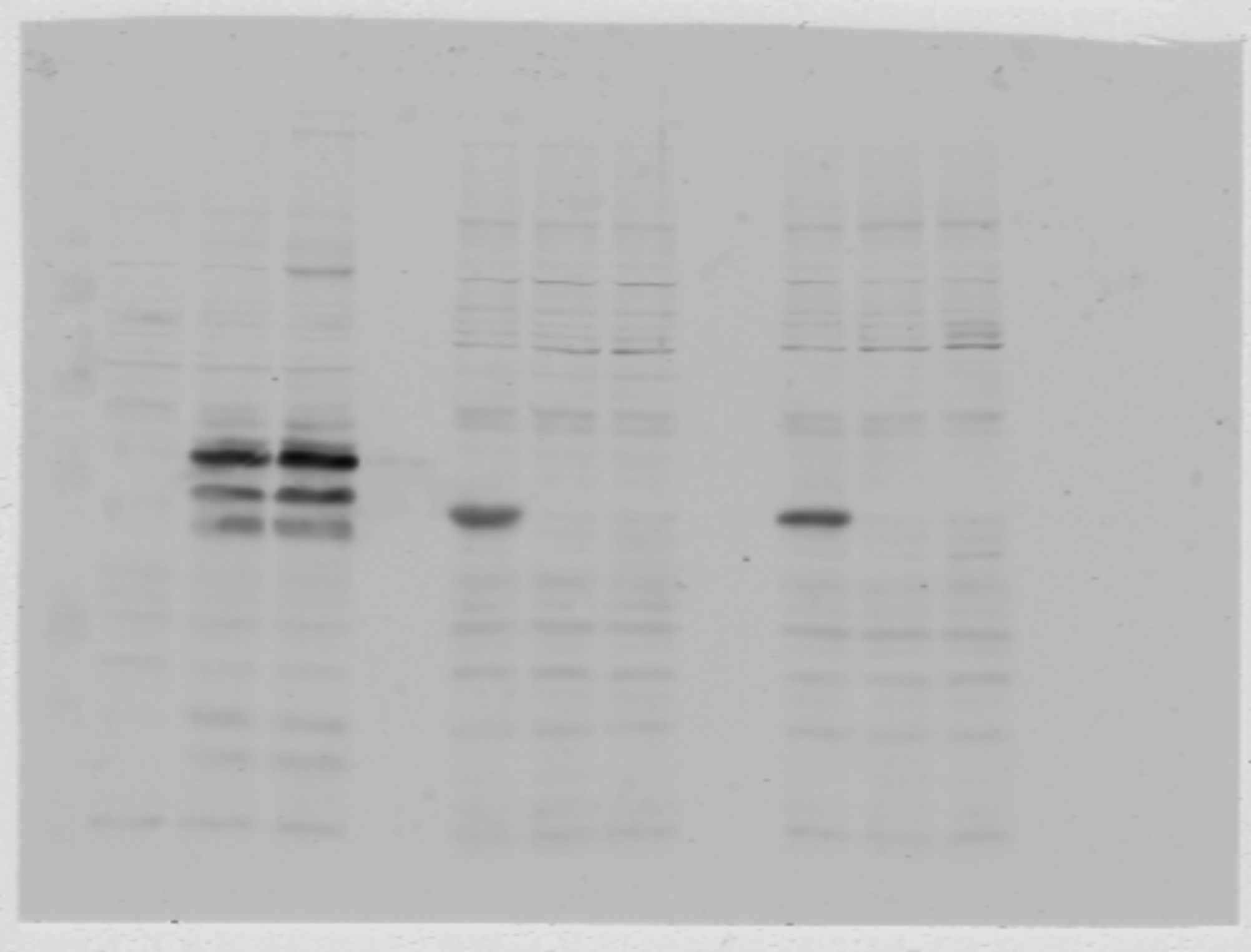

Supplement: Figure 1—figure supplement 3—source data 1. [file elife-82860-fig1-figsupp3-data1.zip › elife_Figure 1 supplement 3/Fig_1_F1S3B Source Data/Fig_1_Supp_2B_Unlabeled/Fig_1_F1S3B Source Data 2 atad1 unlabeled.tif.tif]

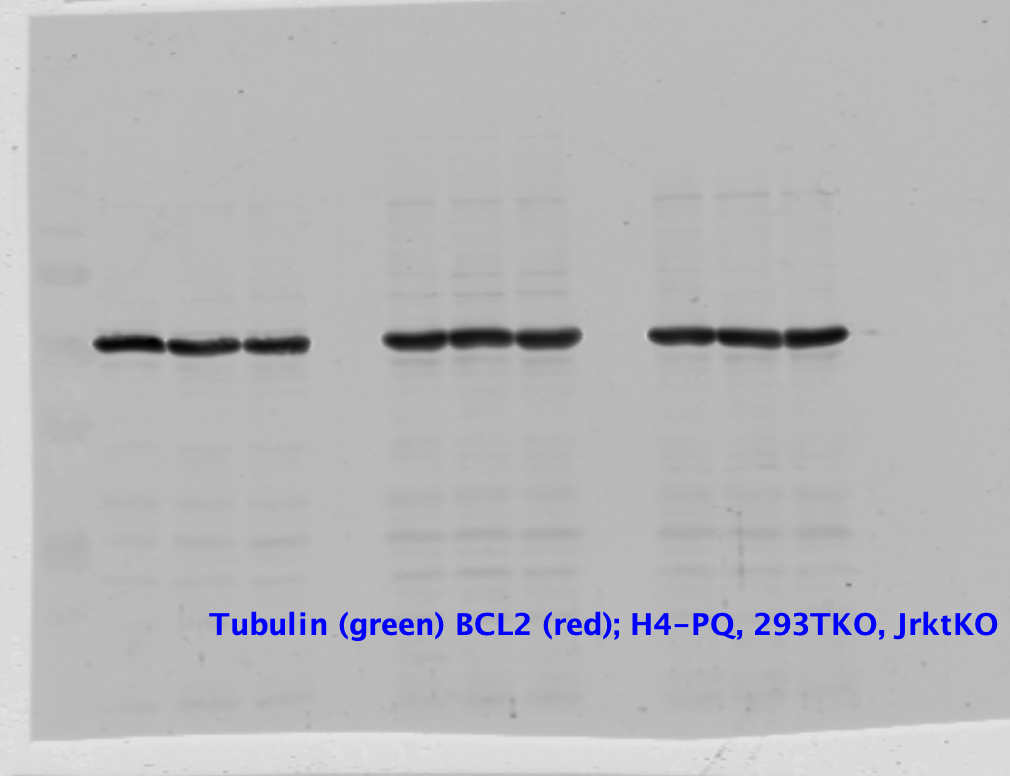

Supplement: Figure 1—figure supplement 3—source data 1. [file elife-82860-fig1-figsupp3-data1.zip › elife_Figure 1 supplement 3/Fig_1_F1S3B Source Data/Fig_1_Supp_2B_labeled/Fig_1_F1S3B Source Data 1 tubulin labeled.tif]

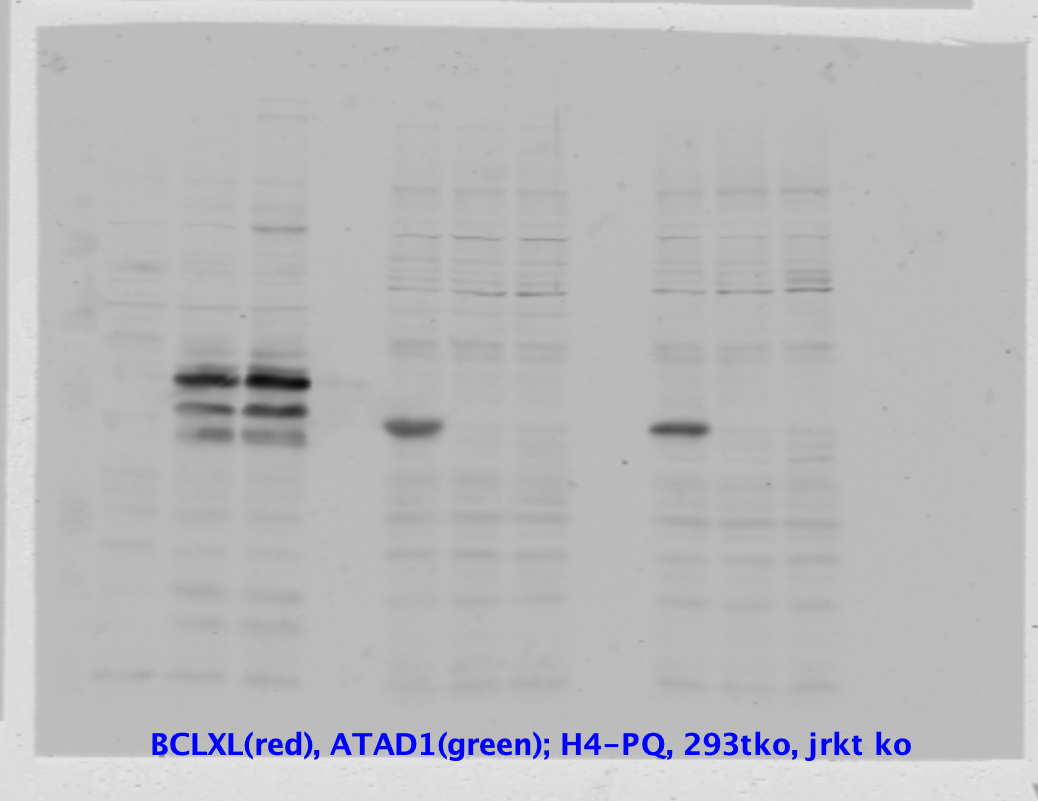

Supplement: Figure 1—figure supplement 3—source data 1. [file elife-82860-fig1-figsupp3-data1.zip › elife_Figure 1 supplement 3/Fig_1_F1S3B Source Data/Fig_1_Supp_2B_labeled/Fig_1_F1S3B Source Data 2 atad1 labeled.tif]

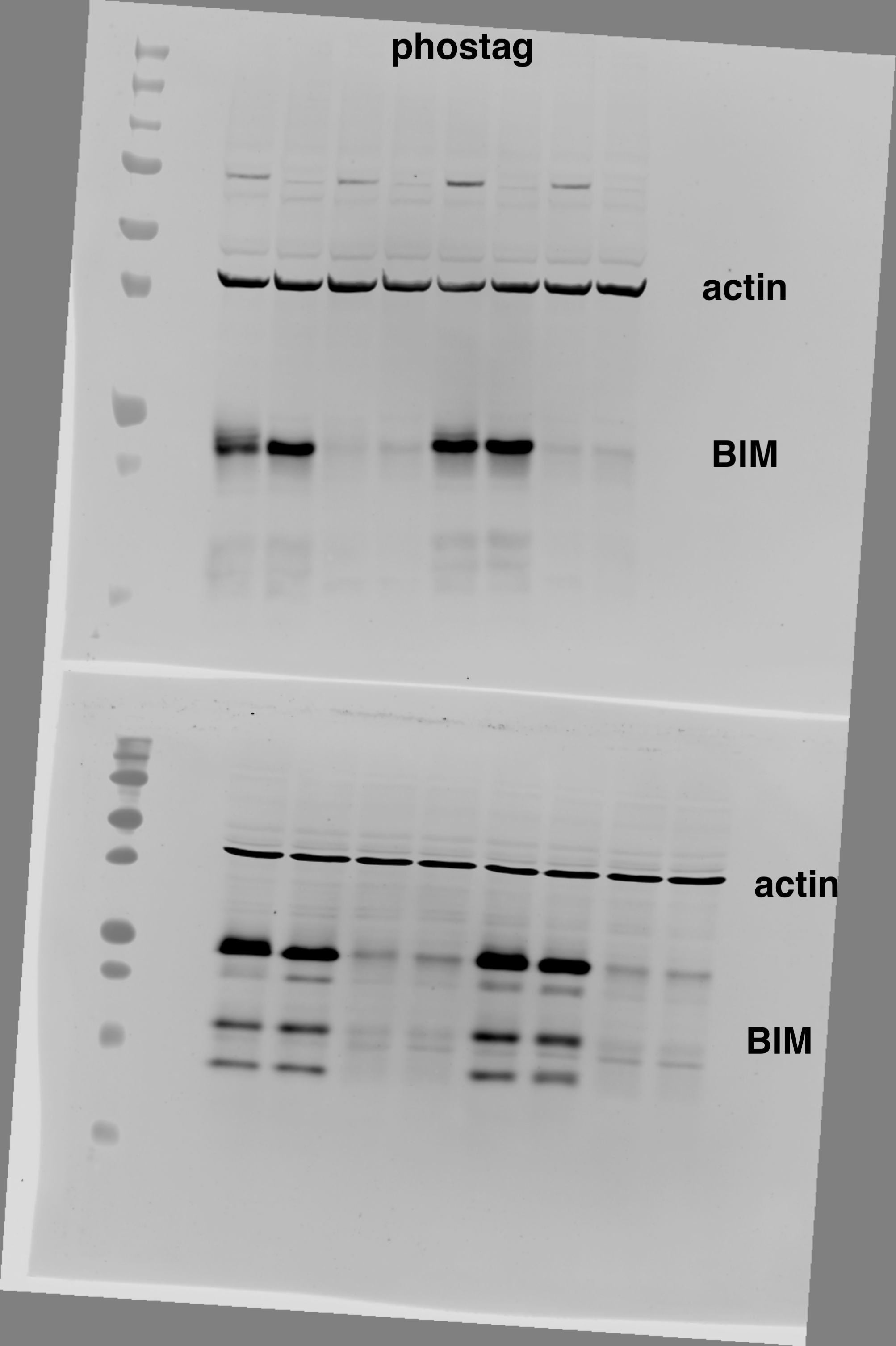

Supplement: Figure 2—source data 1. [file elife-82860-fig2-data1.zip › elife_Fig 2 source data/elife_Fig 2 source data 3/Fig_2B Source Data labeled .tif]

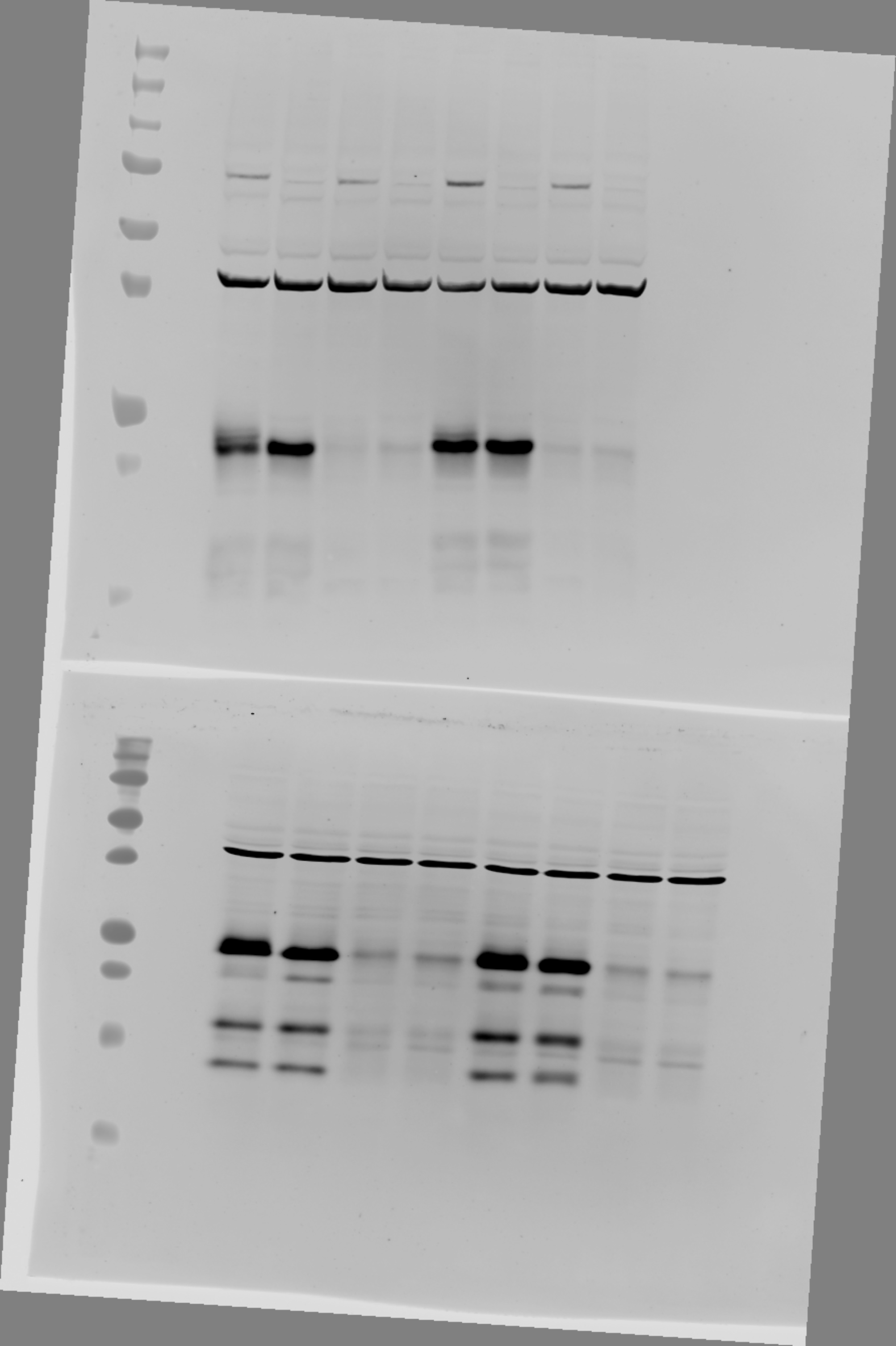

Supplement: Figure 2—source data 1. [file elife-82860-fig2-data1.zip › elife_Fig 2 source data/elife_Fig 2 source data 3/Fig_2B Source Data Unlabeled .tif]

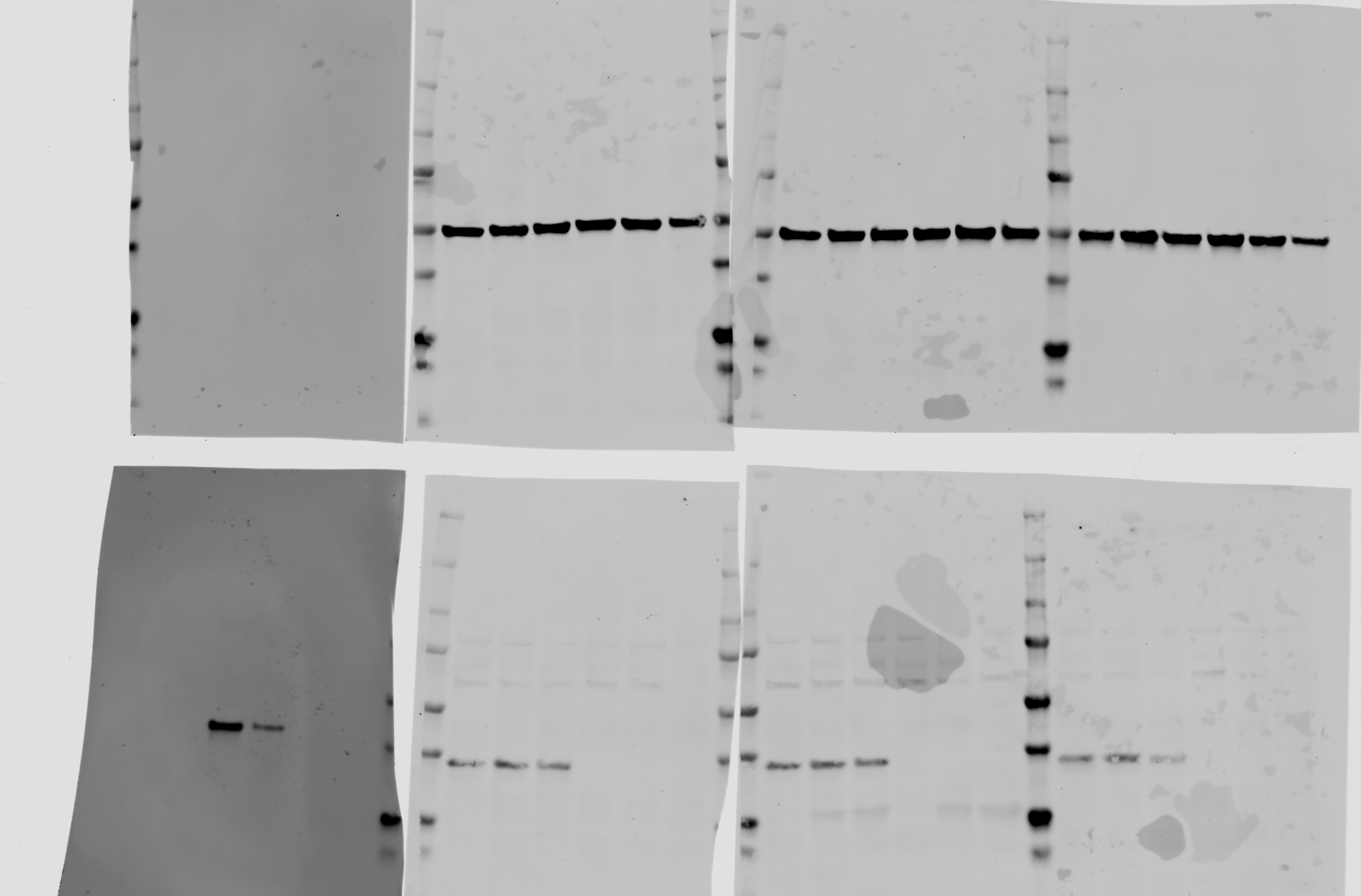

Supplement: Figure 2—source data 1. [file elife-82860-fig2-data1.zip › elife_Fig 2 source data/elife_Fig 2 source data 5/Fig_2F_Source_Data_Unlabeled/Fig_2F Source Data 2 unlabeled.tif]

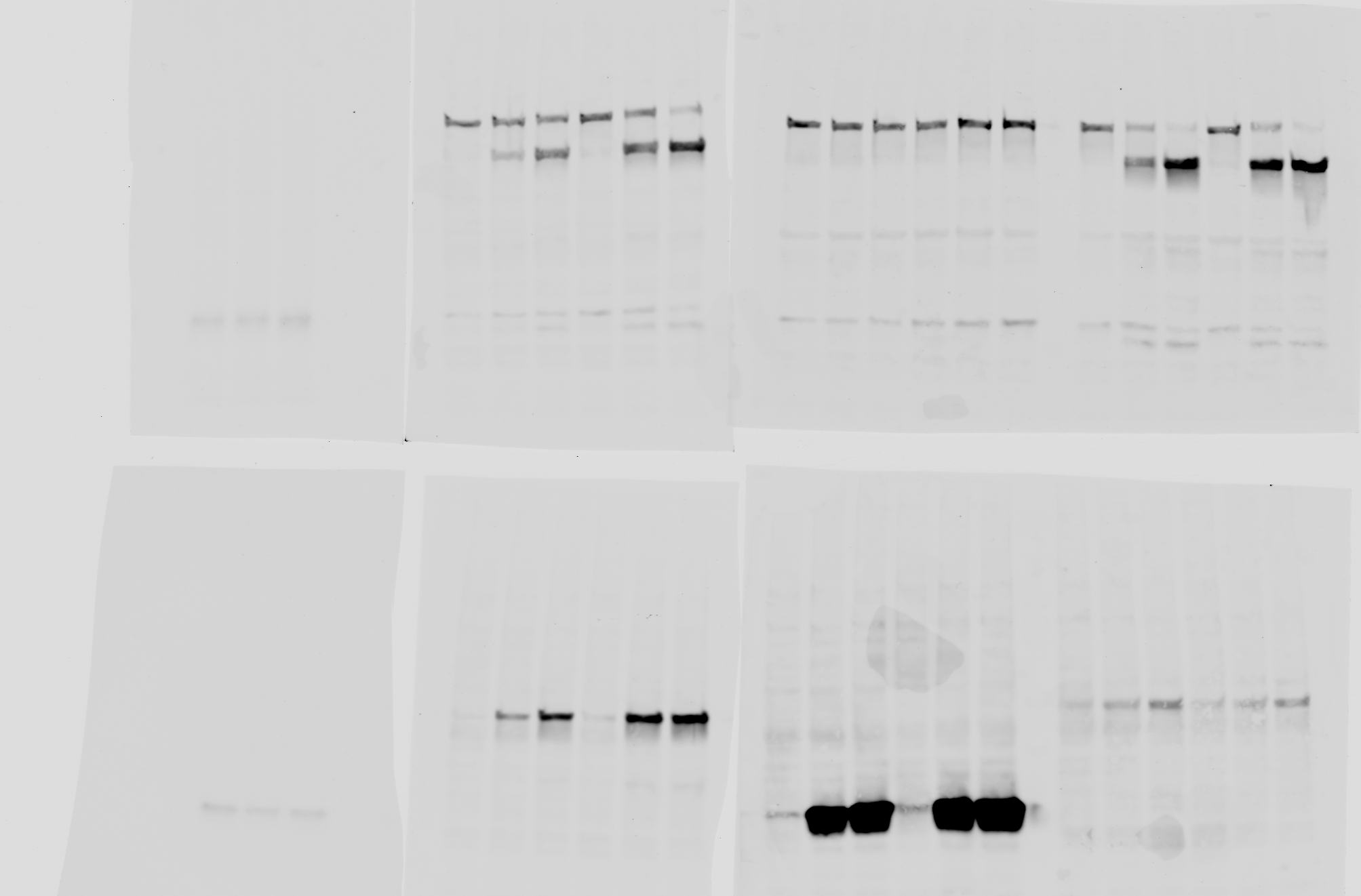

Supplement: Figure 2—source data 1. [file elife-82860-fig2-data1.zip › elife_Fig 2 source data/elife_Fig 2 source data 5/Fig_2F_Source_Data_Unlabeled/Fig_2F Source Data 1 unlabeled.tif]

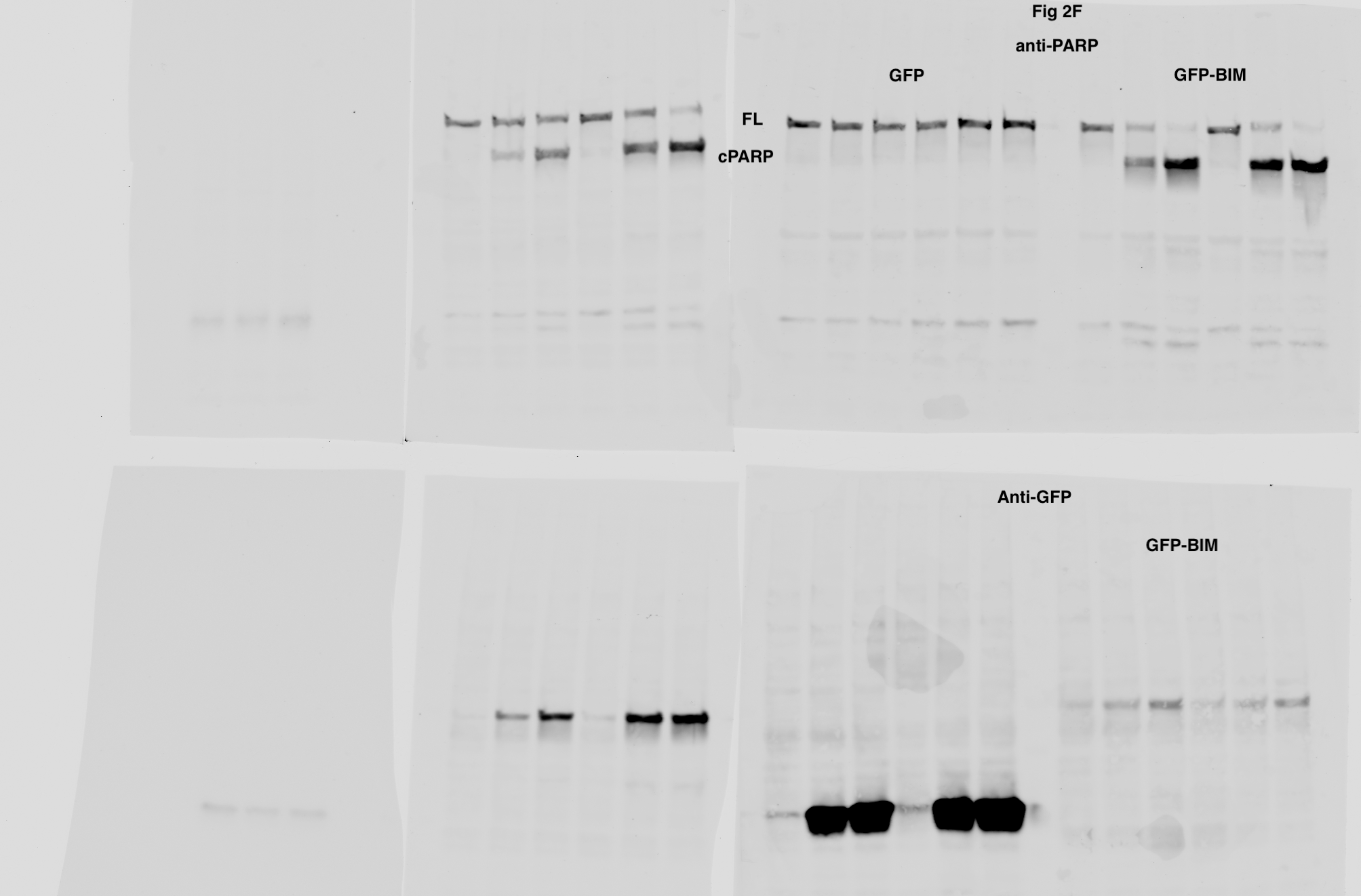

Supplement: Figure 2—source data 1. [file elife-82860-fig2-data1.zip › elife_Fig 2 source data/elife_Fig 2 source data 5/Fig_2F_Source_Data_Labeled/Fig_2F Source Data 1 labeled.tif]

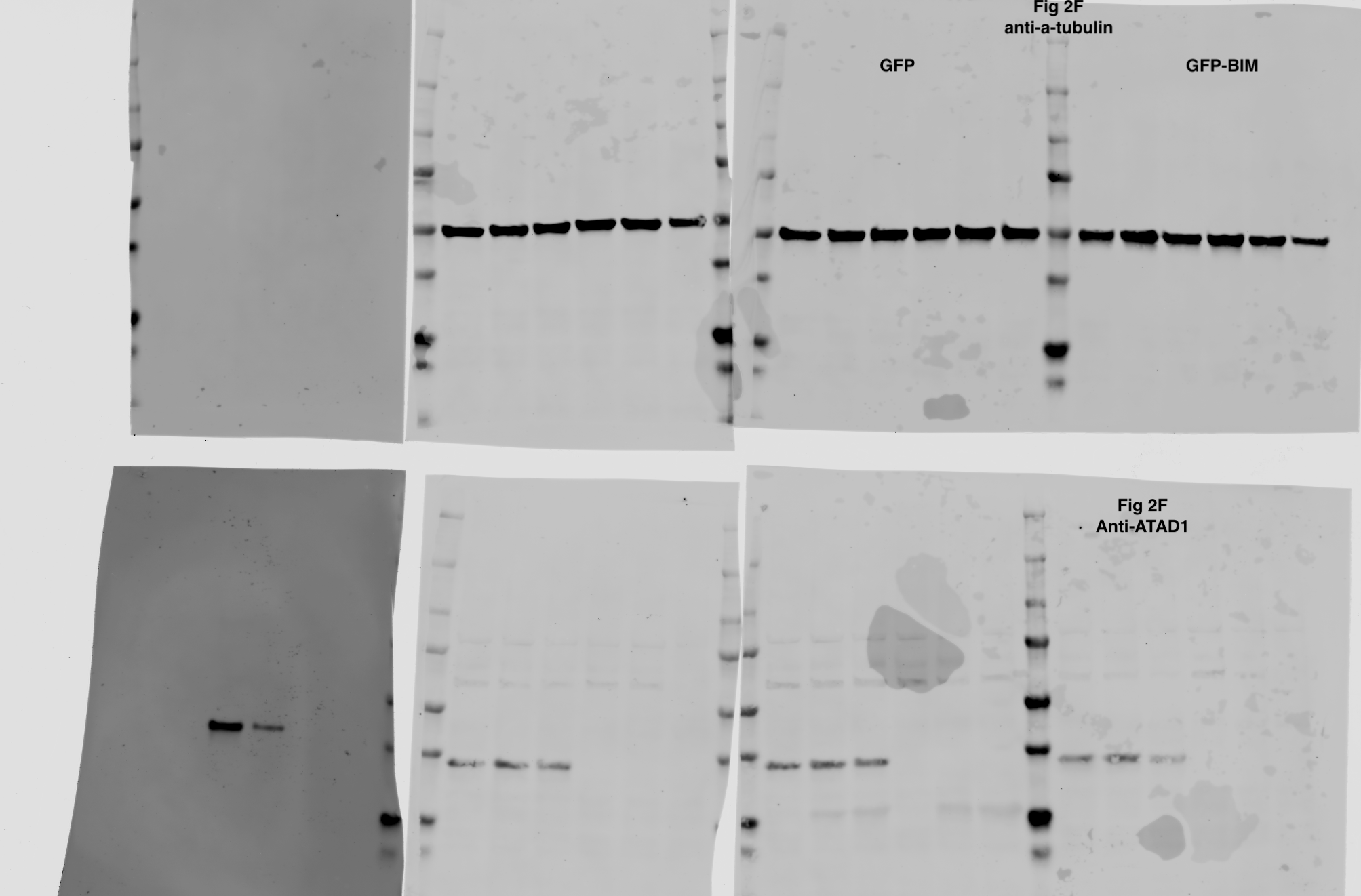

Supplement: Figure 2—source data 1. [file elife-82860-fig2-data1.zip › elife_Fig 2 source data/elife_Fig 2 source data 5/Fig_2F_Source_Data_Labeled/Fig_2F Source Data 2 labeled.tif]

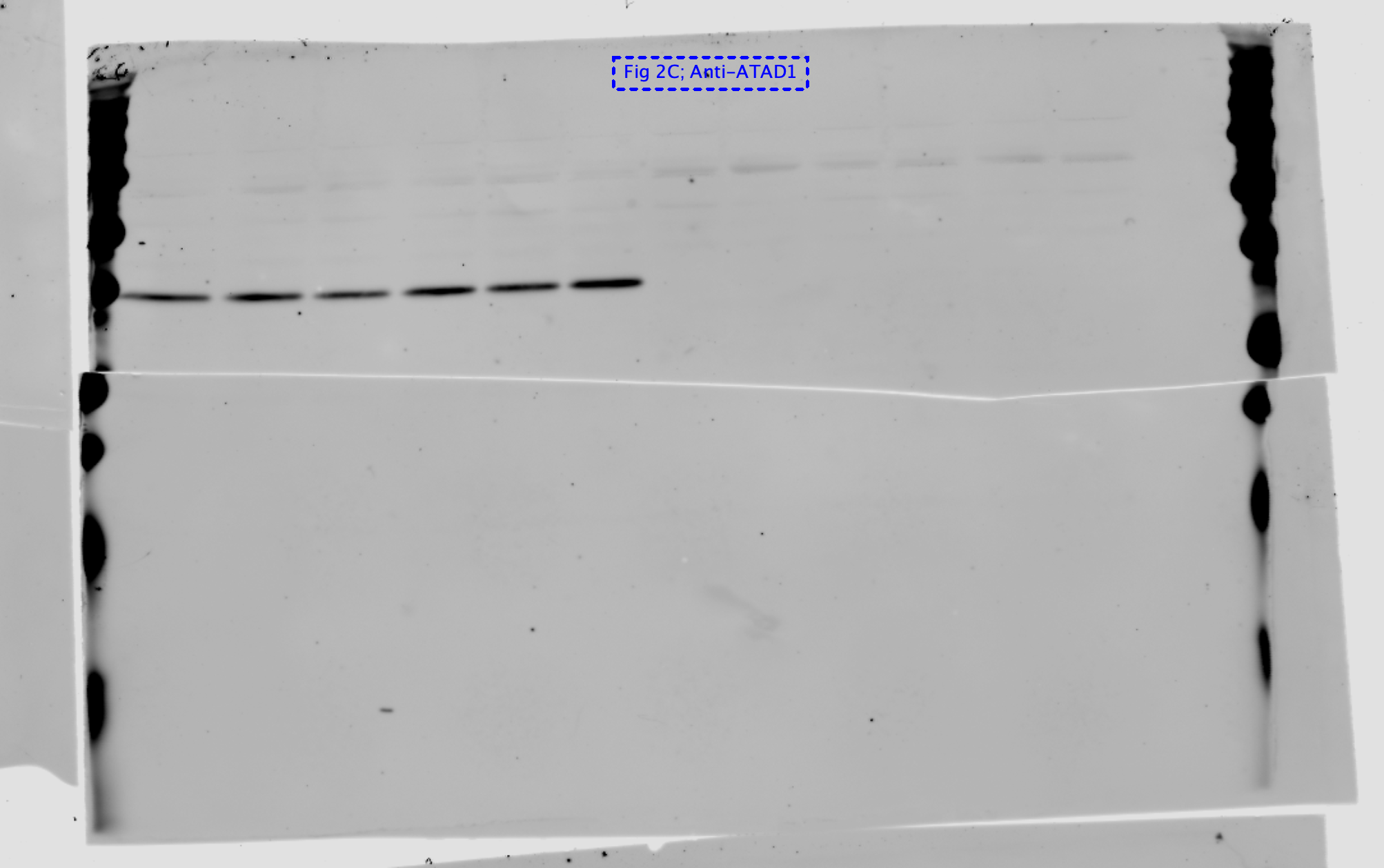

Supplement: Figure 2—source data 1. [file elife-82860-fig2-data1.zip › elife_Fig 2 source data/elife_Fig 2 source data 4/Fig_2C_Source_Data_Labeled/Fig_2C_ATAD1_labeled.tif]

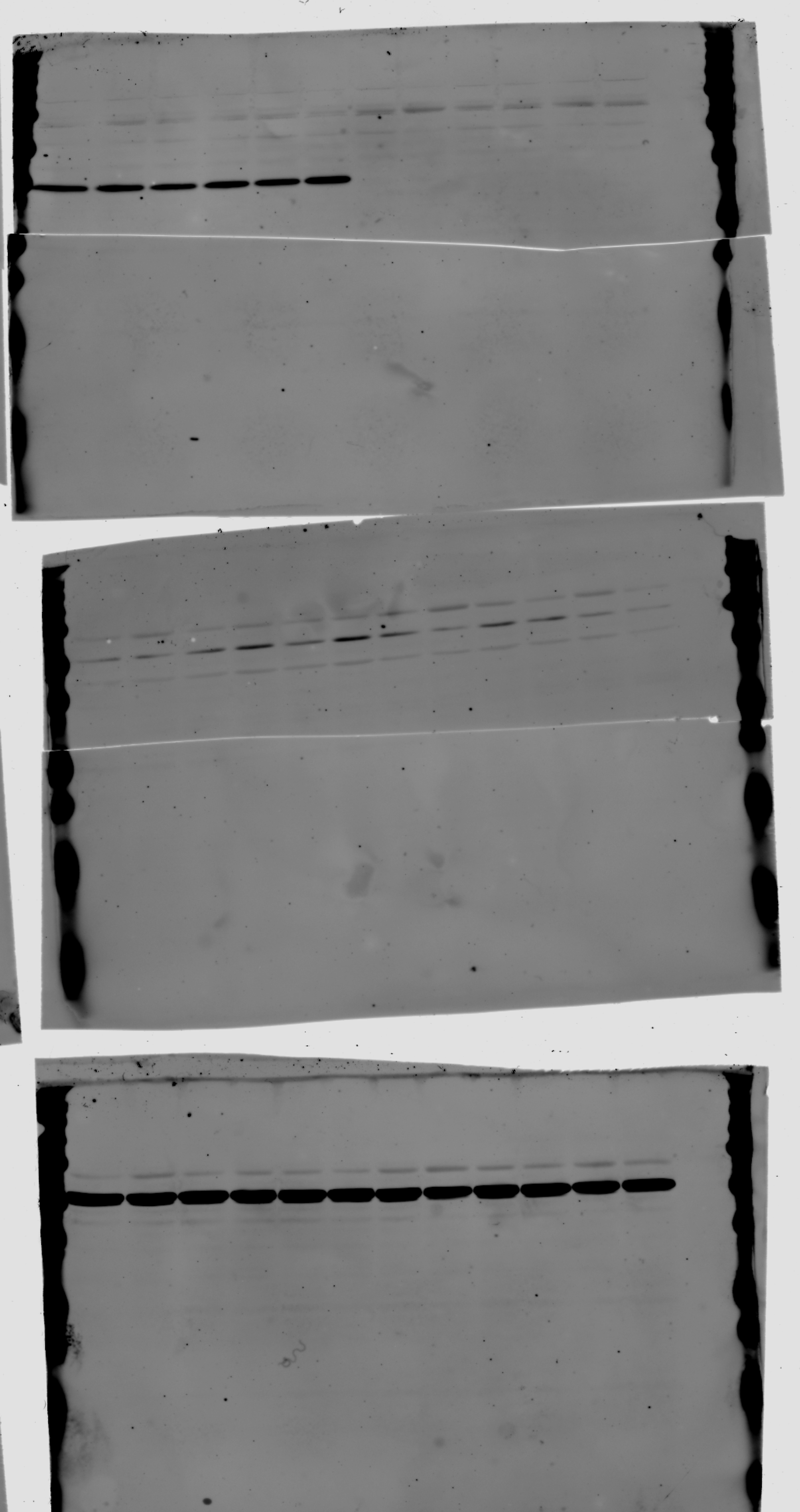

Supplement: Figure 2—source data 1. [file elife-82860-fig2-data1.zip › elife_Fig 2 source data/elife_Fig 2 source data 4/Fig_2C_Source_Data_Labeled/Fig_2C_ATAD1_Tubulin_Unlabeled.tif]

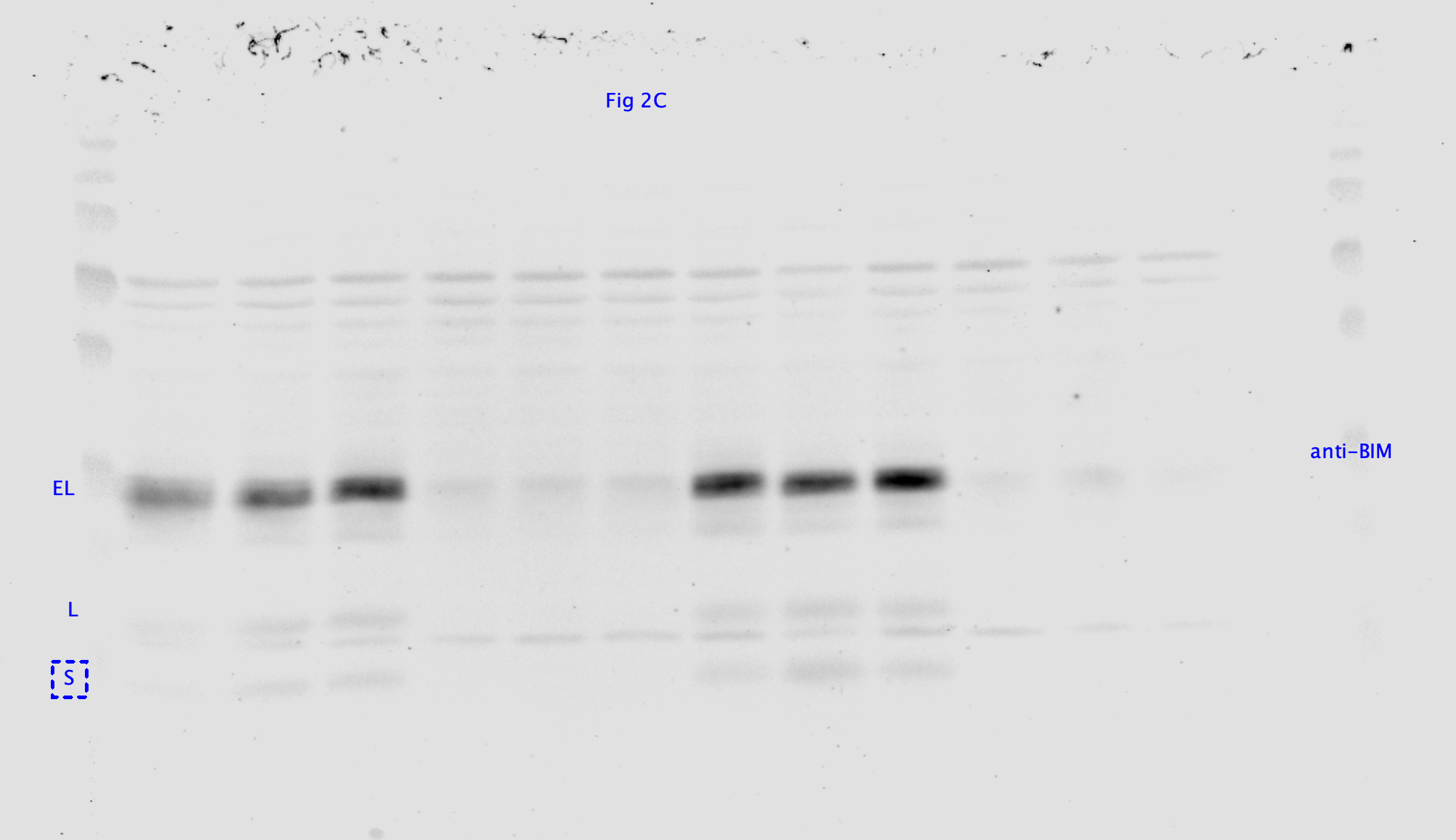

Supplement: Figure 2—source data 1. [file elife-82860-fig2-data1.zip › elife_Fig 2 source data/elife_Fig 2 source data 4/Fig_2C_Source_Data_Labeled/Fig_2C_BIM_labeled.tif]

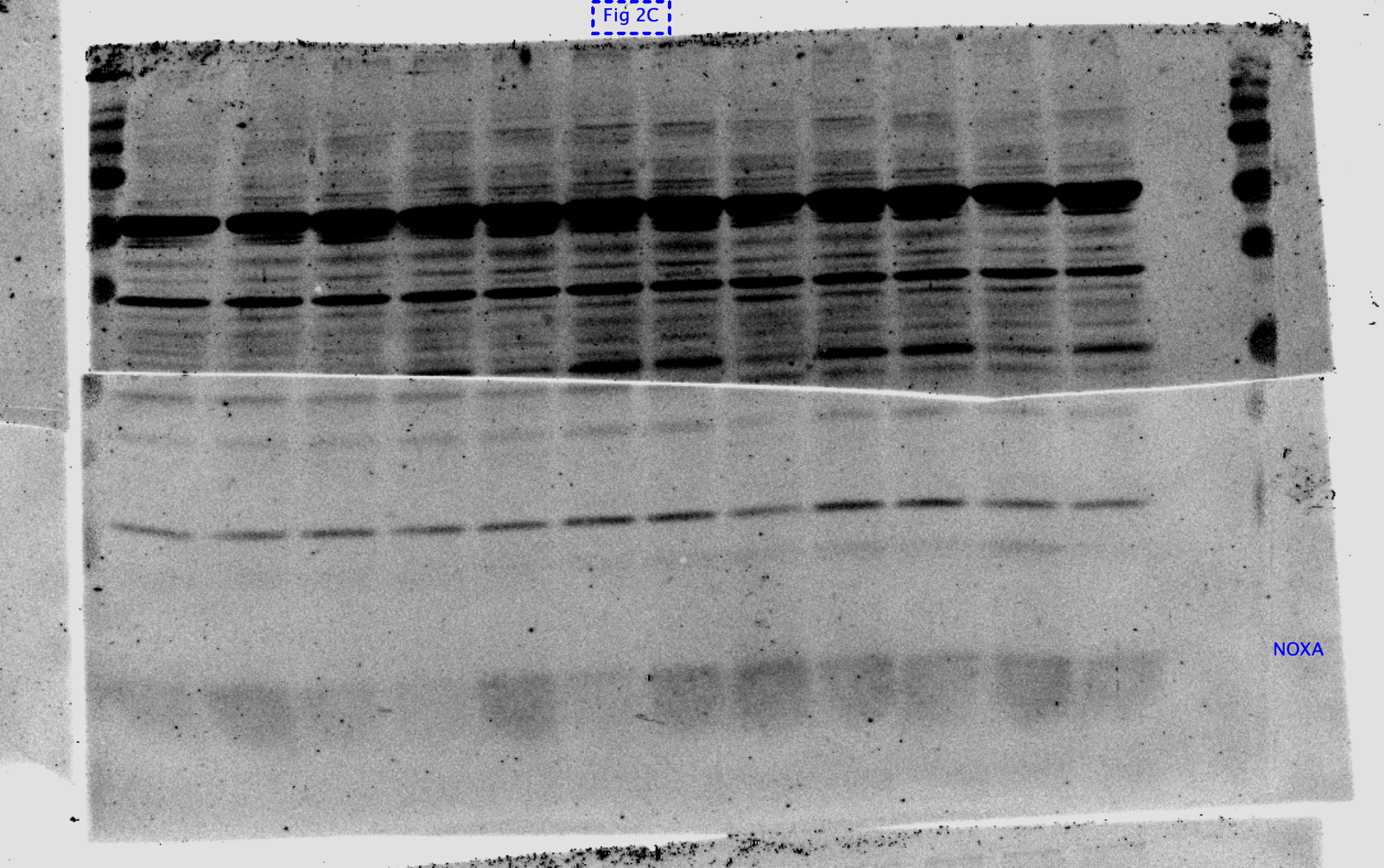

Supplement: Figure 2—source data 1. [file elife-82860-fig2-data1.zip › elife_Fig 2 source data/elife_Fig 2 source data 4/Fig_2C_Source_Data_Labeled/Fig_2C_NOXA_labeled.tif]

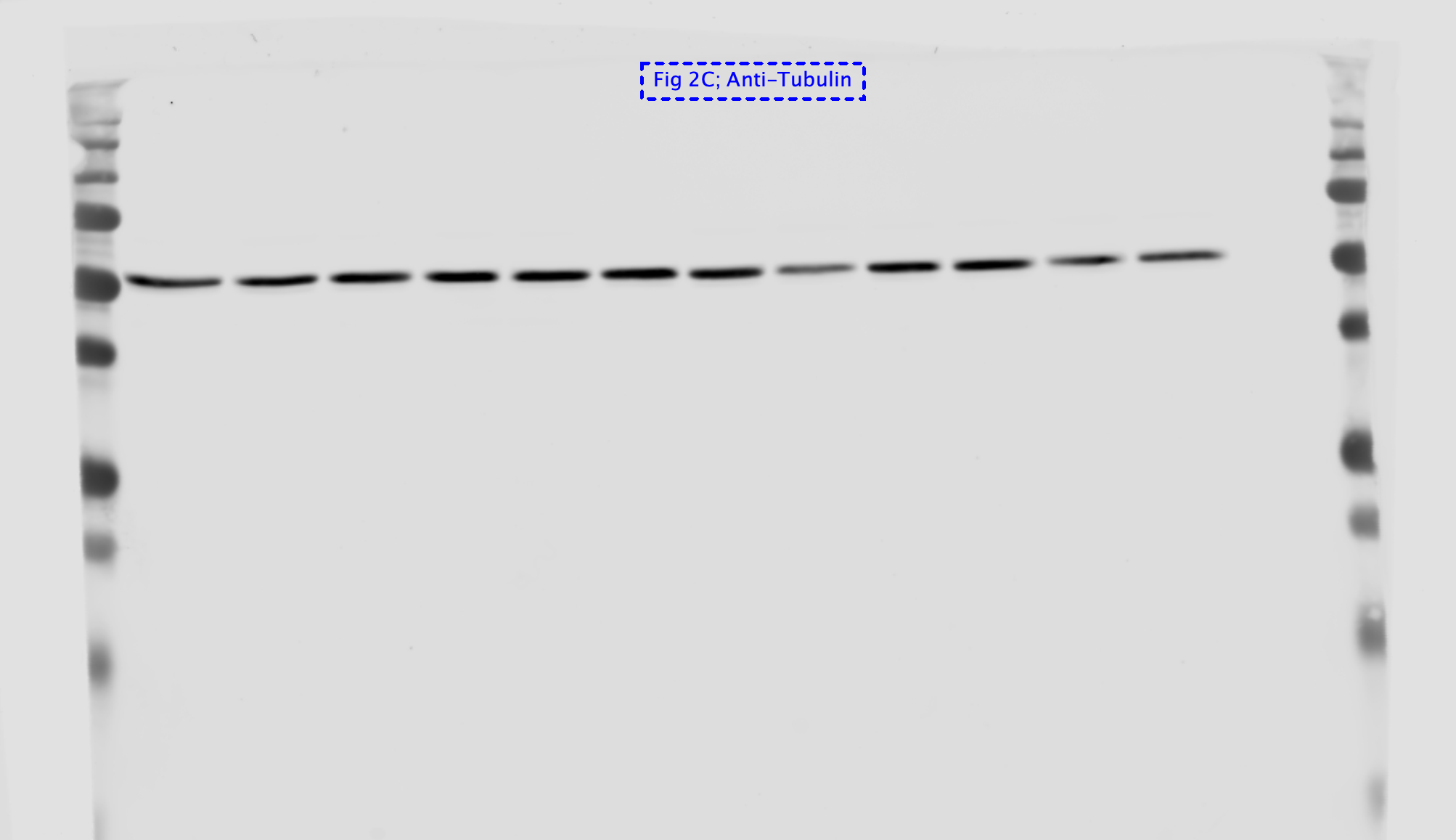

Supplement: Figure 2—source data 1. [file elife-82860-fig2-data1.zip › elife_Fig 2 source data/elife_Fig 2 source data 4/Fig_2C_Source_Data_Labeled/Fig_2C_Tubulin_labeled.tif]

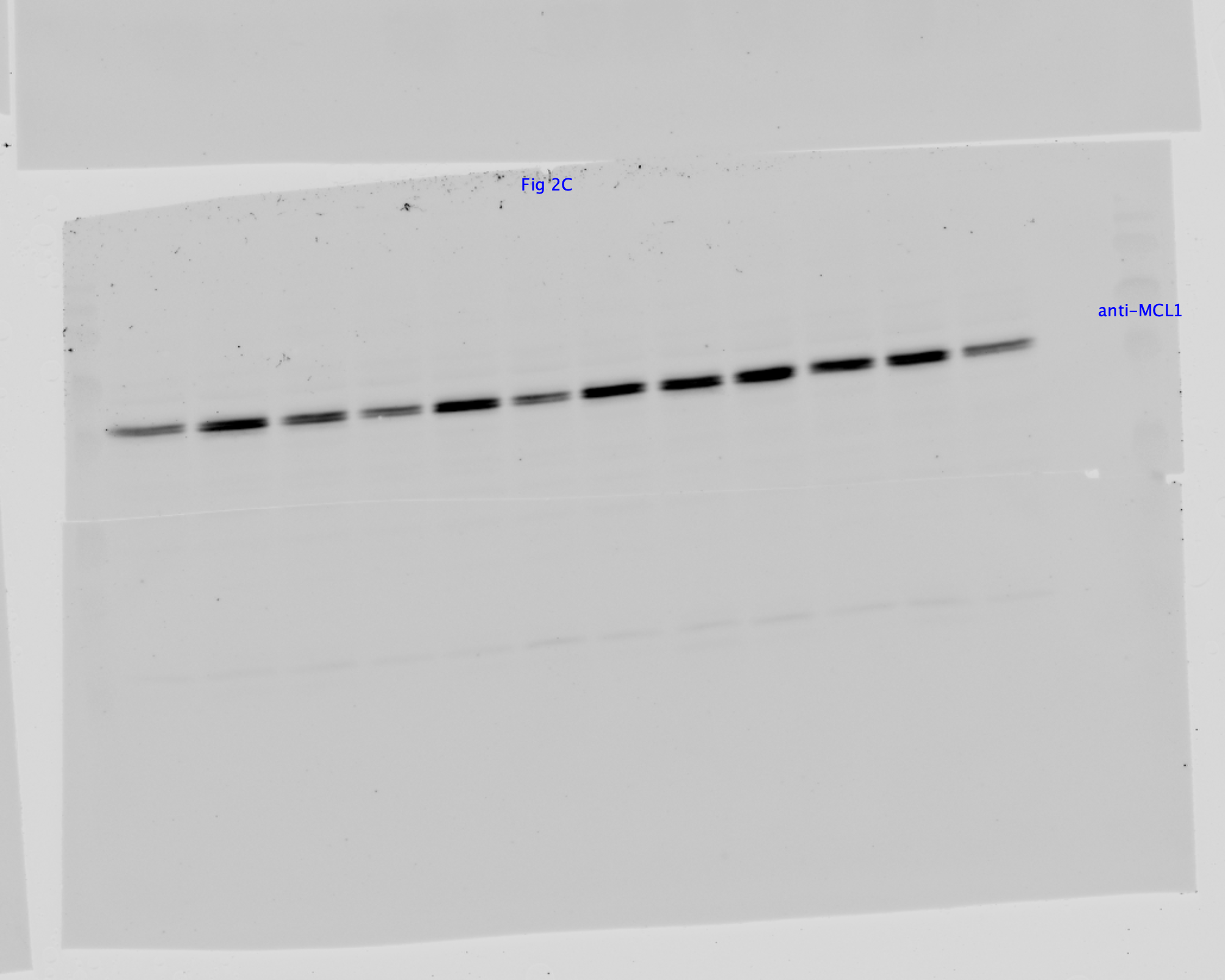

Supplement: Figure 2—source data 1. [file elife-82860-fig2-data1.zip › elife_Fig 2 source data/elife_Fig 2 source data 4/Fig_2C_Source_Data_Labeled/Fig_2C_MCL1_labeled.tif]

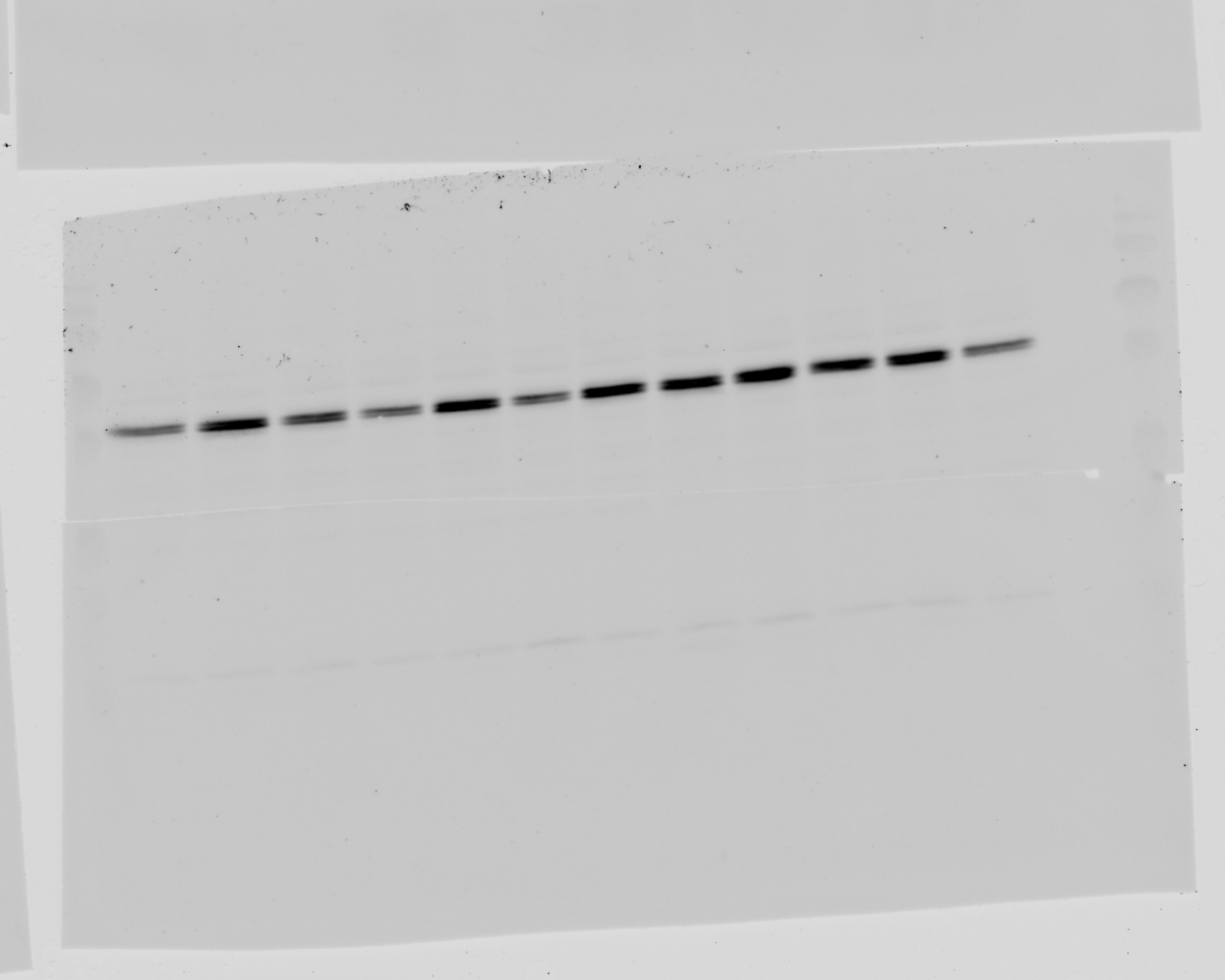

Supplement: Figure 2—source data 1. [file elife-82860-fig2-data1.zip › elife_Fig 2 source data/elife_Fig 2 source data 4/Fig_2C_Source_Data_Unlabeled/Fig_2C_MCL1_Unlabeled.tif]

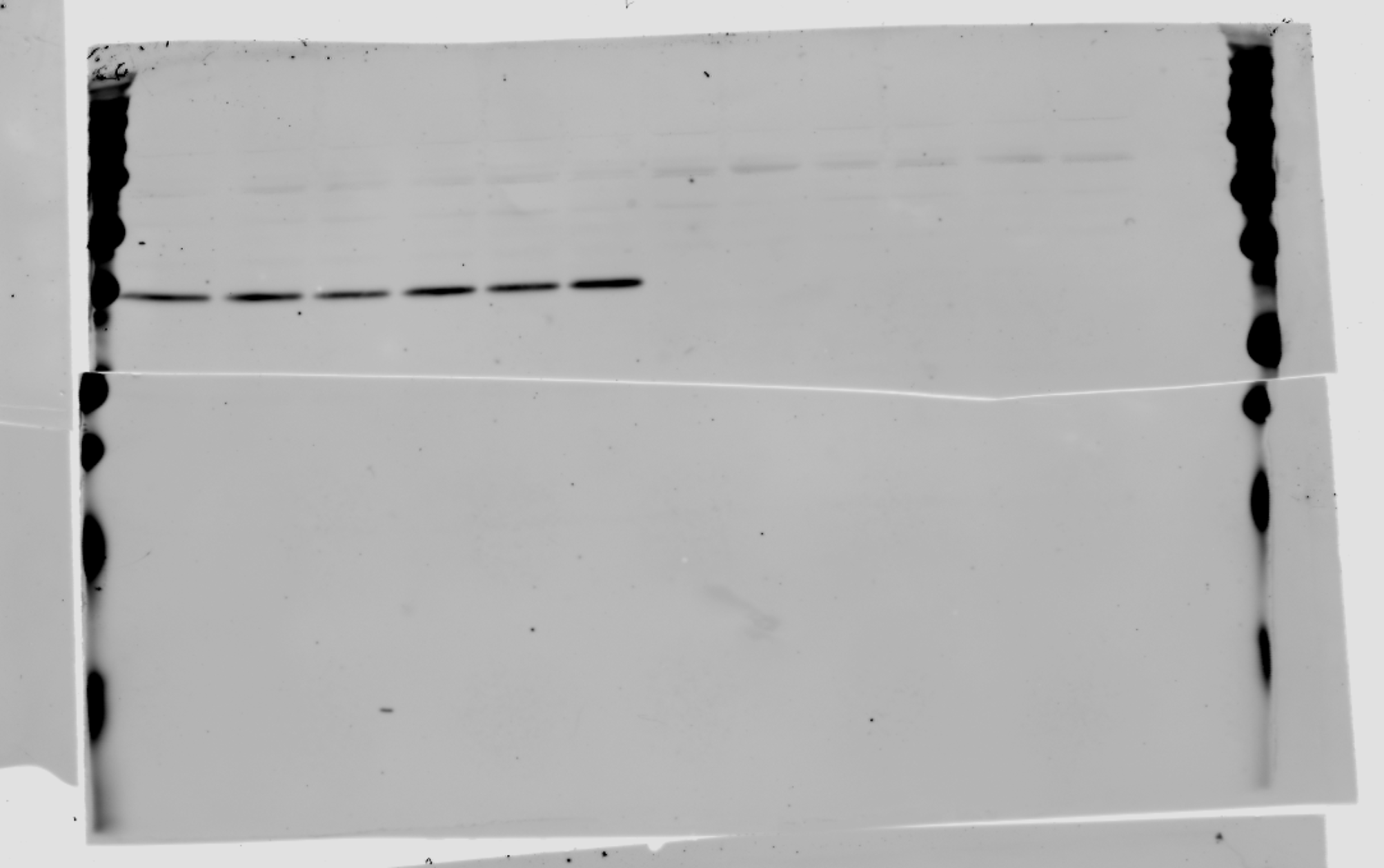

Supplement: Figure 2—source data 1. [file elife-82860-fig2-data1.zip › elife_Fig 2 source data/elife_Fig 2 source data 4/Fig_2C_Source_Data_Unlabeled/Fig_2C_ATAD1_unlabeled.tif]

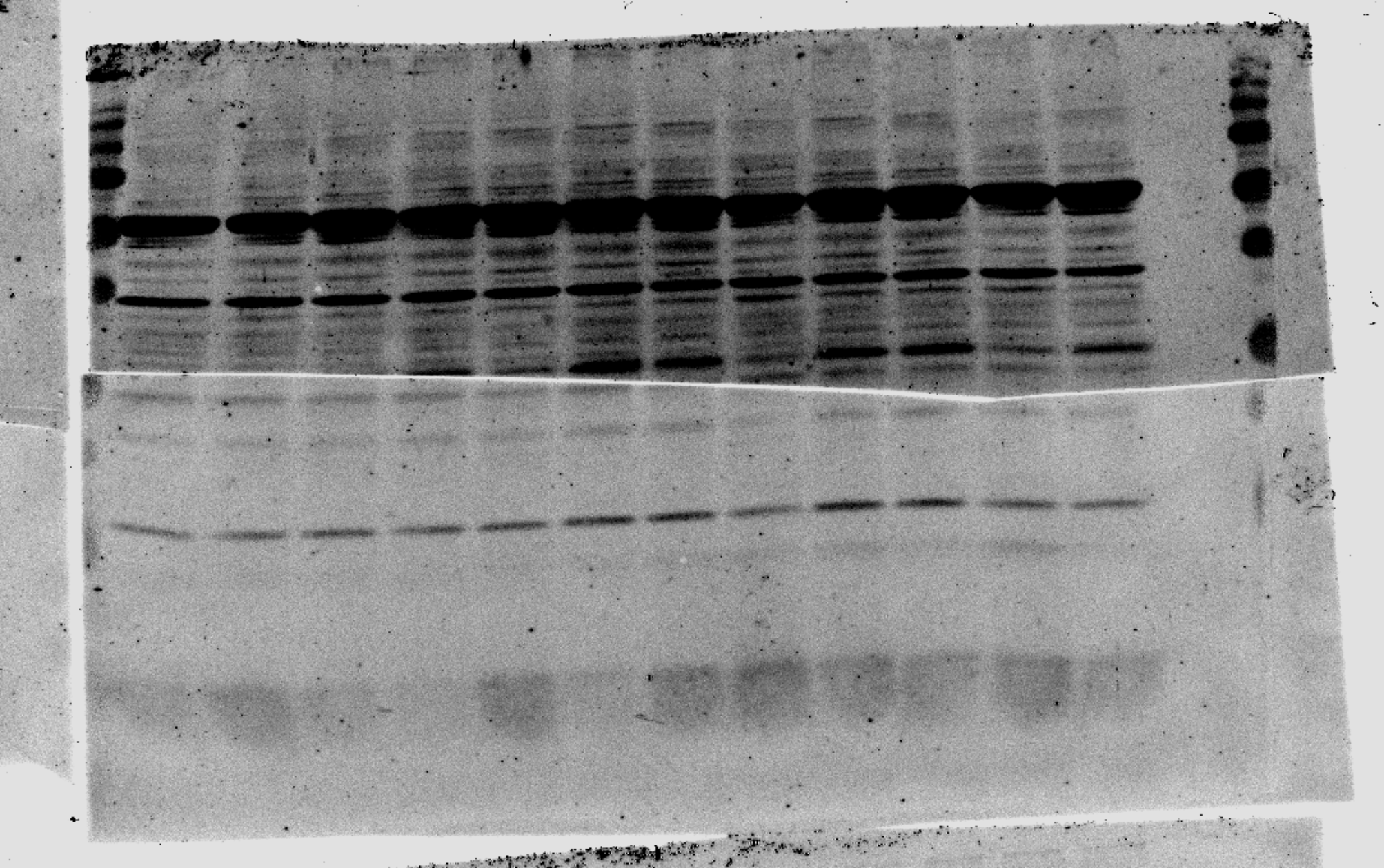

Supplement: Figure 2—source data 1. [file elife-82860-fig2-data1.zip › elife_Fig 2 source data/elife_Fig 2 source data 4/Fig_2C_Source_Data_Unlabeled/Fig_2C_NOXA_unlabeled.tif]

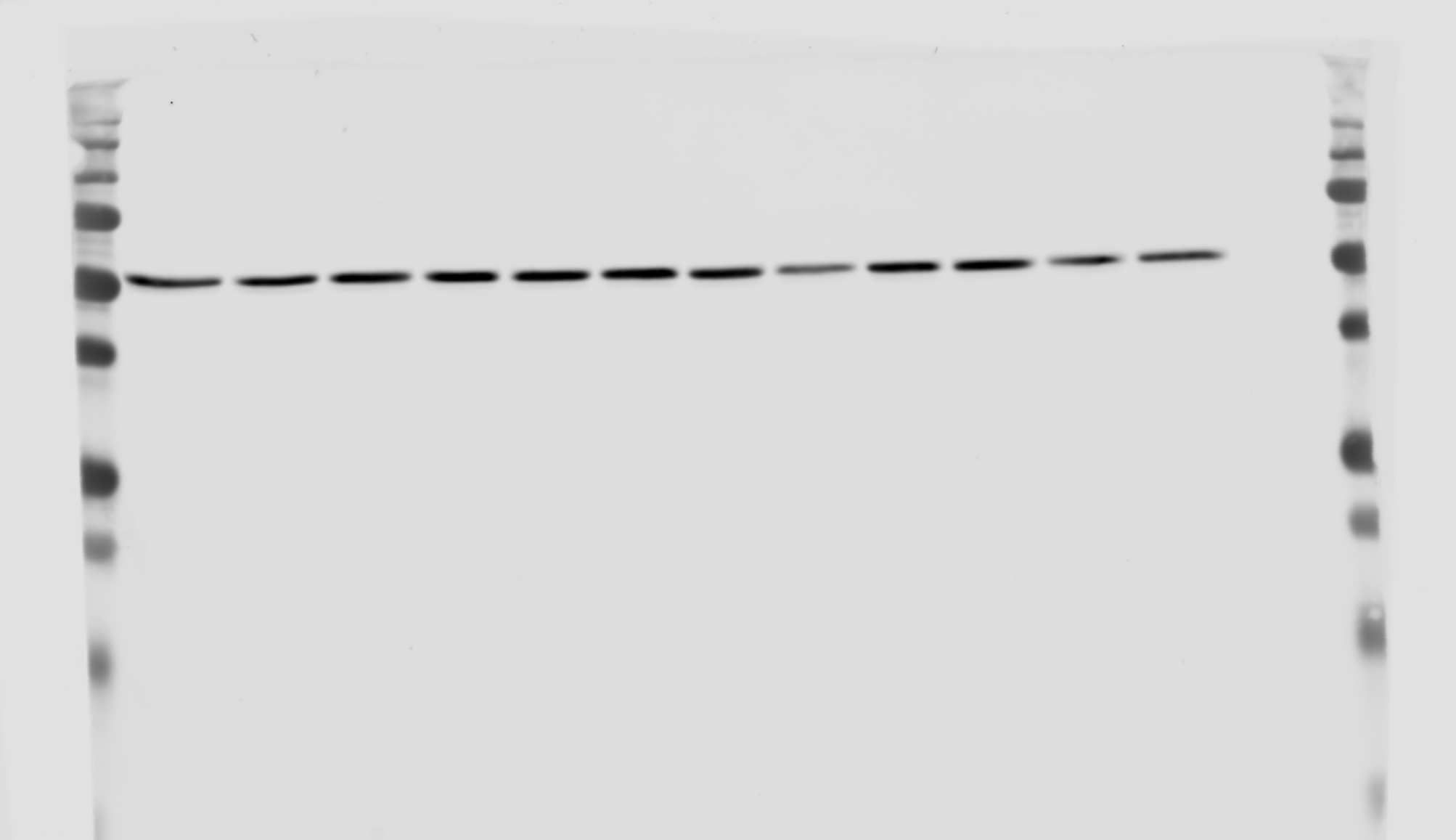

Supplement: Figure 2—source data 1. [file elife-82860-fig2-data1.zip › elife_Fig 2 source data/elife_Fig 2 source data 4/Fig_2C_Source_Data_Unlabeled/Fig_2C_Tubulin_unlabeled.tif]

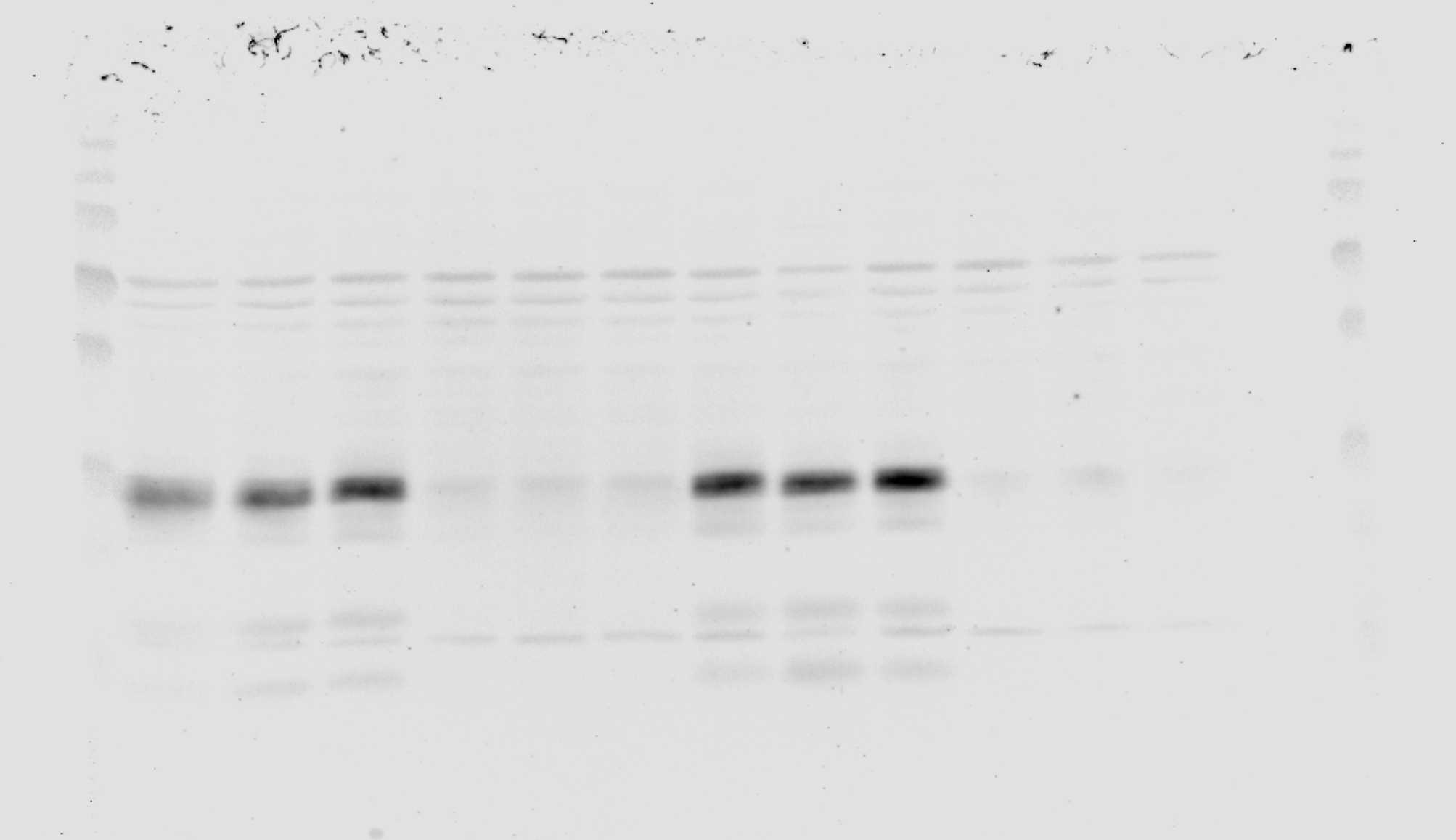

Supplement: Figure 2—source data 1. [file elife-82860-fig2-data1.zip › elife_Fig 2 source data/elife_Fig 2 source data 4/Fig_2C_Source_Data_Unlabeled/Fig_2C_BIM_Unlabeled.tif]

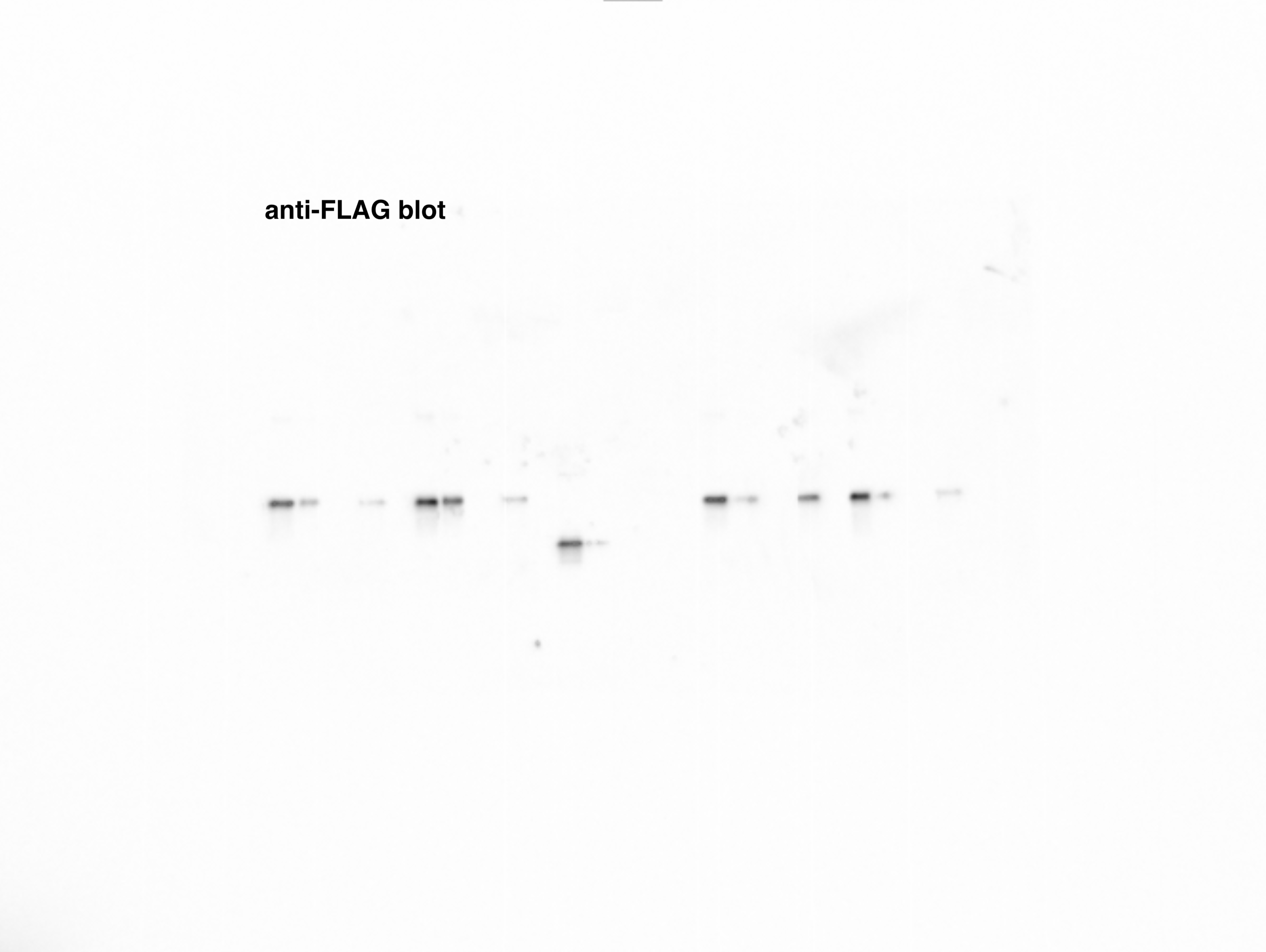

Supplement: Figure 2—source data 1. [file elife-82860-fig2-data1.zip › elife_Fig 2 source data/elife_Fig 2 source data 6/Fig_2I_Source_Data_Labeled/Fig_2I_Anti-Flag_labeled.tif]

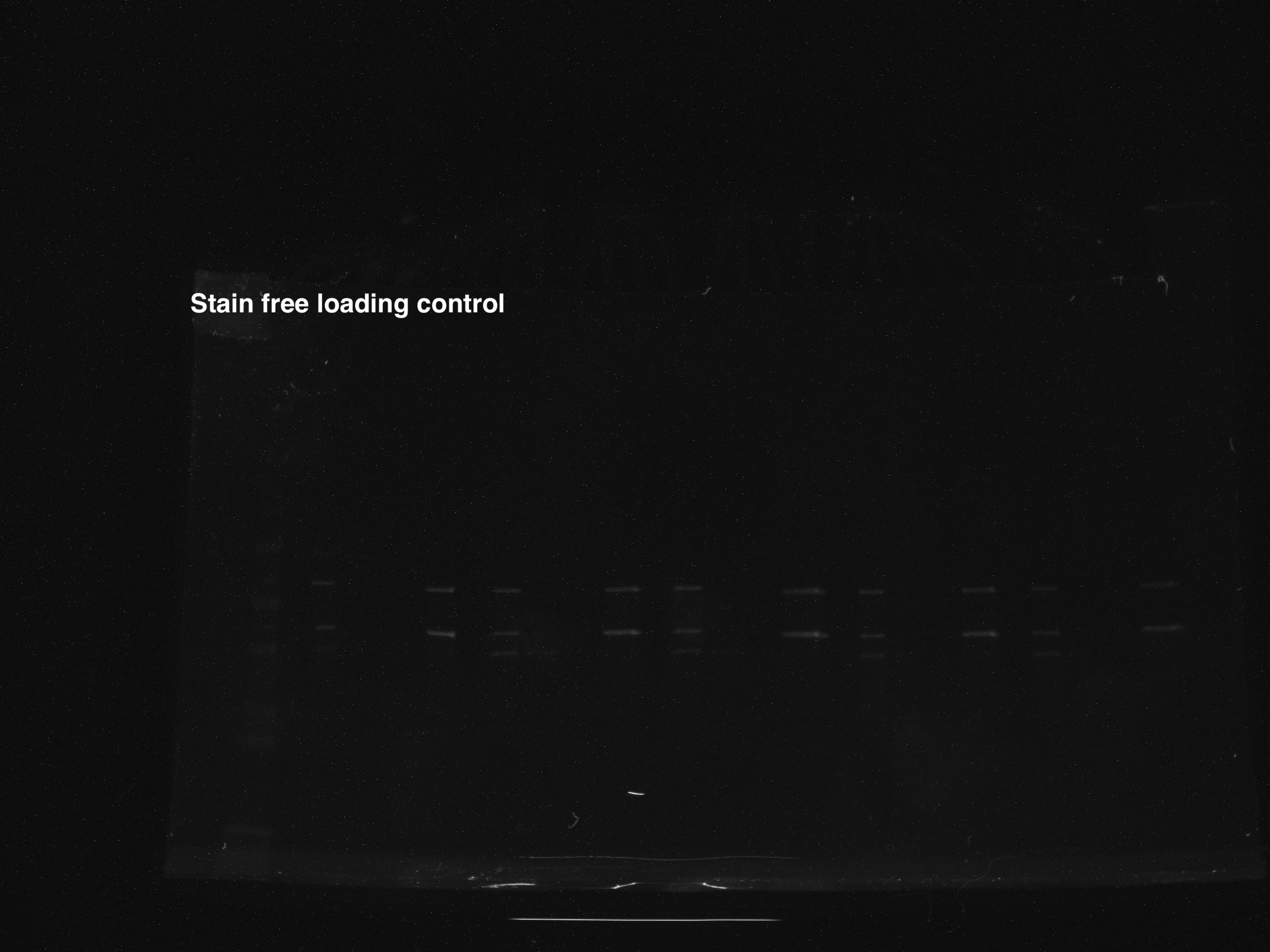

Supplement: Figure 2—source data 1. [file elife-82860-fig2-data1.zip › elife_Fig 2 source data/elife_Fig 2 source data 6/Fig_2I_Source_Data_Labeled/Fig_2I_Stain-Free labeled.jpg]

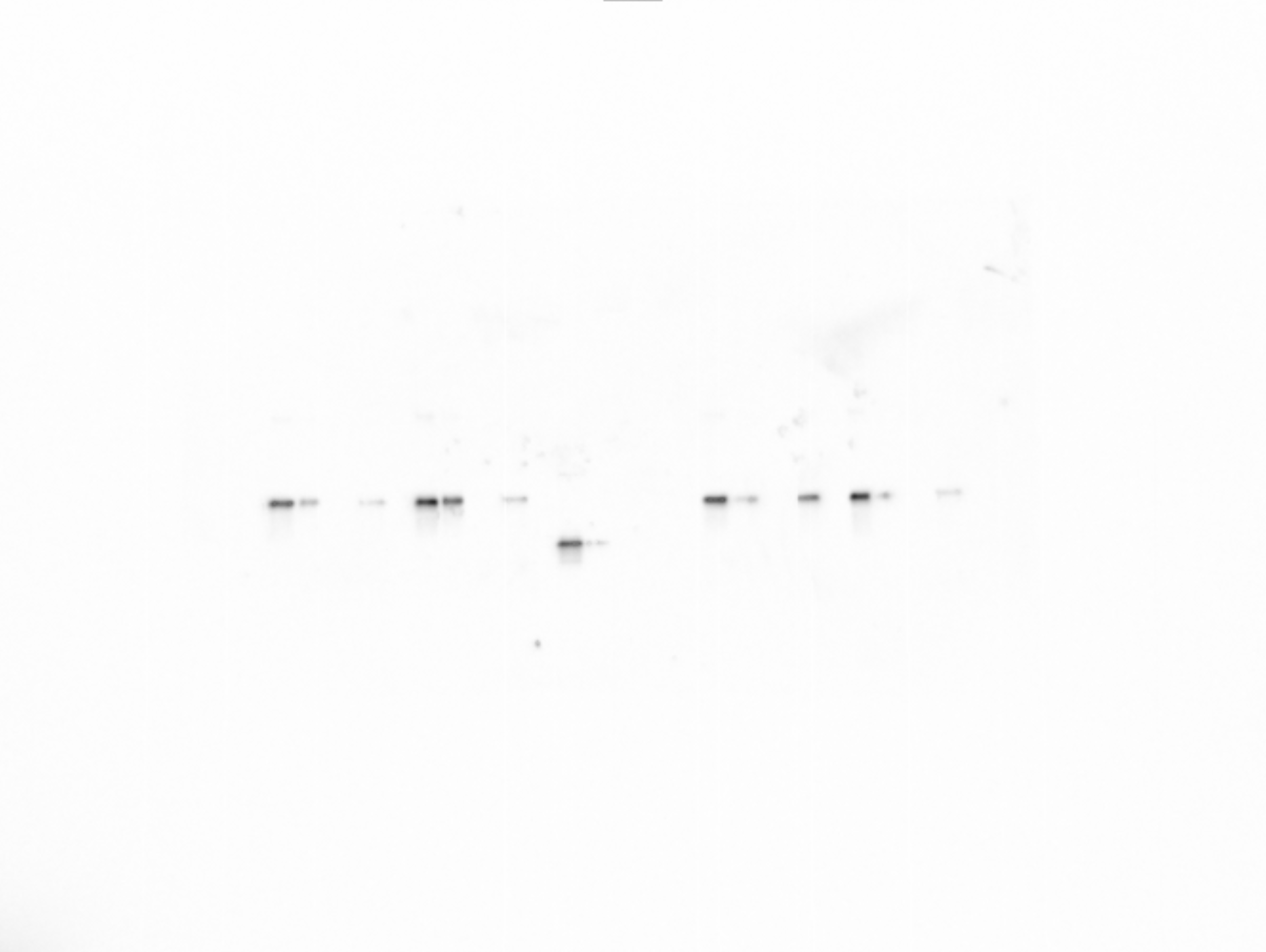

Supplement: Figure 2—source data 1. [file elife-82860-fig2-data1.zip › elife_Fig 2 source data/elife_Fig 2 source data 6/Fig_2I_Source_Data_Unlabeled/Fig_2I_Anti-Flag_unlabeled.tif]

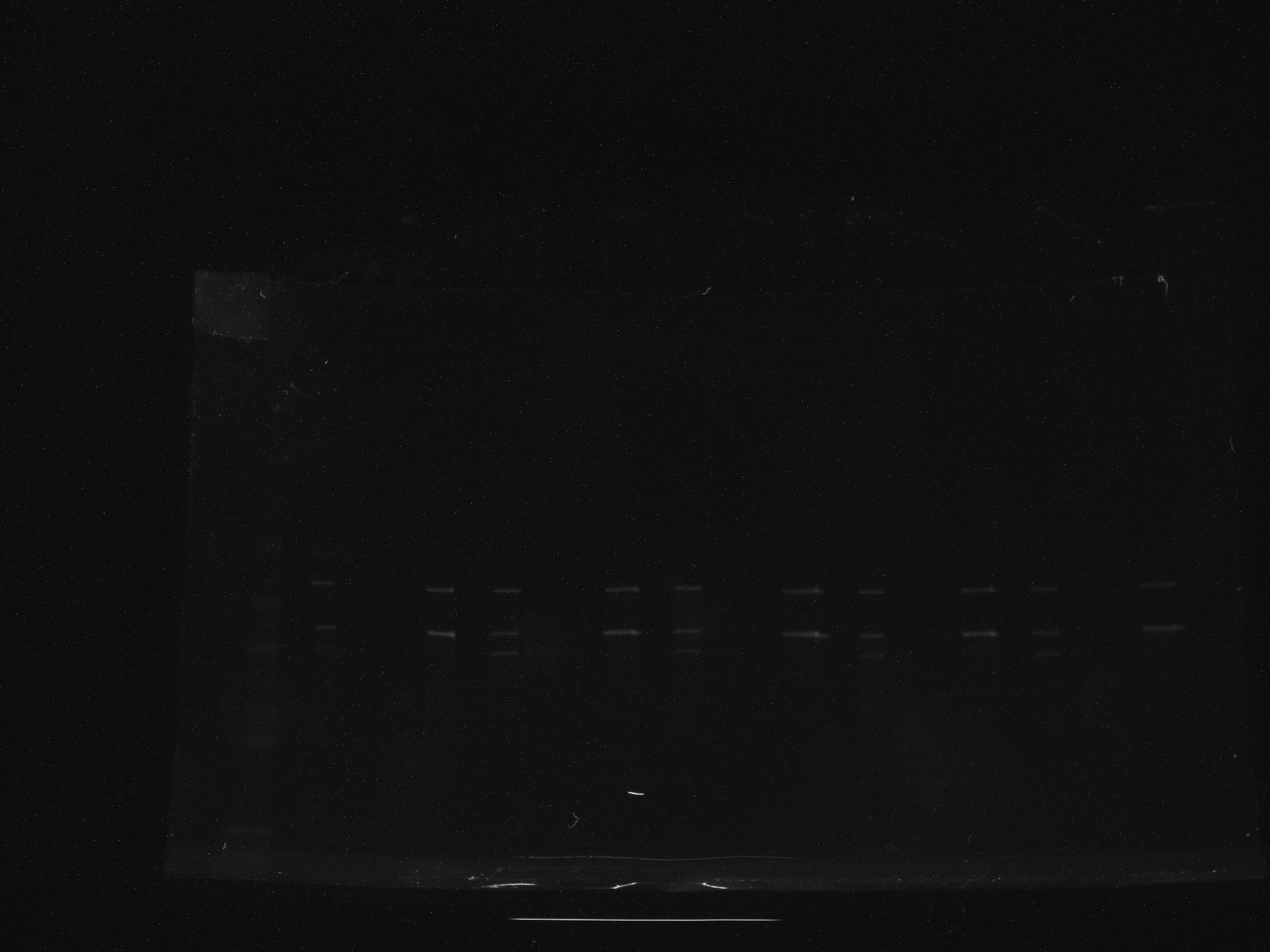

Supplement: Figure 2—source data 1. [file elife-82860-fig2-data1.zip › elife_Fig 2 source data/elife_Fig 2 source data 6/Fig_2I_Source_Data_Unlabeled/Fig_2I_Stain-Free unlabeled.jpg]

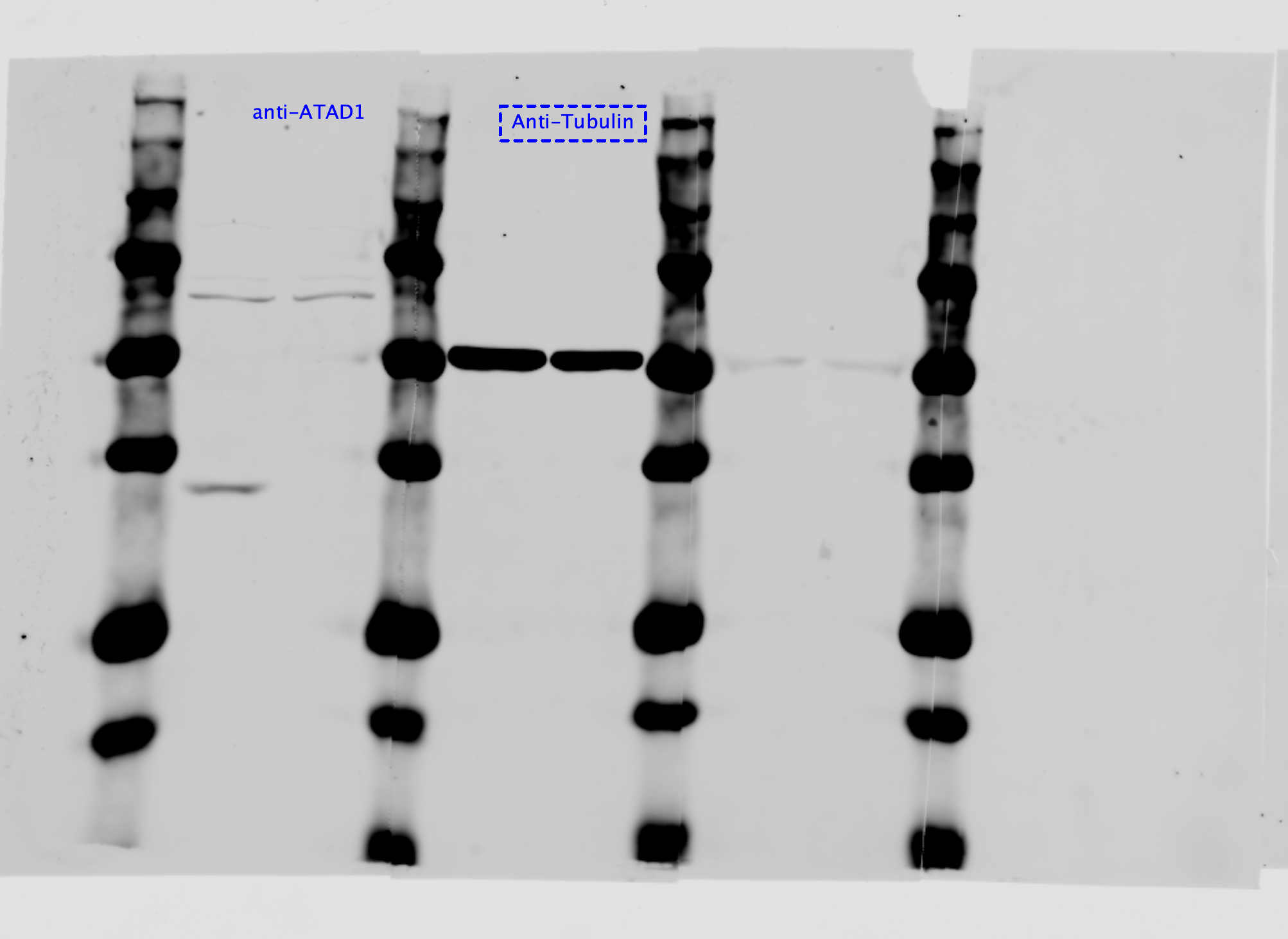

Supplement: Figure 2—source data 1. [file elife-82860-fig2-data1.zip › elife_Fig 2 source data/elife_Fig 2 source data 1/Fig_2A_Source Data_labeled/Fig_2A_ATAD1_Tub labeled.tif]

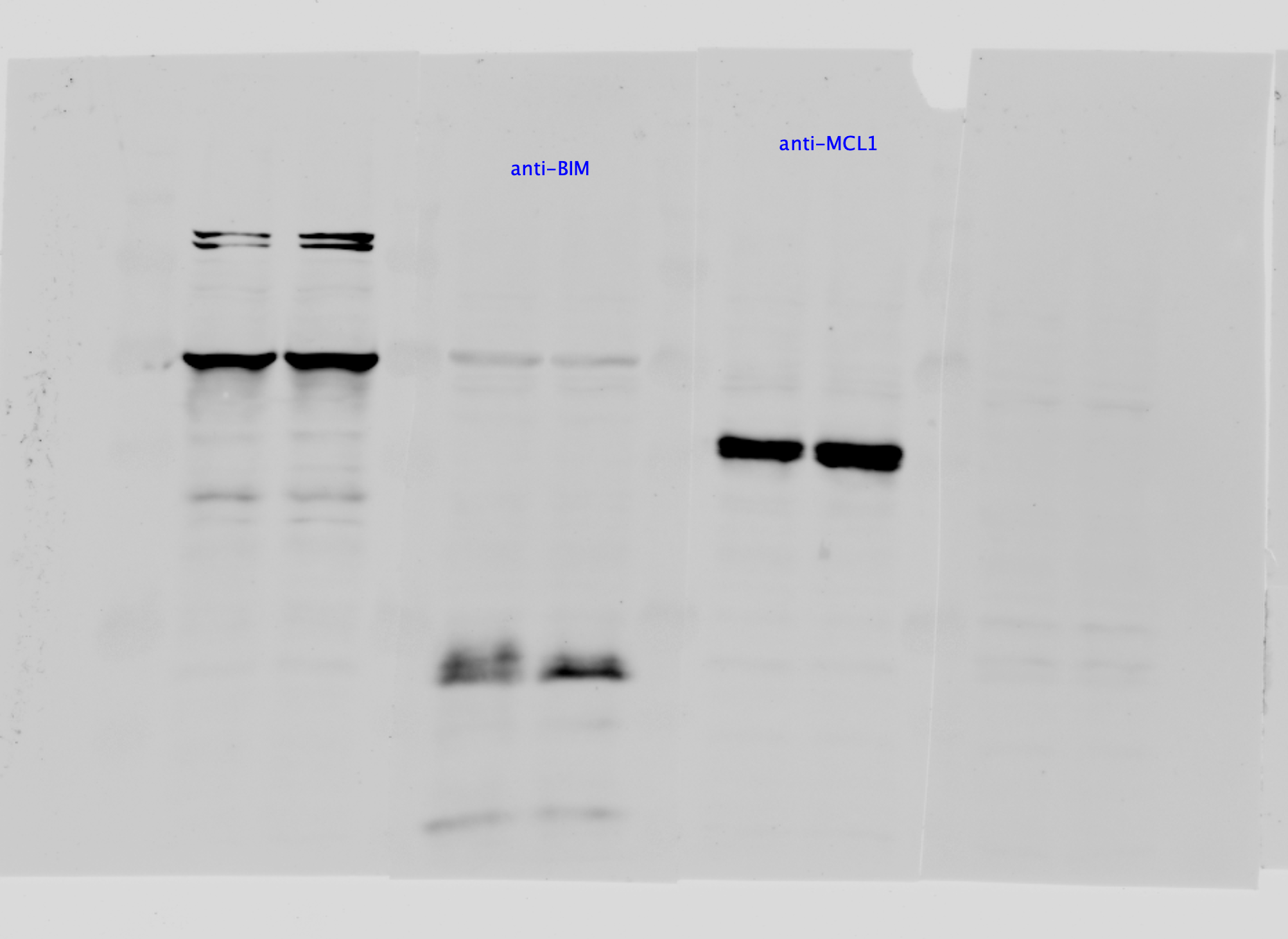

Supplement: Figure 2—source data 1. [file elife-82860-fig2-data1.zip › elife_Fig 2 source data/elife_Fig 2 source data 1/Fig_2A_Source Data_labeled/Fig_2A_BIM_MCL1 labeled.tif]

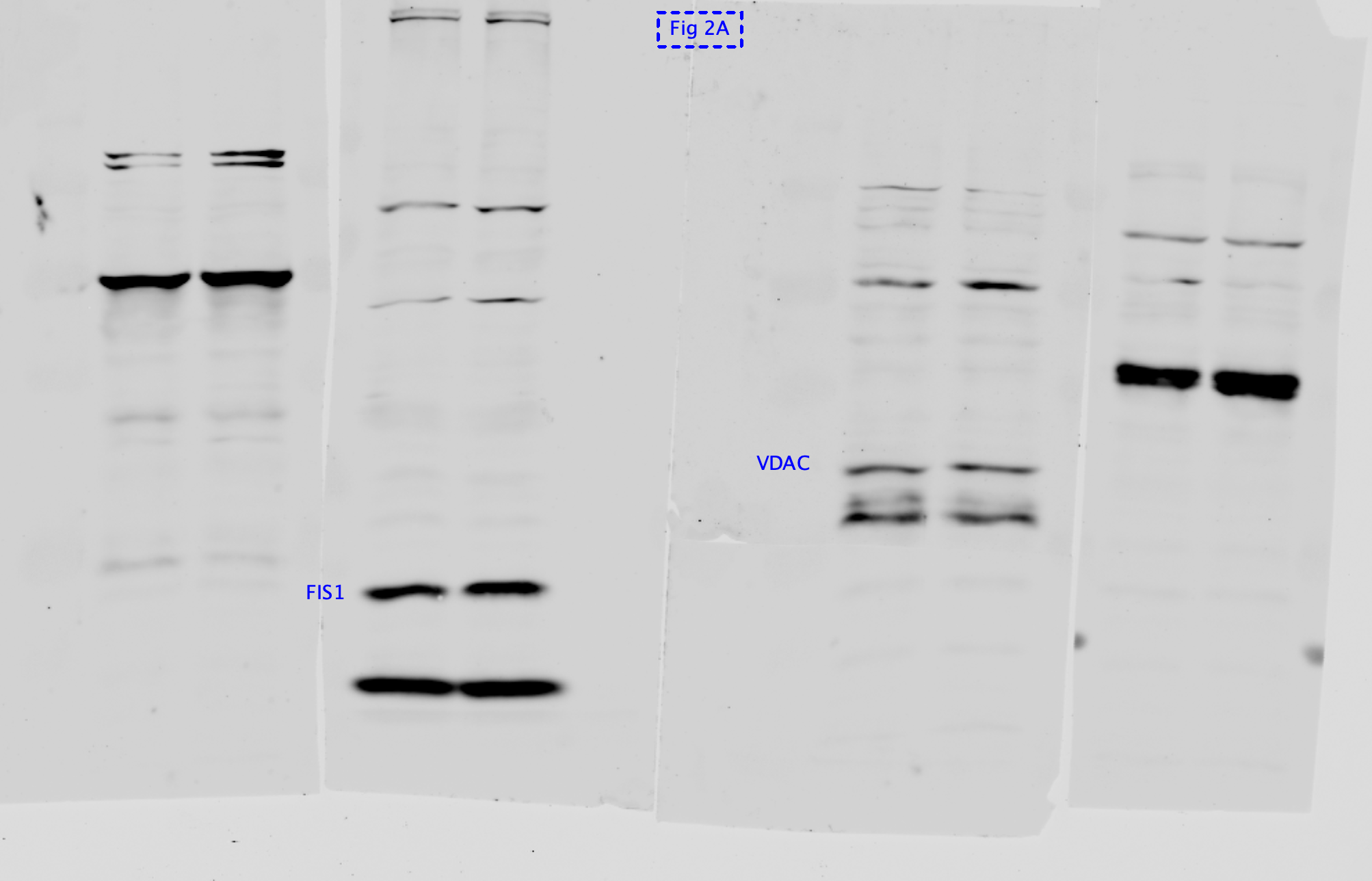

Supplement: Figure 2—source data 1. [file elife-82860-fig2-data1.zip › elife_Fig 2 source data/elife_Fig 2 source data 1/Fig_2A_Source Data_labeled/Fig_2A_Fis1_VDAC labeled.tif]

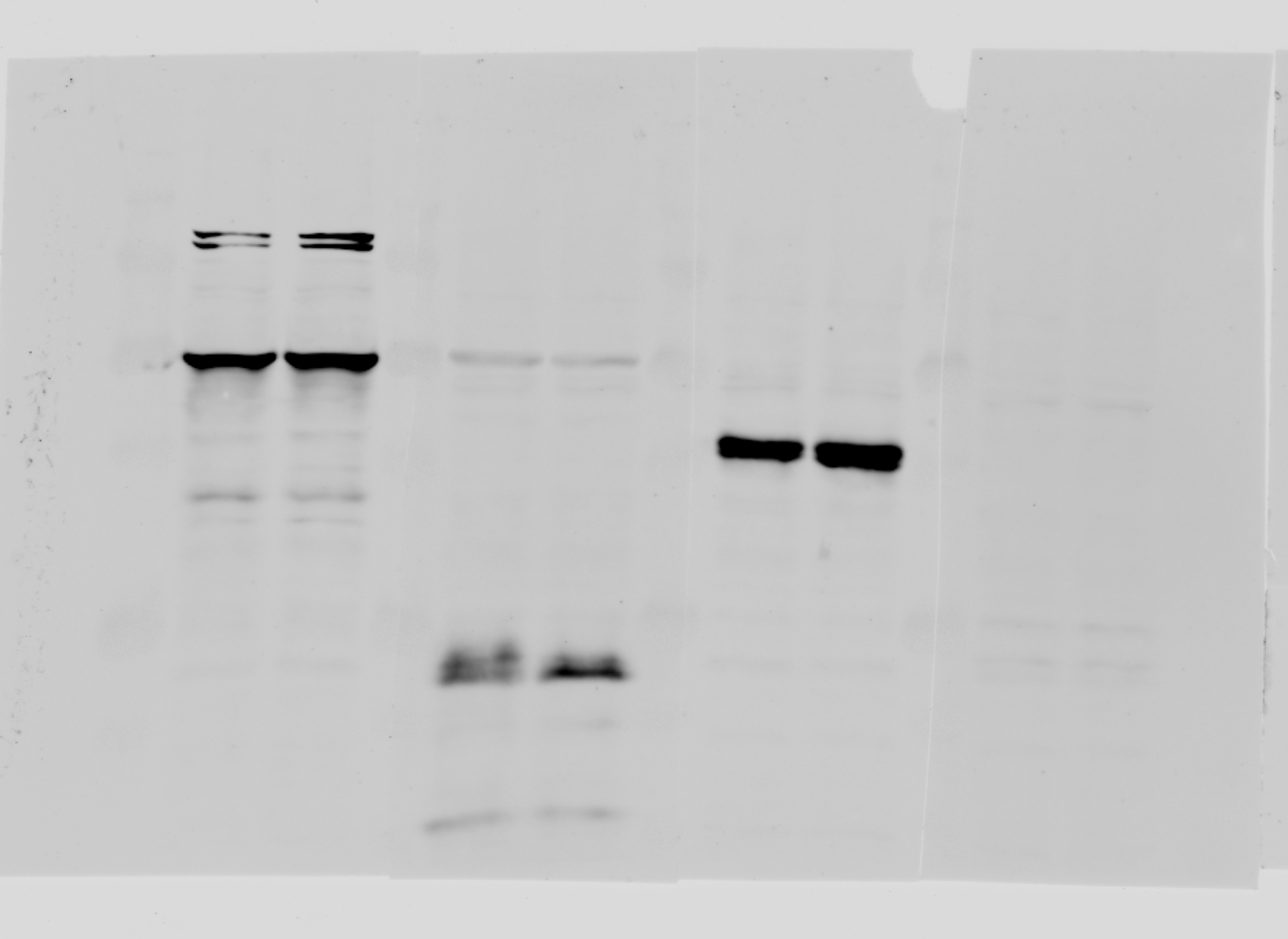

Supplement: Figure 2—source data 1. [file elife-82860-fig2-data1.zip › elife_Fig 2 source data/elife_Fig 2 source data 1/Fig_2A_Source Data_unlabeled/Fig_2A_ATAD1_BIM_MCL1_Tub unlabeled.tif]

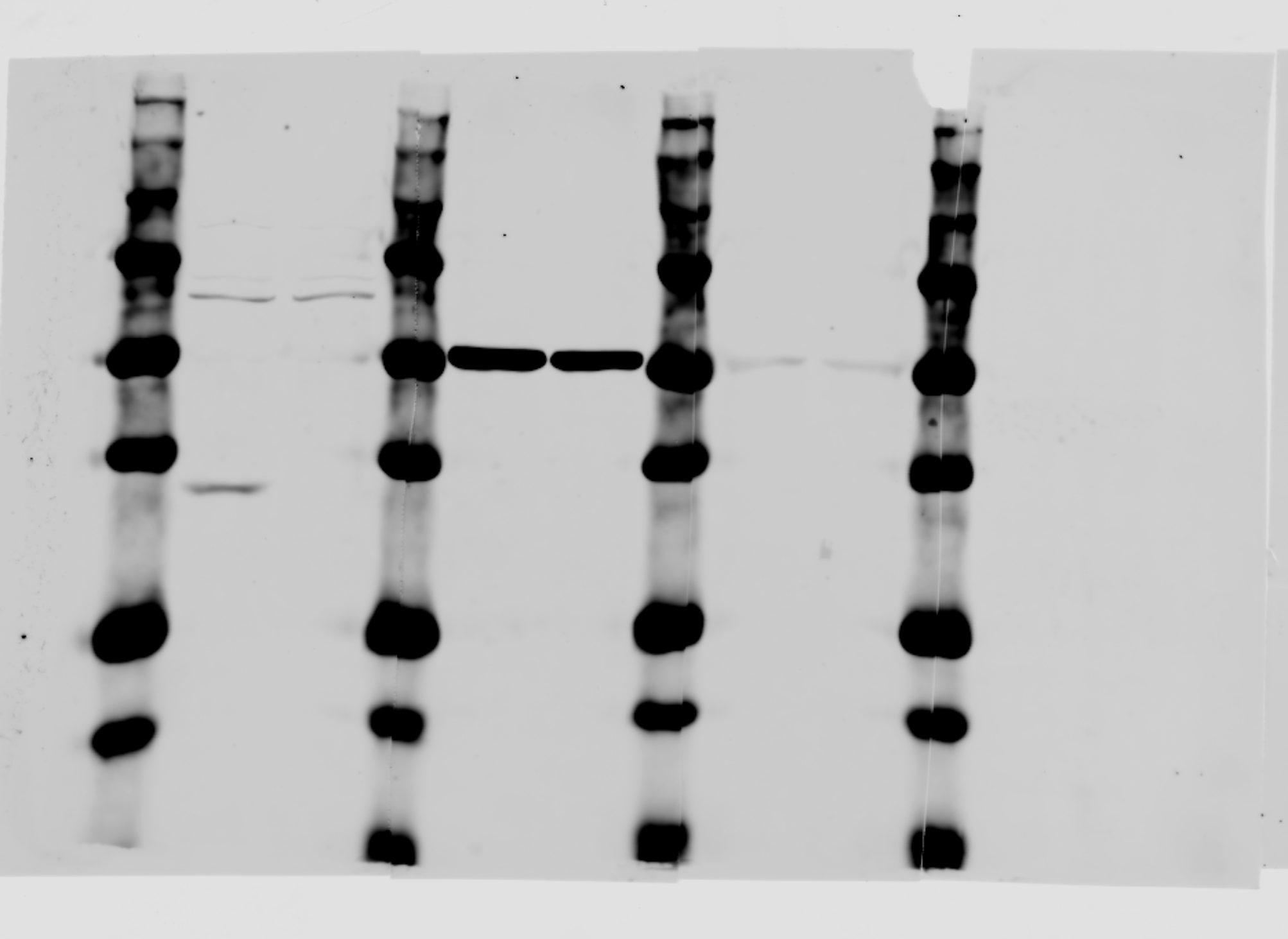

Supplement: Figure 2—source data 1. [file elife-82860-fig2-data1.zip › elife_Fig 2 source data/elife_Fig 2 source data 1/Fig_2A_Source Data_unlabeled/Fig_2A_ATAD1_Tub unlabeled.tif]

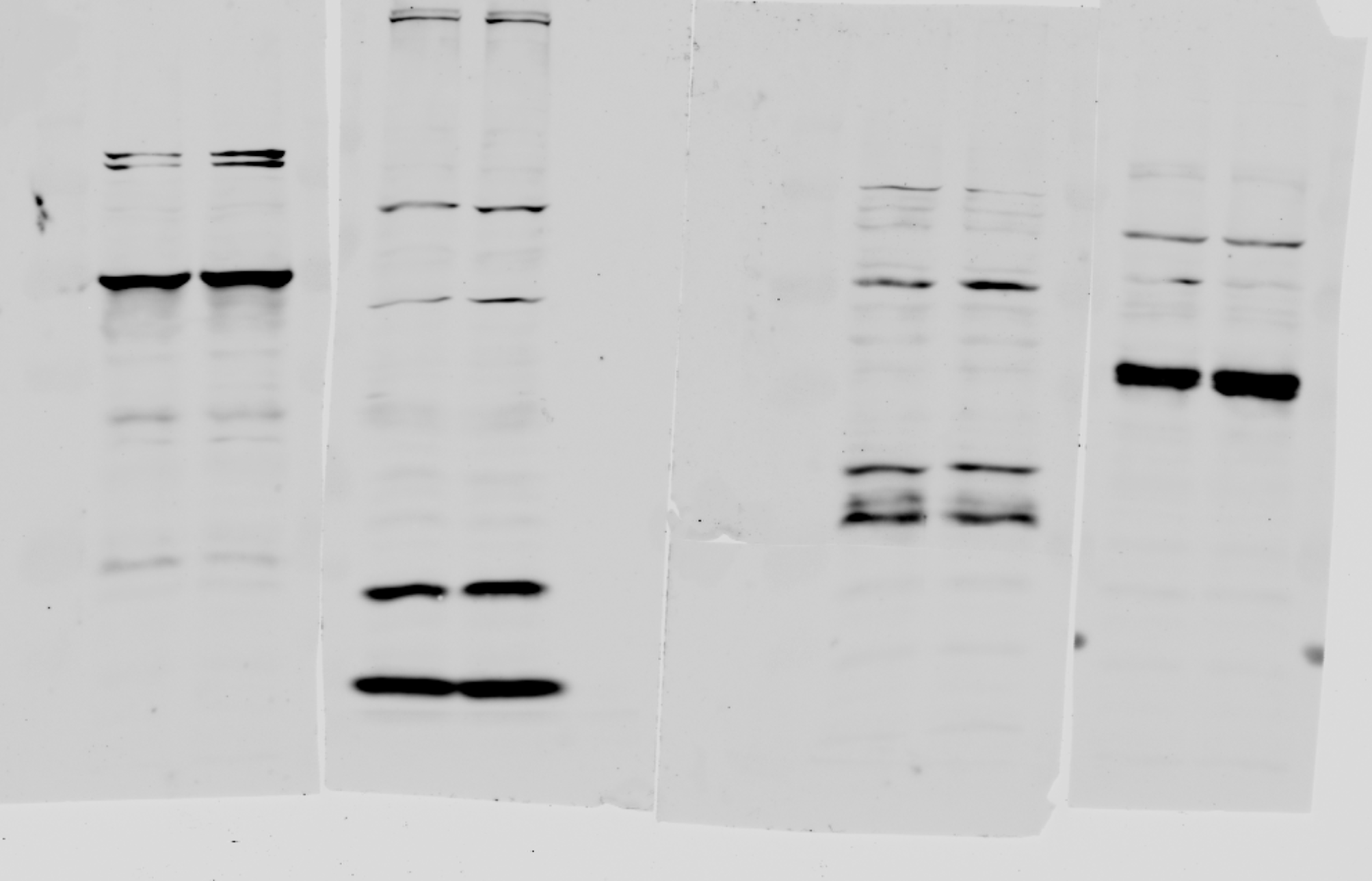

Supplement: Figure 2—source data 1. [file elife-82860-fig2-data1.zip › elife_Fig 2 source data/elife_Fig 2 source data 1/Fig_2A_Source Data_unlabeled/Fig_2A_Fis1_VDAC unlabeled.tif]

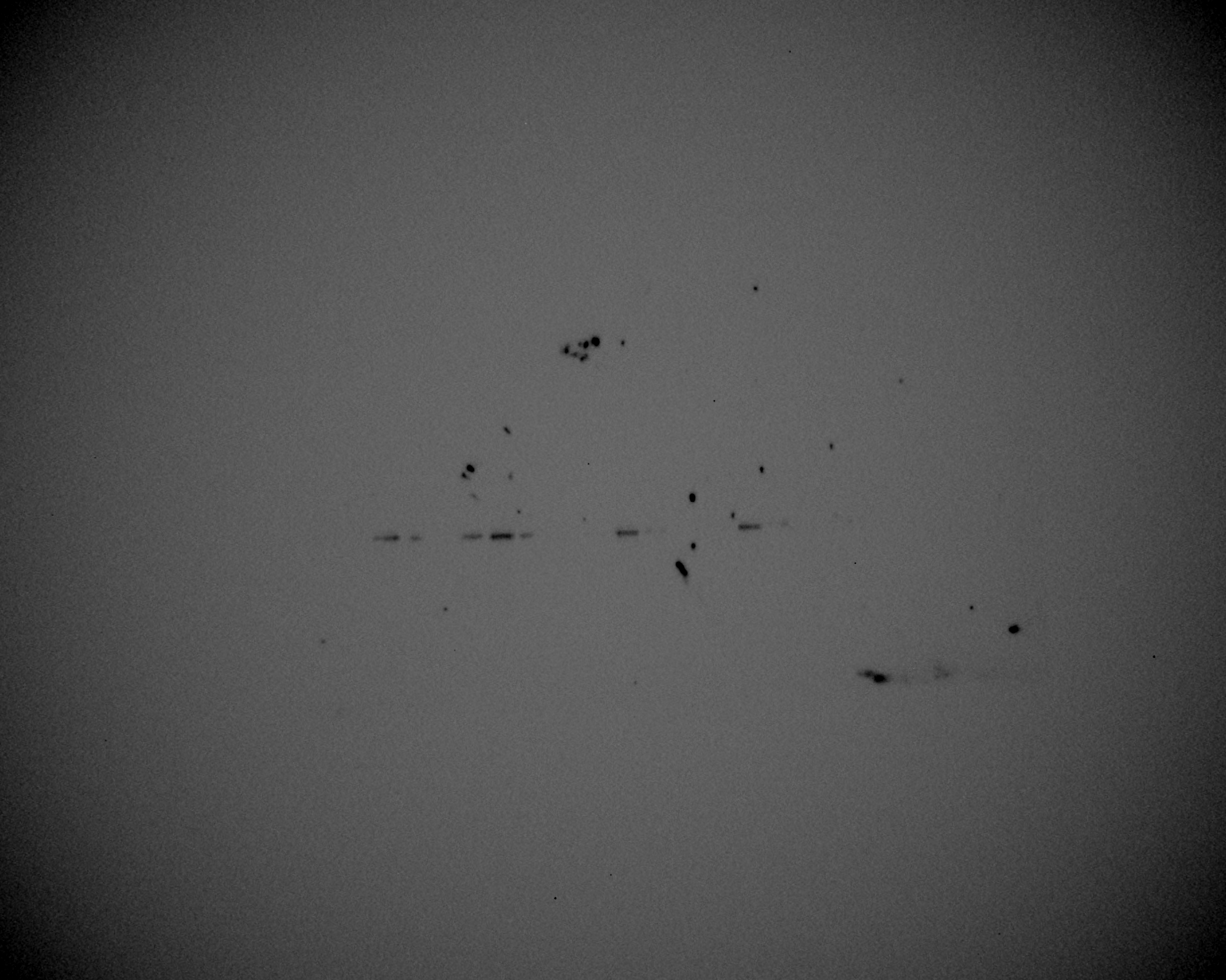

Supplement: Figure 2—source data 1. [file elife-82860-fig2-data1.zip › elife_Fig 2 source data/elife_Fig 2 source data 7/Fig_2J_Source_Data_Unlabeled/Fig_2J_Anti-Flag_Unlabeled.jpg]

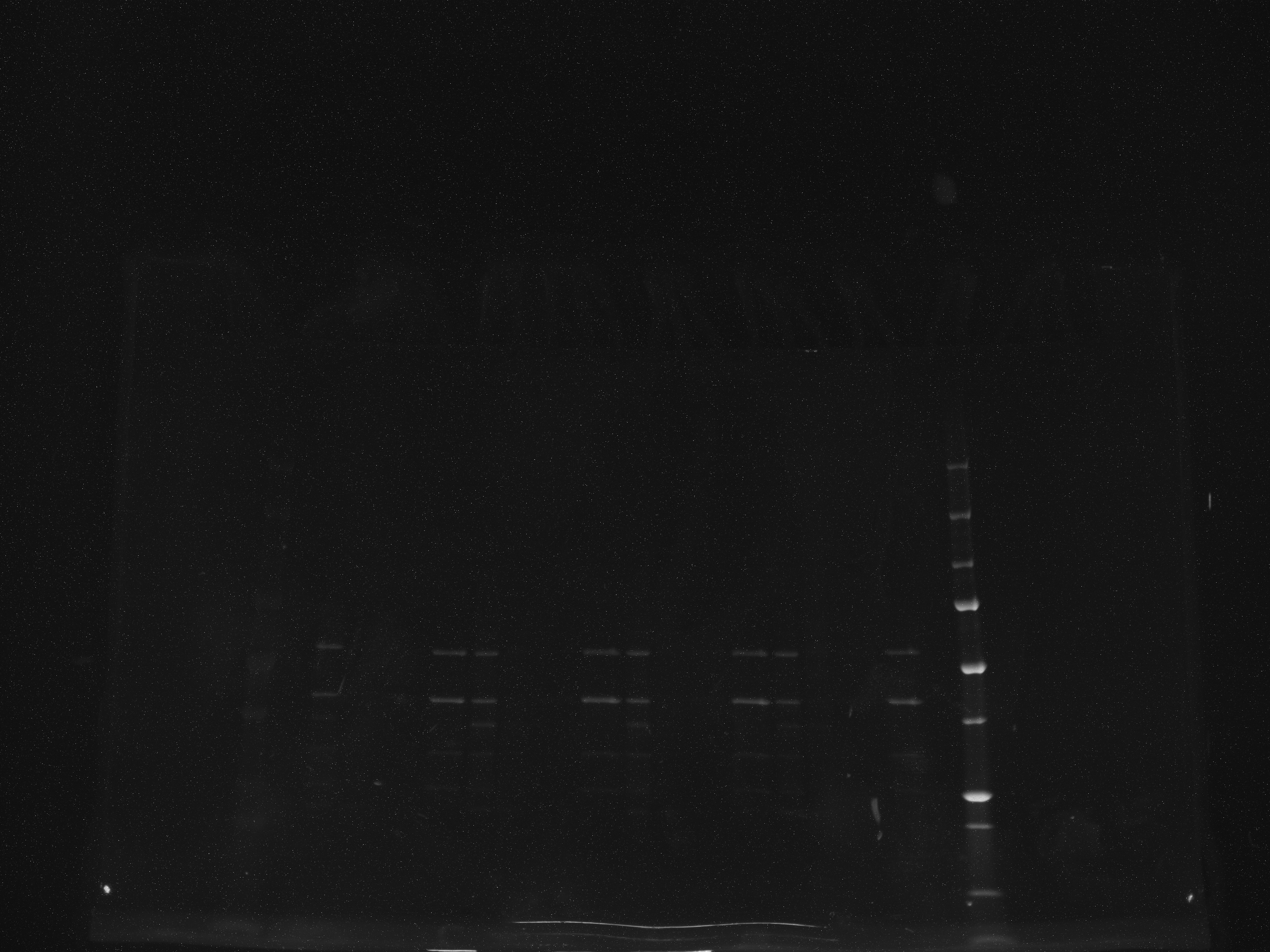

Supplement: Figure 2—source data 1. [file elife-82860-fig2-data1.zip › elife_Fig 2 source data/elife_Fig 2 source data 7/Fig_2J_Source_Data_Unlabeled/Fig_2J_StainFree_Unlabeled.jpg]

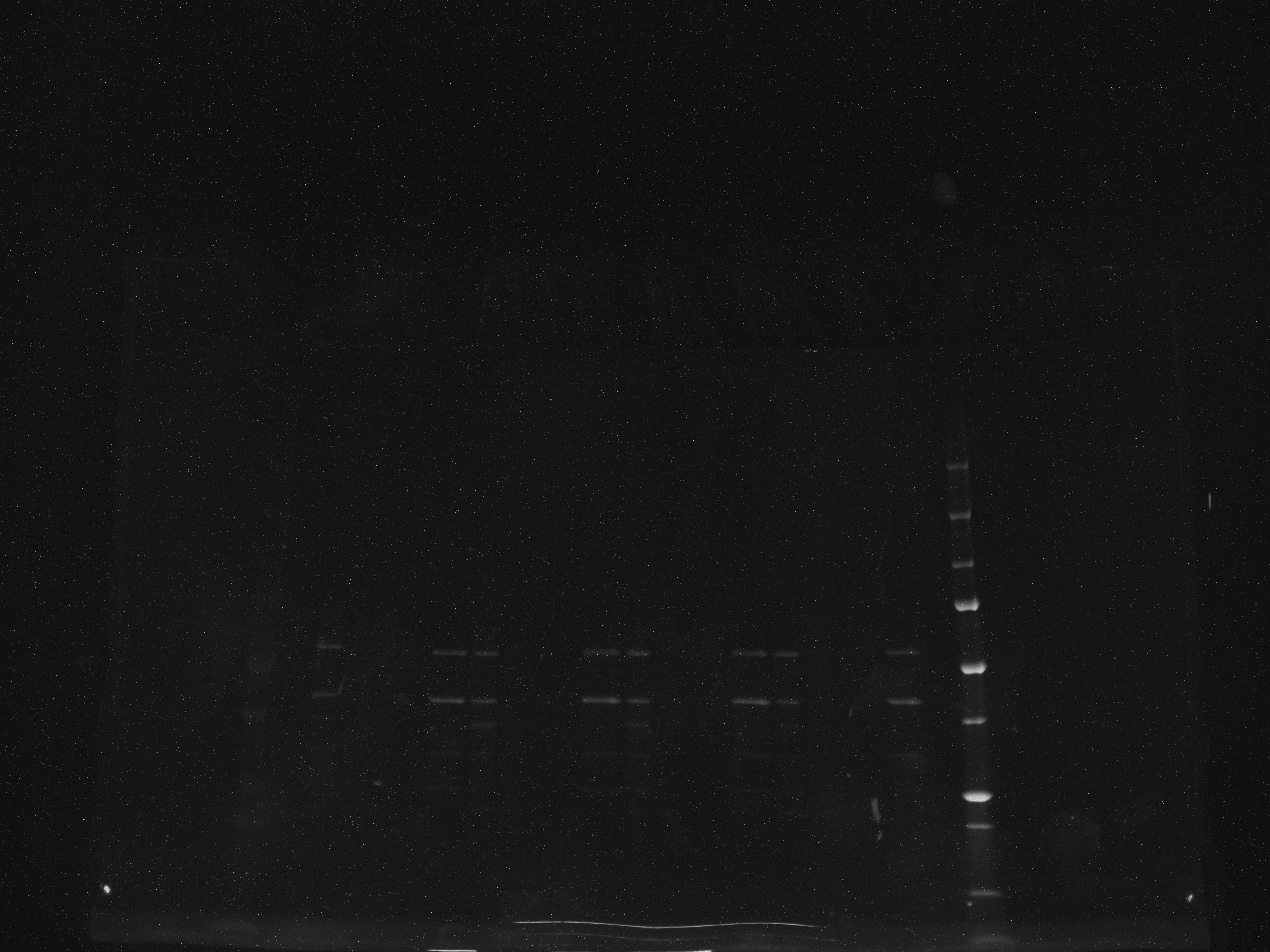

Supplement: Figure 2—source data 1. [file elife-82860-fig2-data1.zip › elife_Fig 2 source data/elife_Fig 2 source data 7/Fig_2J_Source_Data_Labeled/Fig_2J_StainFree_Labeled.jpeg]

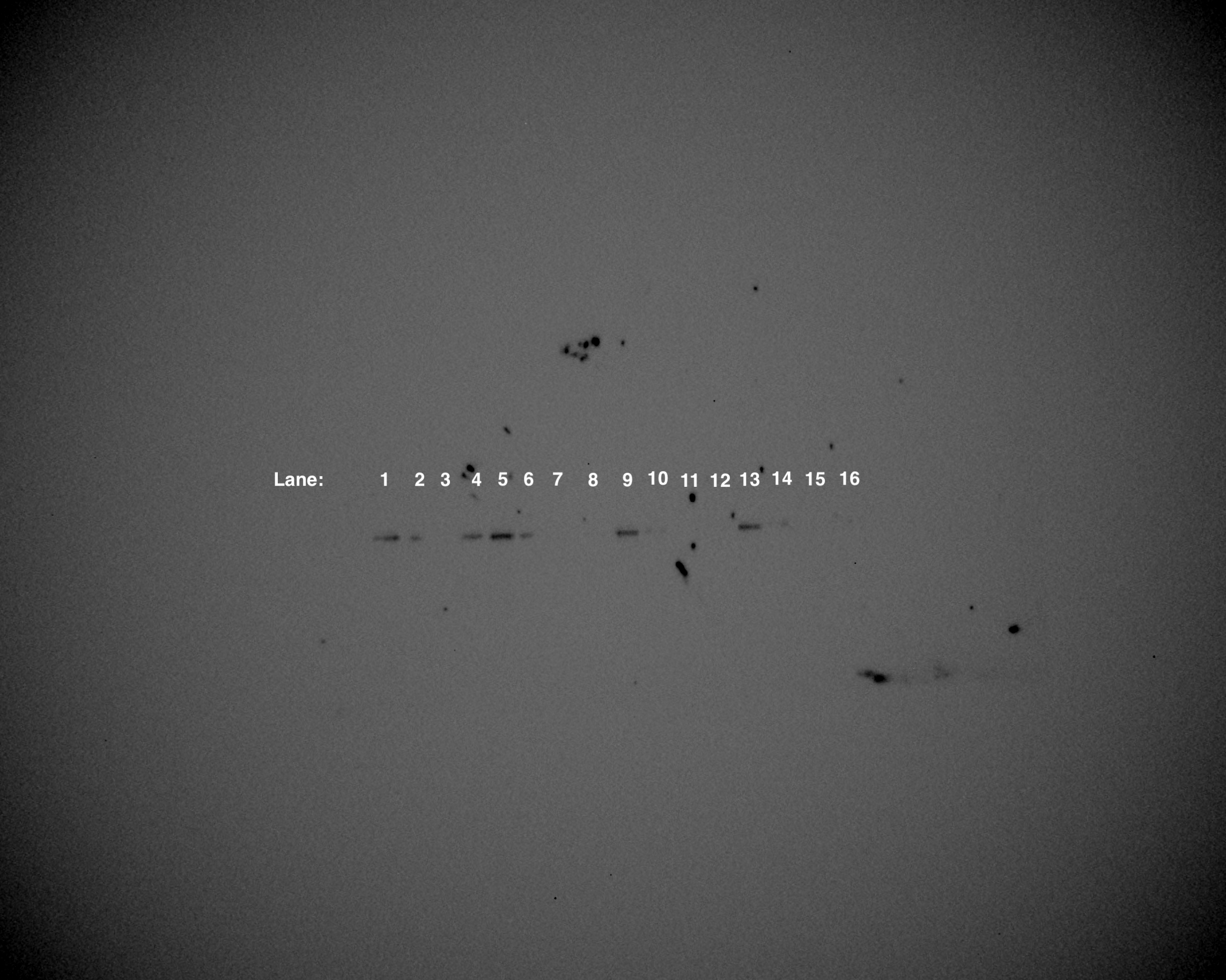

Supplement: Figure 2—source data 1. [file elife-82860-fig2-data1.zip › elife_Fig 2 source data/elife_Fig 2 source data 7/Fig_2J_Source_Data_Labeled/Fig_2J_Anti-Flag_Labeled.jpeg]

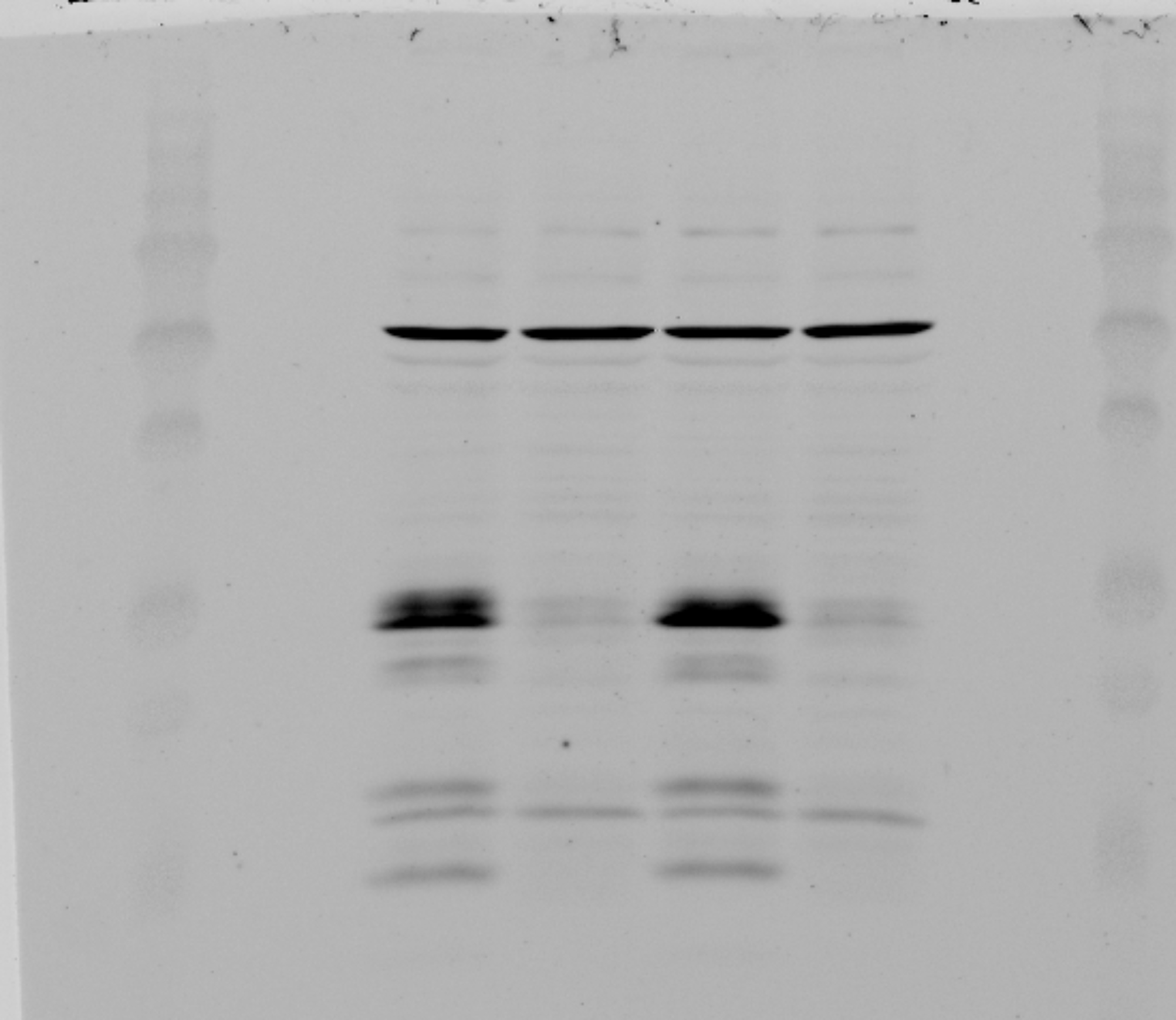

Supplement: Figure 2—figure supplement 1—source data 1. [file elife-82860-fig2-figsupp1-data1.zip › elife_Fig 2 Supp 1 source data/elife_Fig 2 Supp 1 source data 1/Fig_2_Supp_1A_Source_Data_Unlabeled/Fig_2_Supp_1A_BIM_Unlabeled.tif]

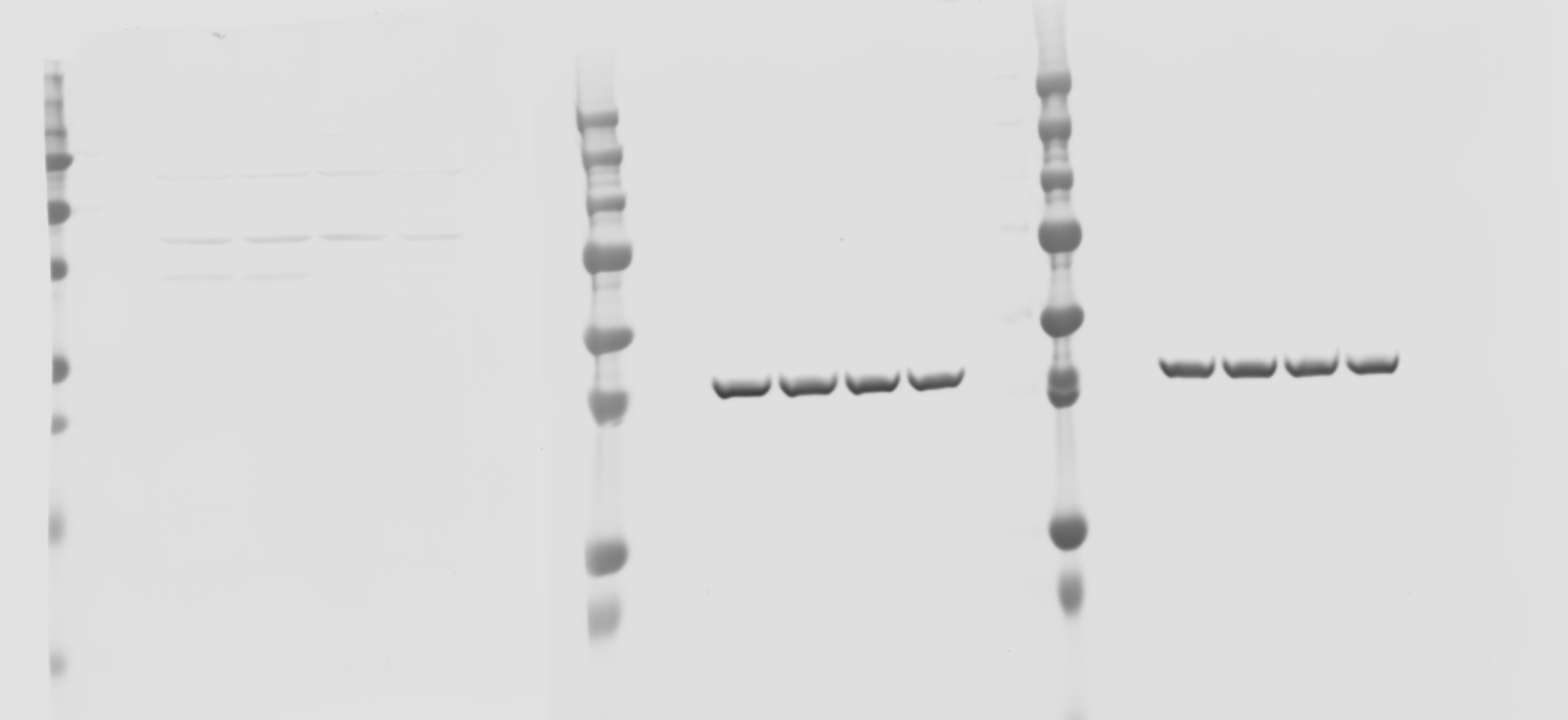

Supplement: Figure 2—figure supplement 1—source data 1. [file elife-82860-fig2-figsupp1-data1.zip › elife_Fig 2 Supp 1 source data/elife_Fig 2 Supp 1 source data 1/Fig_2_Supp_1A_Source_Data_Unlabeled/Fig_2_Supp_1A_Actin_PTAG_Unlabeled.tif]

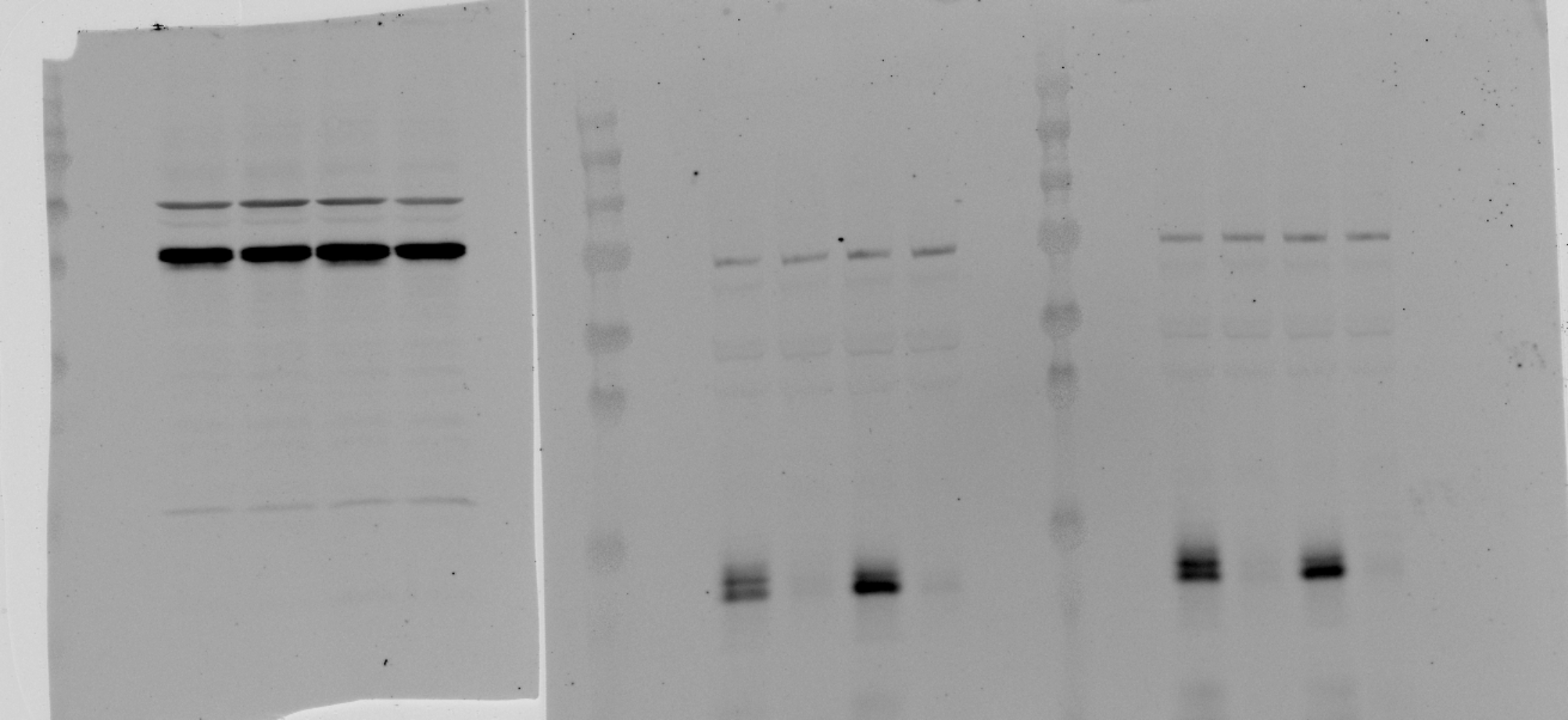

Supplement: Figure 2—figure supplement 1—source data 1. [file elife-82860-fig2-figsupp1-data1.zip › elife_Fig 2 Supp 1 source data/elife_Fig 2 Supp 1 source data 1/Fig_2_Supp_1A_Source_Data_Unlabeled/Fig_2_Supp_1_BIM_Phostag_Unlabeled.tif]

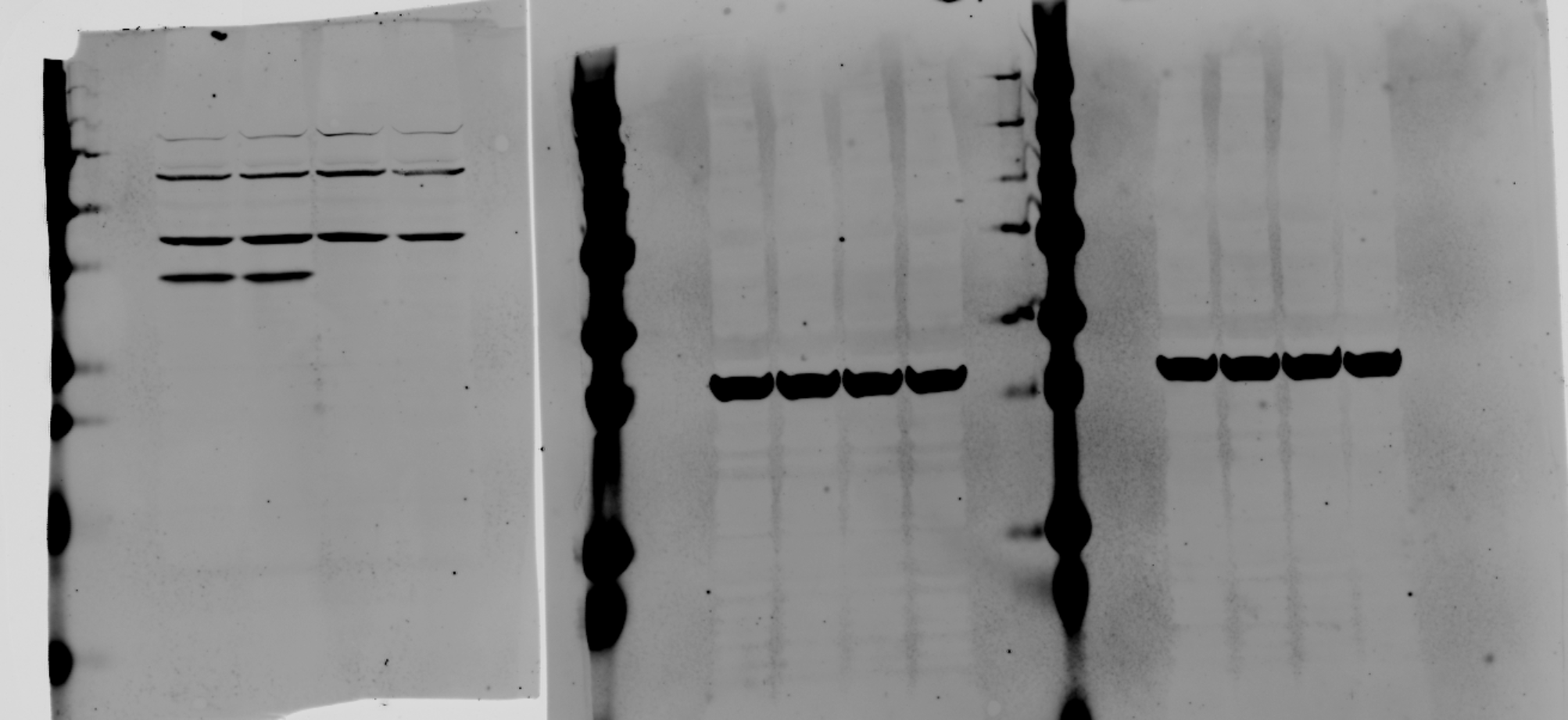

Supplement: Figure 2—figure supplement 1—source data 1. [file elife-82860-fig2-figsupp1-data1.zip › elife_Fig 2 Supp 1 source data/elife_Fig 2 Supp 1 source data 1/Fig_2_Supp_1A_Source_Data_Unlabeled/Fig_2_Supp_1A_ATAD1_Actin_Unlabeled.tiff]

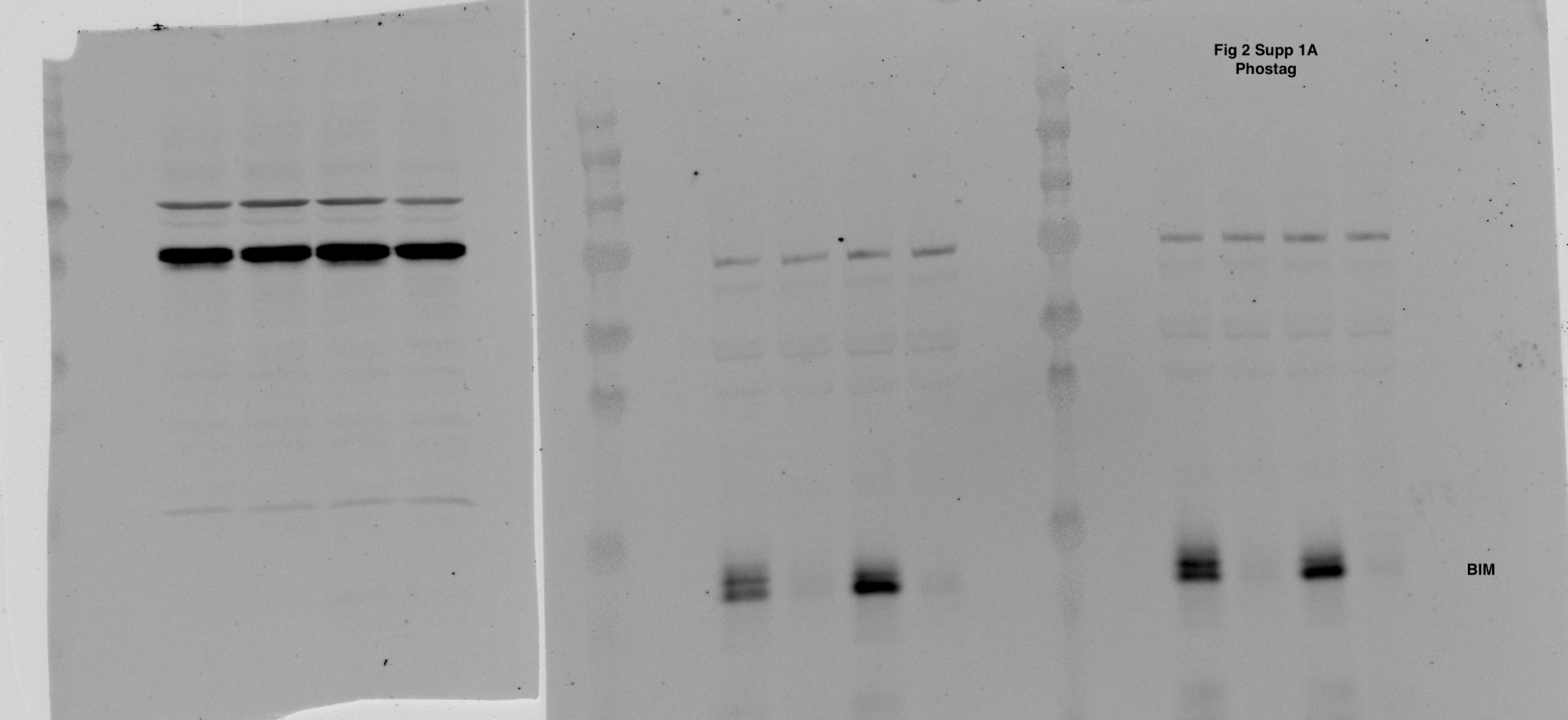

Supplement: Figure 2—figure supplement 1—source data 1. [file elife-82860-fig2-figsupp1-data1.zip › elife_Fig 2 Supp 1 source data/elife_Fig 2 Supp 1 source data 1/Fig_2_Supp_1A_Source_Data_labeled/Fig_2_Supp_1_BIM_Phostag_labeled.tif]

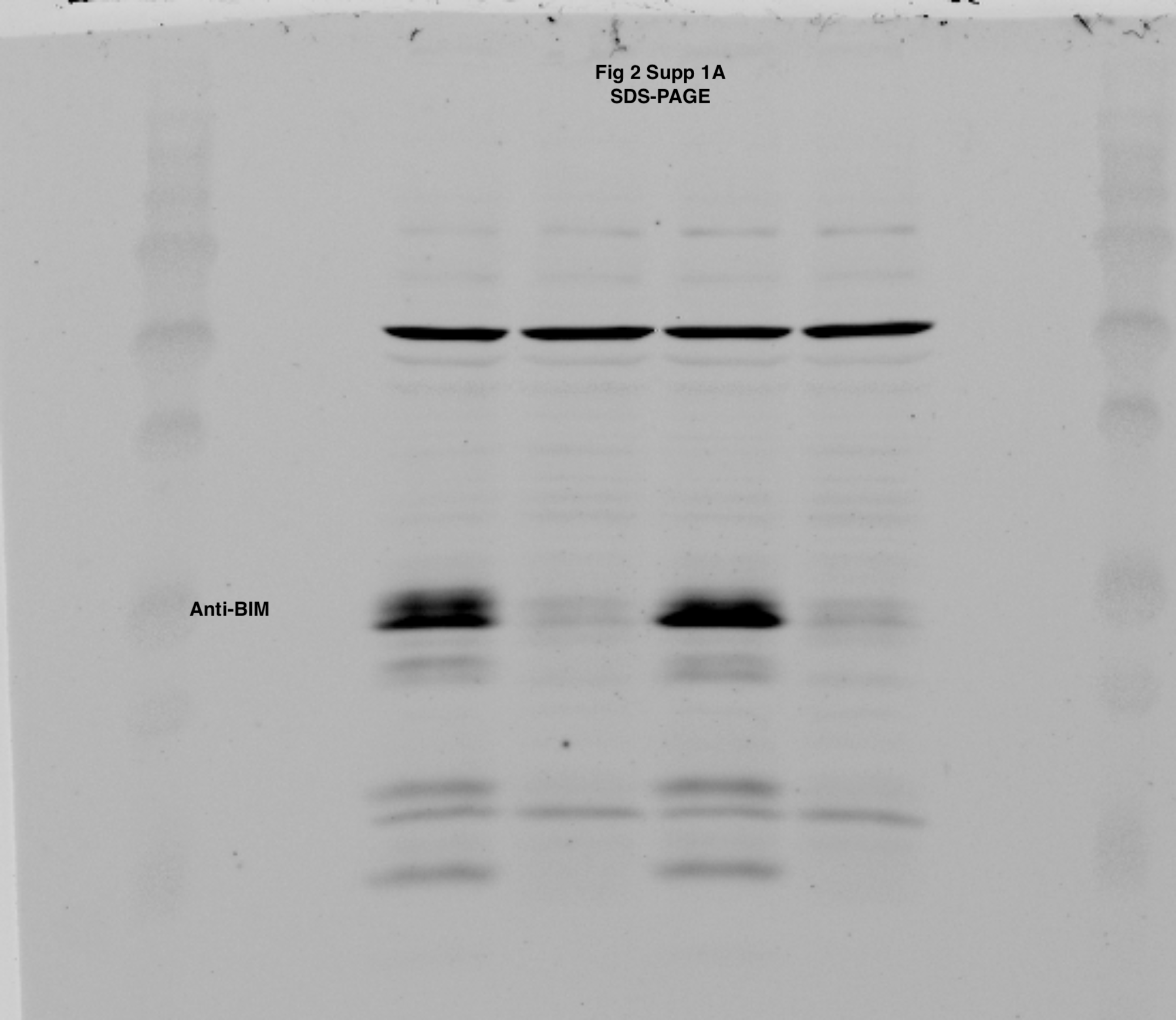

Supplement: Figure 2—figure supplement 1—source data 1. [file elife-82860-fig2-figsupp1-data1.zip › elife_Fig 2 Supp 1 source data/elife_Fig 2 Supp 1 source data 1/Fig_2_Supp_1A_Source_Data_labeled/Fig_2_Supp_1A_BIM_labeled.tif]

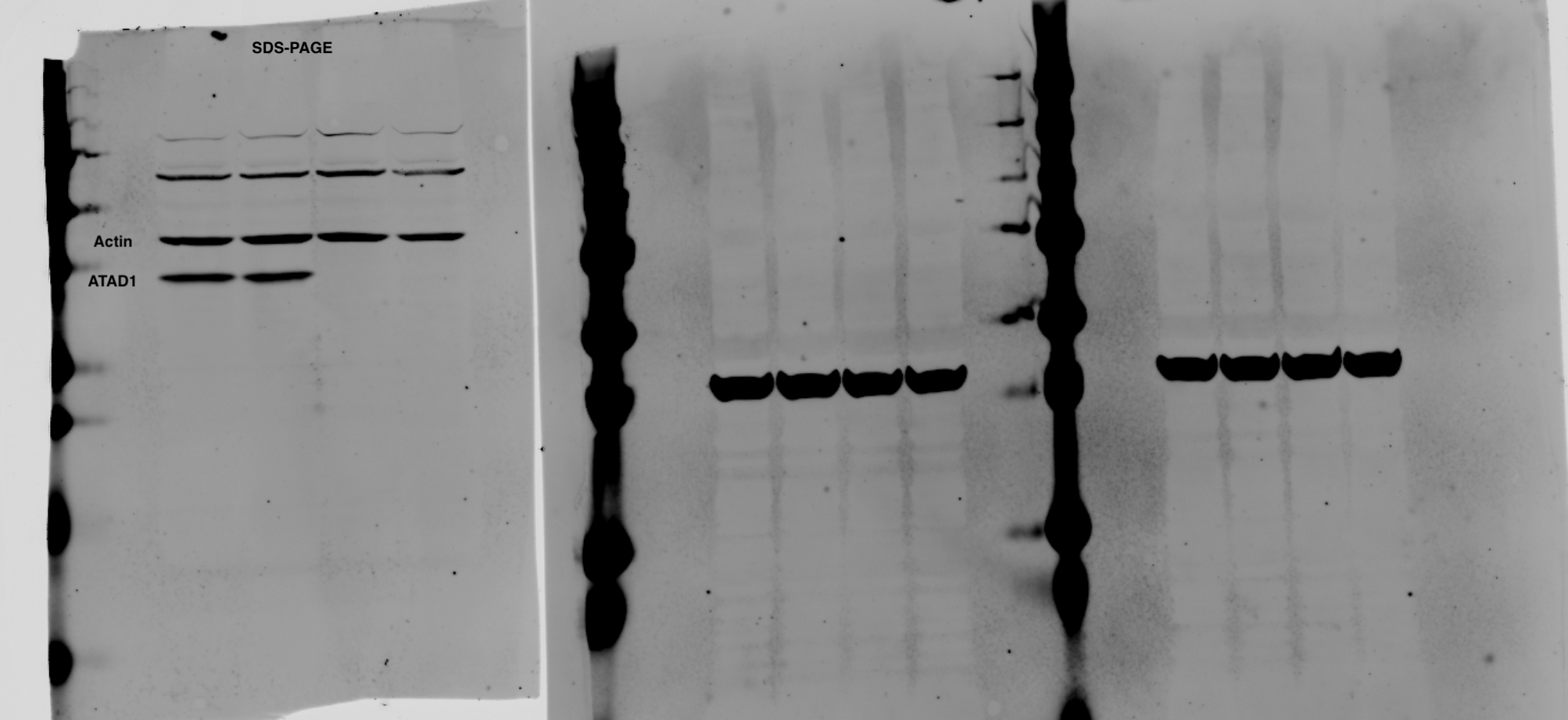

Supplement: Figure 2—figure supplement 1—source data 1. [file elife-82860-fig2-figsupp1-data1.zip › elife_Fig 2 Supp 1 source data/elife_Fig 2 Supp 1 source data 1/Fig_2_Supp_1A_Source_Data_labeled/Fig_2_Supp_1_ATAD1_Actin_Labeled.tif]

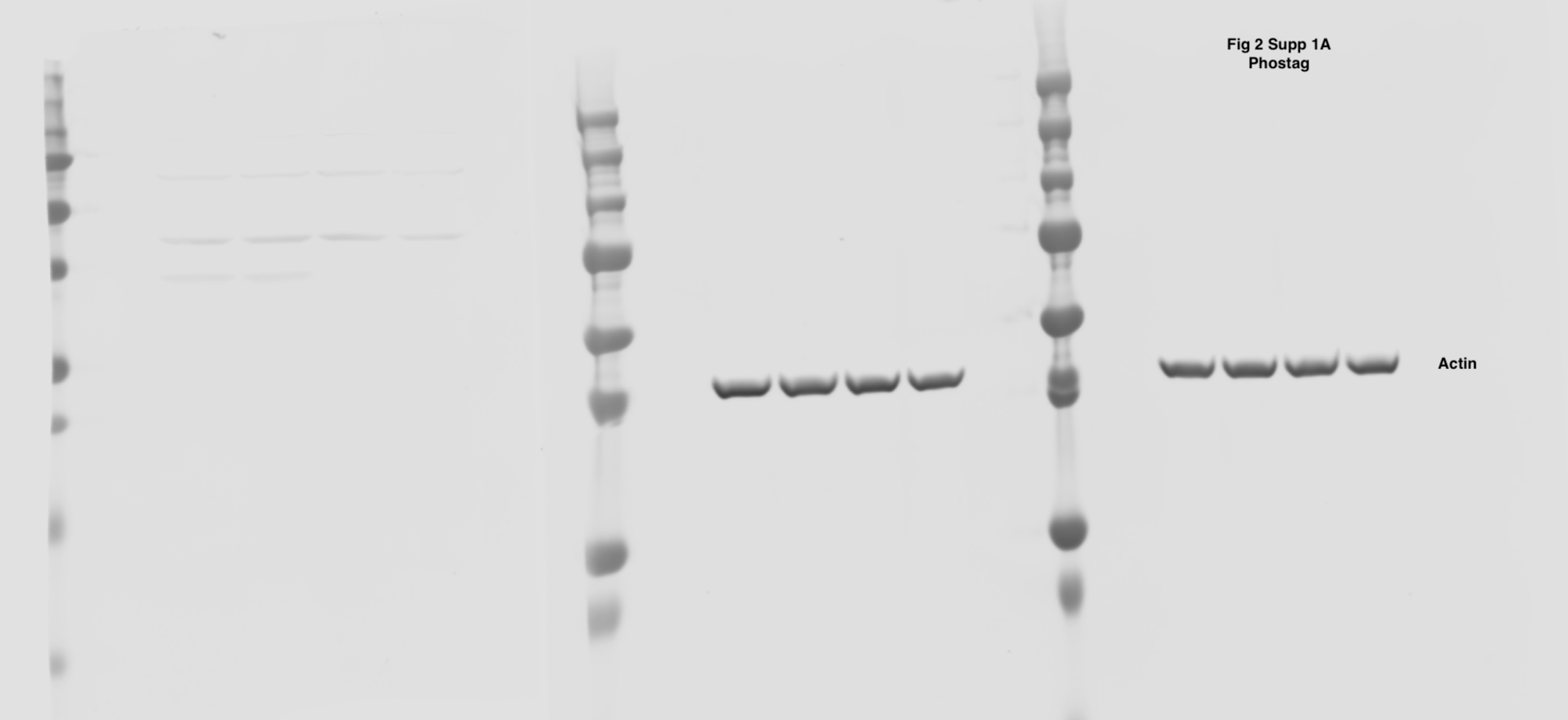

Supplement: Figure 2—figure supplement 1—source data 1. [file elife-82860-fig2-figsupp1-data1.zip › elife_Fig 2 Supp 1 source data/elife_Fig 2 Supp 1 source data 1/Fig_2_Supp_1A_Source_Data_labeled/Fig_2_Supp_1A_Actin_Ptag_Labeled.tif]

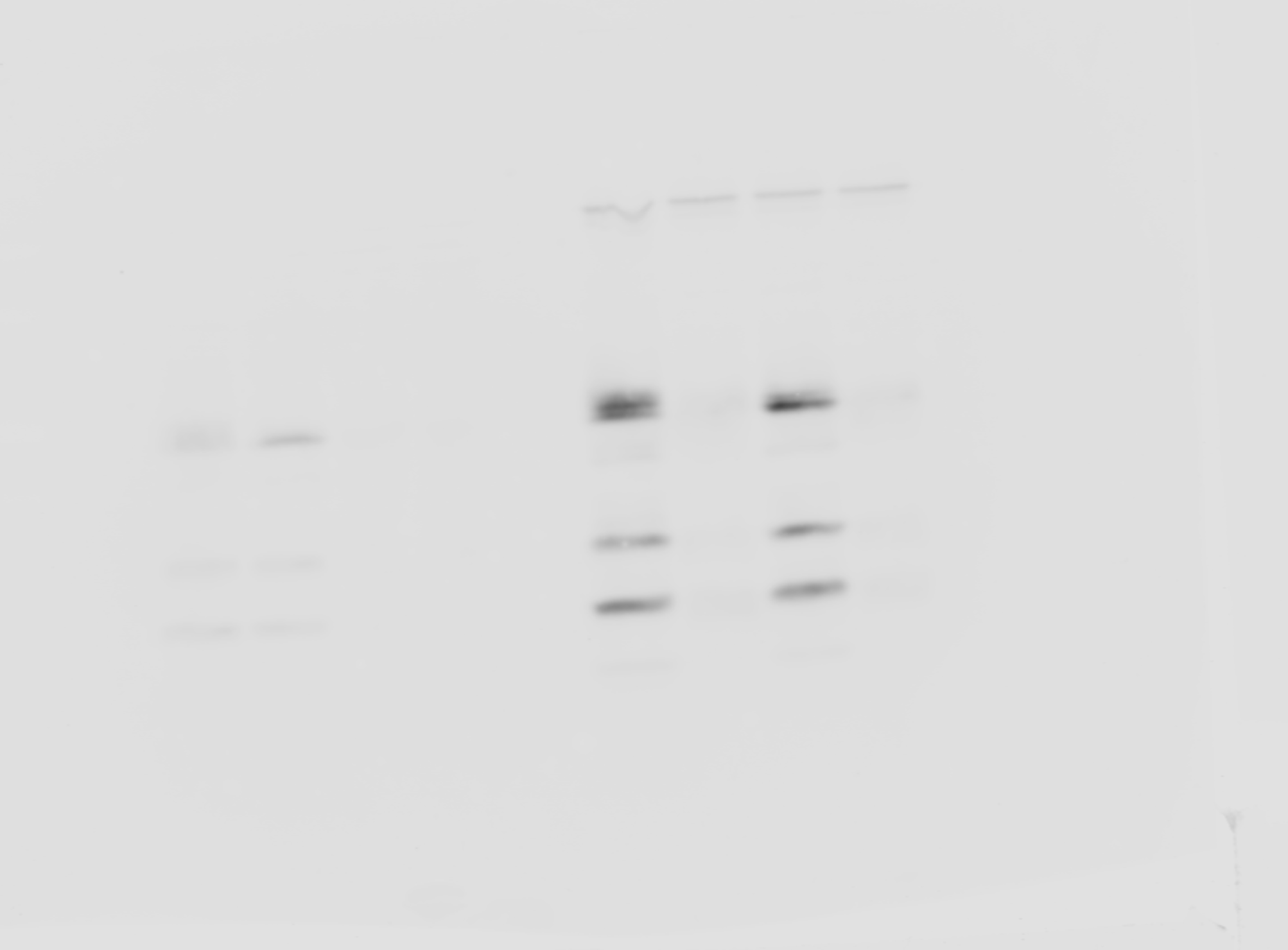

Supplement: Figure 2—figure supplement 1—source data 1. [file elife-82860-fig2-figsupp1-data1.zip › elife_Fig 2 Supp 1 source data/elife_Fig 2 Supp 1 source data 2/Fig_2_Supp_2B_Source_Data_Unlabeled/Fig_2_Supp_2_BIM_Unlabeled.tif]

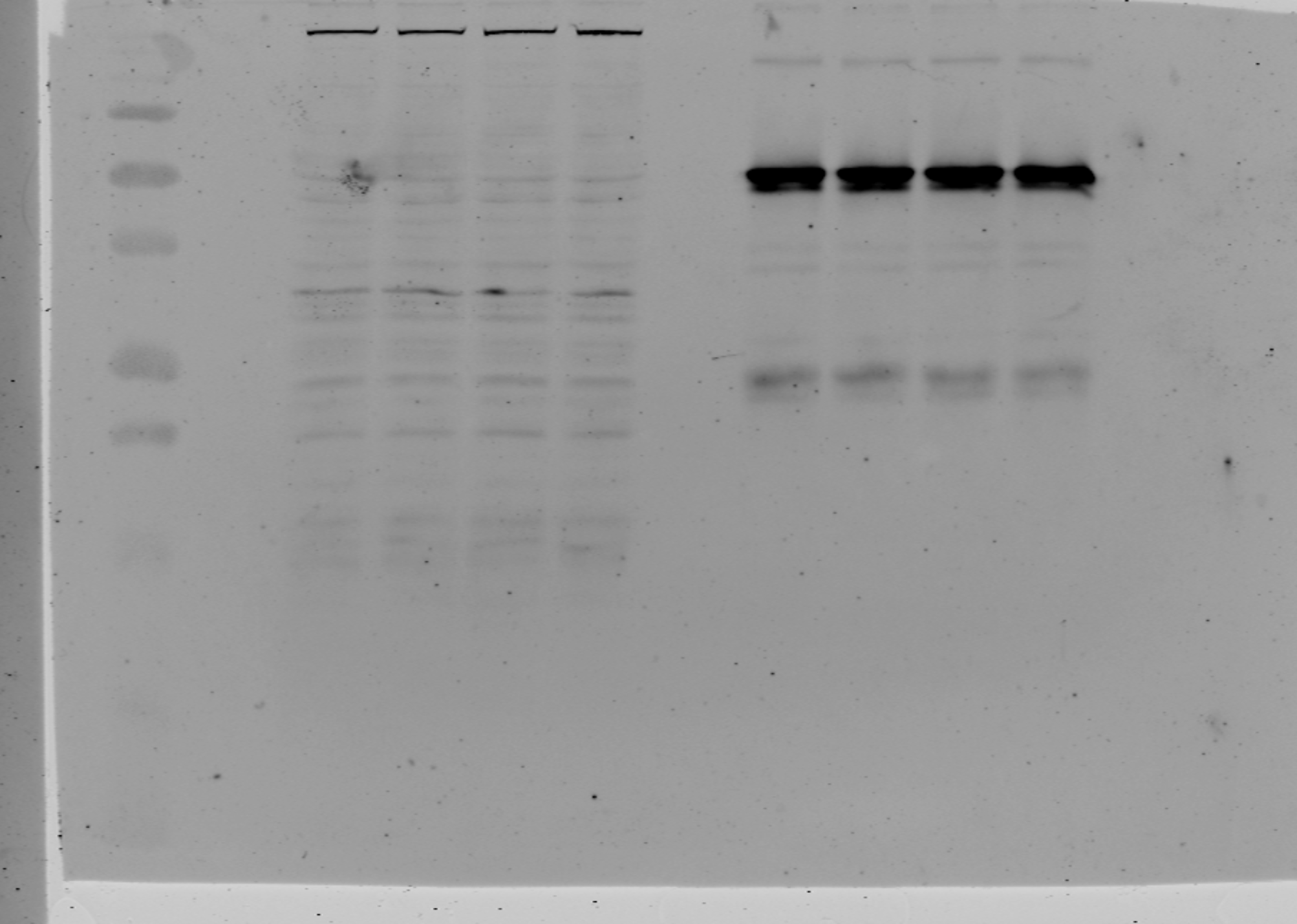

Supplement: Figure 2—figure supplement 1—source data 1. [file elife-82860-fig2-figsupp1-data1.zip › elife_Fig 2 Supp 1 source data/elife_Fig 2 Supp 1 source data 2/Fig_2_Supp_2B_Source_Data_Unlabeled/Fig_2_Supp_2B_BIM_Thr112_Unlabeled.tif]

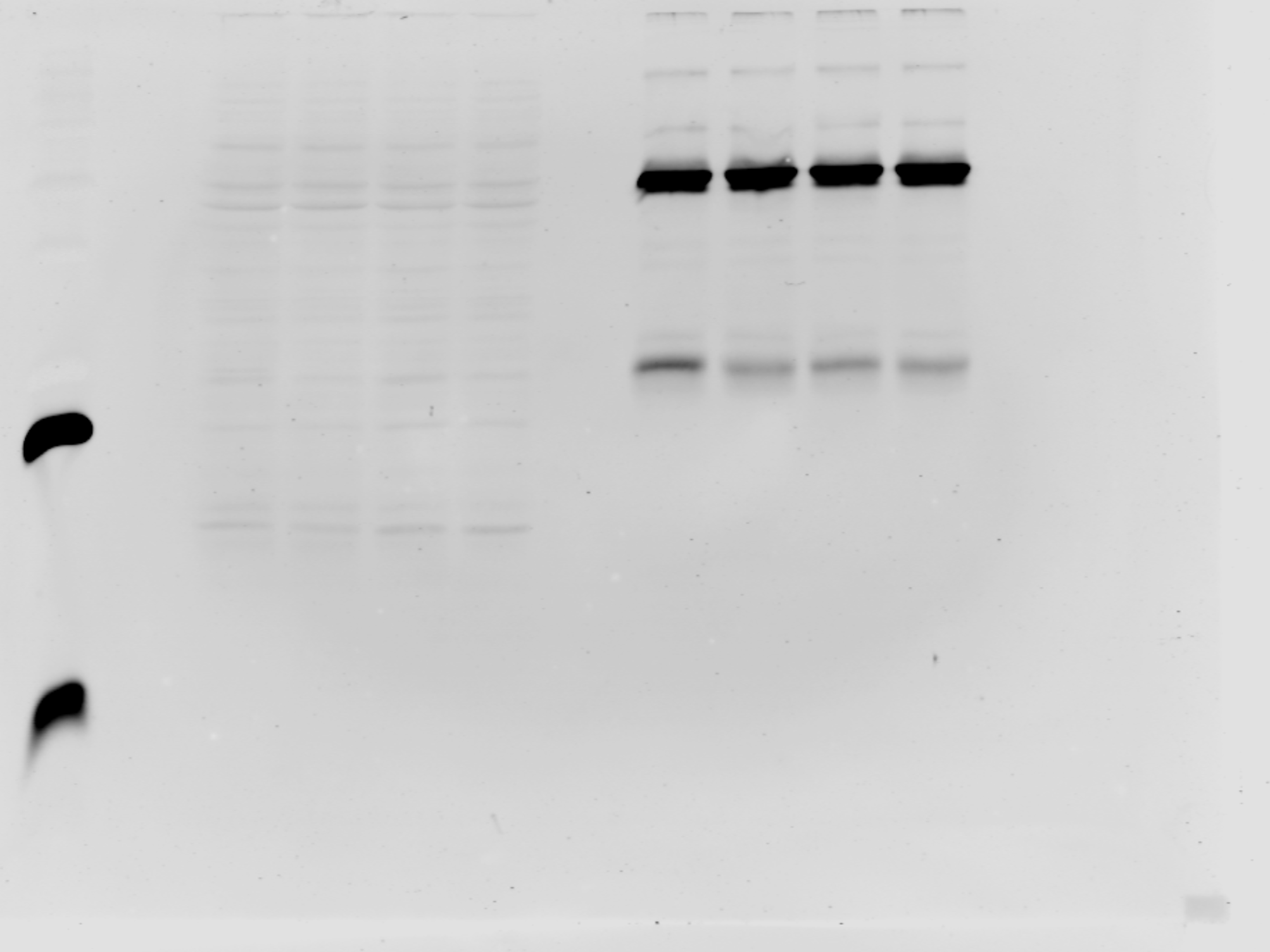

Supplement: Figure 2—figure supplement 1—source data 1. [file elife-82860-fig2-figsupp1-data1.zip › elife_Fig 2 Supp 1 source data/elife_Fig 2 Supp 1 source data 2/Fig_2_Supp_2B_Source_Data_Unlabeled/Fig_2_Supp_2B_BIM_Ser69_Unlabeled.tif]

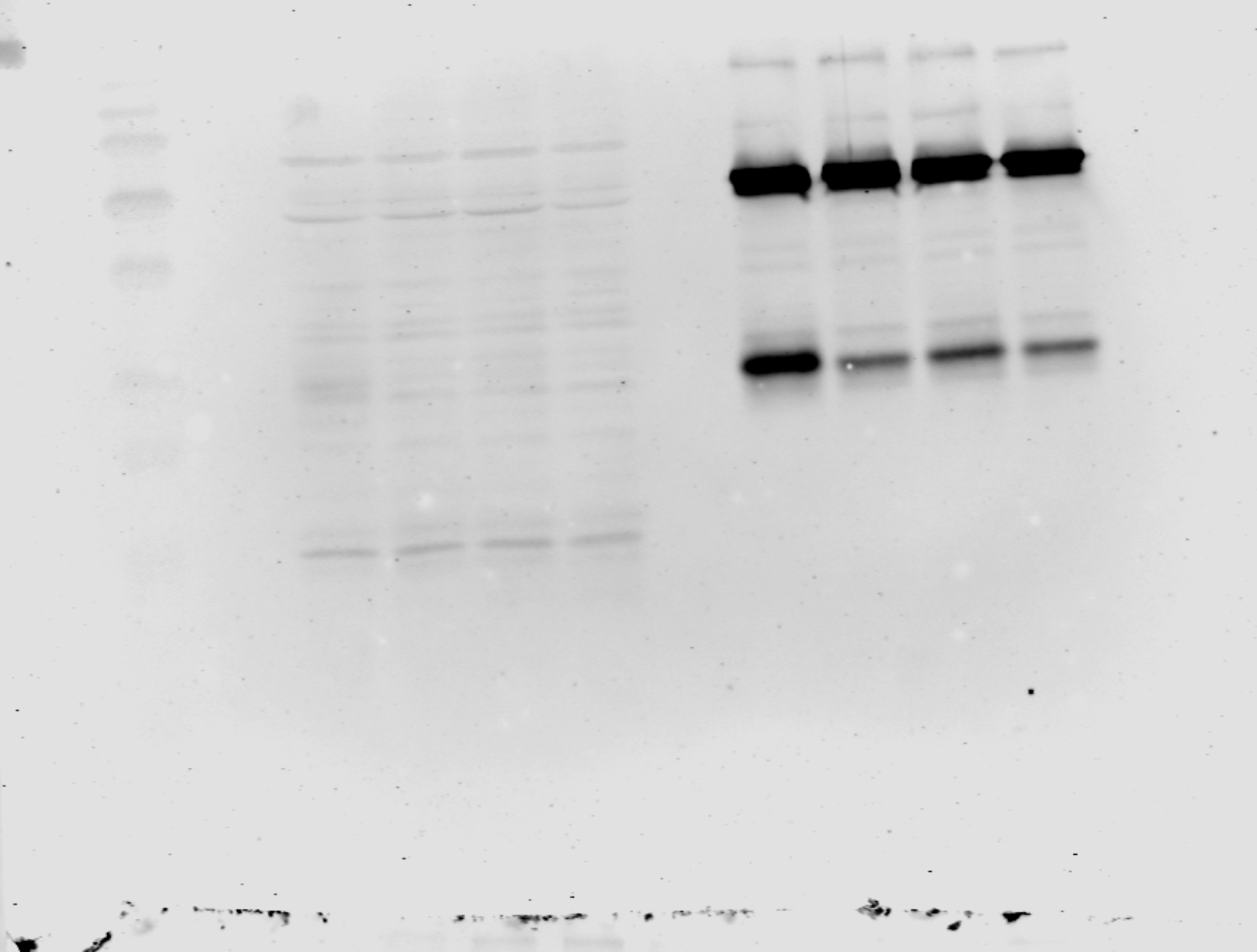

Supplement: Figure 2—figure supplement 1—source data 1. [file elife-82860-fig2-figsupp1-data1.zip › elife_Fig 2 Supp 1 source data/elife_Fig 2 Supp 1 source data 2/Fig_2_Supp_2B_Source_Data_Unlabeled/Fig_2_Supp_2B_BIM_Ser77_Unlabeled.tif]

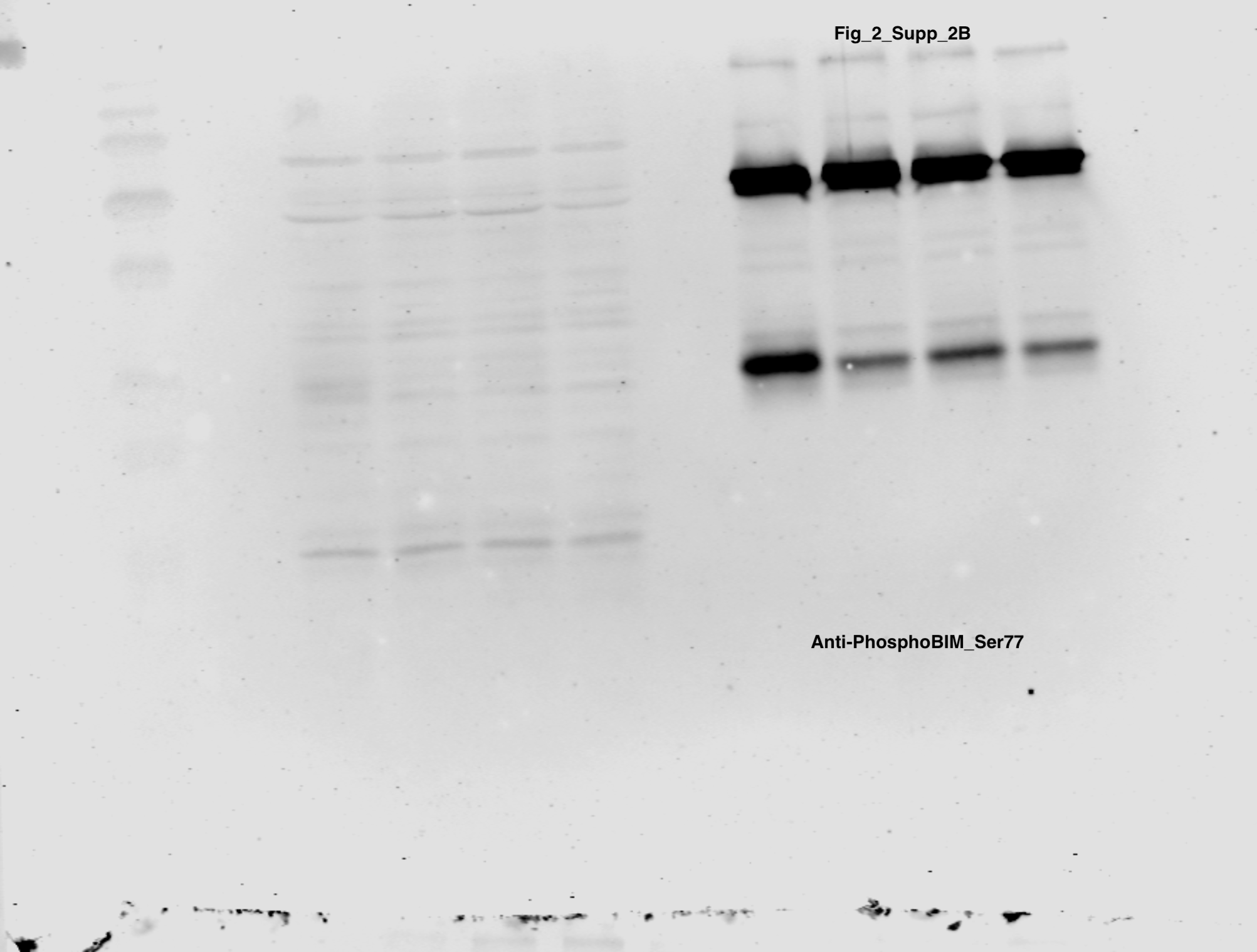

Supplement: Figure 2—figure supplement 1—source data 1. [file elife-82860-fig2-figsupp1-data1.zip › elife_Fig 2 Supp 1 source data/elife_Fig 2 Supp 1 source data 2/Fig_2_Supp_2B_Source_Data_Labeled/Fig_2_Supp_2B_BIM_Ser77_labeled.tif]

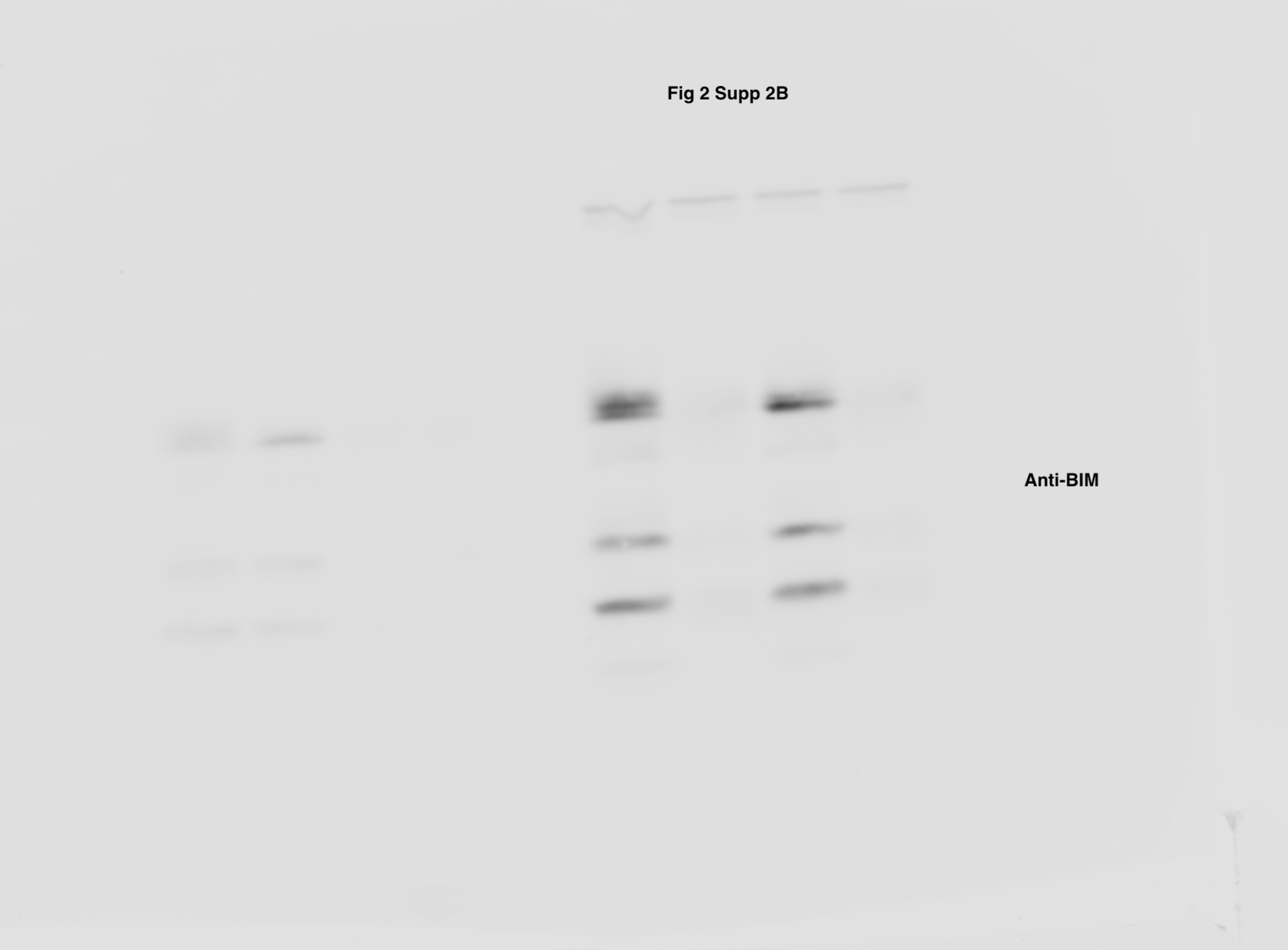

Supplement: Figure 2—figure supplement 1—source data 1. [file elife-82860-fig2-figsupp1-data1.zip › elife_Fig 2 Supp 1 source data/elife_Fig 2 Supp 1 source data 2/Fig_2_Supp_2B_Source_Data_Labeled/Fig_2_Supp_2_BIM_labeled.tif]

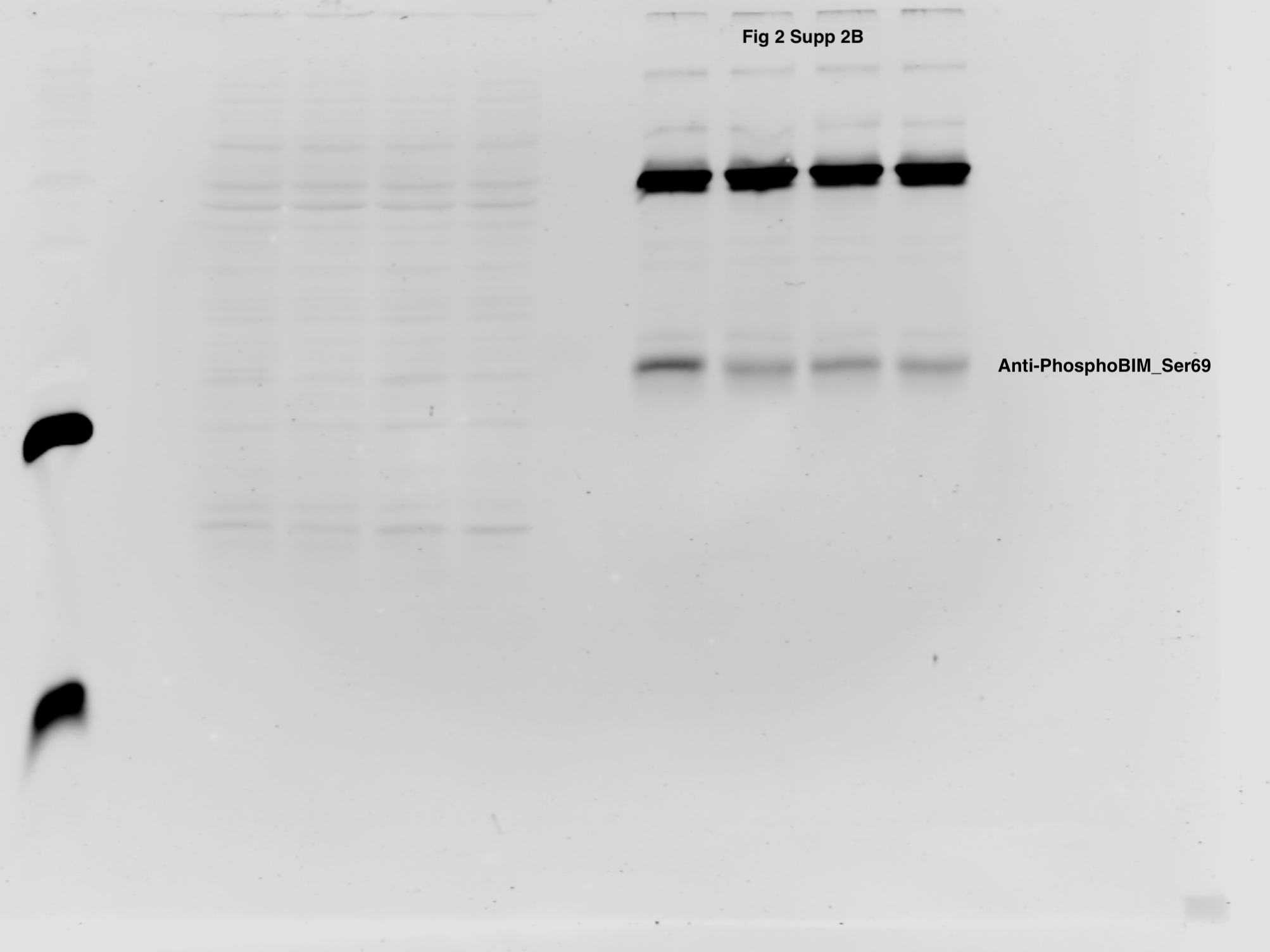

Supplement: Figure 2—figure supplement 1—source data 1. [file elife-82860-fig2-figsupp1-data1.zip › elife_Fig 2 Supp 1 source data/elife_Fig 2 Supp 1 source data 2/Fig_2_Supp_2B_Source_Data_Labeled/Fig_2_Supp_2B_BIM_Ser69_labeled.tif]

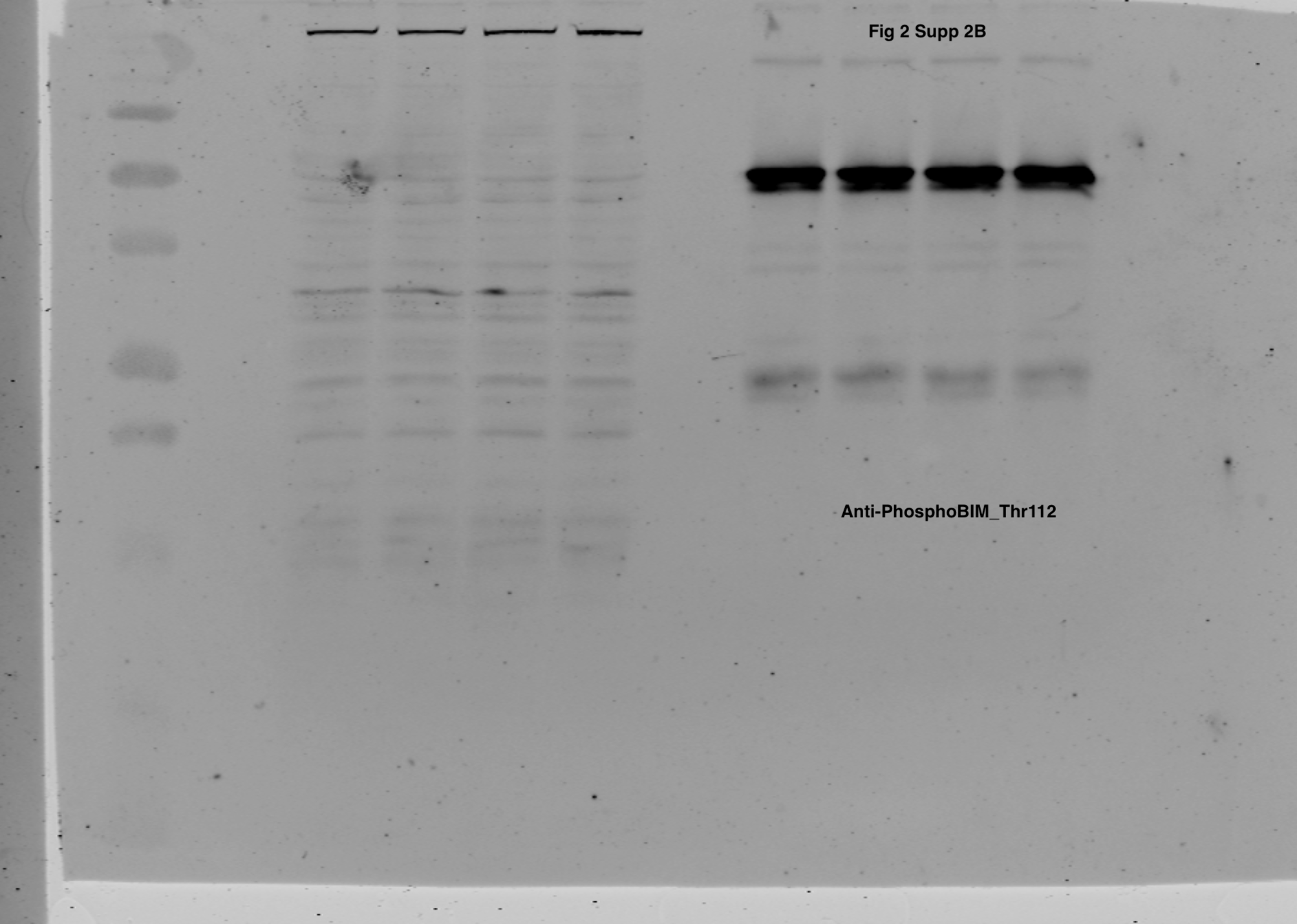

Supplement: Figure 2—figure supplement 1—source data 1. [file elife-82860-fig2-figsupp1-data1.zip › elife_Fig 2 Supp 1 source data/elife_Fig 2 Supp 1 source data 2/Fig_2_Supp_2B_Source_Data_Labeled/Fig_2_Supp_2B_BIM_Thr112_labeled.tif]

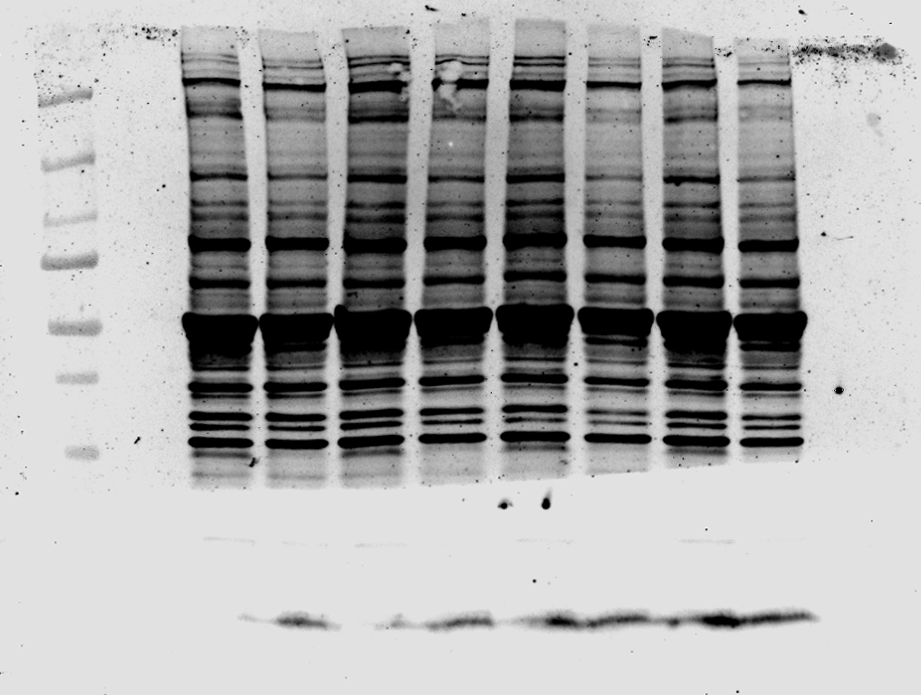

Supplement: Figure 2—figure supplement 2—source data 1. [file elife-82860-fig2-figsupp2-data1.zip › elife_Fig 2 Supp 2 source data 1/Fig_2_F2S2 Source Data Unlabeled/Fig_2_F2S2D MARCH5 unlabeled.tif]

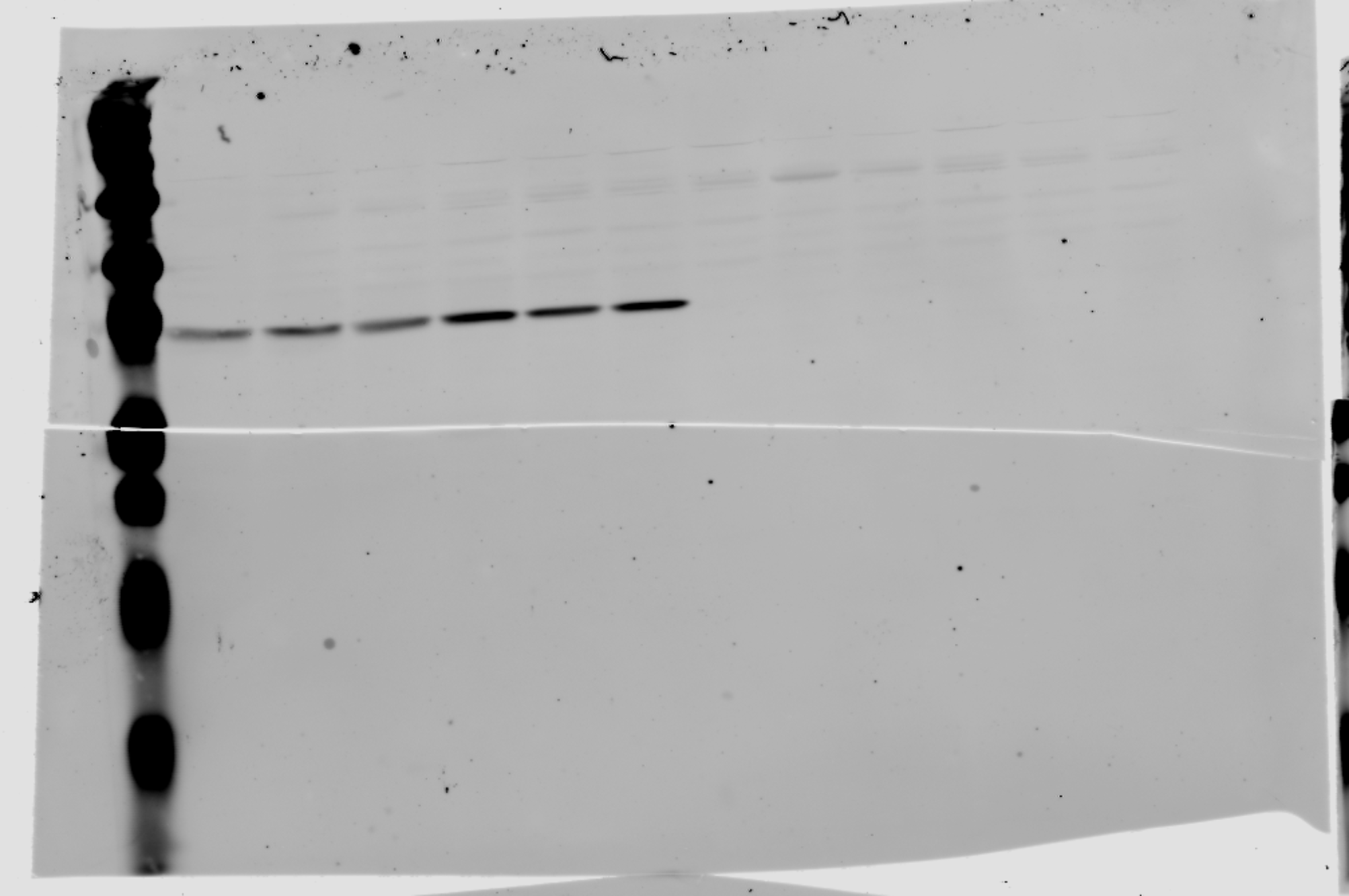

Supplement: Figure 2—figure supplement 2—source data 1. [file elife-82860-fig2-figsupp2-data1.zip › elife_Fig 2 Supp 2 source data 1/Fig_2_F2S2 Source Data Unlabeled/Fig_2_F2S2A ATAD1 unlabeled.tif]

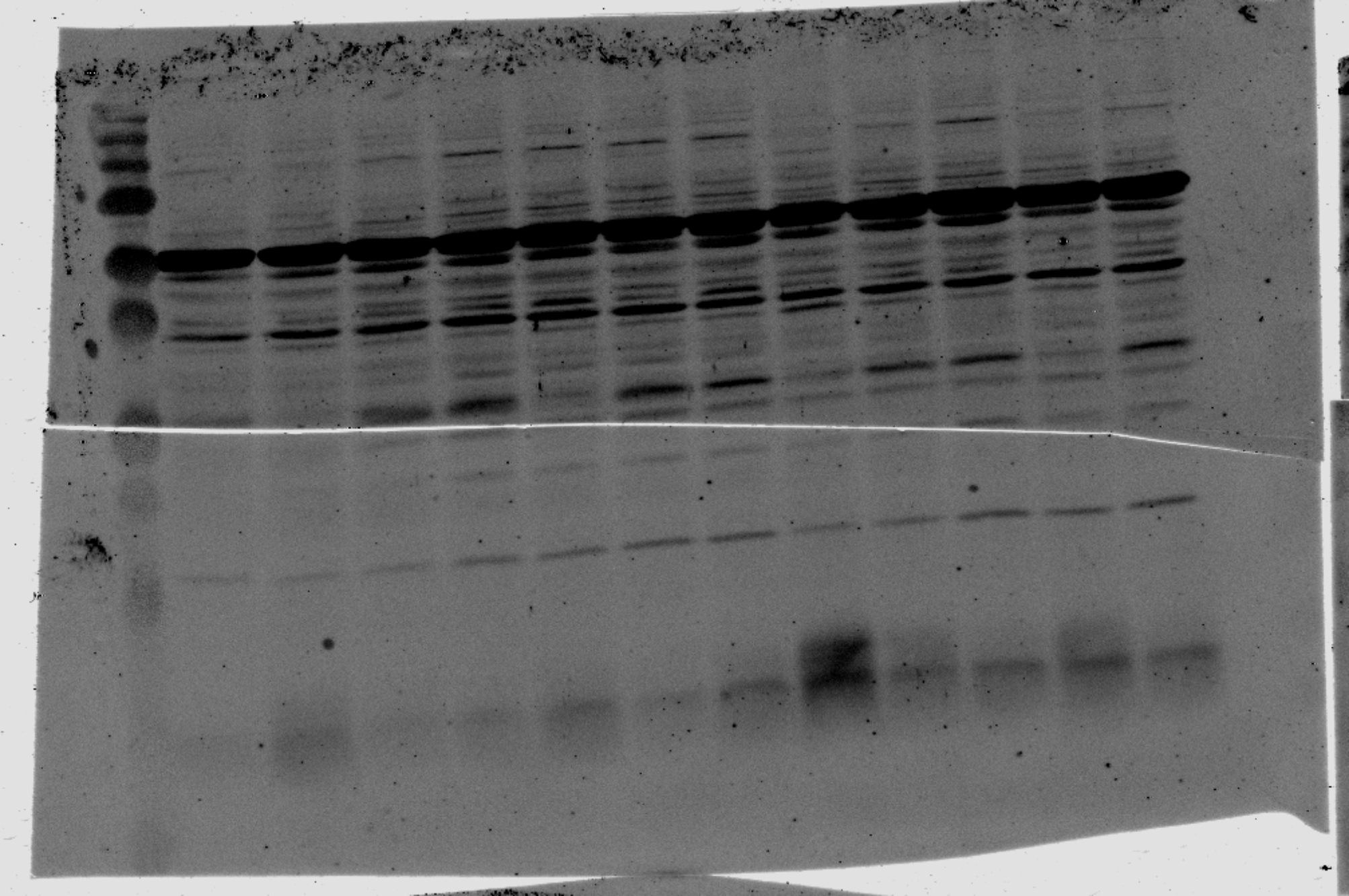

Supplement: Figure 2—figure supplement 2—source data 1. [file elife-82860-fig2-figsupp2-data1.zip › elife_Fig 2 Supp 2 source data 1/Fig_2_F2S2 Source Data Unlabeled/Fig_2_F2S2A NOXA unlabeled.tif]

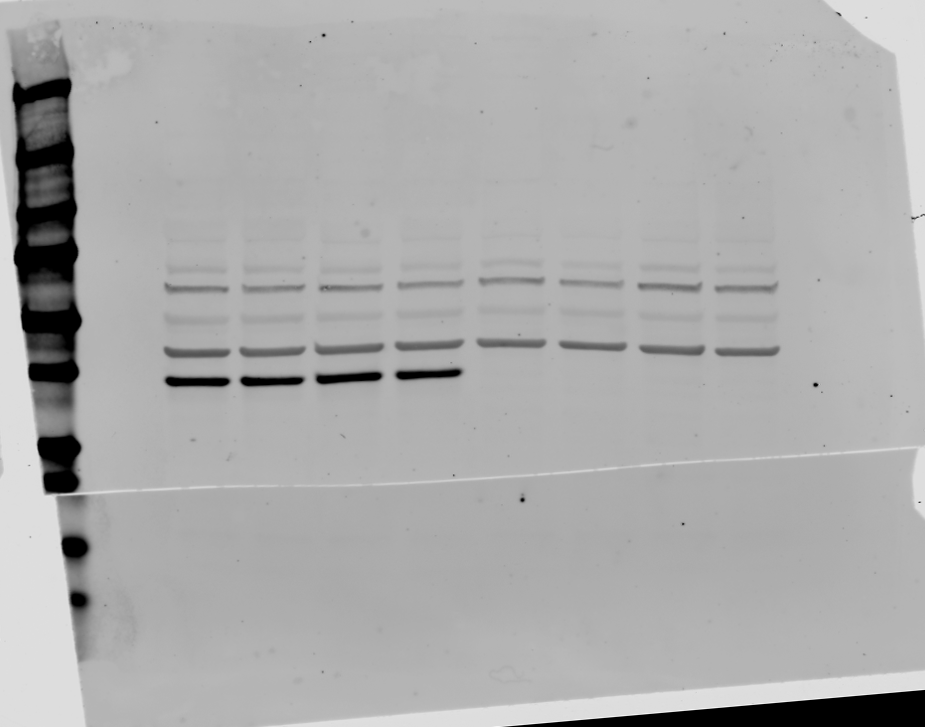

Supplement: Figure 2—figure supplement 2—source data 1. [file elife-82860-fig2-figsupp2-data1.zip › elife_Fig 2 Supp 2 source data 1/Fig_2_F2S2 Source Data Unlabeled/Fig_2_F2S2D ATAD1 Actin unlabeled.tif]

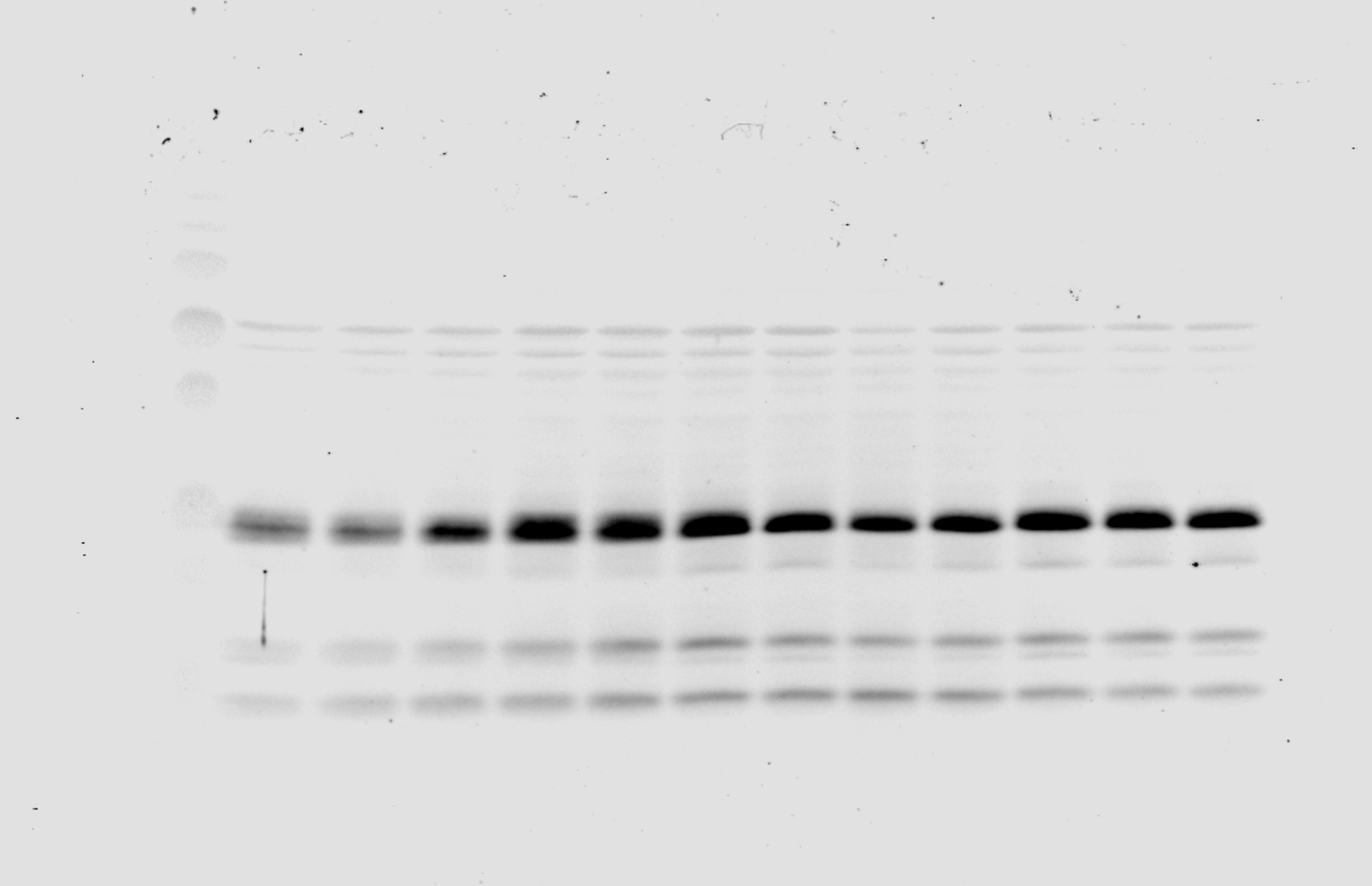

Supplement: Figure 2—figure supplement 2—source data 1. [file elife-82860-fig2-figsupp2-data1.zip › elife_Fig 2 Supp 2 source data 1/Fig_2_F2S2 Source Data Unlabeled/Fig_2_F2S2A BIM unlabeled.tif.tif]

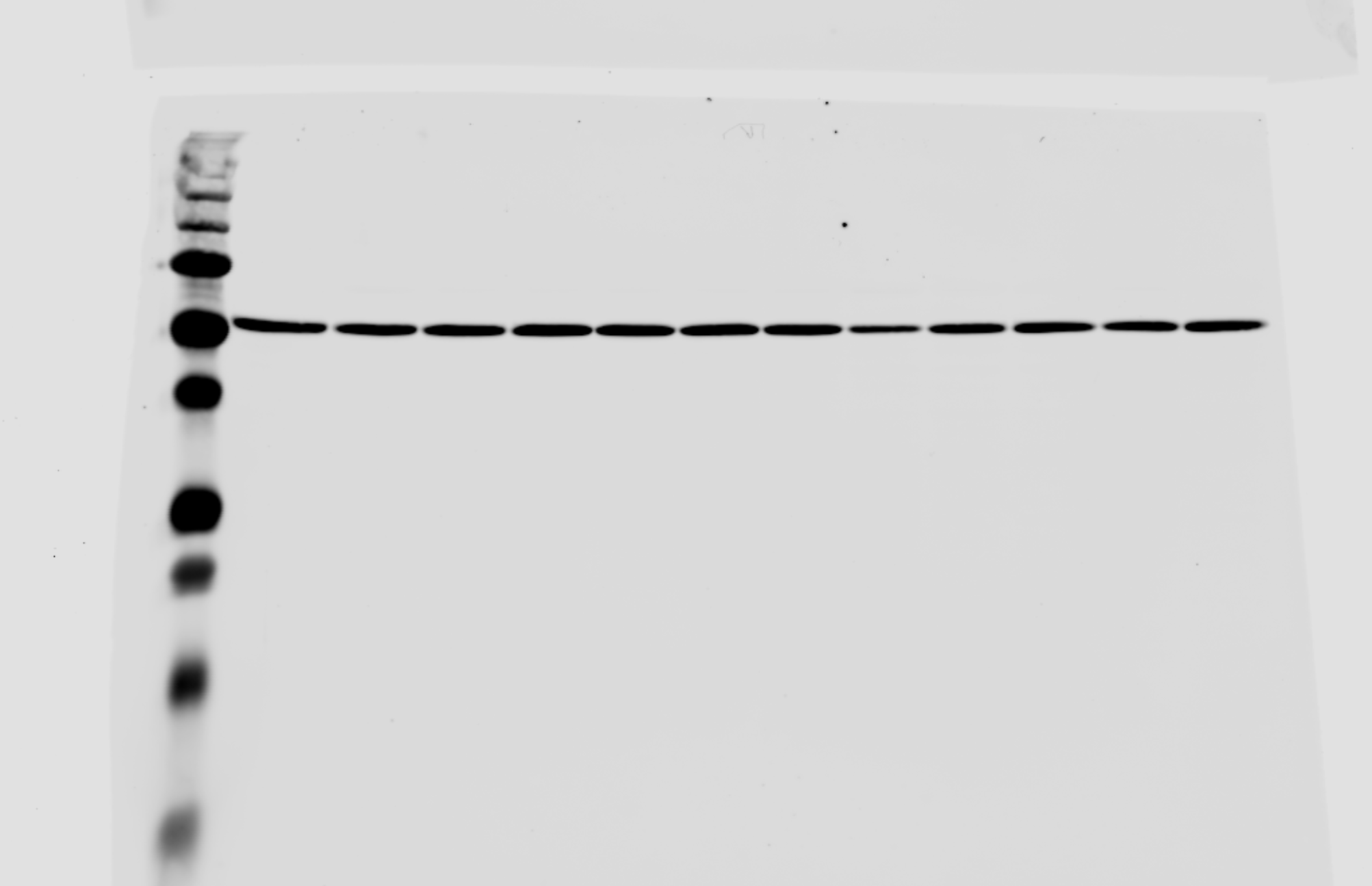

Supplement: Figure 2—figure supplement 2—source data 1. [file elife-82860-fig2-figsupp2-data1.zip › elife_Fig 2 Supp 2 source data 1/Fig_2_F2S2 Source Data Unlabeled/Fig_2_F2S2A actin unlabeled.tif.tif]

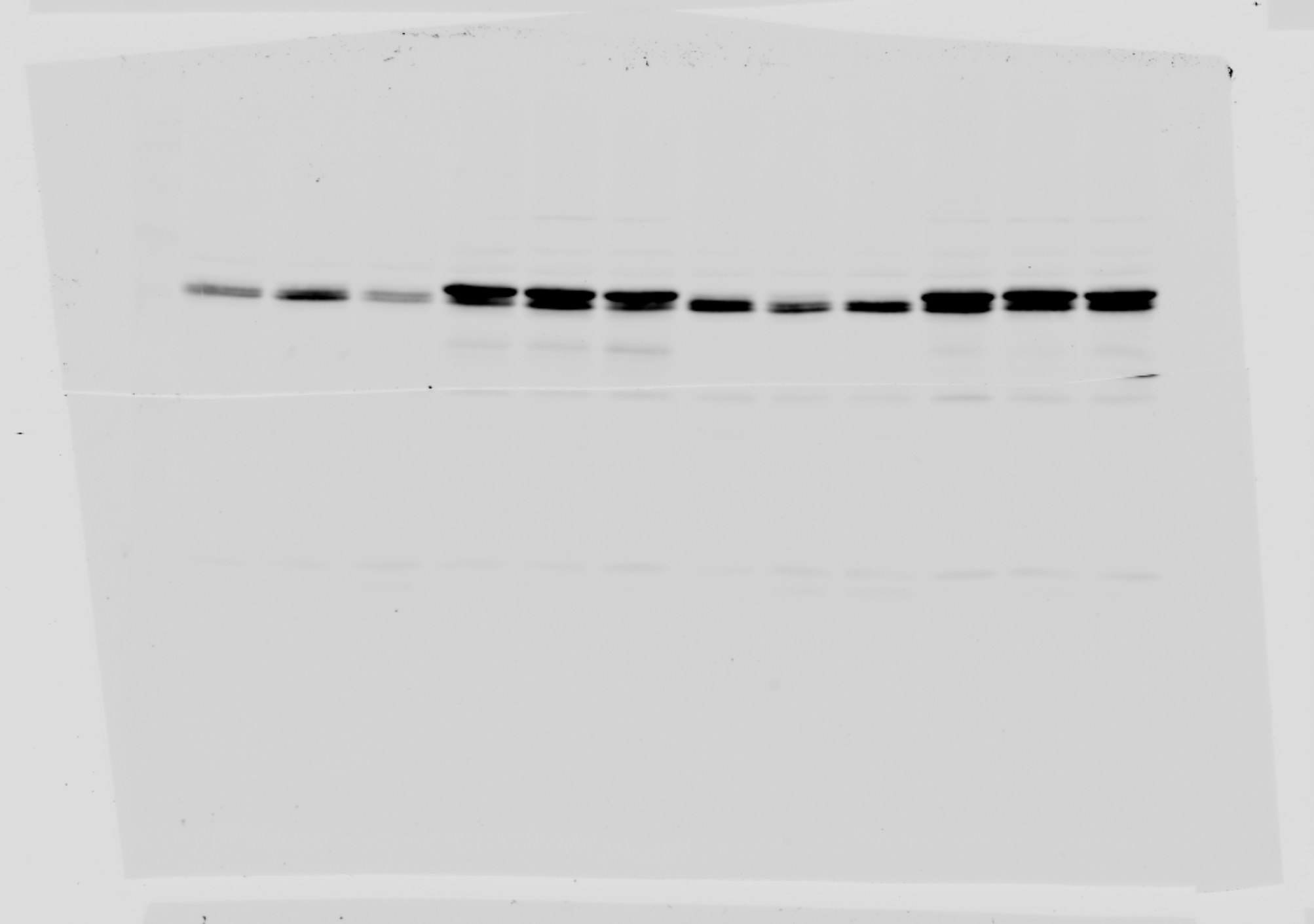

Supplement: Figure 2—figure supplement 2—source data 1. [file elife-82860-fig2-figsupp2-data1.zip › elife_Fig 2 Supp 2 source data 1/Fig_2_F2S2 Source Data Unlabeled/Fig_2_F2S2A MCL1 unlabeled.tif.tif]

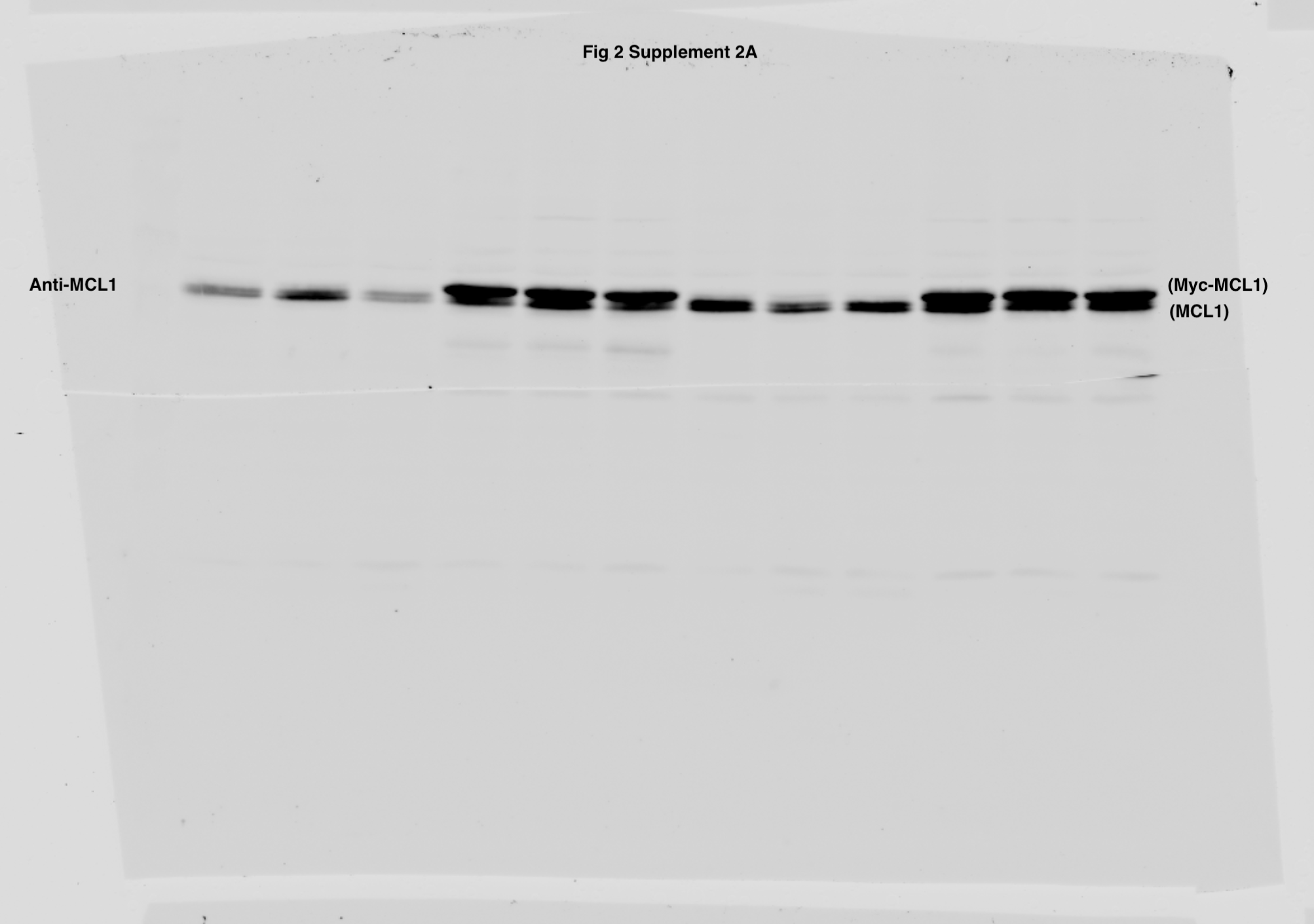

Supplement: Figure 2—figure supplement 2—source data 1. [file elife-82860-fig2-figsupp2-data1.zip › elife_Fig 2 Supp 2 source data 1/Fig_2_F2S2 Source Data Labeled/Fig_2_F2S2A MCL1 labeled.tif]

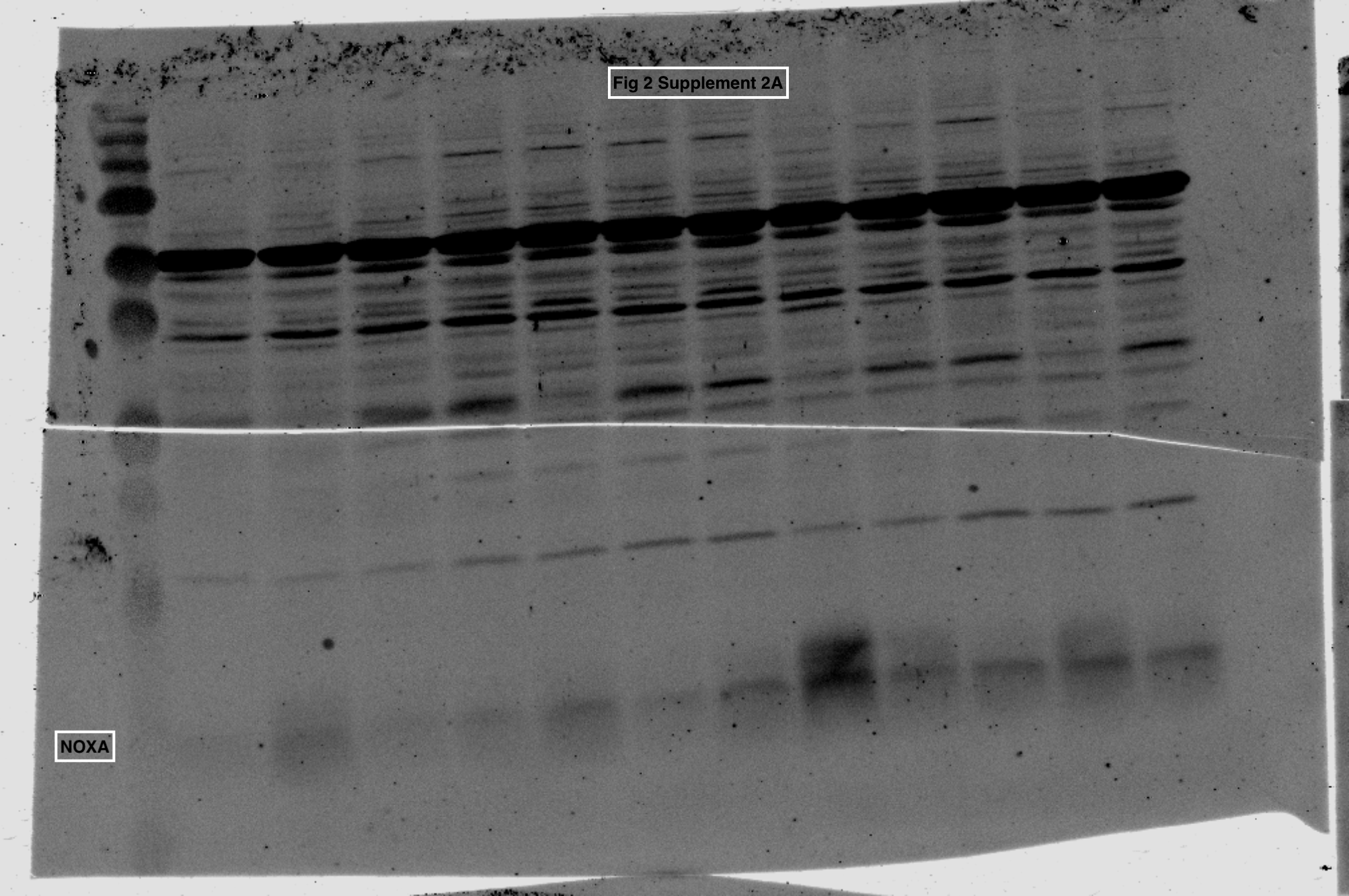

Supplement: Figure 2—figure supplement 2—source data 1. [file elife-82860-fig2-figsupp2-data1.zip › elife_Fig 2 Supp 2 source data 1/Fig_2_F2S2 Source Data Labeled/Fig_2_F2S2A NOXA labeled.tif]

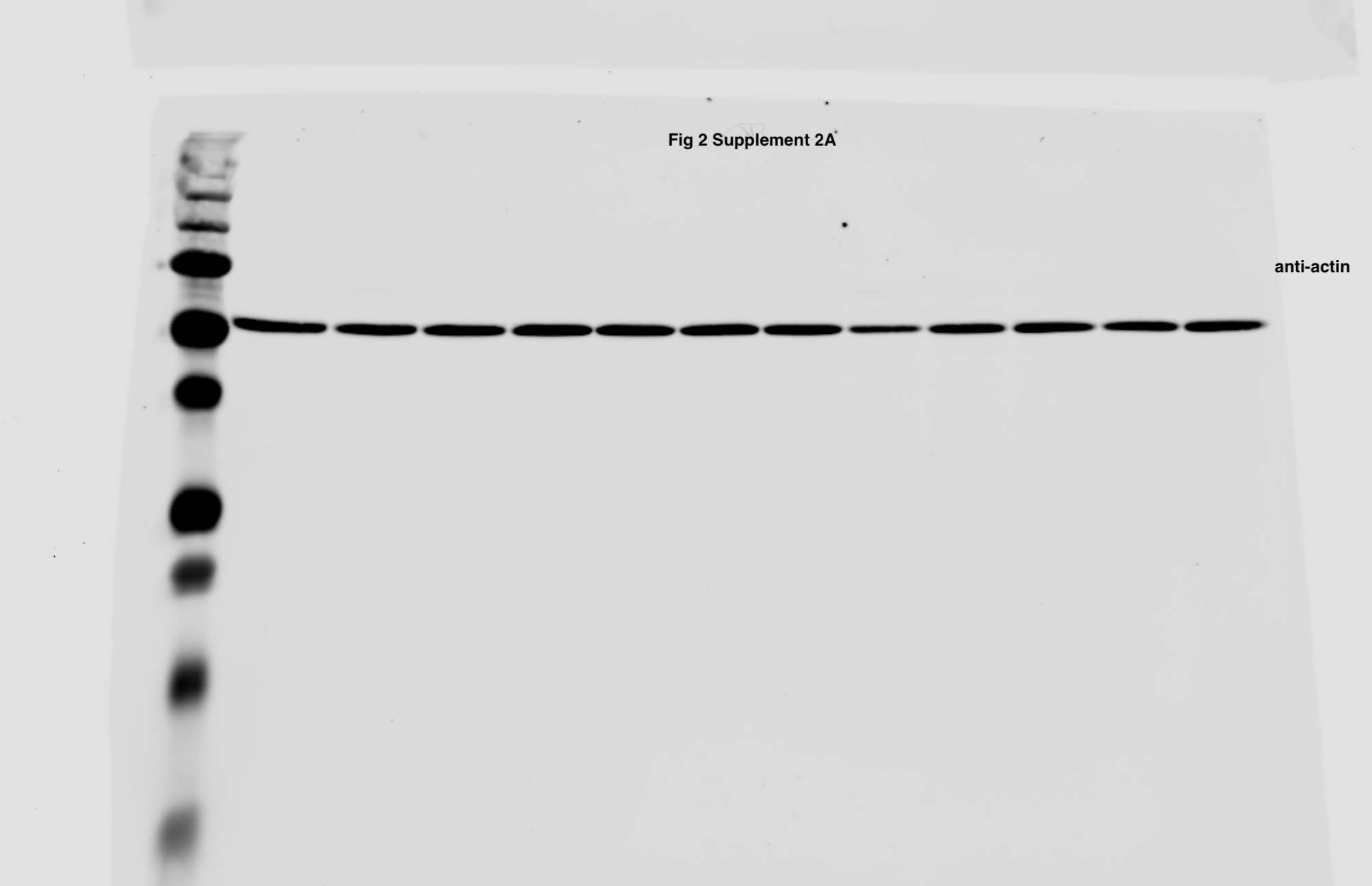

Supplement: Figure 2—figure supplement 2—source data 1. [file elife-82860-fig2-figsupp2-data1.zip › elife_Fig 2 Supp 2 source data 1/Fig_2_F2S2 Source Data Labeled/Fig_2_F2S2A actin labeled.tif]

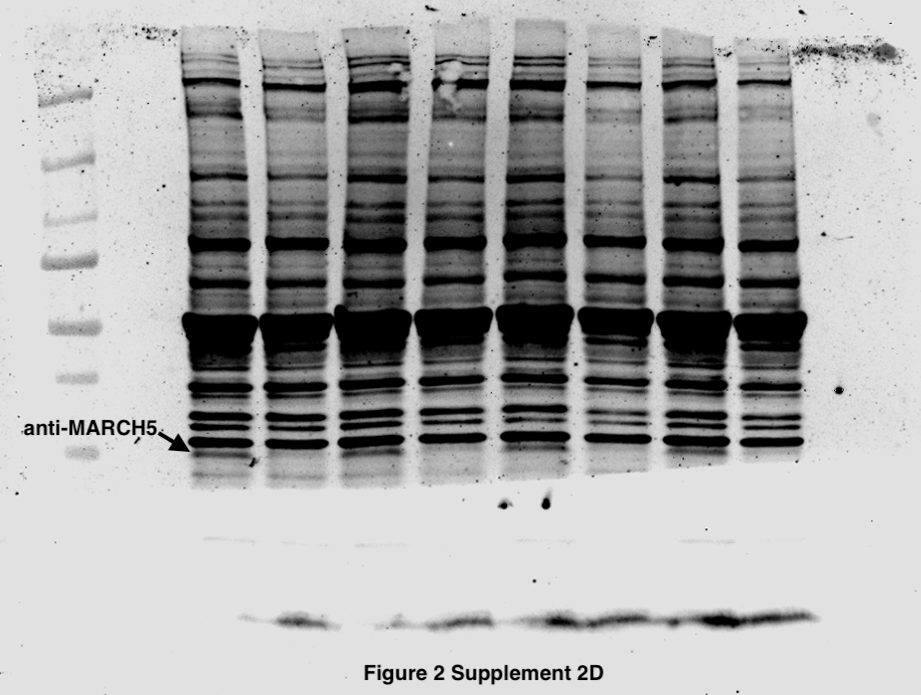

Supplement: Figure 2—figure supplement 2—source data 1. [file elife-82860-fig2-figsupp2-data1.zip › elife_Fig 2 Supp 2 source data 1/Fig_2_F2S2 Source Data Labeled/Fig_2_F2S2D MARCH5 labeled.tiff]

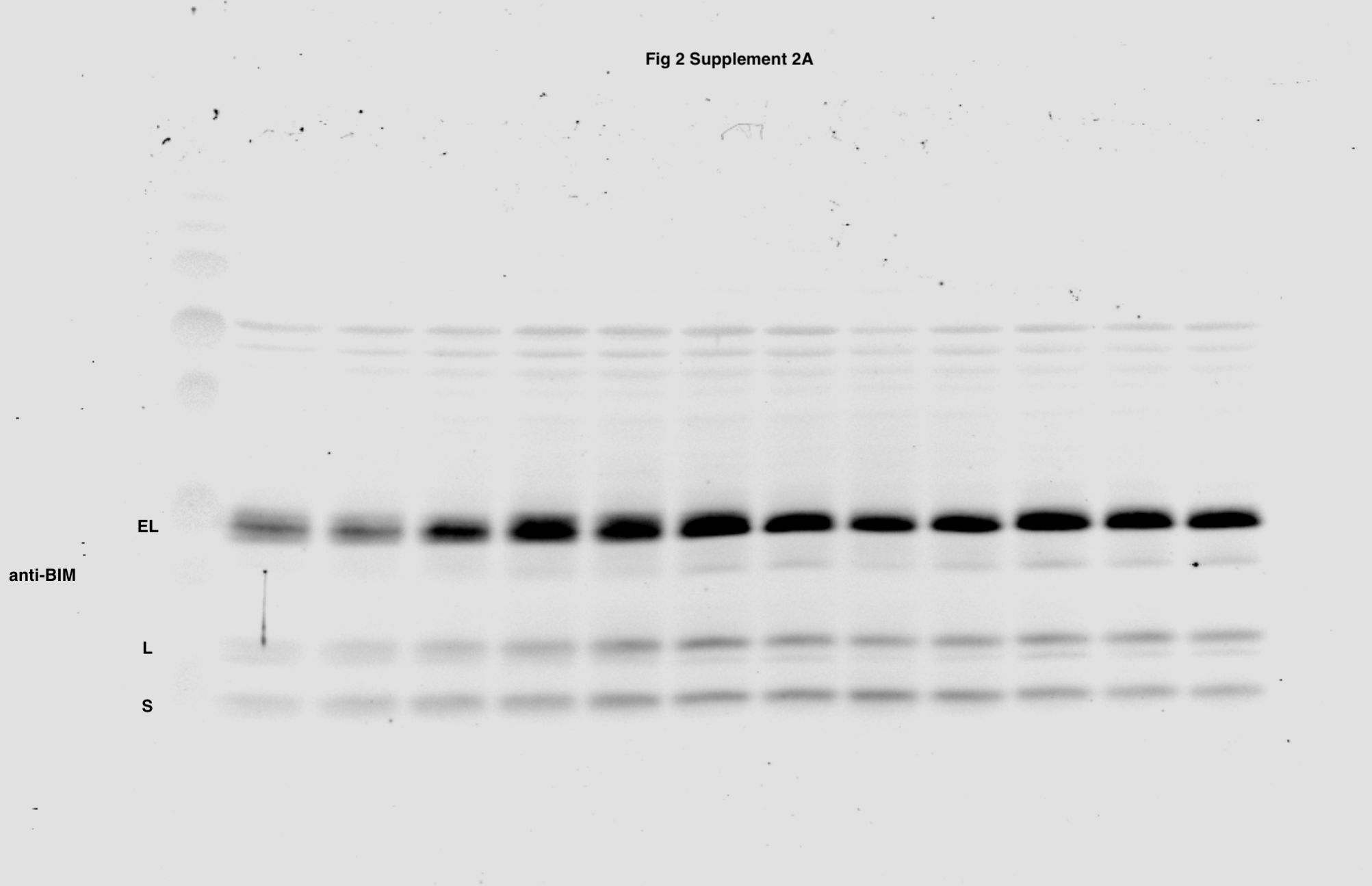

Supplement: Figure 2—figure supplement 2—source data 1. [file elife-82860-fig2-figsupp2-data1.zip › elife_Fig 2 Supp 2 source data 1/Fig_2_F2S2 Source Data Labeled/Fig_2_F2S2A BIM labeled.tif]

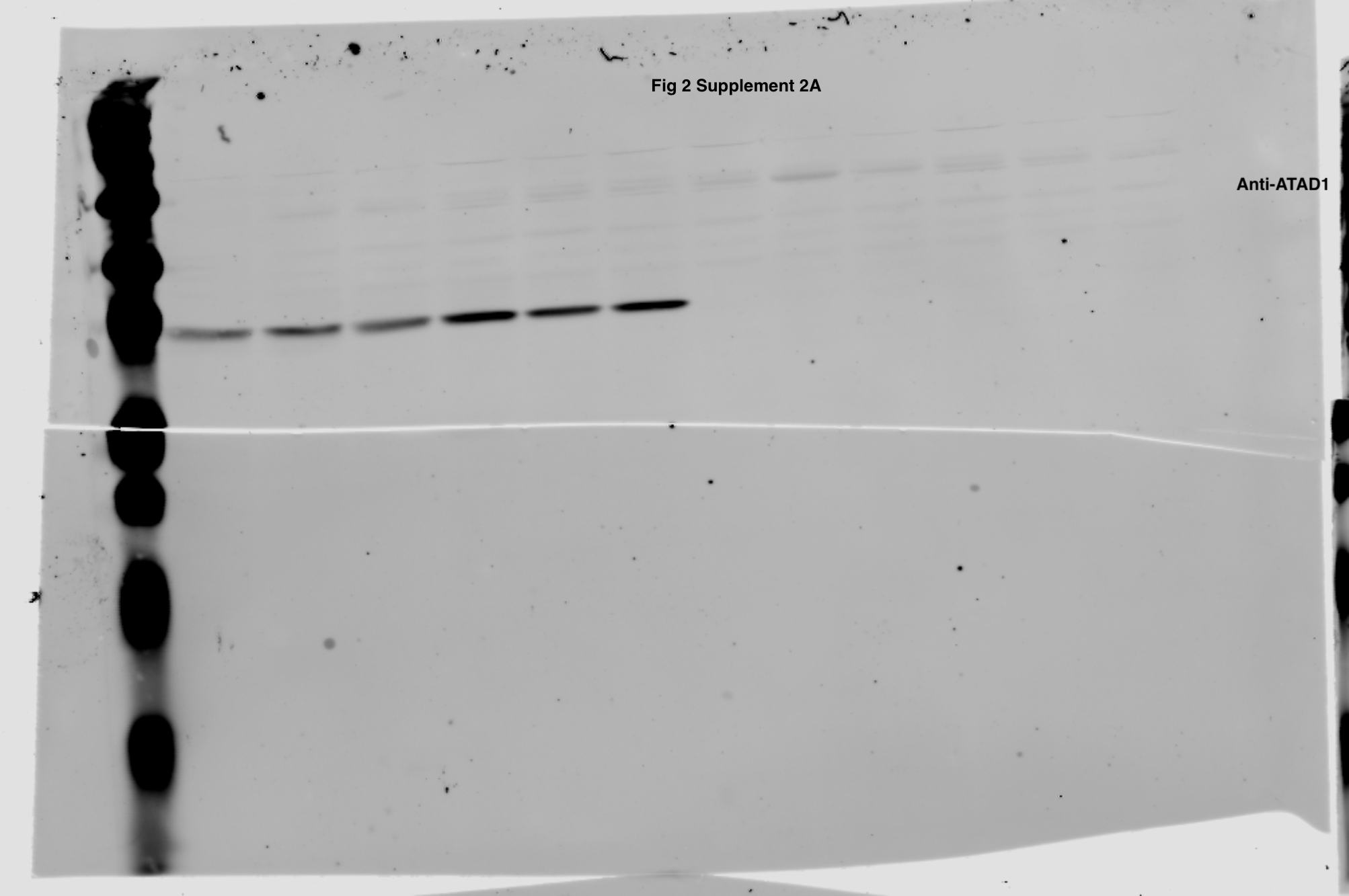

Supplement: Figure 2—figure supplement 2—source data 1. [file elife-82860-fig2-figsupp2-data1.zip › elife_Fig 2 Supp 2 source data 1/Fig_2_F2S2 Source Data Labeled/Fig_2_F2S2A ATAD1 labeled.tif]

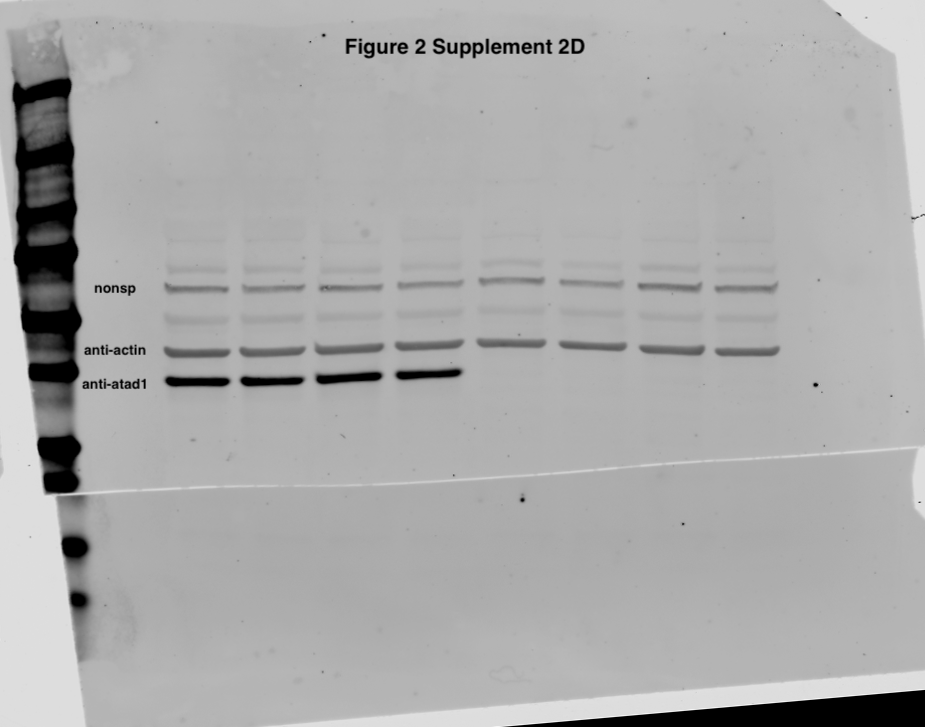

Supplement: Figure 2—figure supplement 2—source data 1. [file elife-82860-fig2-figsupp2-data1.zip › elife_Fig 2 Supp 2 source data 1/Fig_2_F2S2 Source Data Labeled/Fig_2_F2S2D ATAD1 Actin labeled.tiff]

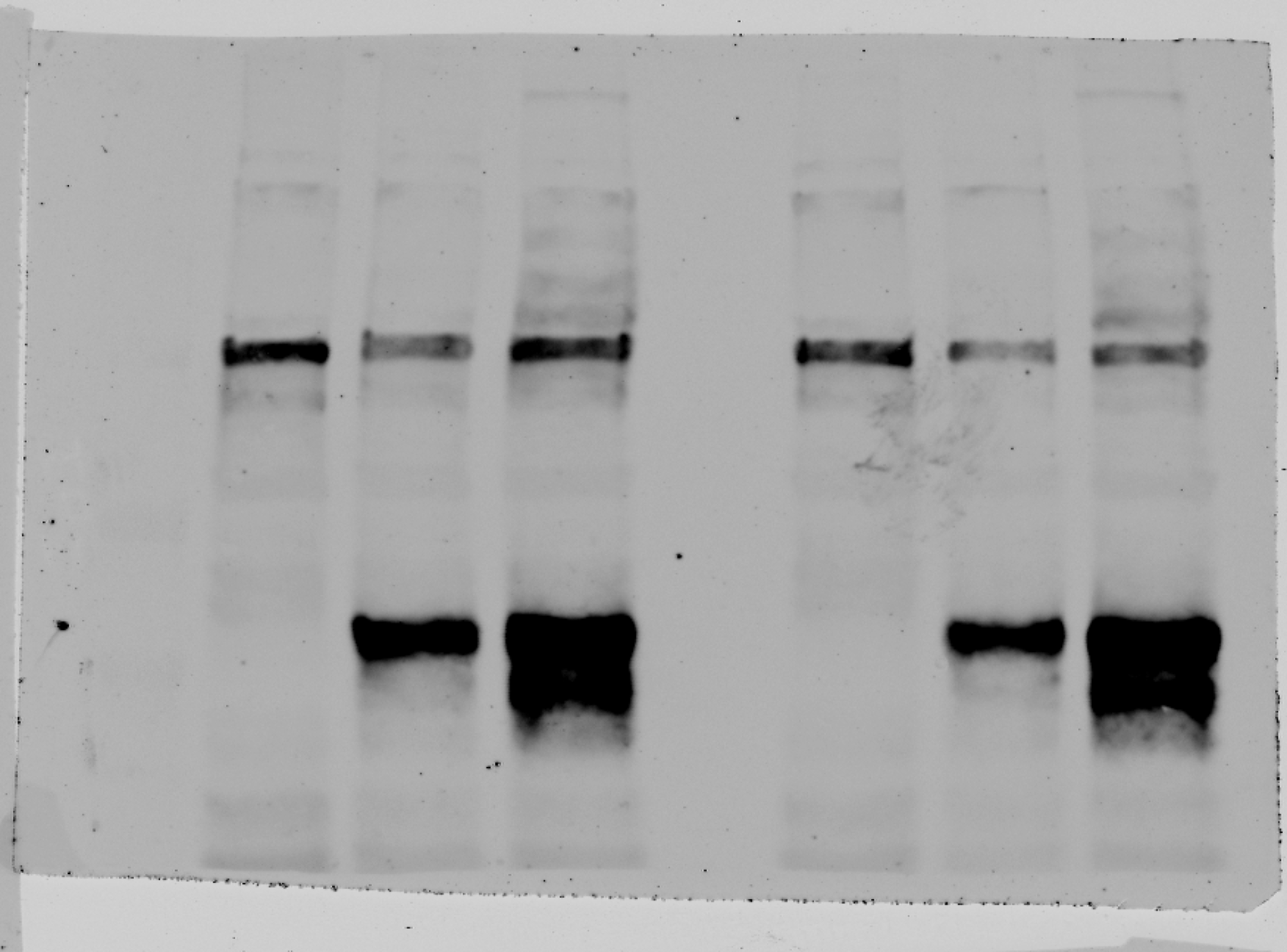

Supplement: Figure 2—figure supplement 3—source data 1. [file elife-82860-fig2-figsupp3-data1.zip › elife_Fig 2 Supp 3 source data/elife_Fig 2 Supp 3 source data 2/Fig_2_Supp_3D_Source_Data_Unlabeled/Fig_2_Supp_3D_flag input_Unlabeled.tif]

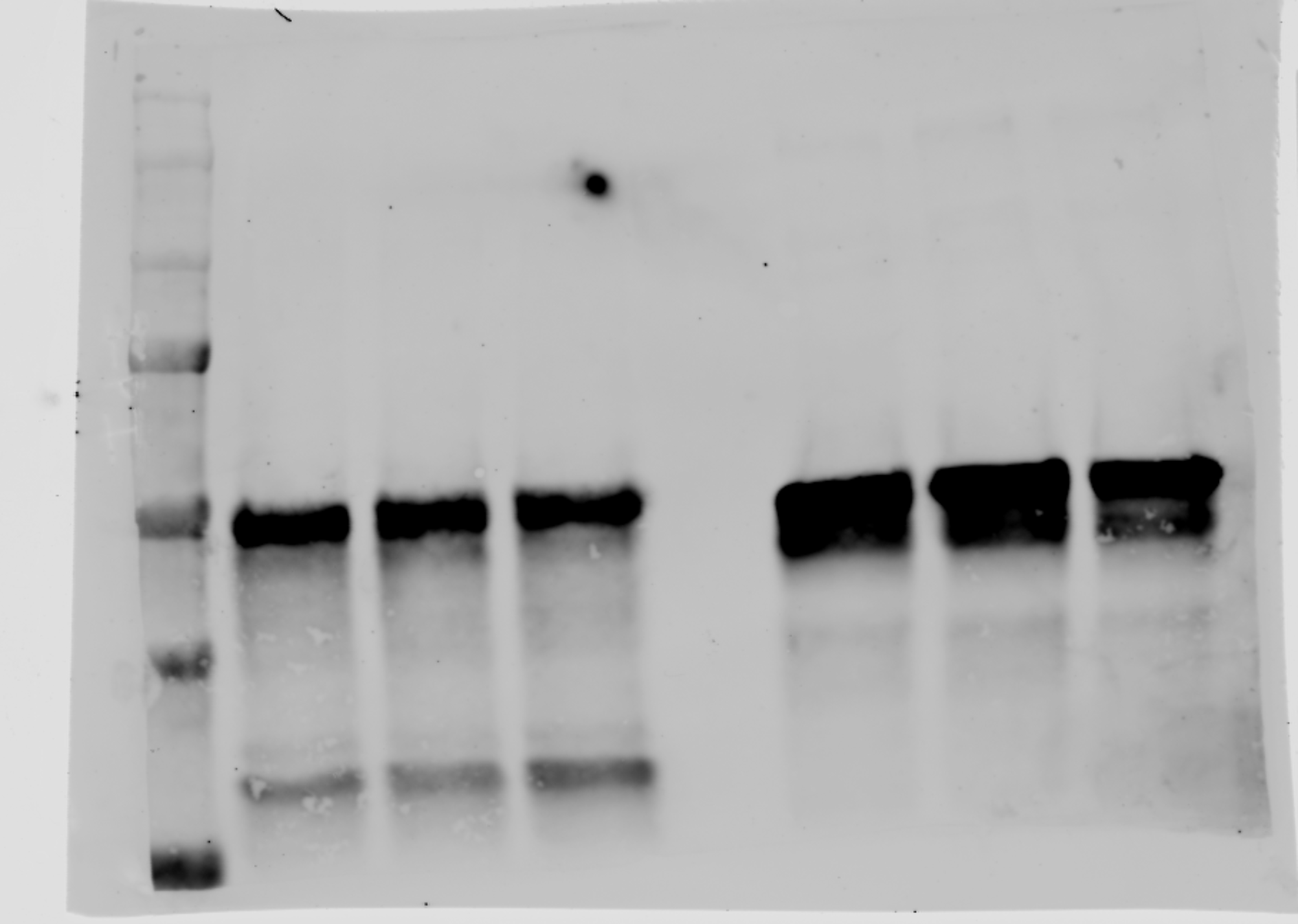

Supplement: Figure 2—figure supplement 3—source data 1. [file elife-82860-fig2-figsupp3-data1.zip › elife_Fig 2 Supp 3 source data/elife_Fig 2 Supp 3 source data 2/Fig_2_Supp_3D_Source_Data_Unlabeled/Fig_2_Supp_3D_GFP_input_Unlabeled.tif]

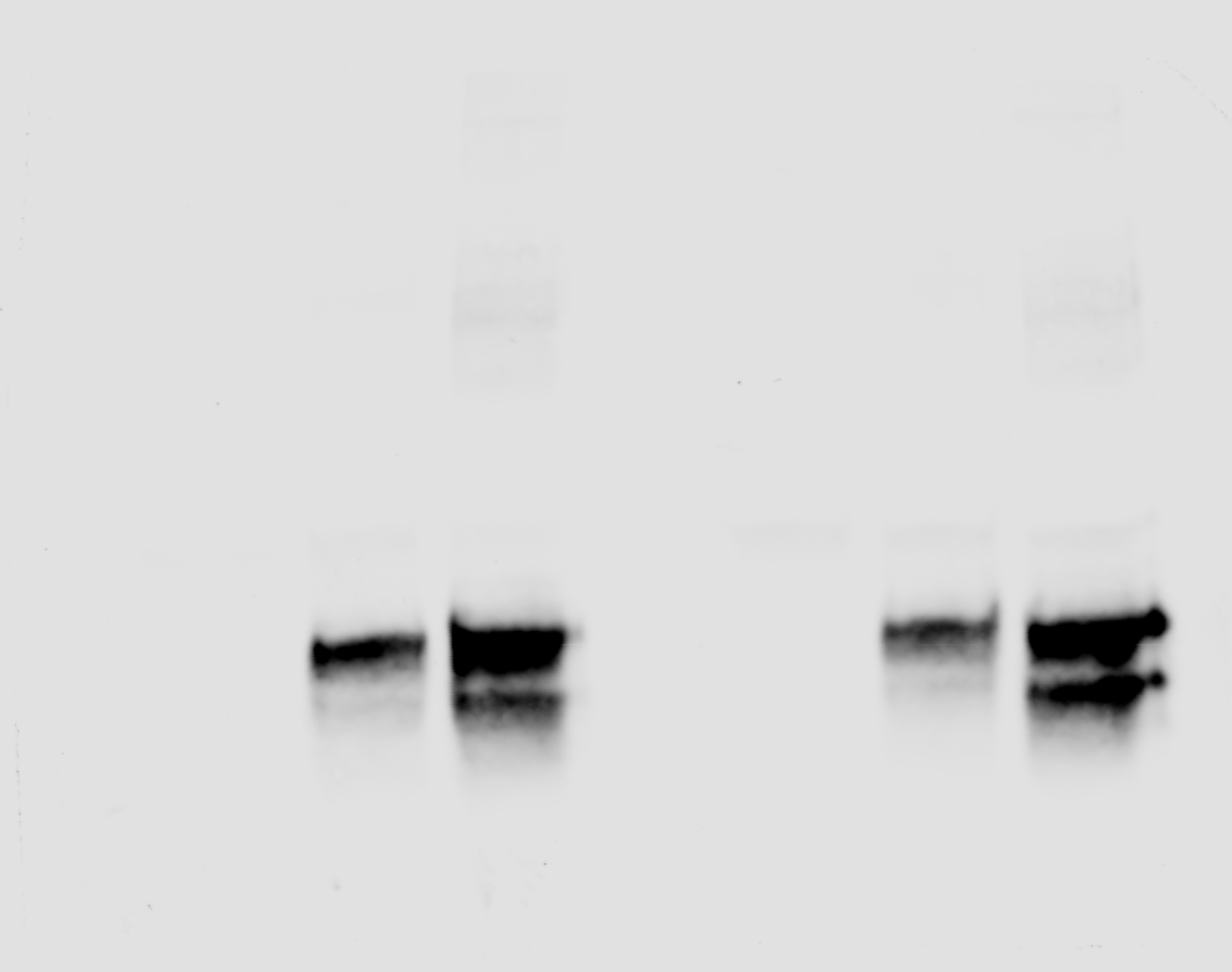

Supplement: Figure 2—figure supplement 3—source data 1. [file elife-82860-fig2-figsupp3-data1.zip › elife_Fig 2 Supp 3 source data/elife_Fig 2 Supp 3 source data 2/Fig_2_Supp_3D_Source_Data_Unlabeled/Fig_2_Supp_3D_flag_ip_blot_Unlabeled.tif]

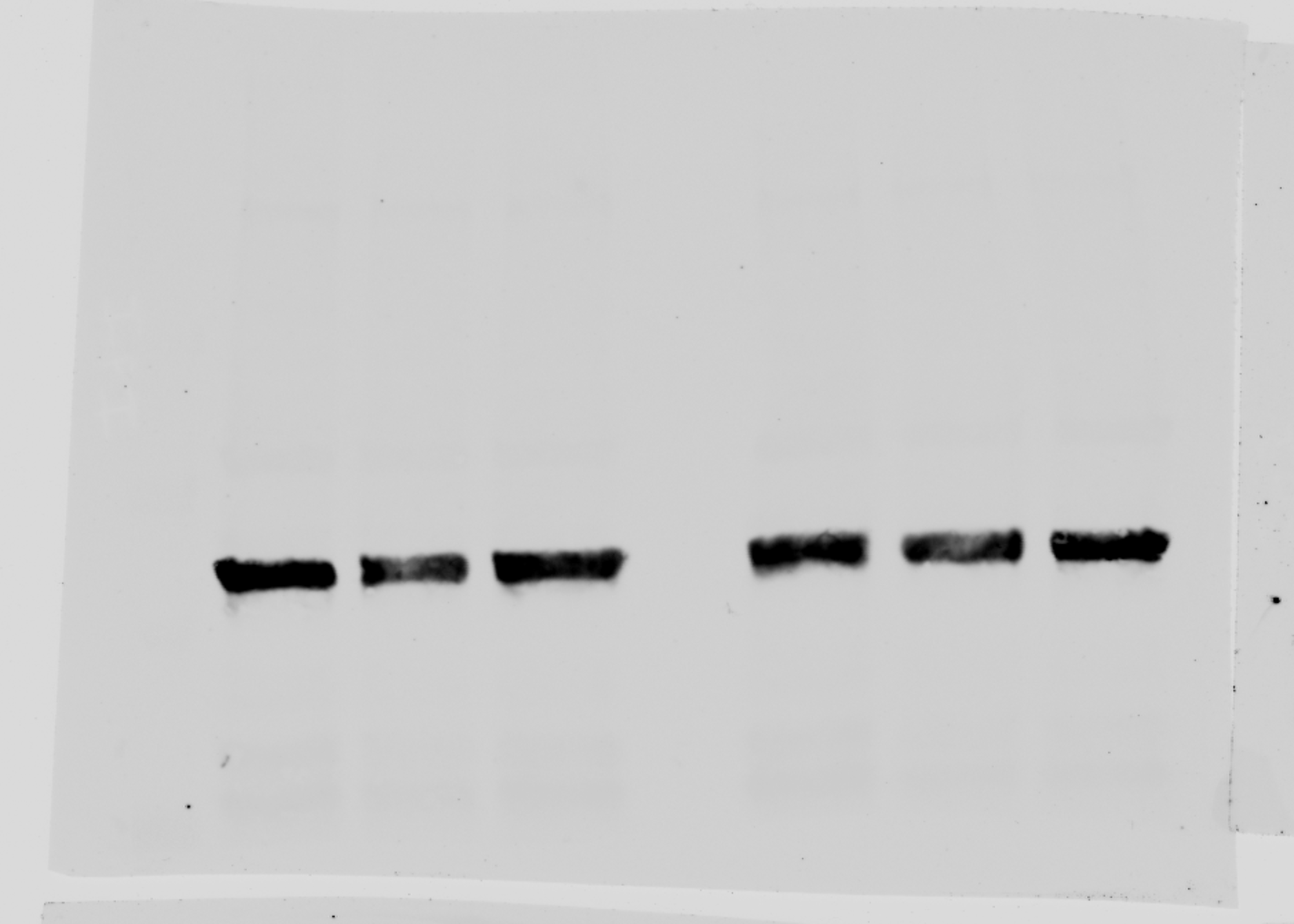

Supplement: Figure 2—figure supplement 3—source data 1. [file elife-82860-fig2-figsupp3-data1.zip › elife_Fig 2 Supp 3 source data/elife_Fig 2 Supp 3 source data 2/Fig_2_Supp_3D_Source_Data_Unlabeled/Fig_2_Supp_3D_actin input_Unlabeled.tif]

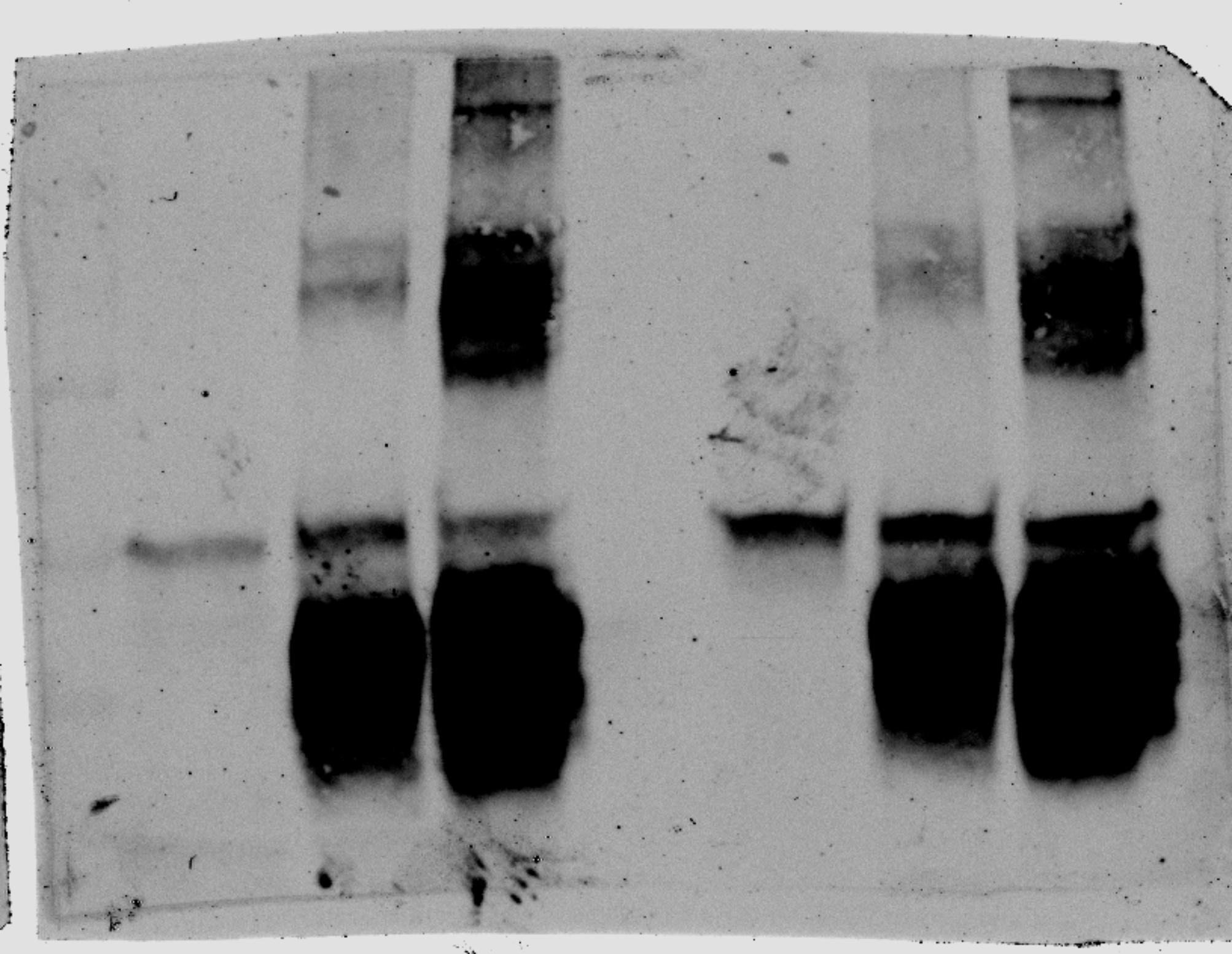

Supplement: Figure 2—figure supplement 3—source data 1. [file elife-82860-fig2-figsupp3-data1.zip › elife_Fig 2 Supp 3 source data/elife_Fig 2 Supp 3 source data 2/Fig_2_Supp_3D_Source_Data_Unlabeled/Fig_2_Supp_3D_GFP blot_Unlabeled.tif]

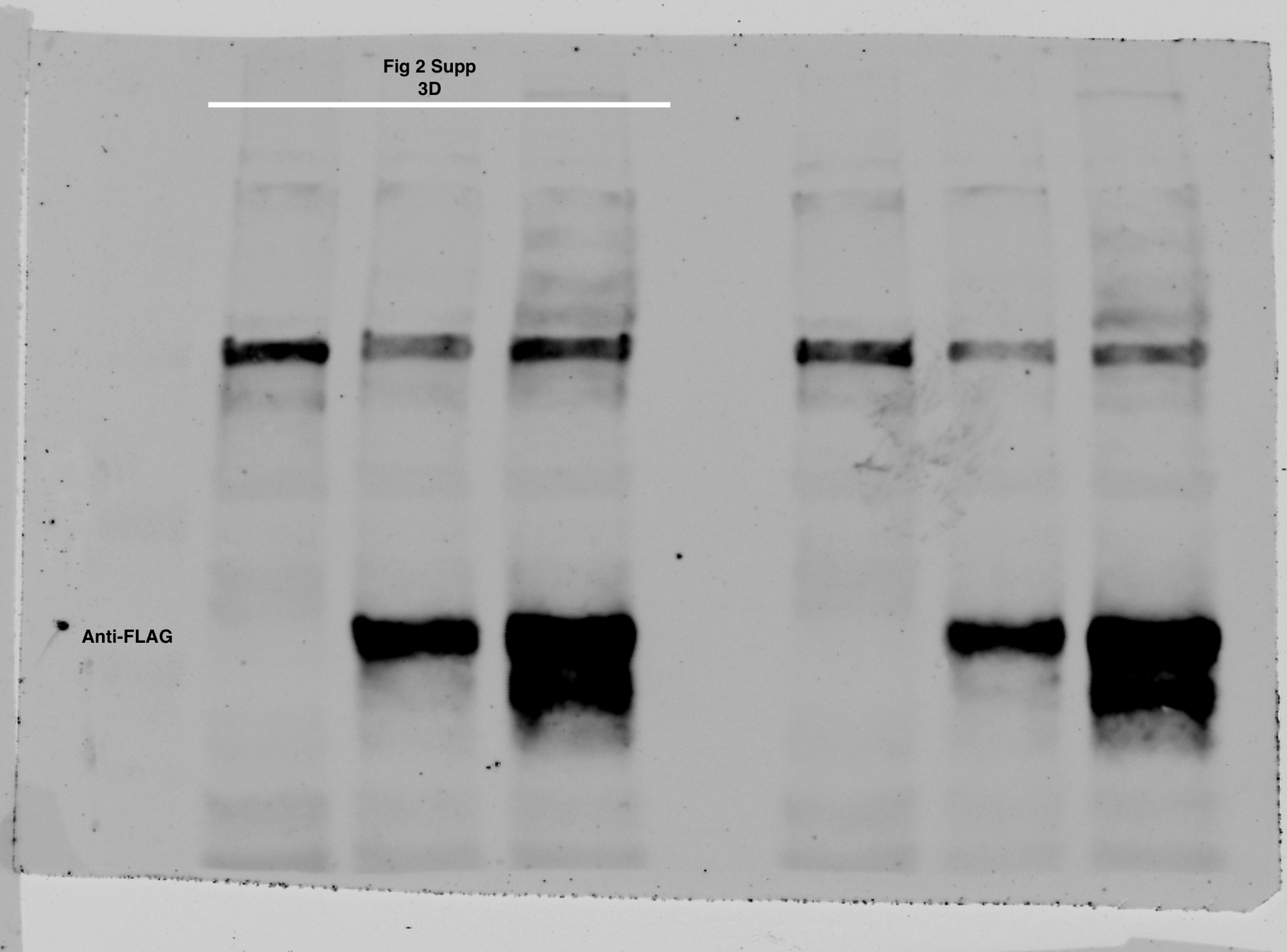

Supplement: Figure 2—figure supplement 3—source data 1. [file elife-82860-fig2-figsupp3-data1.zip › elife_Fig 2 Supp 3 source data/elife_Fig 2 Supp 3 source data 2/Fig_2_Supp_3D_Source_Data_Labeled/Fig_2_Supp_3D_flag input_labeled.tif]

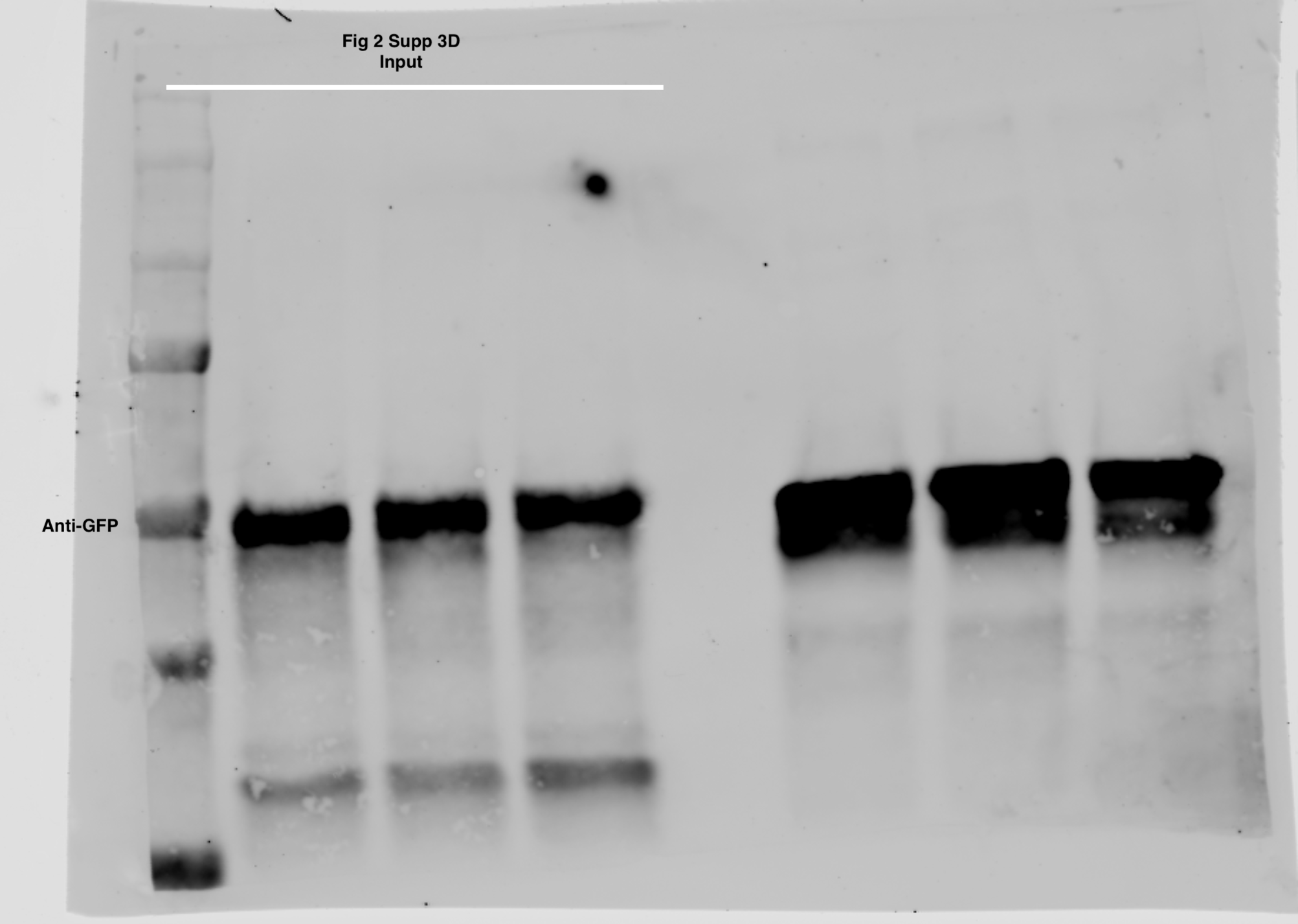

Supplement: Figure 2—figure supplement 3—source data 1. [file elife-82860-fig2-figsupp3-data1.zip › elife_Fig 2 Supp 3 source data/elife_Fig 2 Supp 3 source data 2/Fig_2_Supp_3D_Source_Data_Labeled/Fig_2_Supp_3D_GFP_input_labeled.tif]

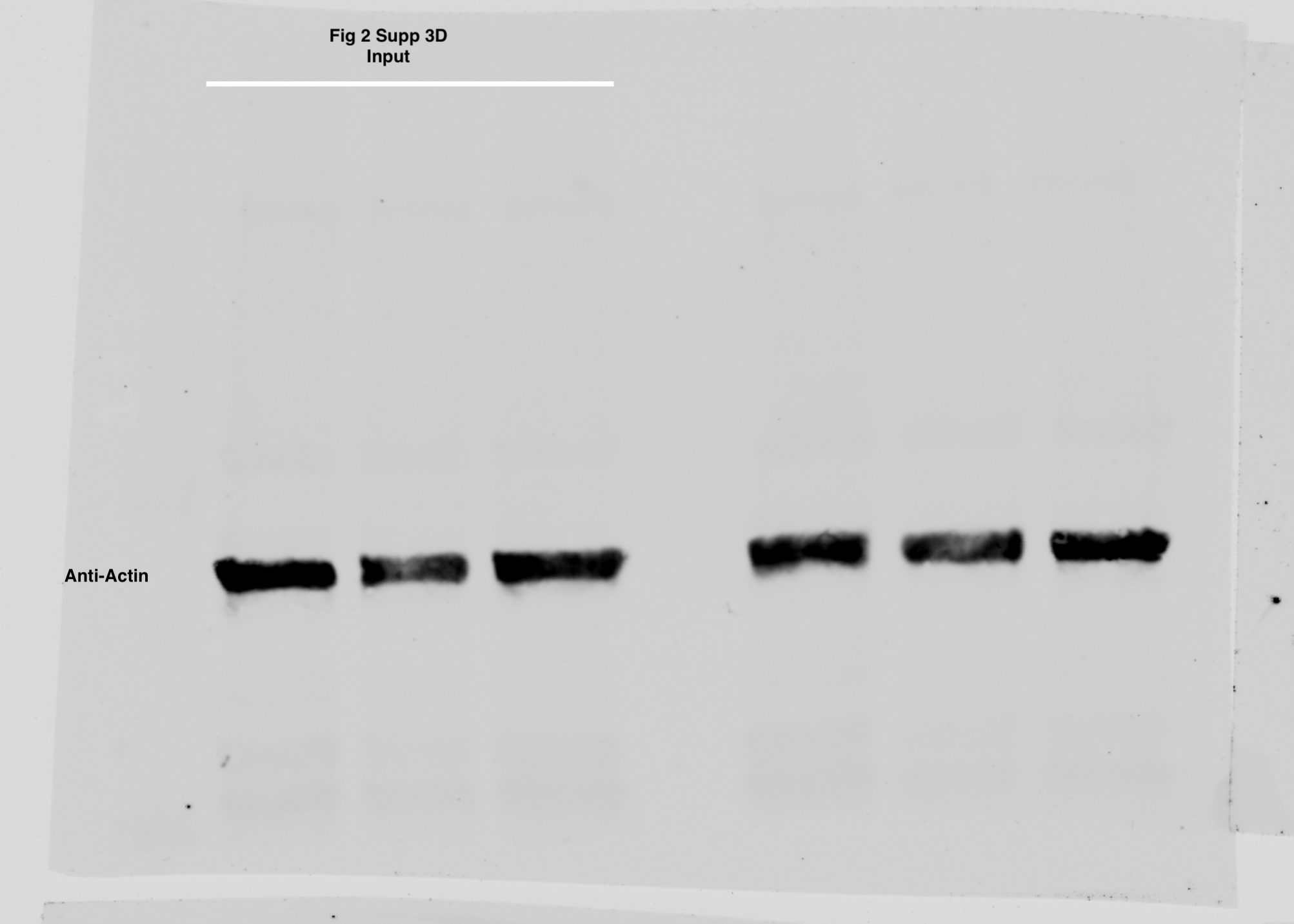

Supplement: Figure 2—figure supplement 3—source data 1. [file elife-82860-fig2-figsupp3-data1.zip › elife_Fig 2 Supp 3 source data/elife_Fig 2 Supp 3 source data 2/Fig_2_Supp_3D_Source_Data_Labeled/Fig_2_Supp_3D_actin input_labeled.tif]

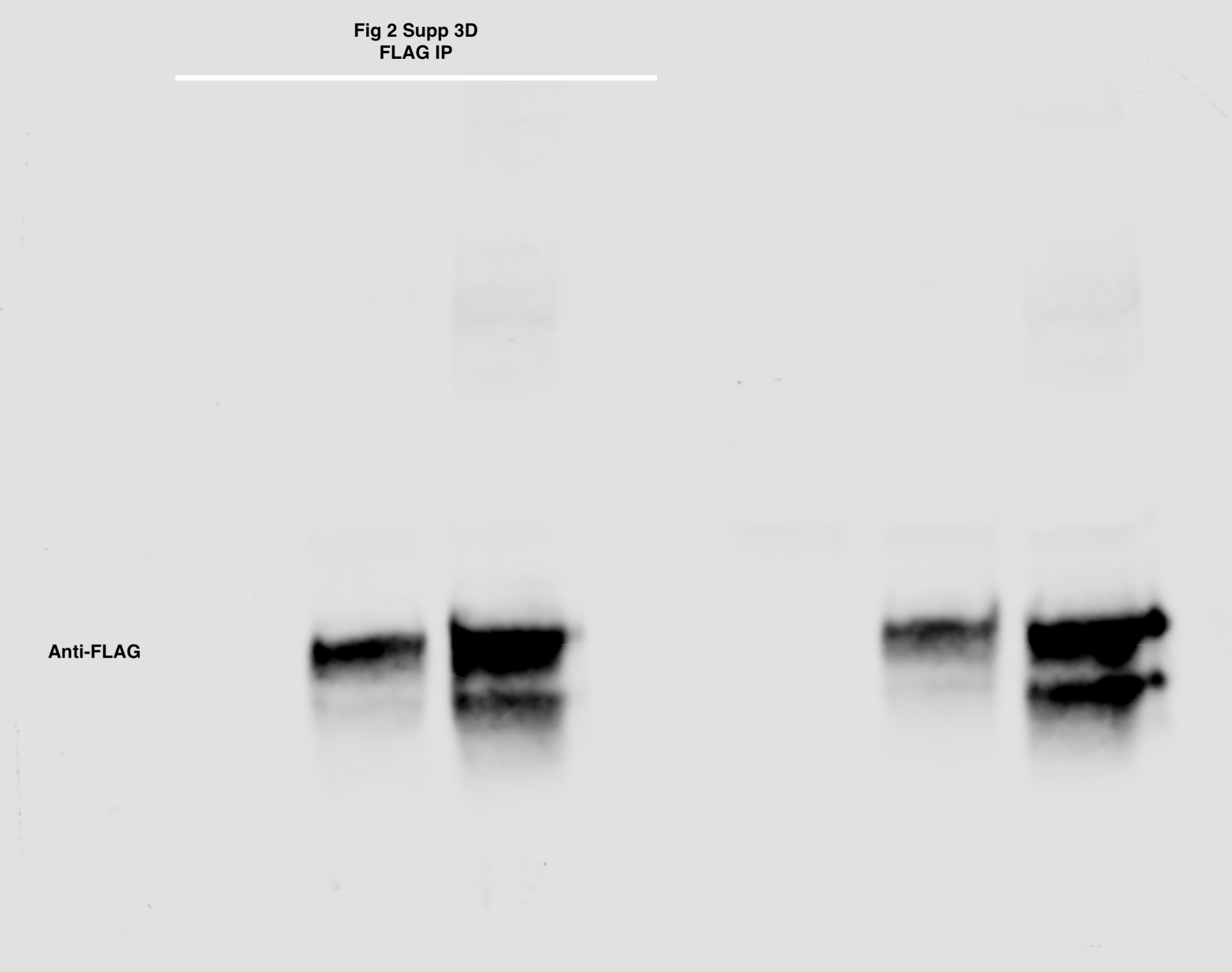

Supplement: Figure 2—figure supplement 3—source data 1. [file elife-82860-fig2-figsupp3-data1.zip › elife_Fig 2 Supp 3 source data/elife_Fig 2 Supp 3 source data 2/Fig_2_Supp_3D_Source_Data_Labeled/Fig_2_Supp_3D_flag_ip_blot_labeled.tif]

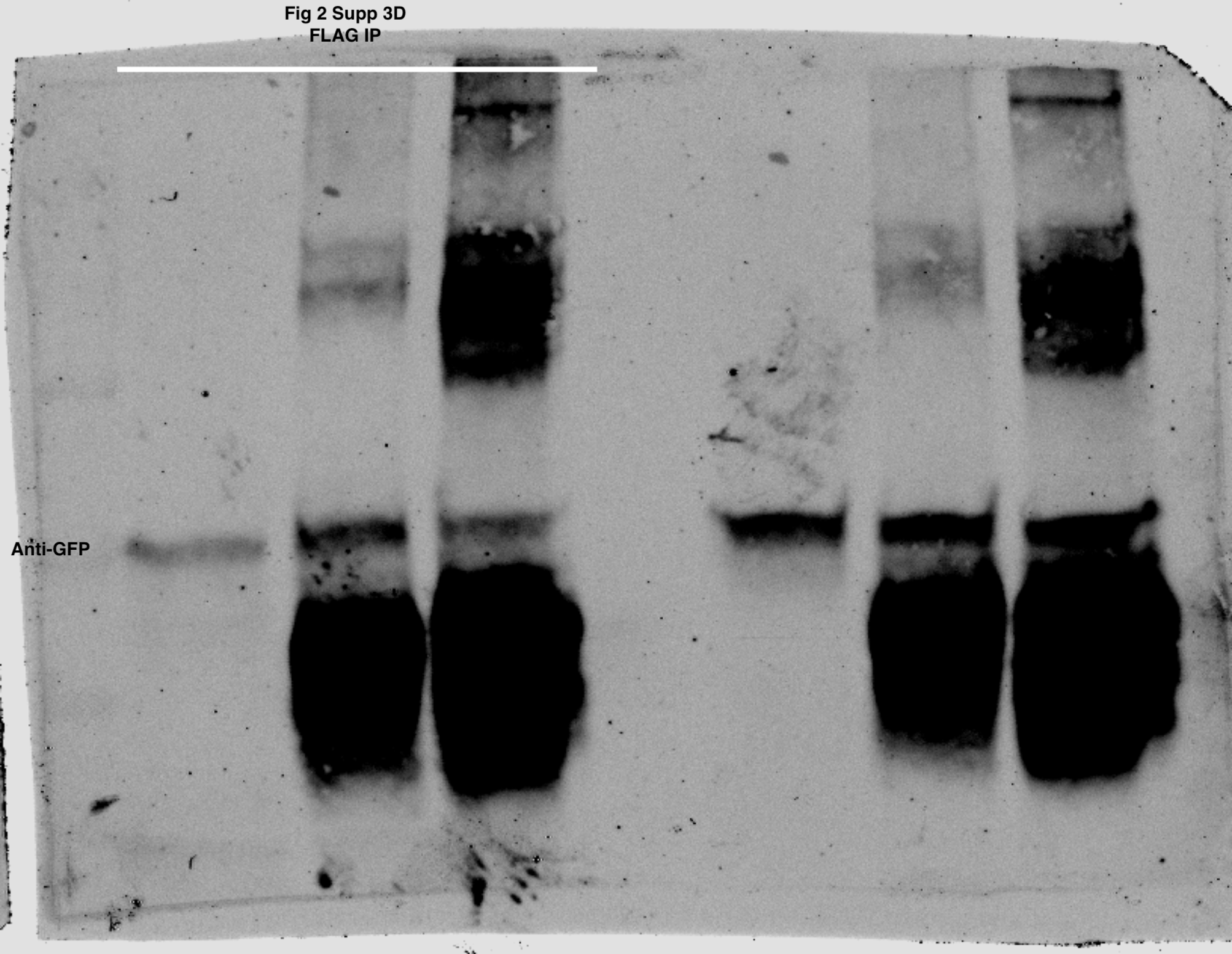

Supplement: Figure 2—figure supplement 3—source data 1. [file elife-82860-fig2-figsupp3-data1.zip › elife_Fig 2 Supp 3 source data/elife_Fig 2 Supp 3 source data 2/Fig_2_Supp_3D_Source_Data_Labeled/Fig_2_Supp_3D_GFP blot_labeled.tif]

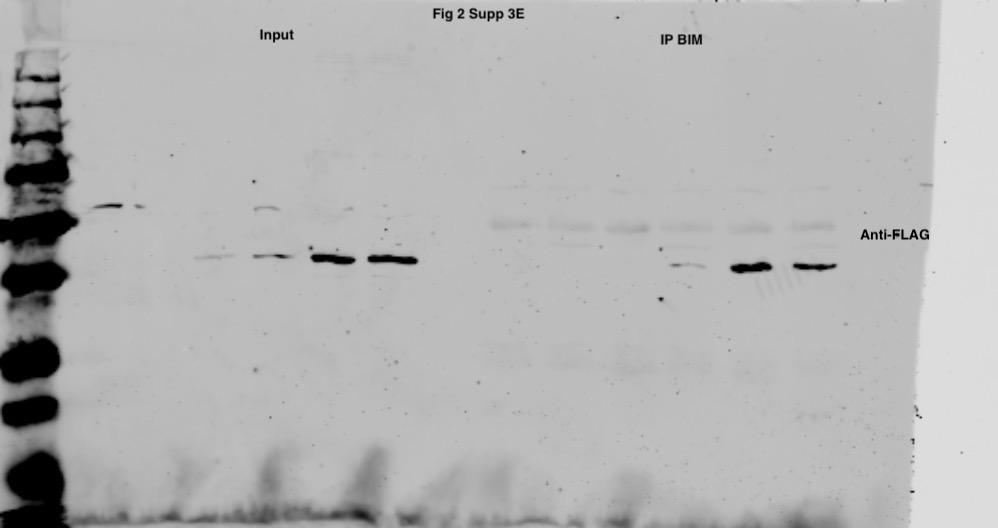

Supplement: Figure 2—figure supplement 3—source data 1. [file elife-82860-fig2-figsupp3-data1.zip › elife_Fig 2 Supp 3 source data/elife_Fig 2 Supp 3 source data 3/Fig_2_Supp_3_Source_Data 3_Labeled/Fig_2_Supp_3E FLAG 1_labeled.JPG]

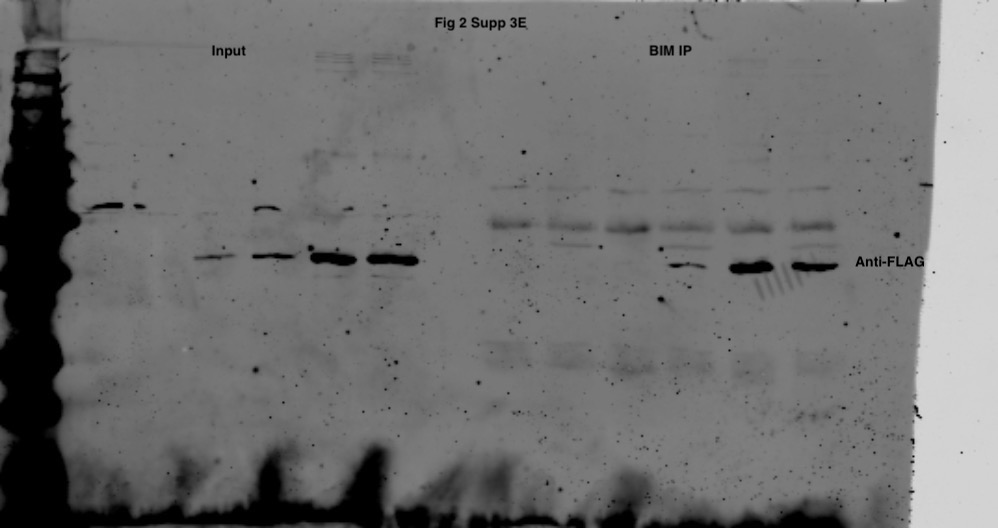

Supplement: Figure 2—figure supplement 3—source data 1. [file elife-82860-fig2-figsupp3-data1.zip › elife_Fig 2 Supp 3 source data/elife_Fig 2 Supp 3 source data 3/Fig_2_Supp_3_Source_Data 3_Labeled/Fig_2_Supp_3E FLAG2_labeled.JPG]

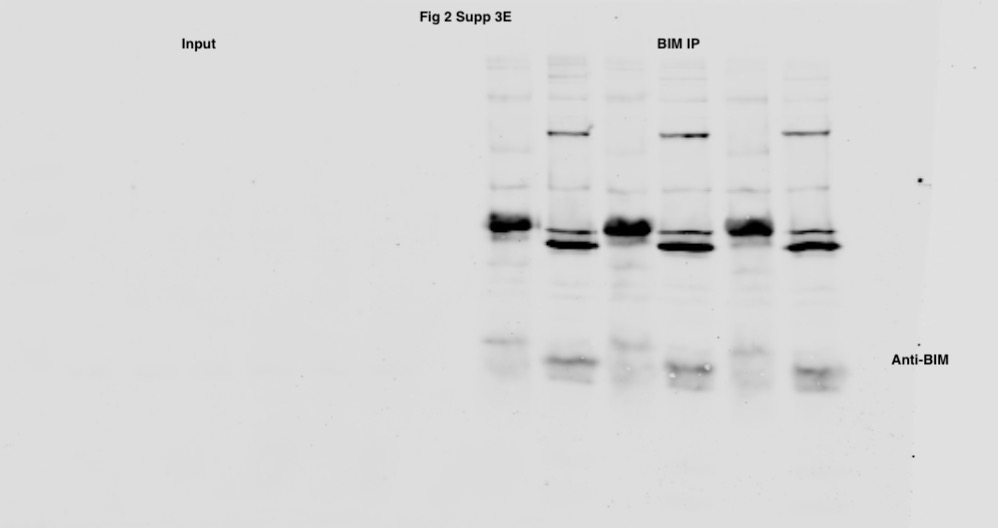

Supplement: Figure 2—figure supplement 3—source data 1. [file elife-82860-fig2-figsupp3-data1.zip › elife_Fig 2 Supp 3 source data/elife_Fig 2 Supp 3 source data 3/Fig_2_Supp_3_Source_Data 3_Labeled/Fig_2_Supp_3E_BIM_labeled.JPG]

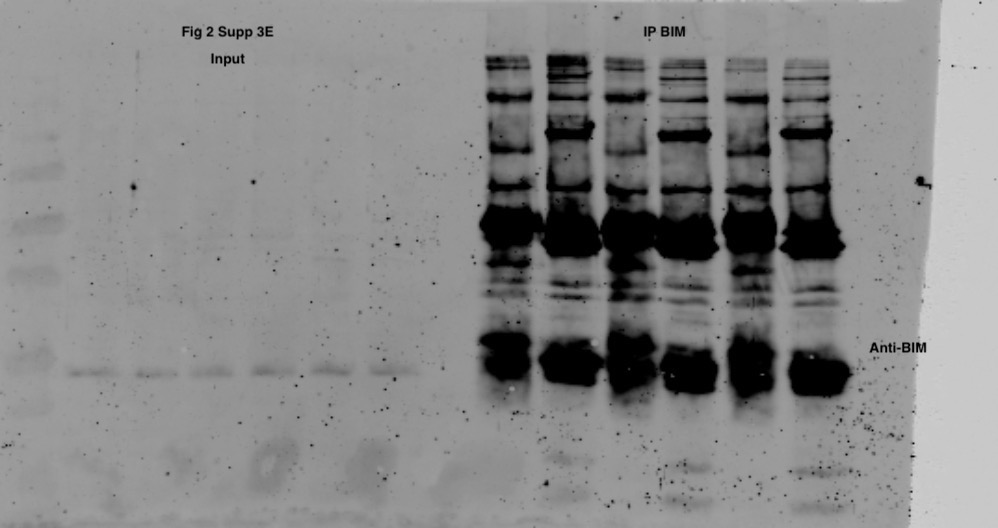

Supplement: Figure 2—figure supplement 3—source data 1. [file elife-82860-fig2-figsupp3-data1.zip › elife_Fig 2 Supp 3 source data/elife_Fig 2 Supp 3 source data 3/Fig_2_Supp_3_Source_Data 3_Labeled/Fig_2_Supp_3E BIM2_labeled.JPG]

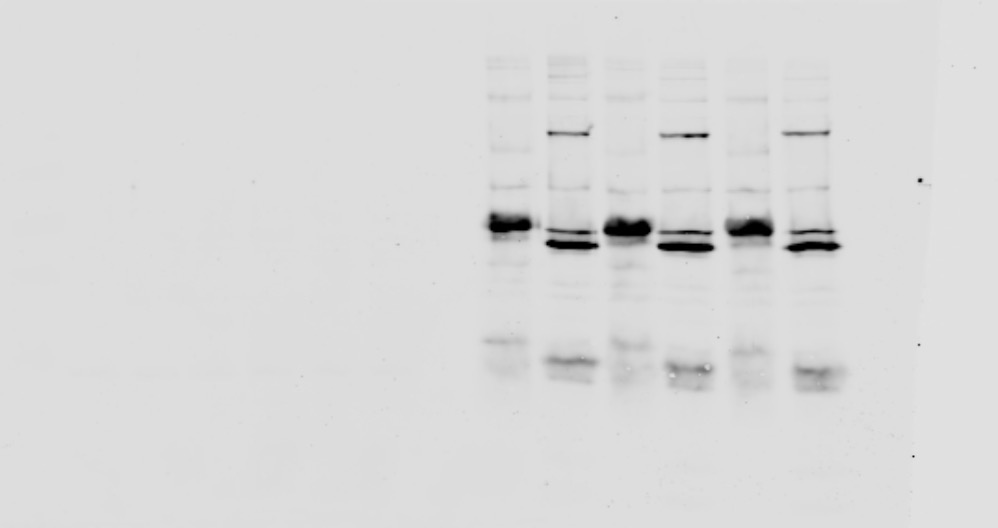

Supplement: Figure 2—figure supplement 3—source data 1. [file elife-82860-fig2-figsupp3-data1.zip › elife_Fig 2 Supp 3 source data/elife_Fig 2 Supp 3 source data 3/Fig_2_Supp_3_Source_Data 3_Unlabeled/Fig_2_Supp_3E_BIM_Unlabeled.JPG]
