# Supplementary figures and images for: Collateral deletion of the mitochondrial AAA+ ATPase ATAD1 sensitizes cancer cells to proteasome dysfunction (part 2 of 2)
Source: eLife. 2022 Nov 21;11:e82860. doi: 10.7554/eLife.82860 (PMC9815822; doi:10.7554/eLife.82860)

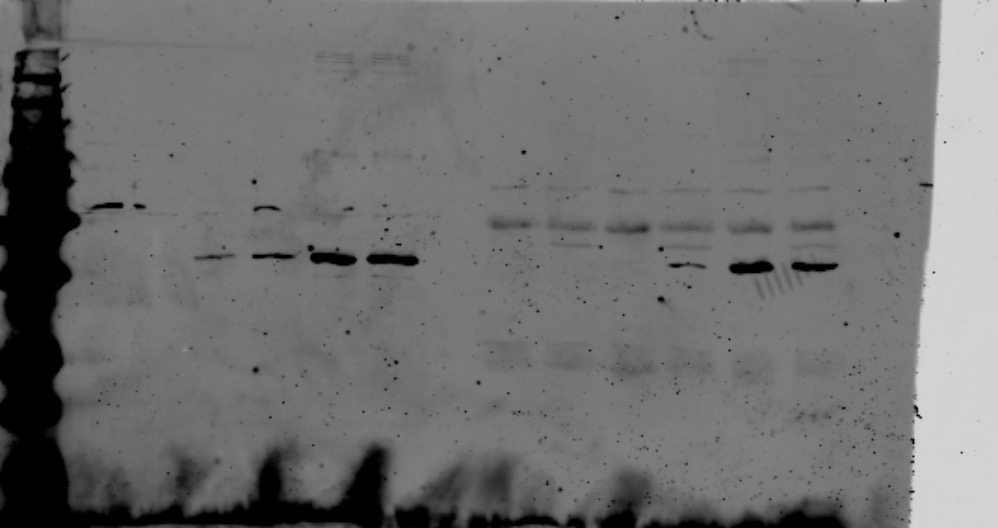

Supplement: Figure 2—figure supplement 3—source data 1. [file elife-82860-fig2-figsupp3-data1.zip › elife_Fig 2 Supp 3 source data/elife_Fig 2 Supp 3 source data 3/Fig_2_Supp_3_Source_Data 3_Unlabeled/Fig_2_Supp_3E FLAG2_Unlabeled.JPG]

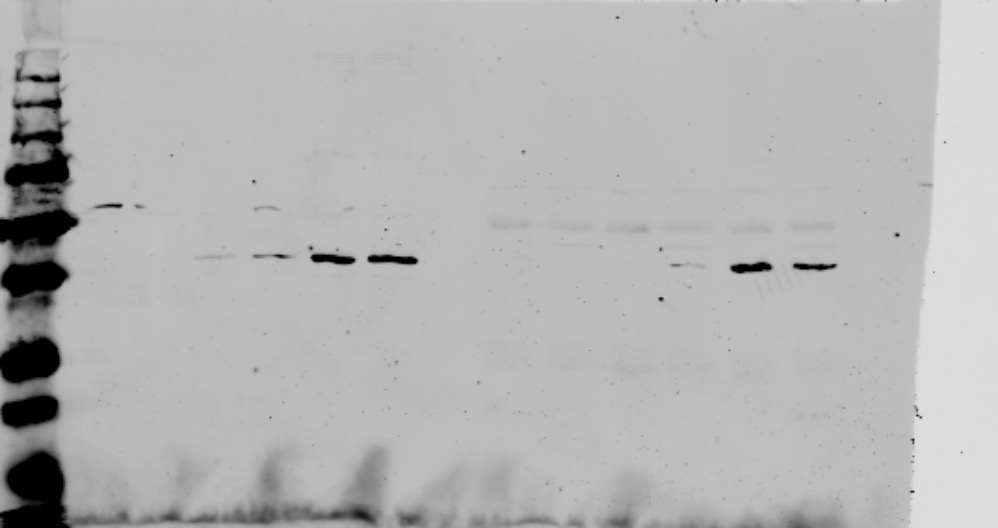

Supplement: Figure 2—figure supplement 3—source data 1. [file elife-82860-fig2-figsupp3-data1.zip › elife_Fig 2 Supp 3 source data/elife_Fig 2 Supp 3 source data 3/Fig_2_Supp_3_Source_Data 3_Unlabeled/Fig_2_Supp_3E FLAG 1_Unlabeled.JPG]

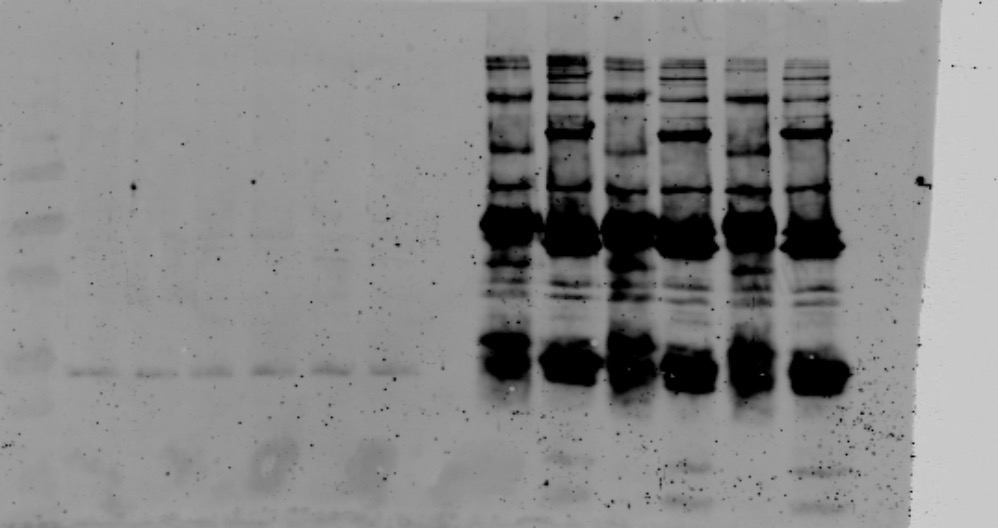

Supplement: Figure 2—figure supplement 3—source data 1. [file elife-82860-fig2-figsupp3-data1.zip › elife_Fig 2 Supp 3 source data/elife_Fig 2 Supp 3 source data 3/Fig_2_Supp_3_Source_Data 3_Unlabeled/Fig_2_Supp_3E BIM2_Unlabeled.JPG]

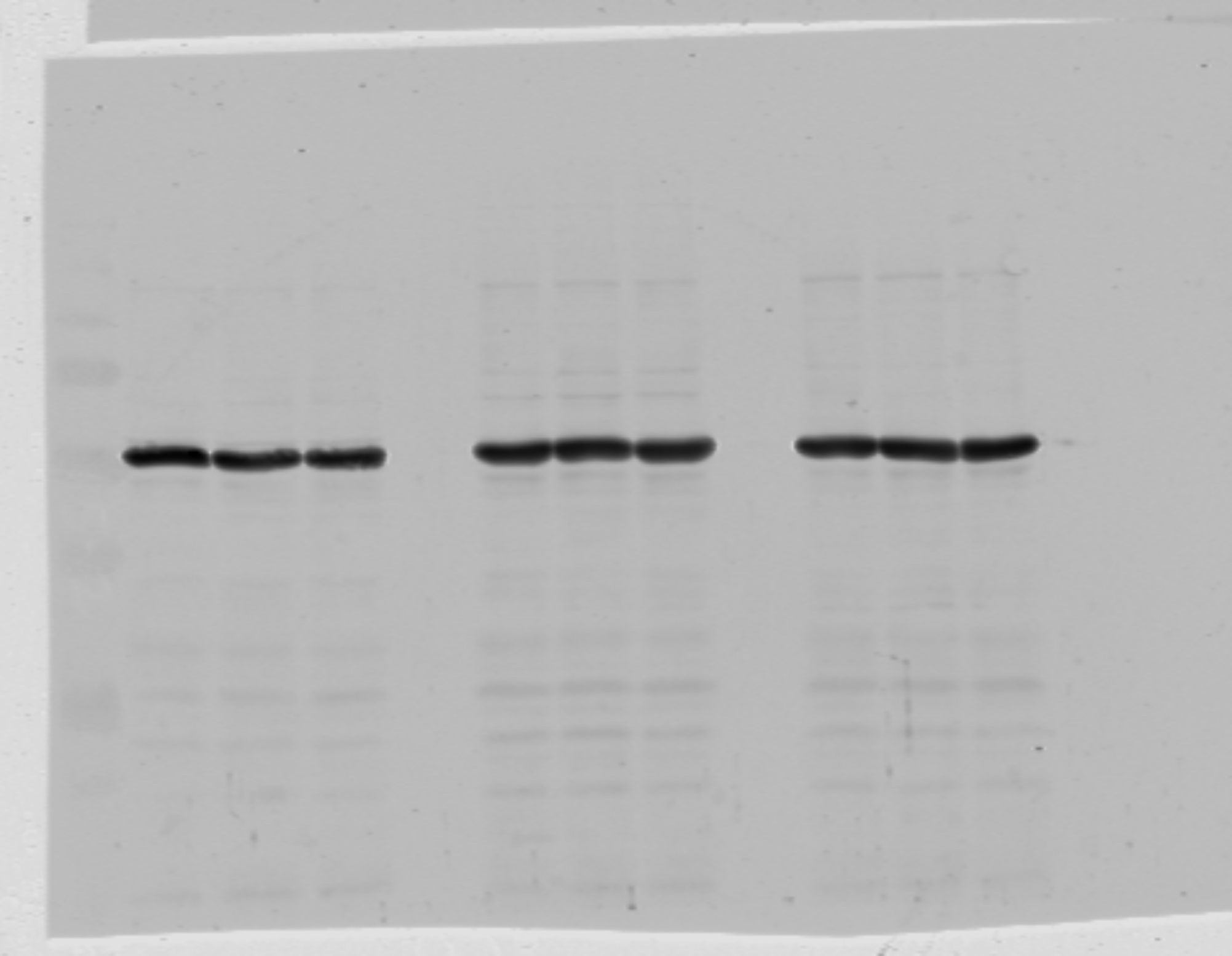

Supplement: Figure 2—figure supplement 3—source data 1. [file elife-82860-fig2-figsupp3-data1.zip › elife_Fig 2 Supp 3 source data/elife_Fig 2 Supp 3 source data 1/Fig_2_Supp_3B_Source_Data_Unlabeled/Fig_2_Supp_3B_Tubulin_Unlabeled.tif]

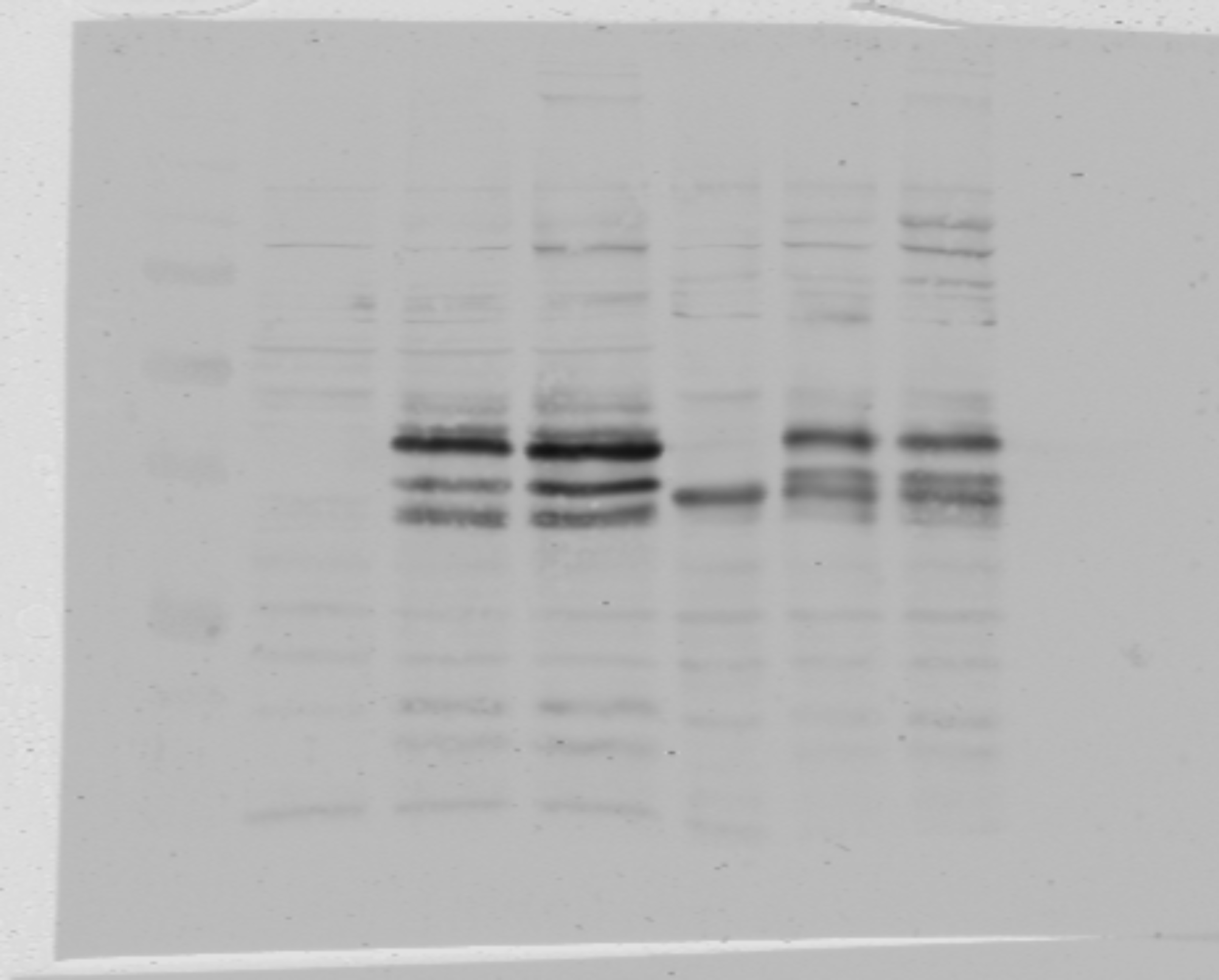

Supplement: Figure 2—figure supplement 3—source data 1. [file elife-82860-fig2-figsupp3-data1.zip › elife_Fig 2 Supp 3 source data/elife_Fig 2 Supp 3 source data 1/Fig_2_Supp_3B_Source_Data_Unlabeled/Fig_2_Supp_3B_ATAD1_Unlabeled.tif]

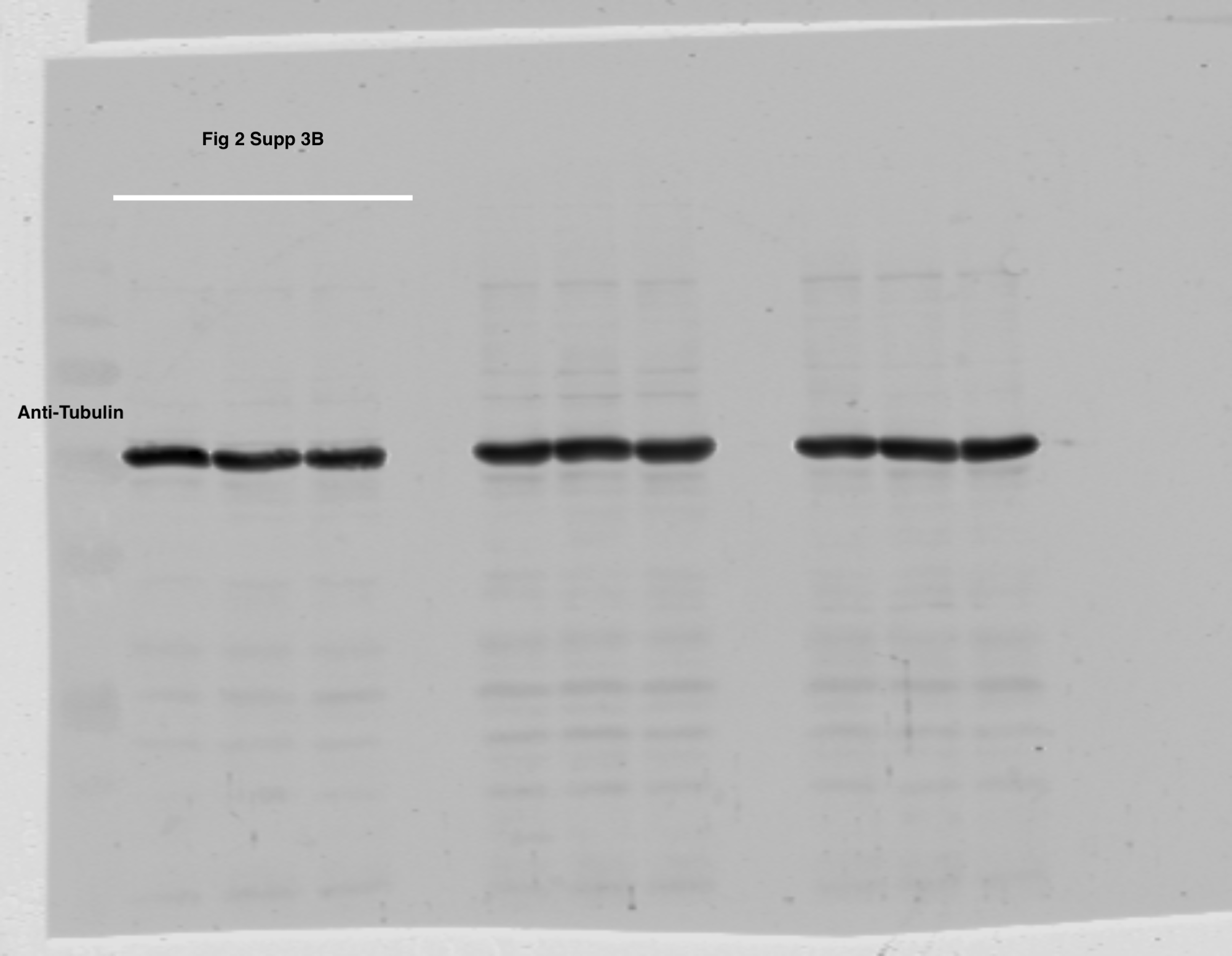

Supplement: Figure 2—figure supplement 3—source data 1. [file elife-82860-fig2-figsupp3-data1.zip › elife_Fig 2 Supp 3 source data/elife_Fig 2 Supp 3 source data 1/Fig_2_Supp_3B_Source_Data_Labeled/Fig_2_Supp_3B_Tubulin_labeled.tif]

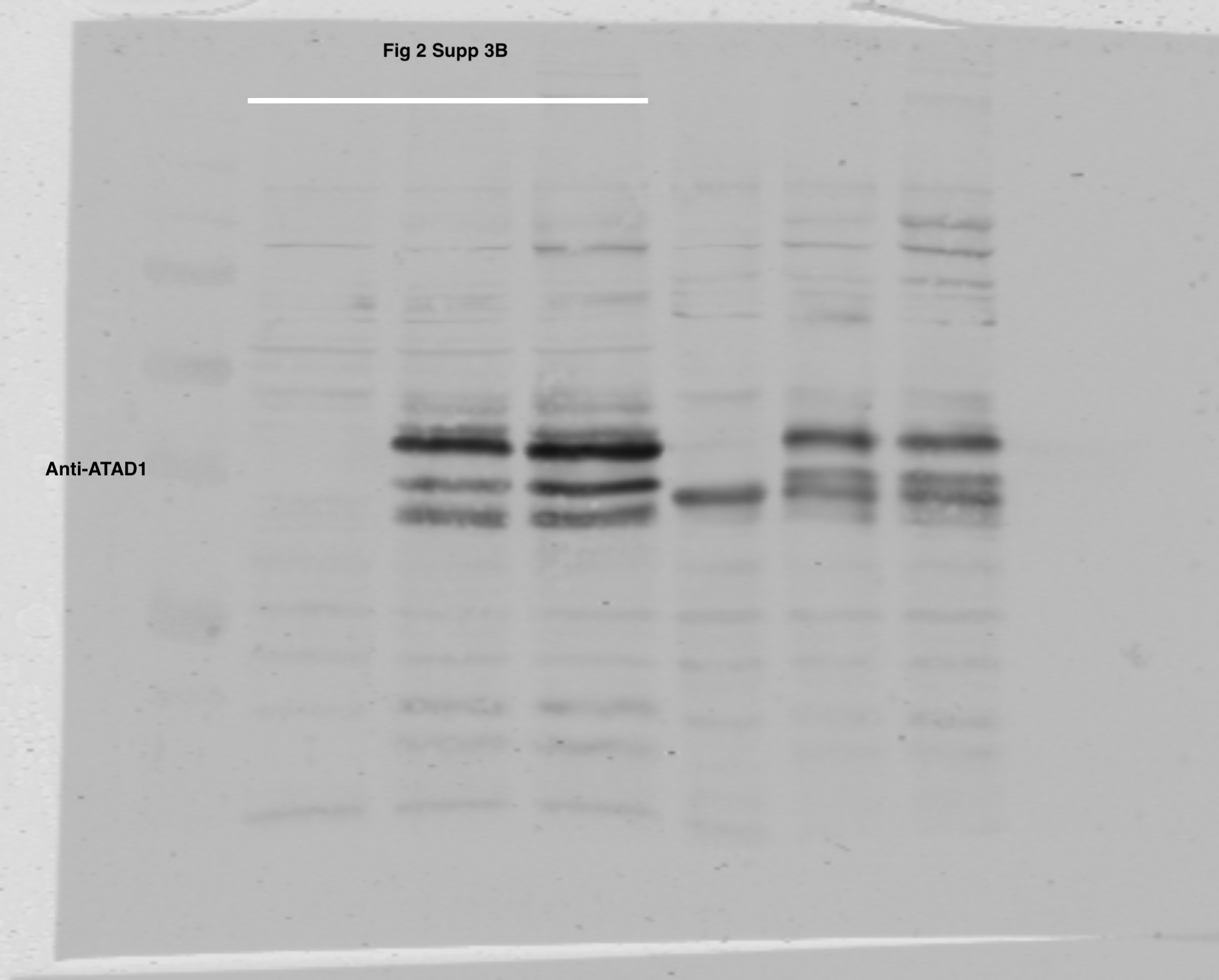

Supplement: Figure 2—figure supplement 3—source data 1. [file elife-82860-fig2-figsupp3-data1.zip › elife_Fig 2 Supp 3 source data/elife_Fig 2 Supp 3 source data 1/Fig_2_Supp_3B_Source_Data_Labeled/Fig_2_Supp_3B_ATAD1_labeled.tif]

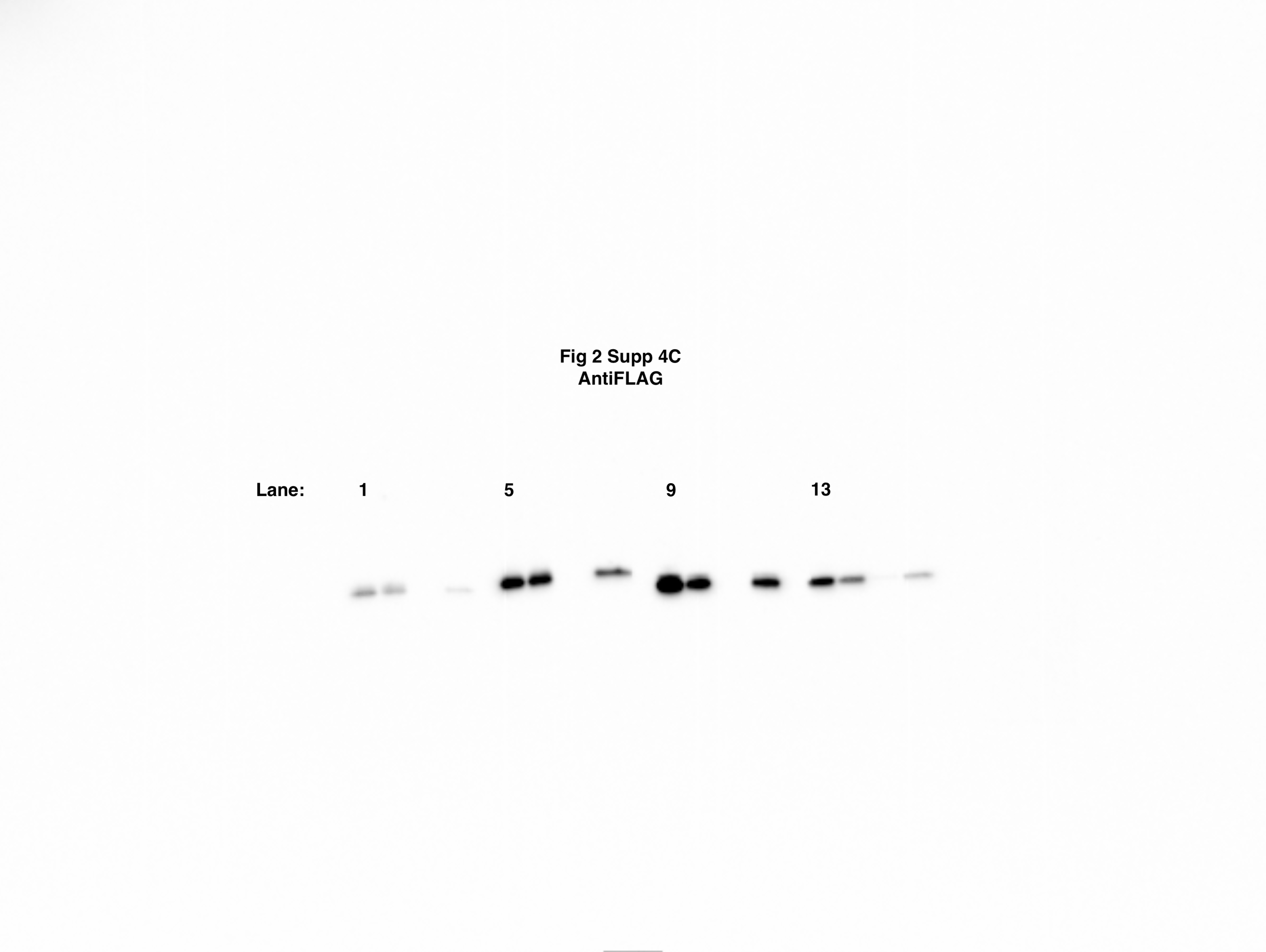

Supplement: Figure 2—figure supplement 4—source data 1. [file elife-82860-fig2-figsupp4-data1.zip › elife_Fig 2 Supp 4 source data/elife_Fig 2 Supp 4 source data 3/Fig_2_Supp_4C_Source_Data_Labeled/Fig_2_Supp_4C_AntiFLAG_labeled.tif]

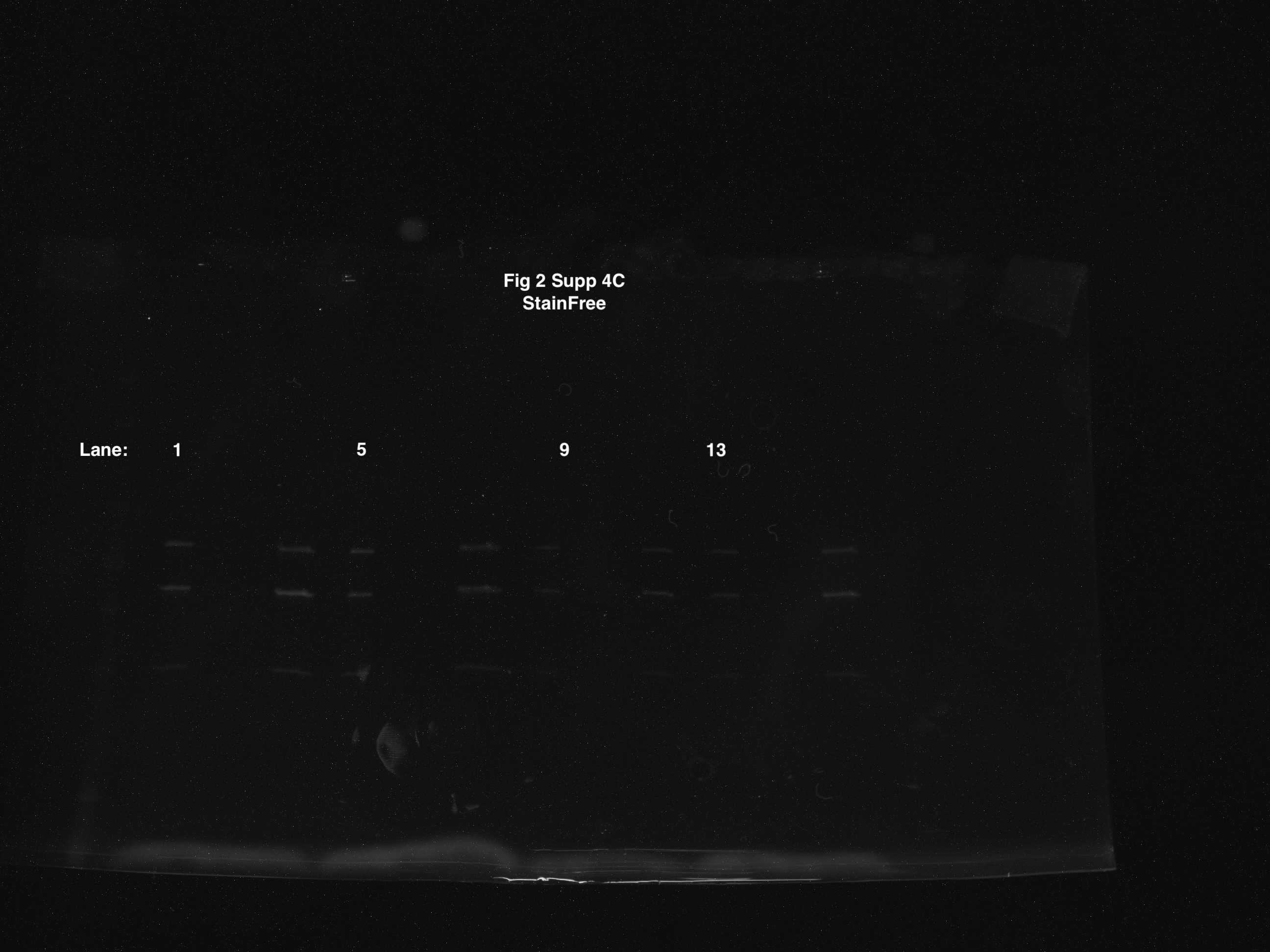

Supplement: Figure 2—figure supplement 4—source data 1. [file elife-82860-fig2-figsupp4-data1.zip › elife_Fig 2 Supp 4 source data/elife_Fig 2 Supp 4 source data 3/Fig_2_Supp_4C_Source_Data_Labeled/Fig_2_Supp_4C_StainFree_labled.jpg]

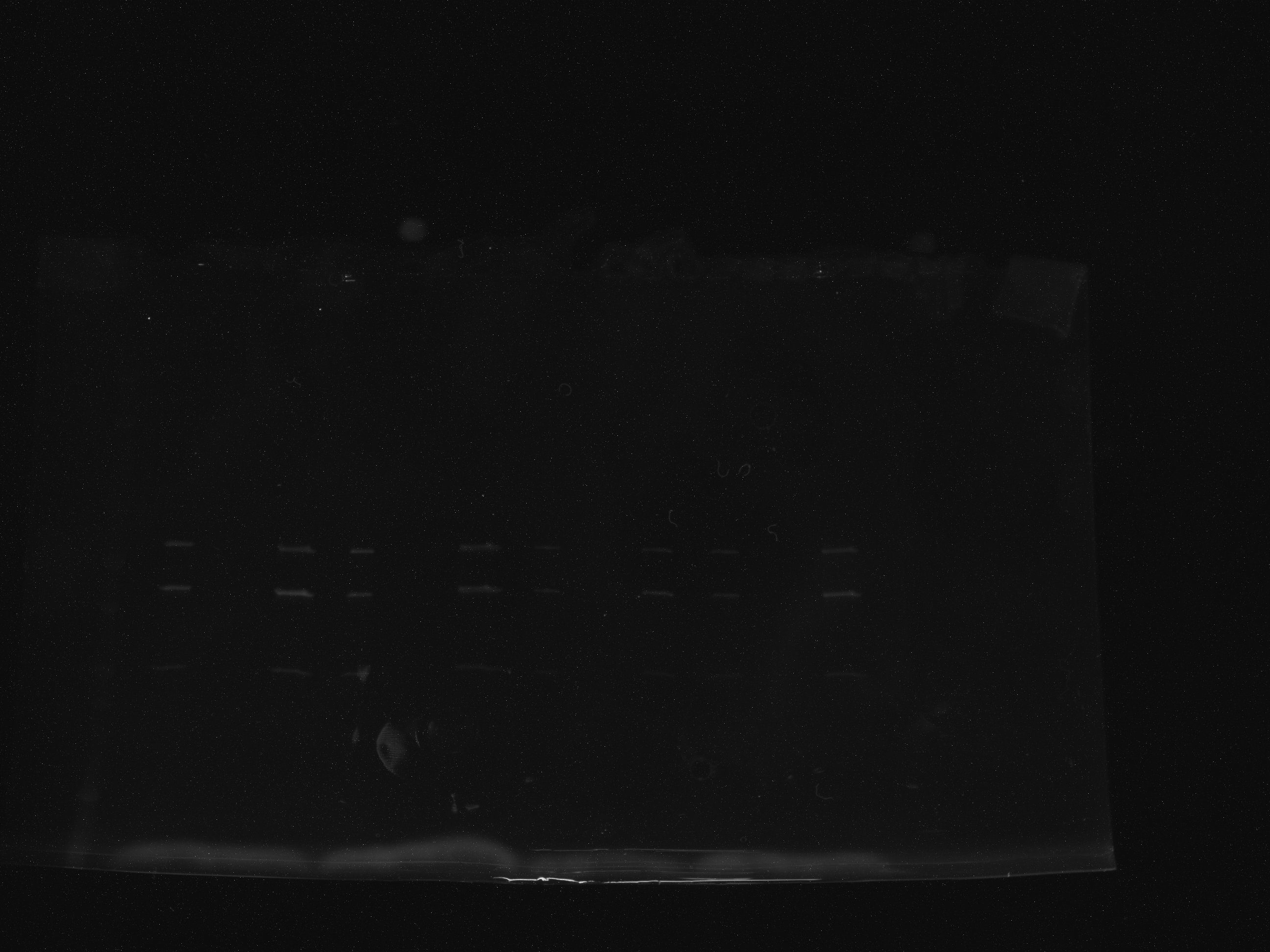

Supplement: Figure 2—figure supplement 4—source data 1. [file elife-82860-fig2-figsupp4-data1.zip › elife_Fig 2 Supp 4 source data/elife_Fig 2 Supp 4 source data 3/Fig_2_Supp_4C_Source_Data_Unlabeled/Fig_2_Supp_4C_StainFree_Unlabled.jpg]

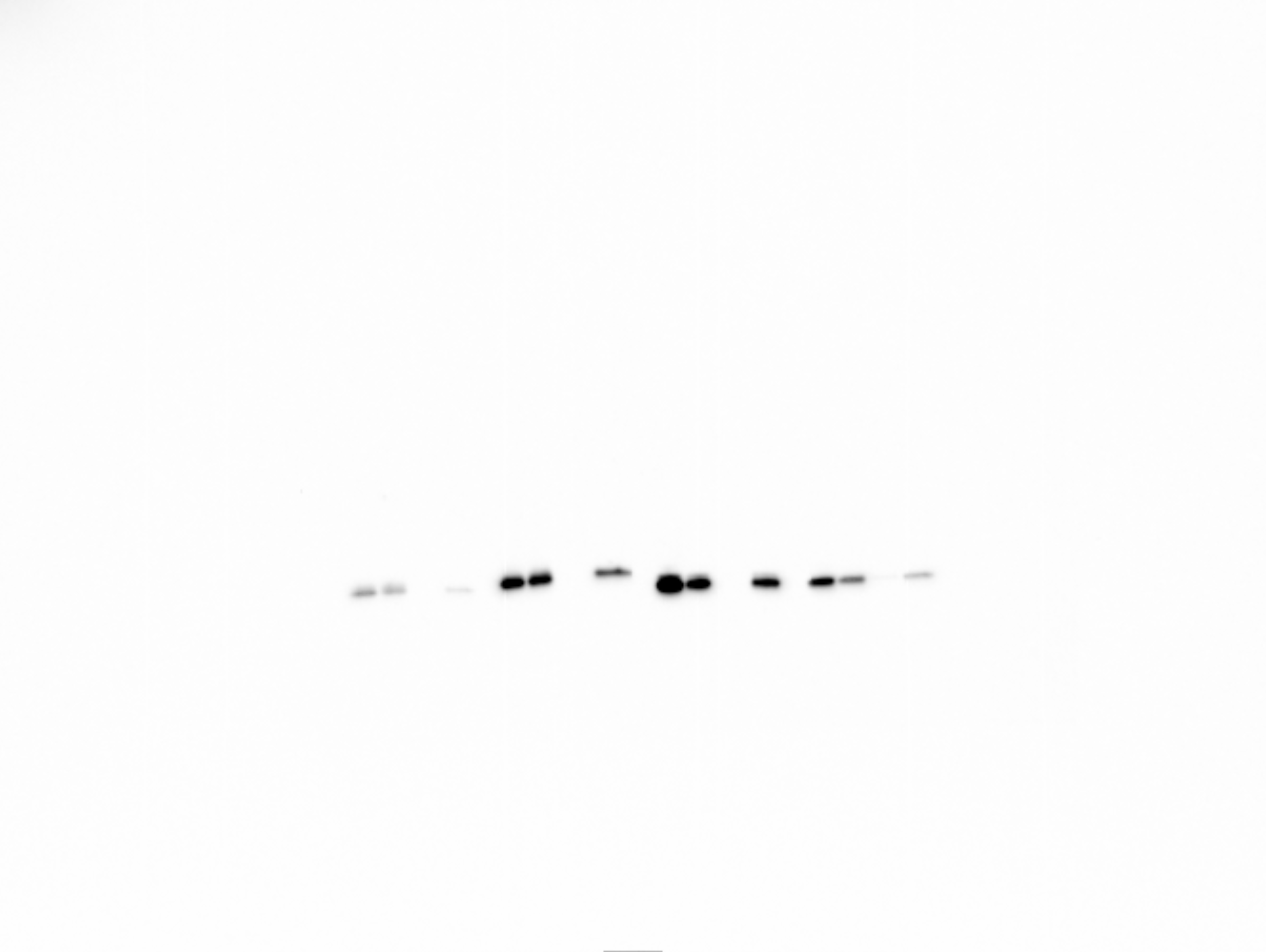

Supplement: Figure 2—figure supplement 4—source data 1. [file elife-82860-fig2-figsupp4-data1.zip › elife_Fig 2 Supp 4 source data/elife_Fig 2 Supp 4 source data 3/Fig_2_Supp_4C_Source_Data_Unlabeled/Fig_2_Supp_4C_AntiFLAG_Unlabeled.tif]

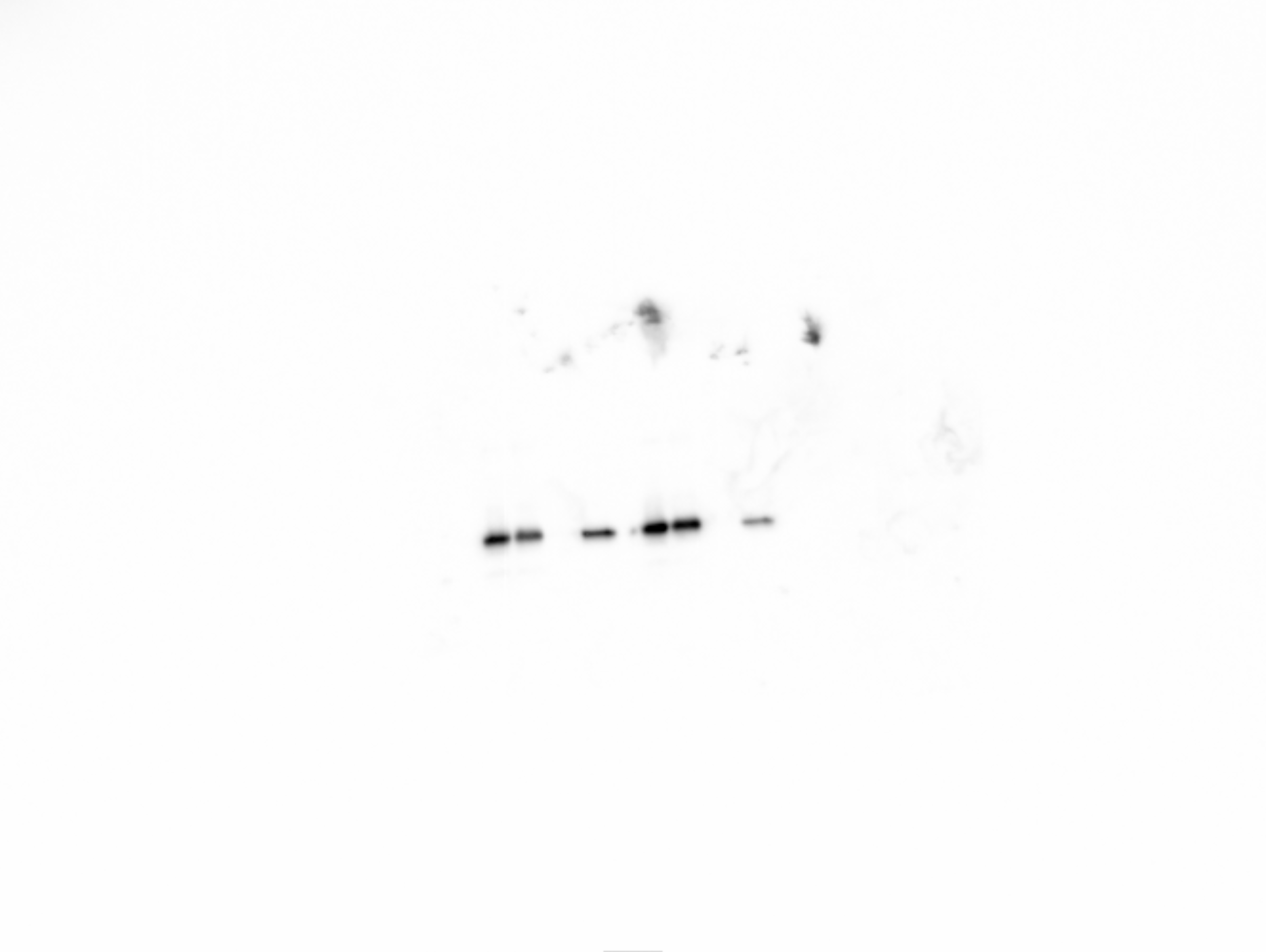

Supplement: Figure 2—figure supplement 4—source data 1. [file elife-82860-fig2-figsupp4-data1.zip › elife_Fig 2 Supp 4 source data/elife_Fig 2 Supp 4 source data 2/Fig_2_Supp_4B_Unlabeled/Fig_2_Supp_4B_AntiFlag_Unlabeled.tif]

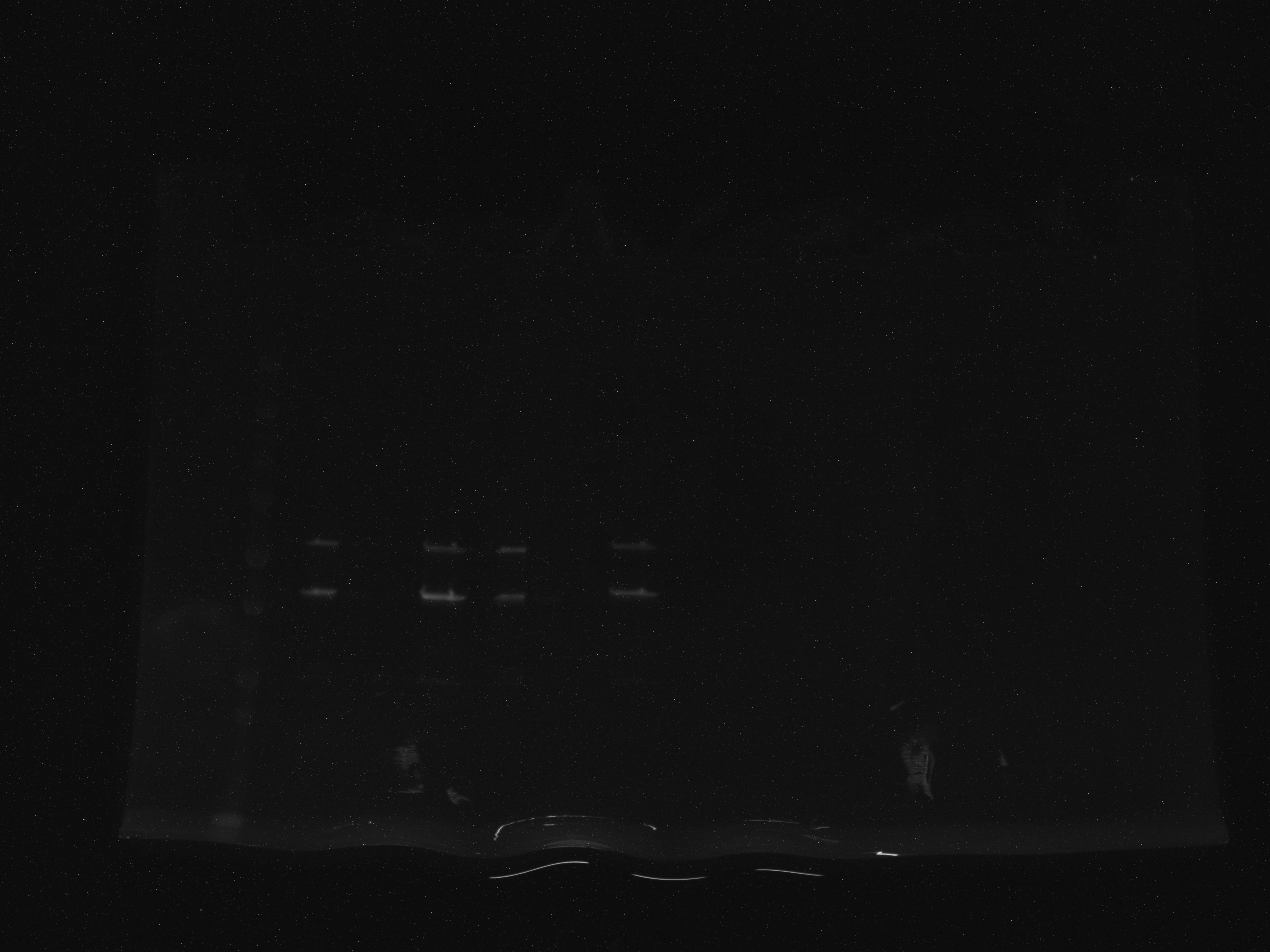

Supplement: Figure 2—figure supplement 4—source data 1. [file elife-82860-fig2-figsupp4-data1.zip › elife_Fig 2 Supp 4 source data/elife_Fig 2 Supp 4 source data 2/Fig_2_Supp_4B_Unlabeled/Fig_2_Supp_4B_StainFree_Unlabeled.jpg]

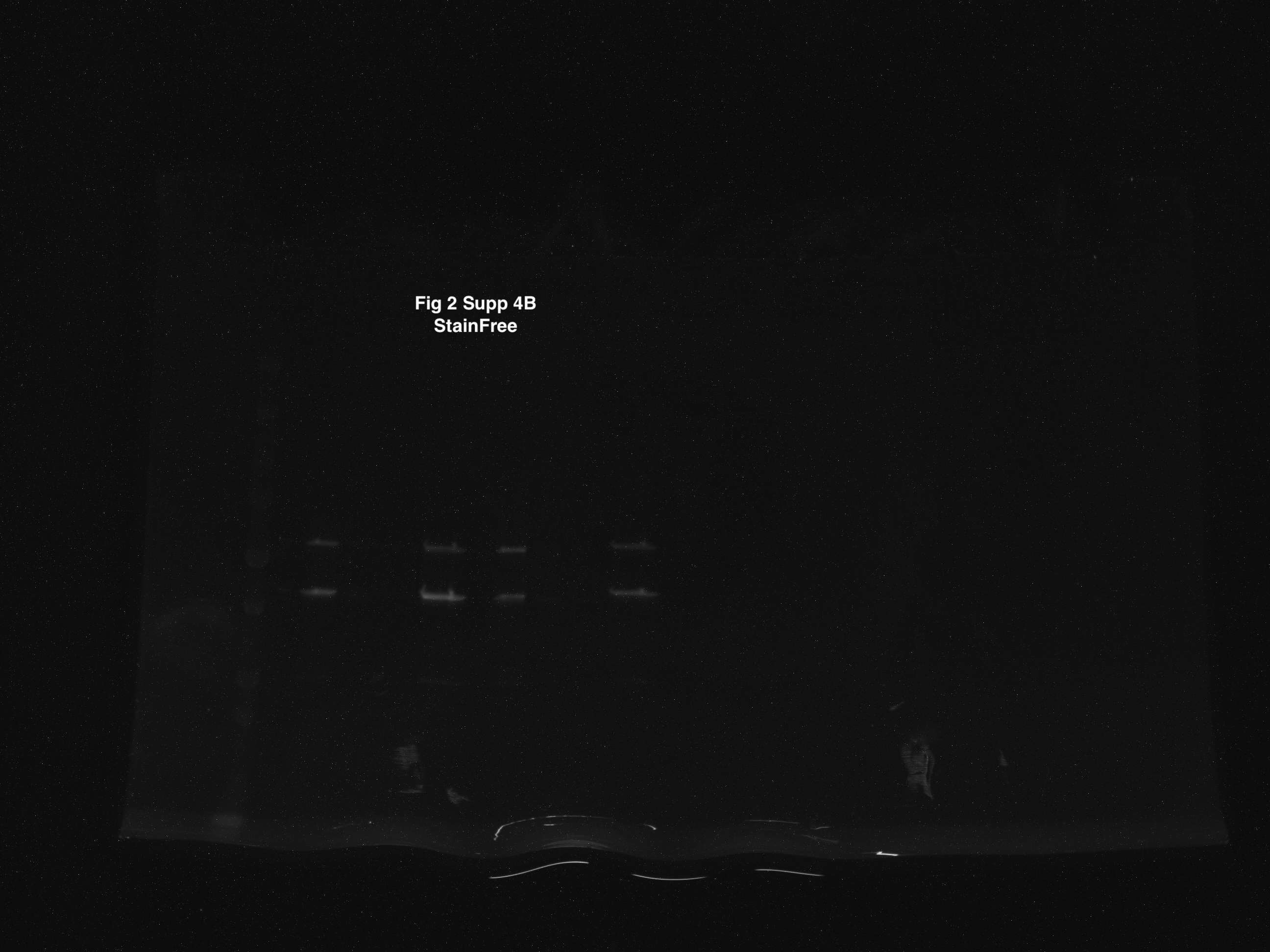

Supplement: Figure 2—figure supplement 4—source data 1. [file elife-82860-fig2-figsupp4-data1.zip › elife_Fig 2 Supp 4 source data/elife_Fig 2 Supp 4 source data 2/Fig_2_Supp_4B_Labeled/Fig_2_Supp_4B_StainFree_labeled.jpg]

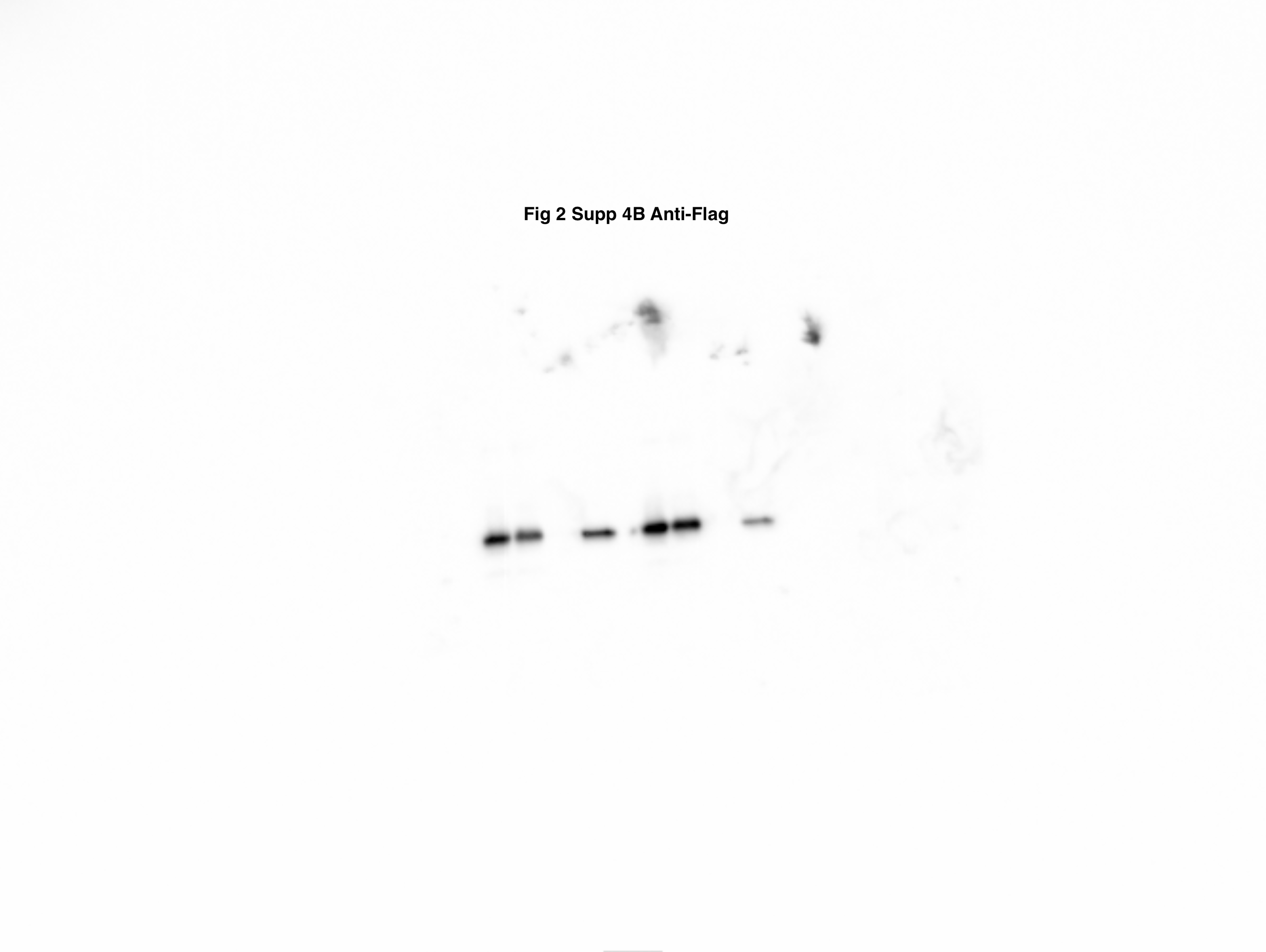

Supplement: Figure 2—figure supplement 4—source data 1. [file elife-82860-fig2-figsupp4-data1.zip › elife_Fig 2 Supp 4 source data/elife_Fig 2 Supp 4 source data 2/Fig_2_Supp_4B_Labeled/Fig_2_Supp_4B_AntiFlag_labeled.tif]

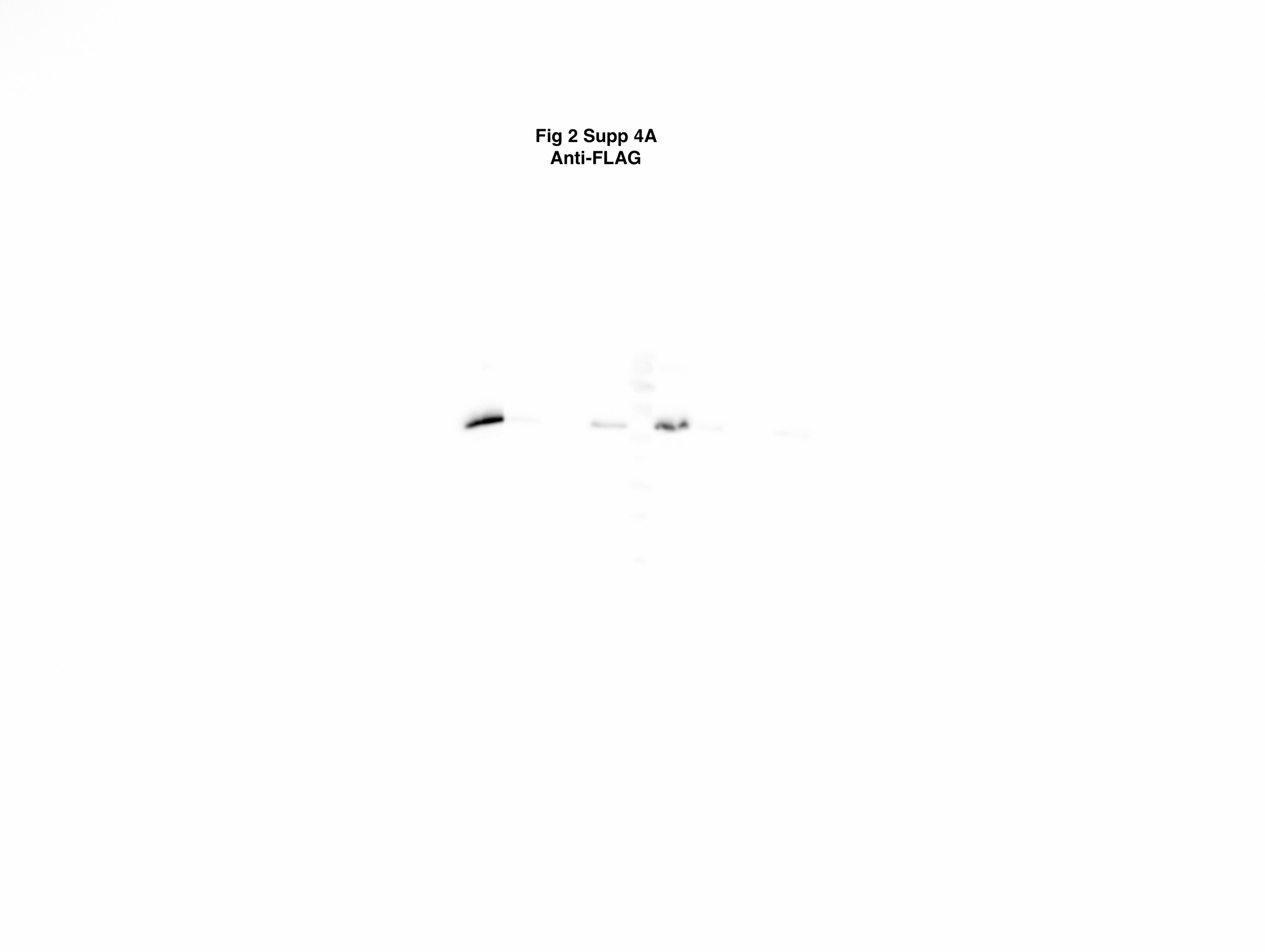

Supplement: Figure 2—figure supplement 4—source data 1. [file elife-82860-fig2-figsupp4-data1.zip › elife_Fig 2 Supp 4 source data/elife_Fig 2 Supp 4 source data 1/Fig_2_Supp_4A_Labeled/Fig_2_Supp_4A_AntiFLAG_labeled.tif]

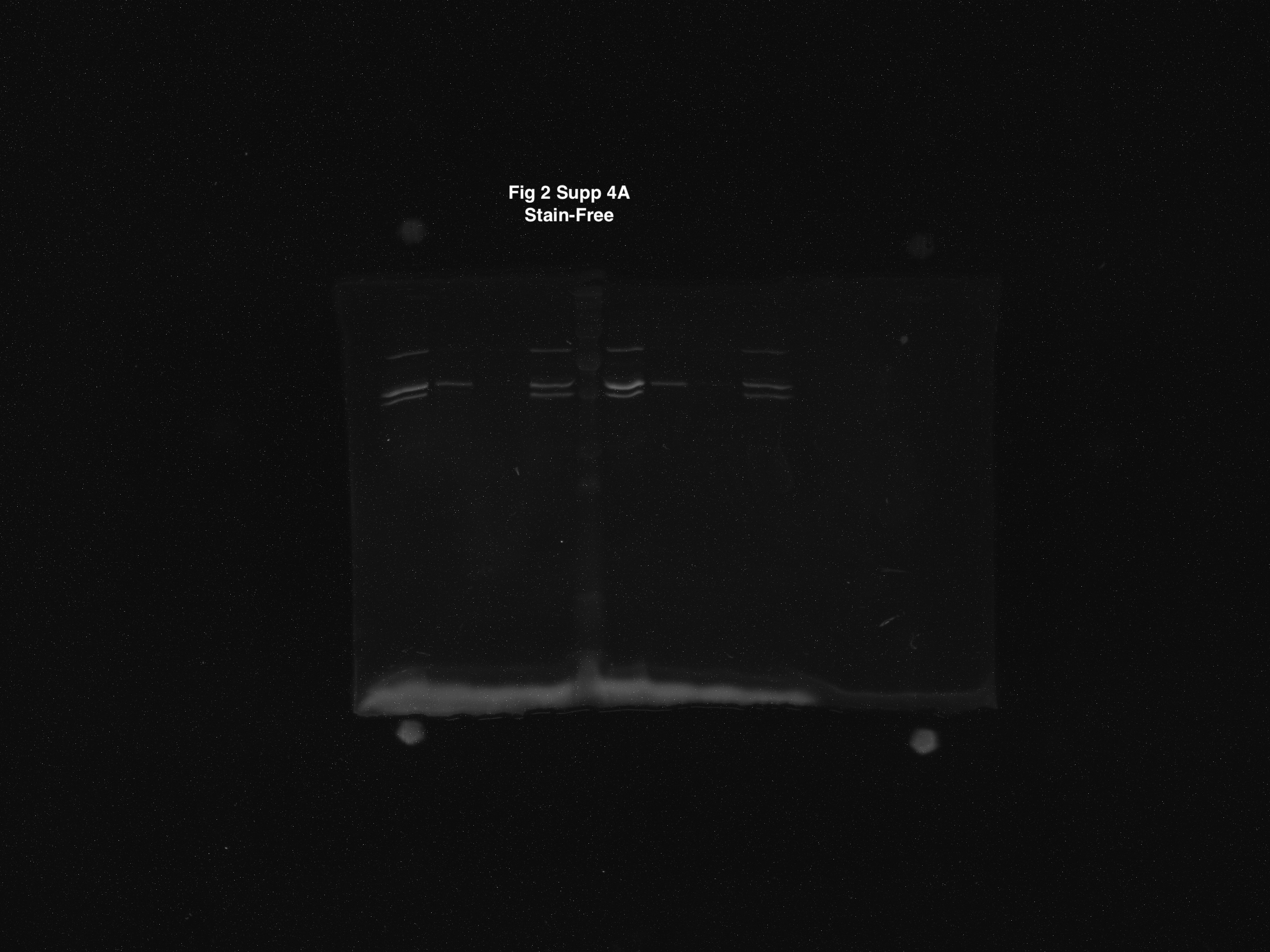

Supplement: Figure 2—figure supplement 4—source data 1. [file elife-82860-fig2-figsupp4-data1.zip › elife_Fig 2 Supp 4 source data/elife_Fig 2 Supp 4 source data 1/Fig_2_Supp_4A_Labeled/Fig_2_Supp_4A_StainFree_labeled.jpg]

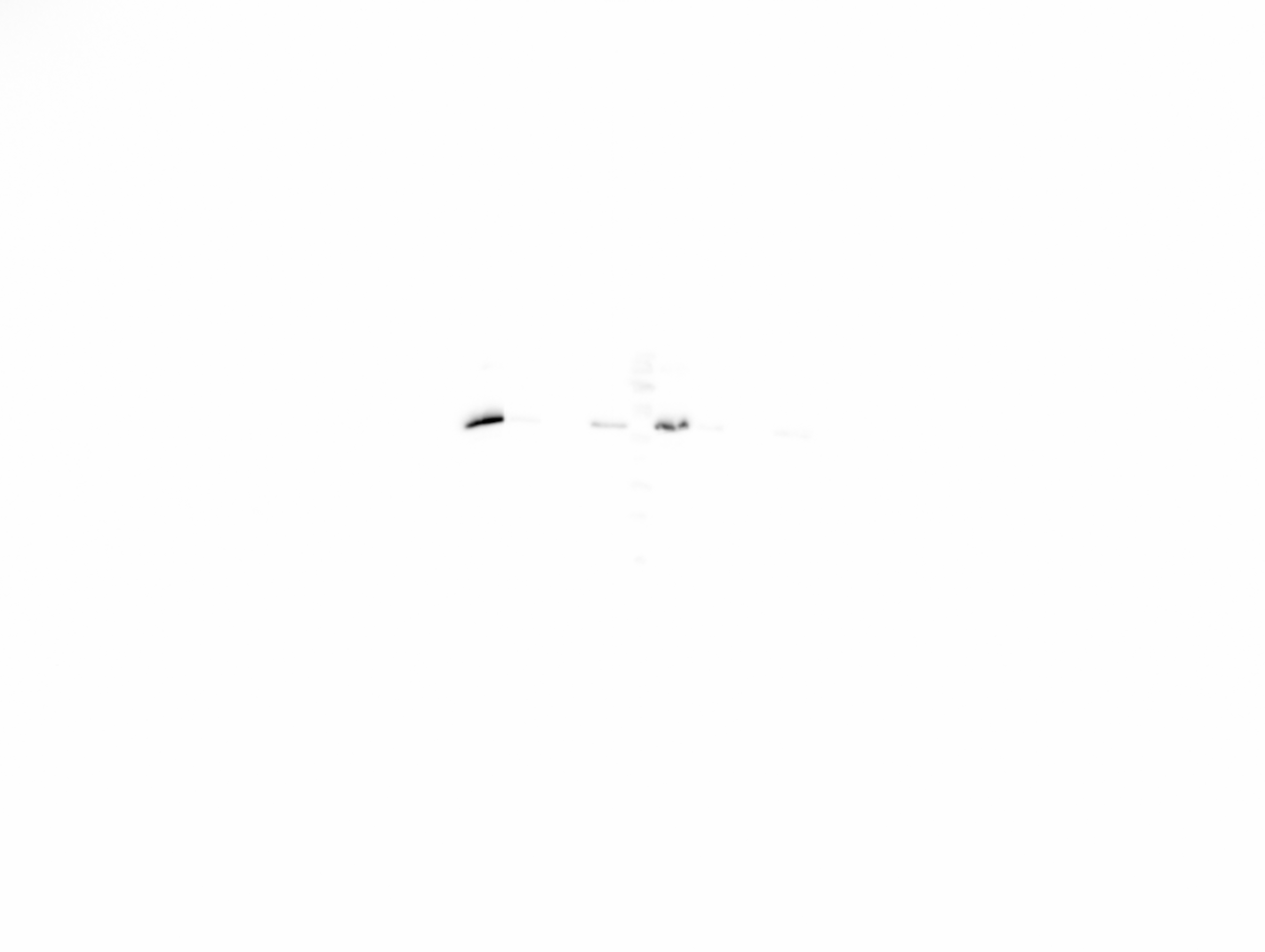

Supplement: Figure 2—figure supplement 4—source data 1. [file elife-82860-fig2-figsupp4-data1.zip › elife_Fig 2 Supp 4 source data/elife_Fig 2 Supp 4 source data 1/Fig_2_Supp_4A_Unlabeled/Fig_2_Supp_4A_AntiFLAG_Unlabeled.tif]

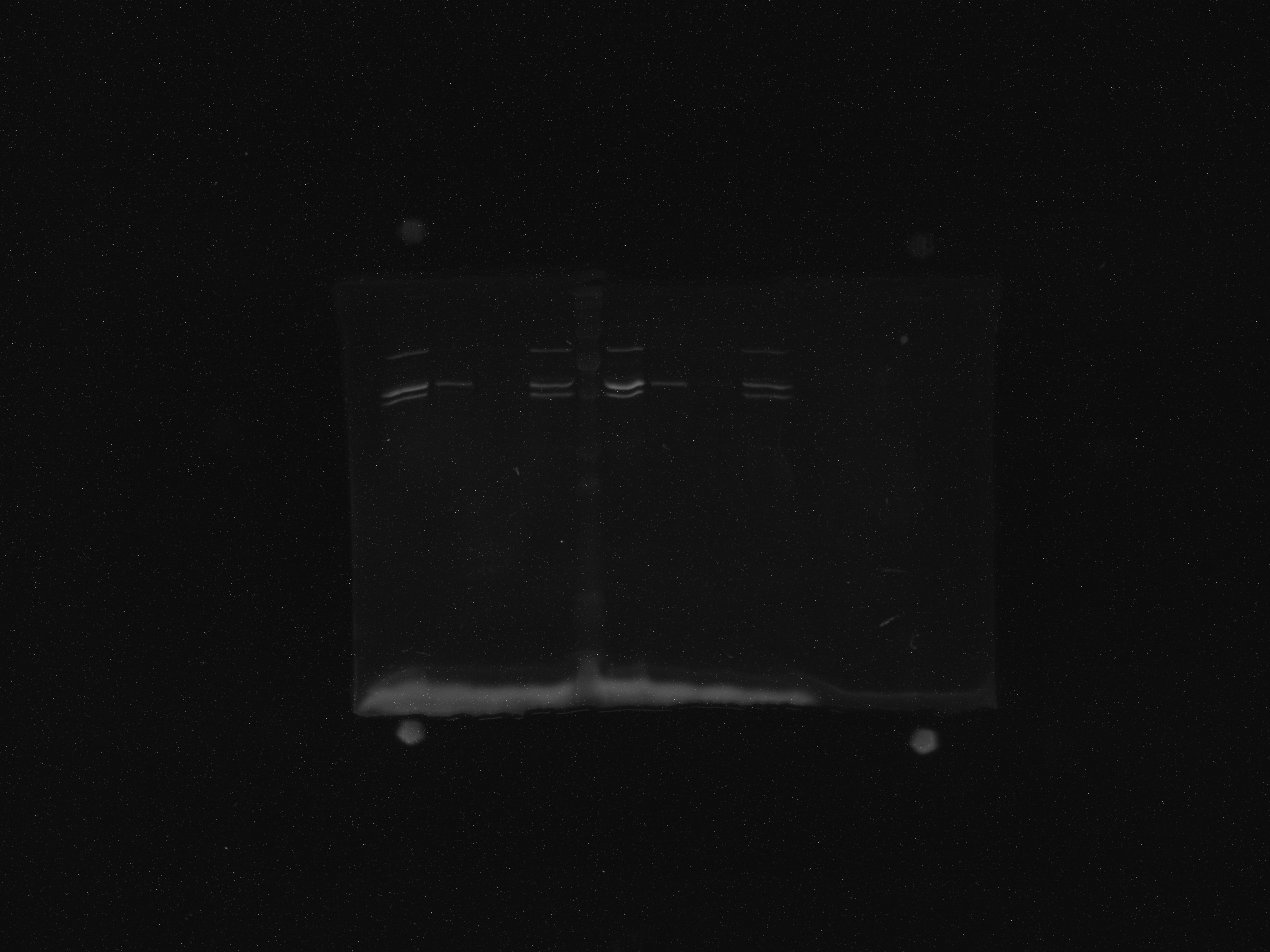

Supplement: Figure 2—figure supplement 4—source data 1. [file elife-82860-fig2-figsupp4-data1.zip › elife_Fig 2 Supp 4 source data/elife_Fig 2 Supp 4 source data 1/Fig_2_Supp_4A_Unlabeled/Fig_2_Supp_4A_StainFree_Unlabeled.jpg]

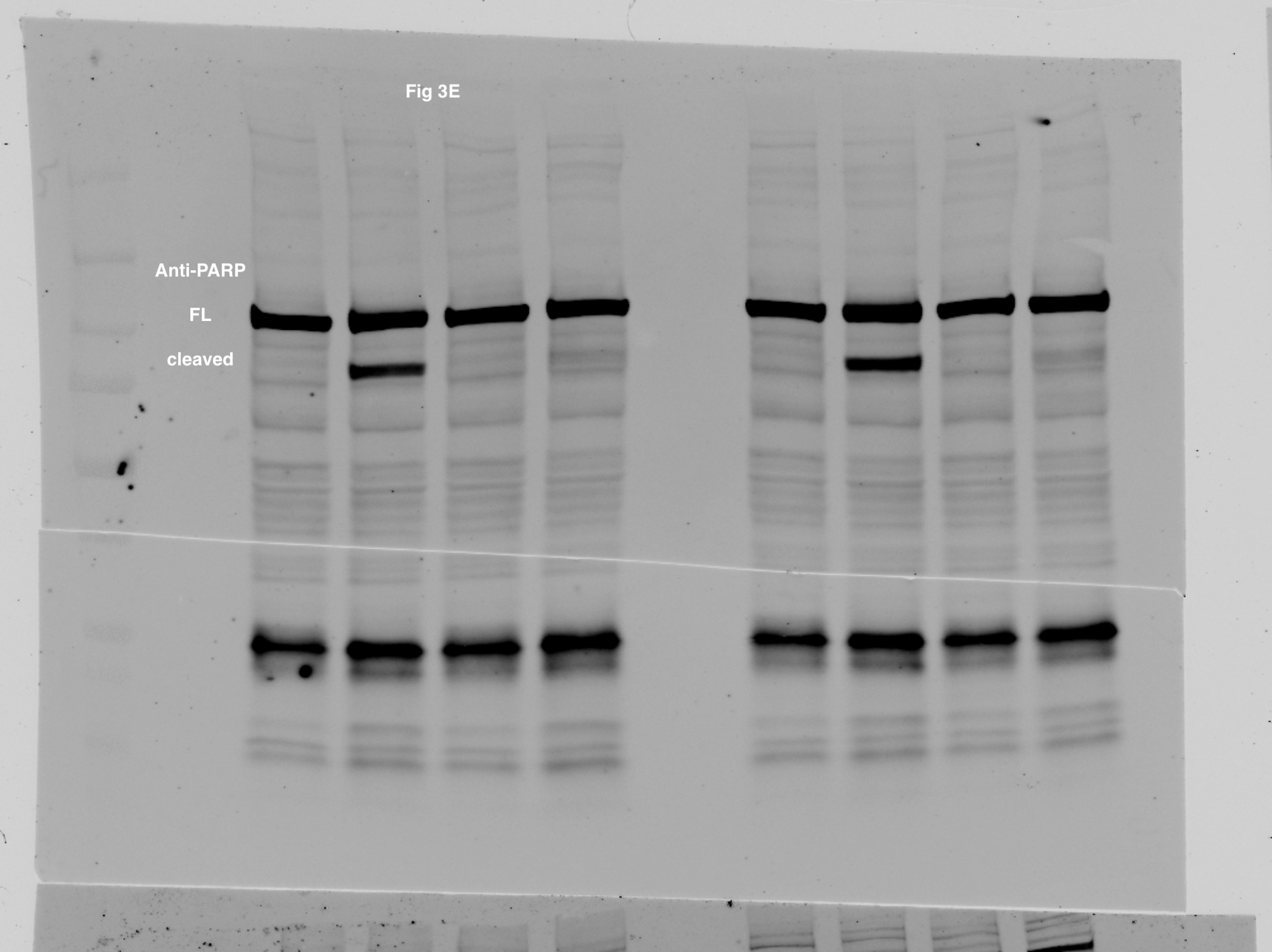

Supplement: Figure 3—source data 1. [file elife-82860-fig3-data1.zip › elife_Figure 3 source data/elife_Fig 3 source data 1/Fig_3E_Source_Data_Labeled/Fig_3E_PARP_labeled.tif]

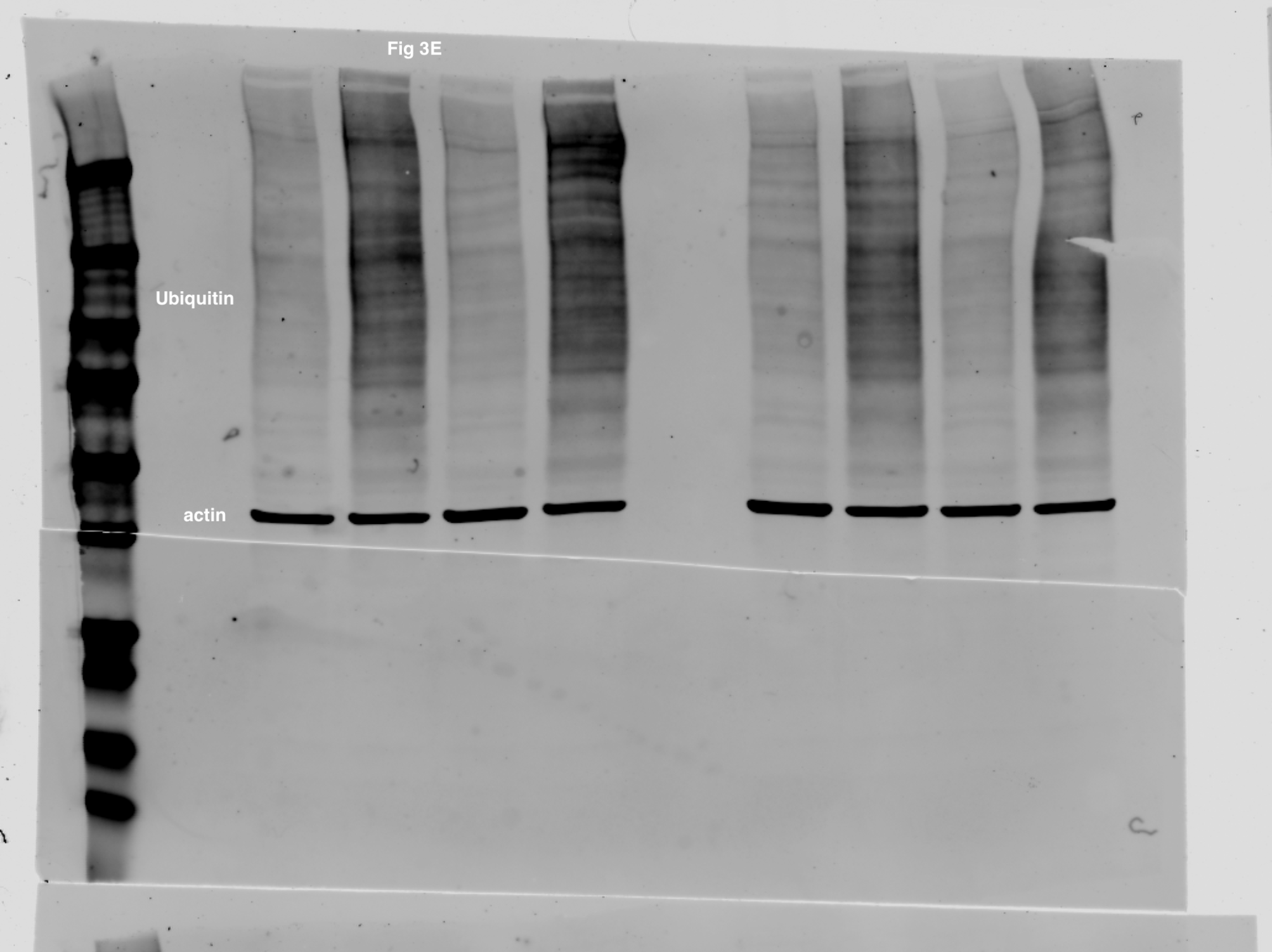

Supplement: Figure 3—source data 1. [file elife-82860-fig3-data1.zip › elife_Figure 3 source data/elife_Fig 3 source data 1/Fig_3E_Source_Data_Labeled/Fig_3E_ub_actin_labeled.tif]

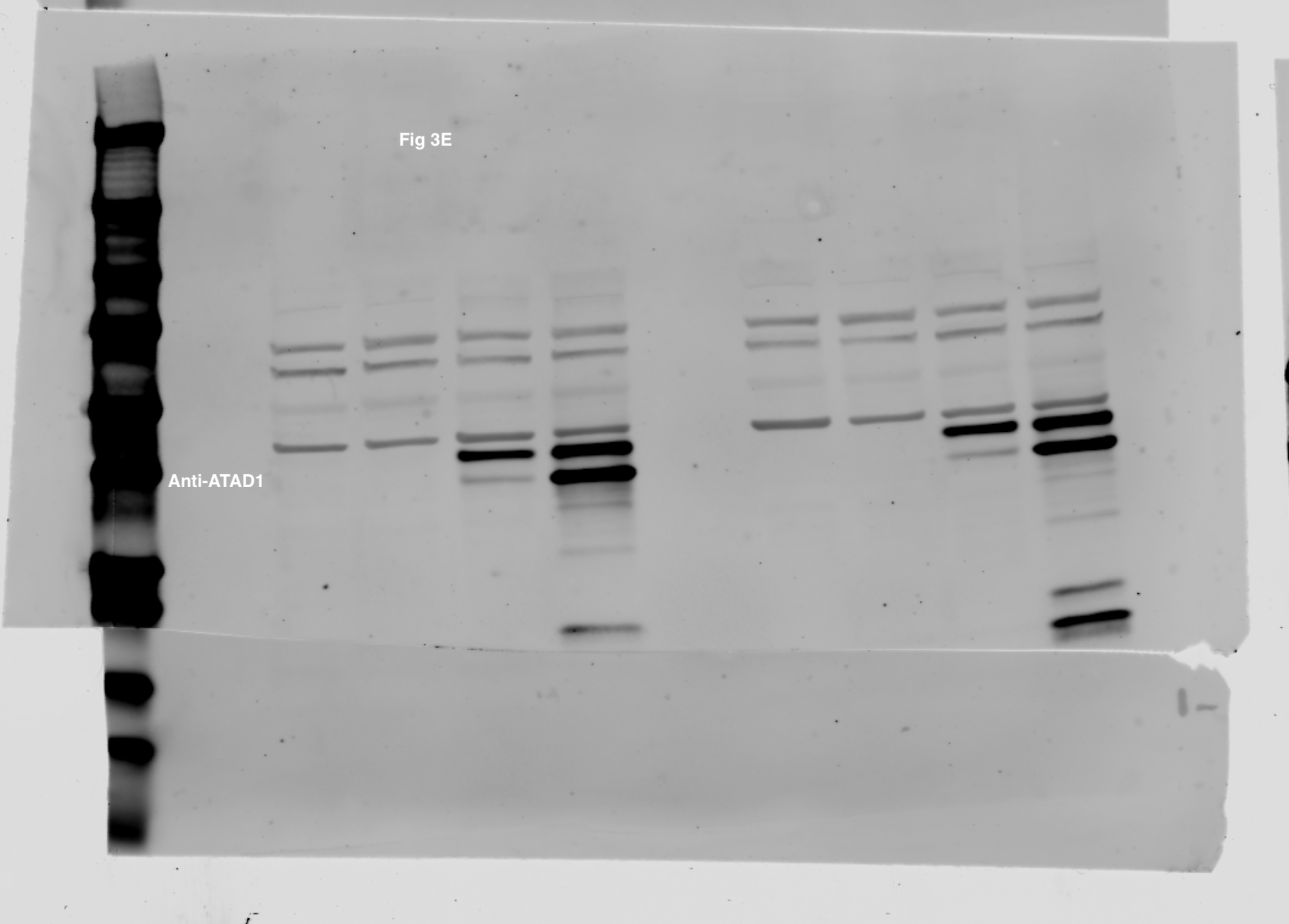

Supplement: Figure 3—source data 1. [file elife-82860-fig3-data1.zip › elife_Figure 3 source data/elife_Fig 3 source data 1/Fig_3E_Source_Data_Labeled/Fig_3E_ATAD1_labeled.tif]

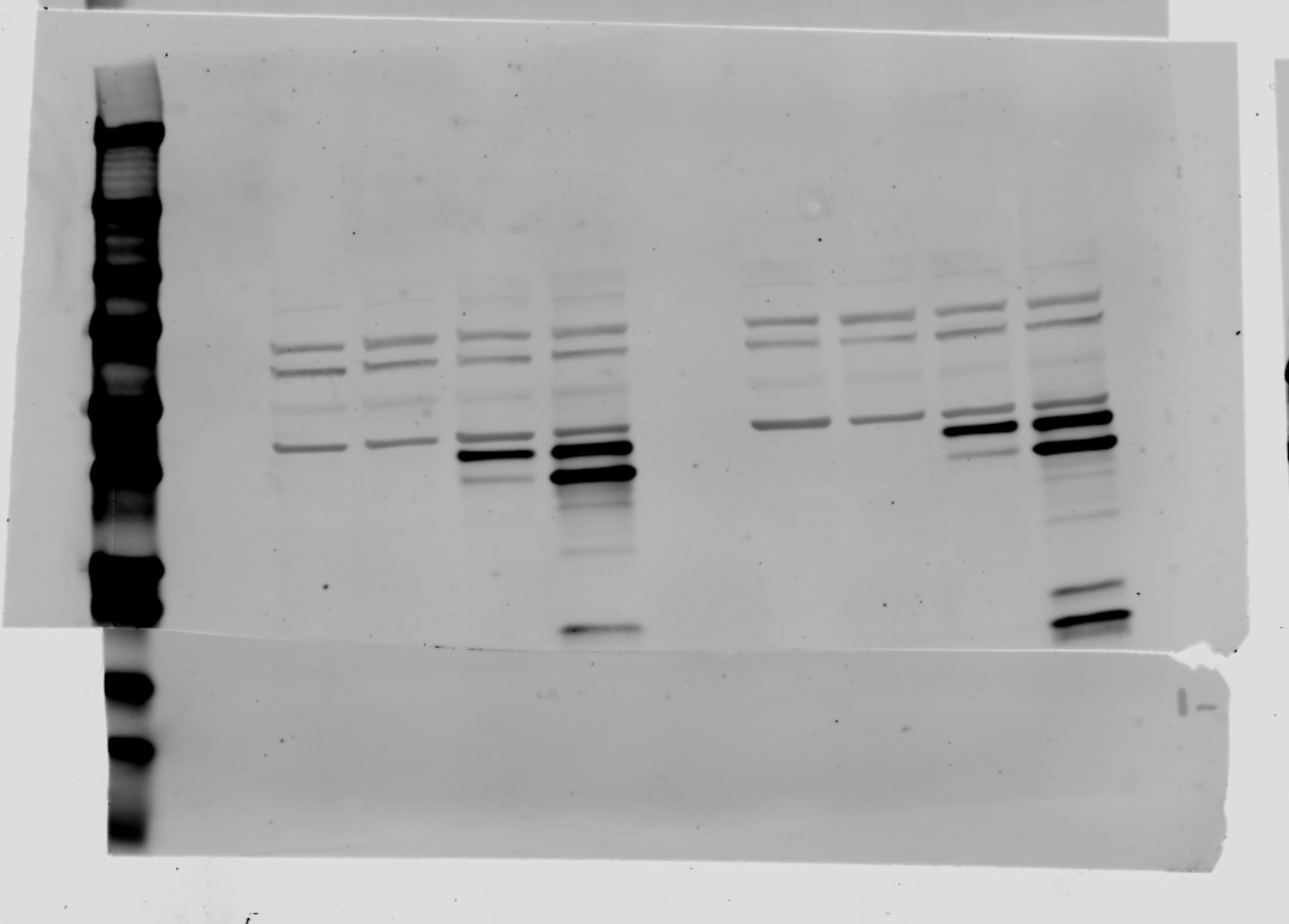

Supplement: Figure 3—source data 1. [file elife-82860-fig3-data1.zip › elife_Figure 3 source data/elife_Fig 3 source data 1/Fig_3E_Source_Data_Unlabeled/Fig_3E_ATAD1_Unlabeled.tif]

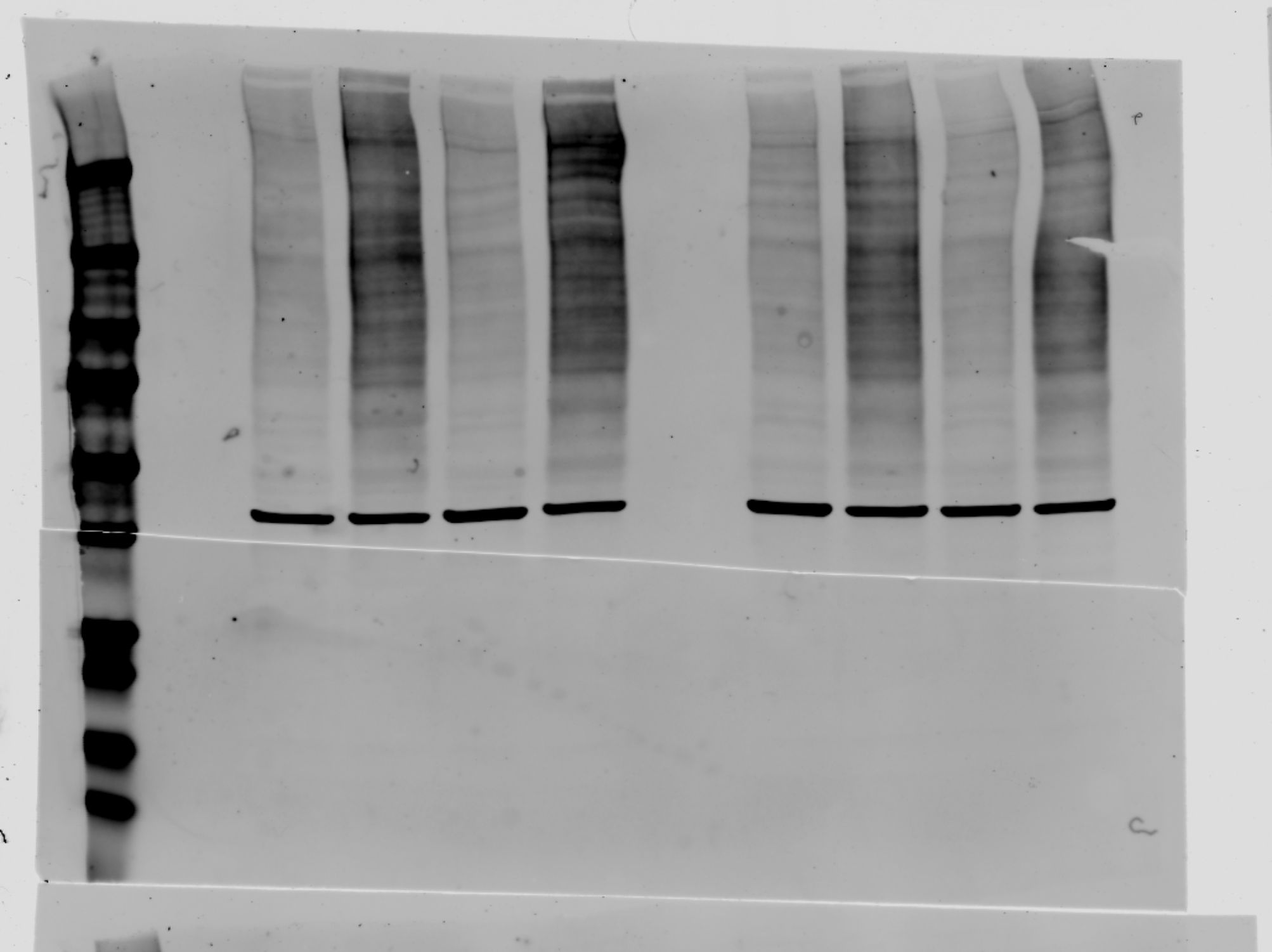

Supplement: Figure 3—source data 1. [file elife-82860-fig3-data1.zip › elife_Figure 3 source data/elife_Fig 3 source data 1/Fig_3E_Source_Data_Unlabeled/Fig_3E_ub_actin_unlabeled.tif]

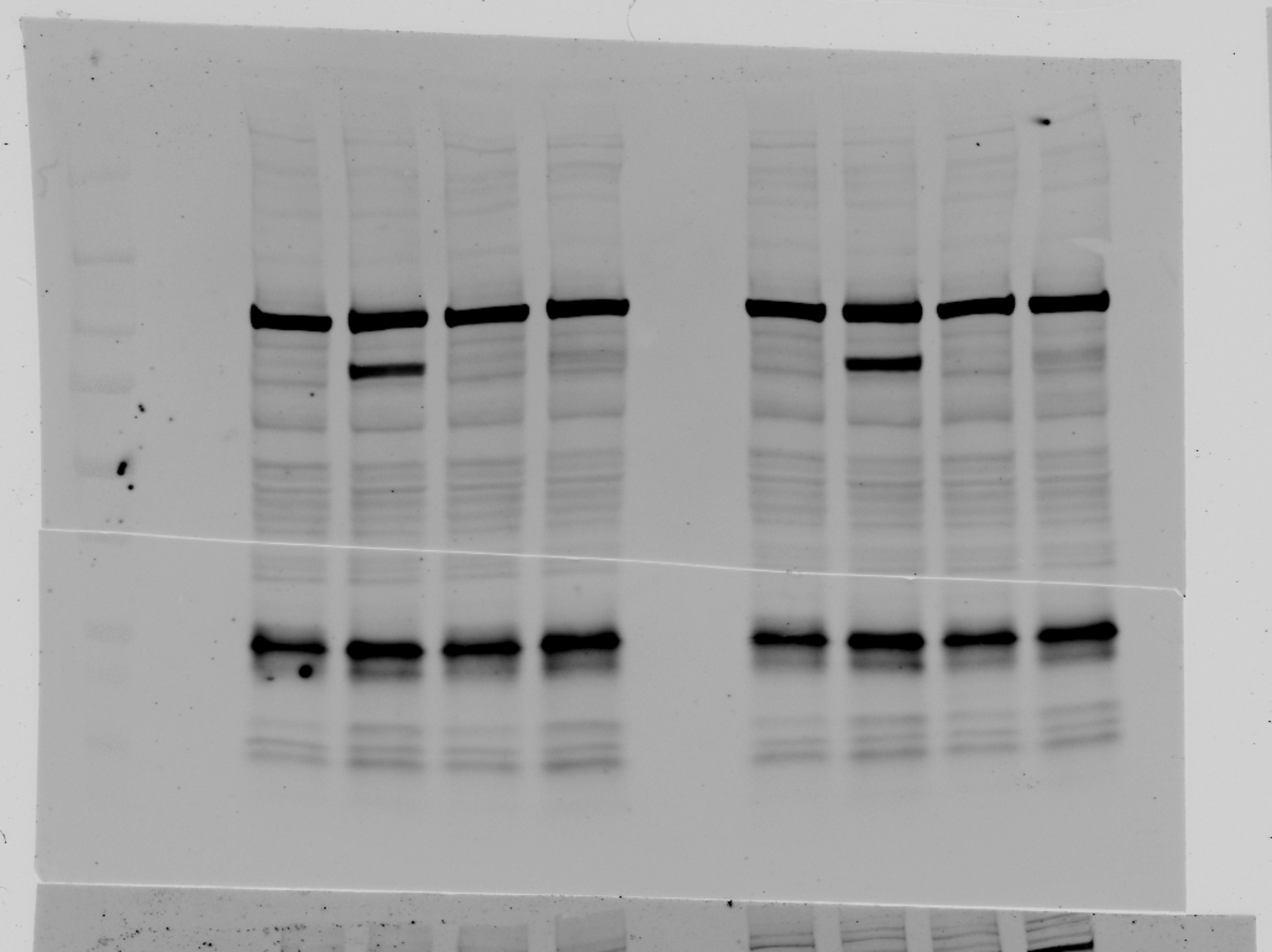

Supplement: Figure 3—source data 1. [file elife-82860-fig3-data1.zip › elife_Figure 3 source data/elife_Fig 3 source data 1/Fig_3E_Source_Data_Unlabeled/Fig_3E_PARP_Unlabeled.tif]

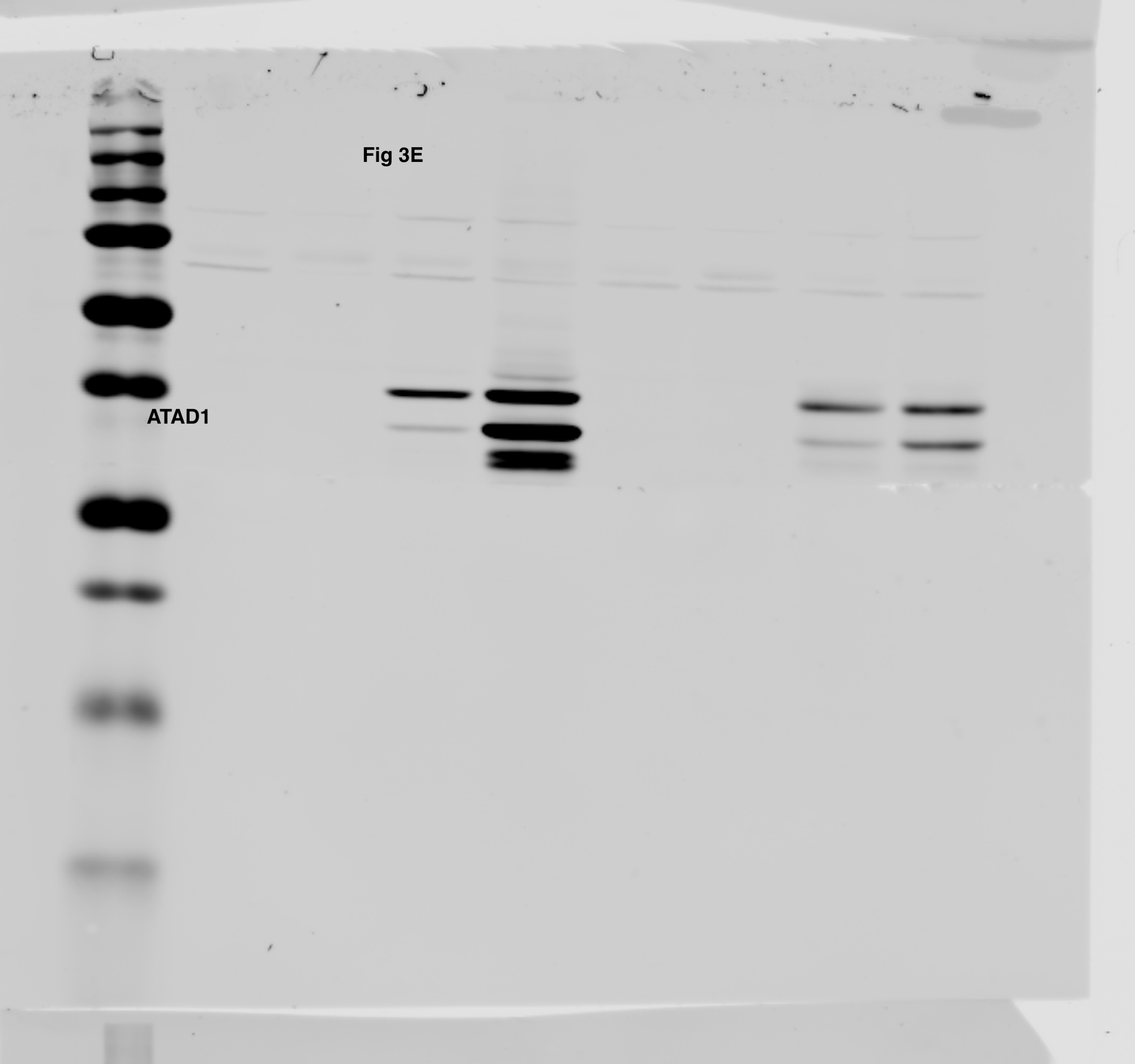

Supplement: Figure 3—source data 1. [file elife-82860-fig3-data1.zip › elife_Figure 3 source data/elife_Fig 3 source data 2/Fig_3F_Source_Data_Labeled/Fig_3F_Source_Data_ATAD1_labeled.tif]

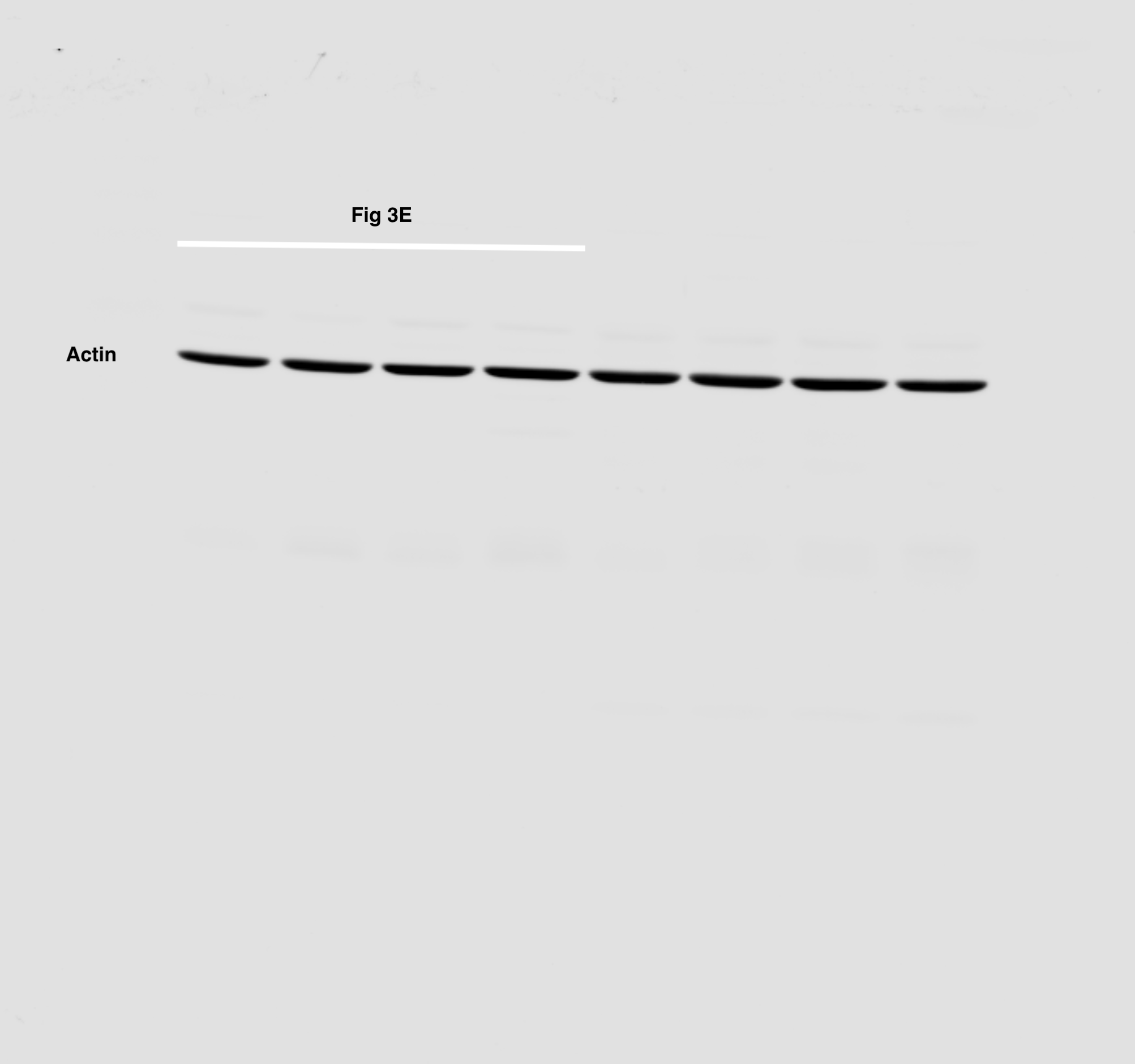

Supplement: Figure 3—source data 1. [file elife-82860-fig3-data1.zip › elife_Figure 3 source data/elife_Fig 3 source data 2/Fig_3F_Source_Data_Labeled/Fig_3F_Source_Data_actin_labeled.tif]

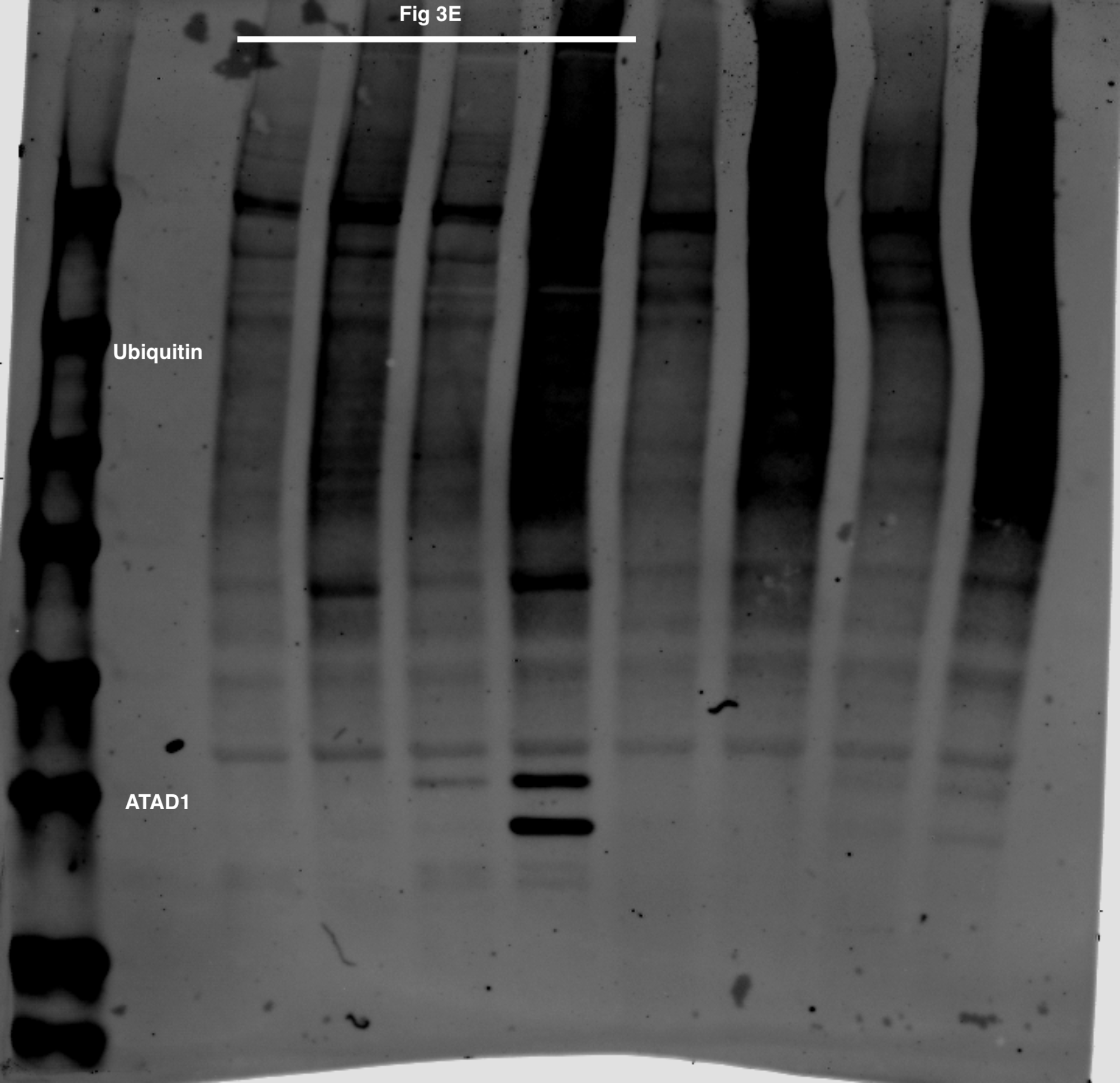

Supplement: Figure 3—source data 1. [file elife-82860-fig3-data1.zip › elife_Figure 3 source data/elife_Fig 3 source data 2/Fig_3F_Source_Data_Labeled/Fig_3F_Source_Data_Ub_ATAD1_labeled.tif]

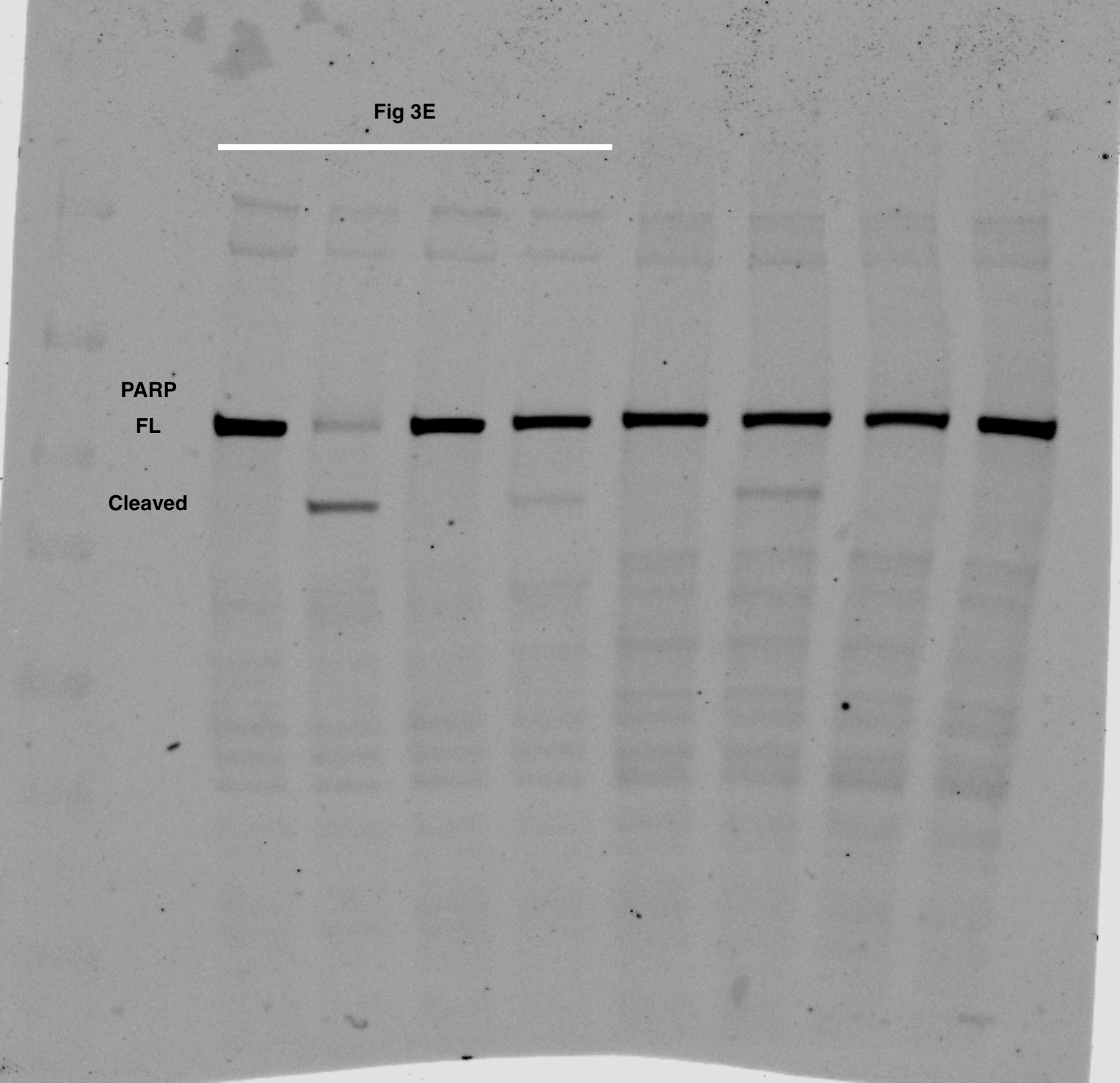

Supplement: Figure 3—source data 1. [file elife-82860-fig3-data1.zip › elife_Figure 3 source data/elife_Fig 3 source data 2/Fig_3F_Source_Data_Labeled/Fig_3F_Source_Data_PARP_labeled.tif]

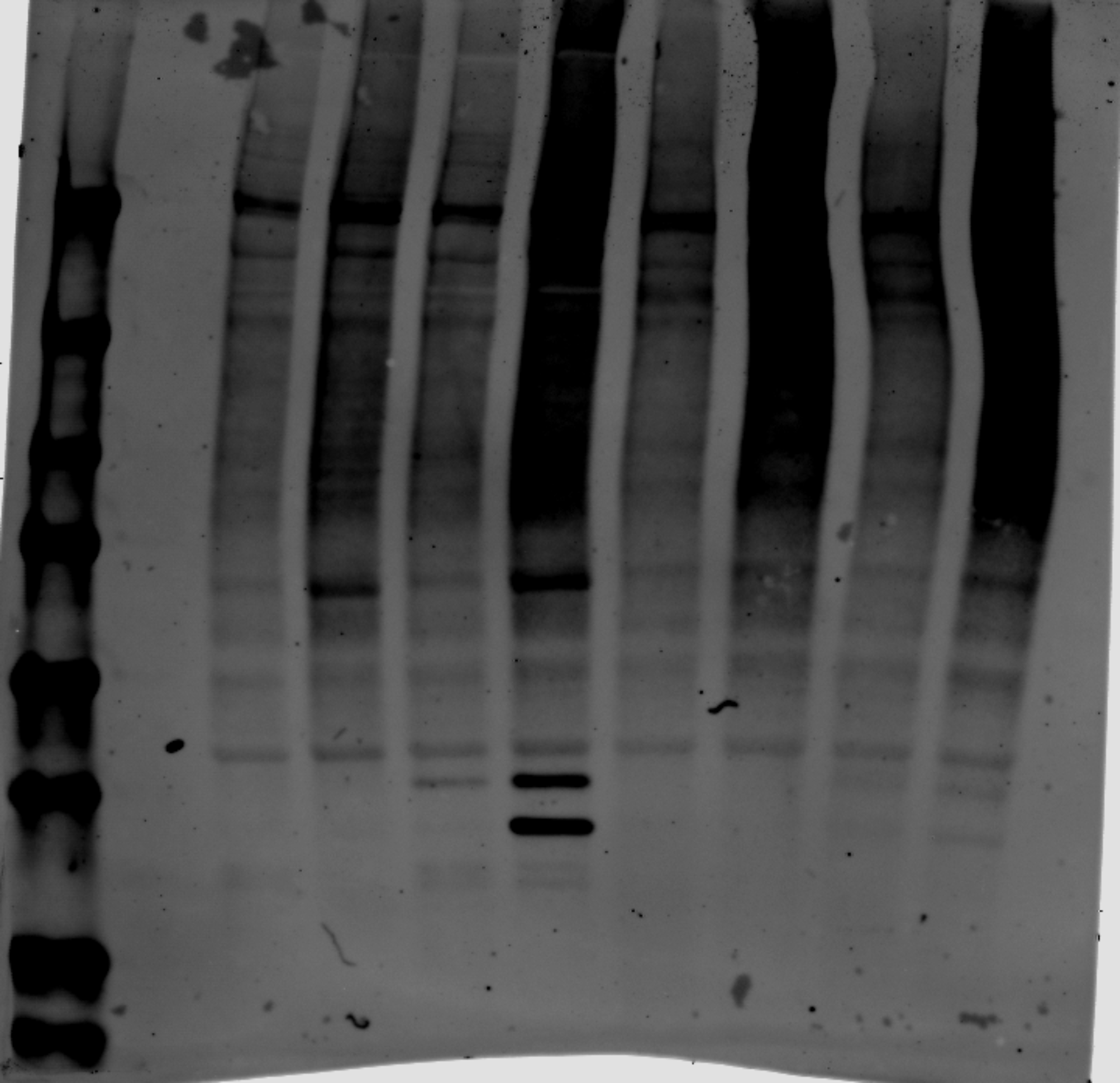

Supplement: Figure 3—source data 1. [file elife-82860-fig3-data1.zip › elife_Figure 3 source data/elife_Fig 3 source data 2/Fig_3F_Source_Data_Unlabled/Fig_3F_Source_Data_Ub_ATAD1_Unlabeled.tif]

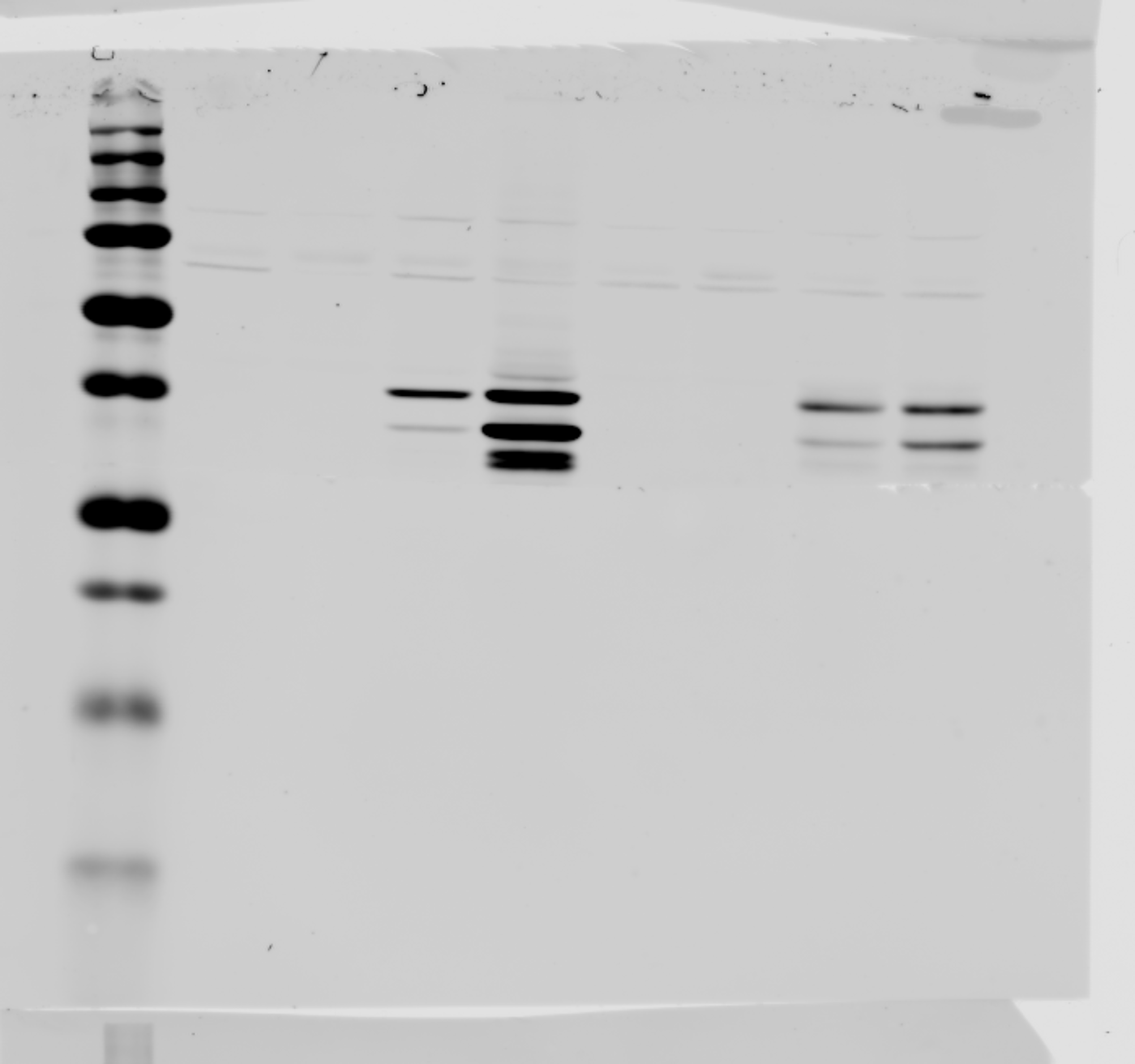

Supplement: Figure 3—source data 1. [file elife-82860-fig3-data1.zip › elife_Figure 3 source data/elife_Fig 3 source data 2/Fig_3F_Source_Data_Unlabled/Fig_3F_Source_Data_ATAD1_unlabeled.tif]

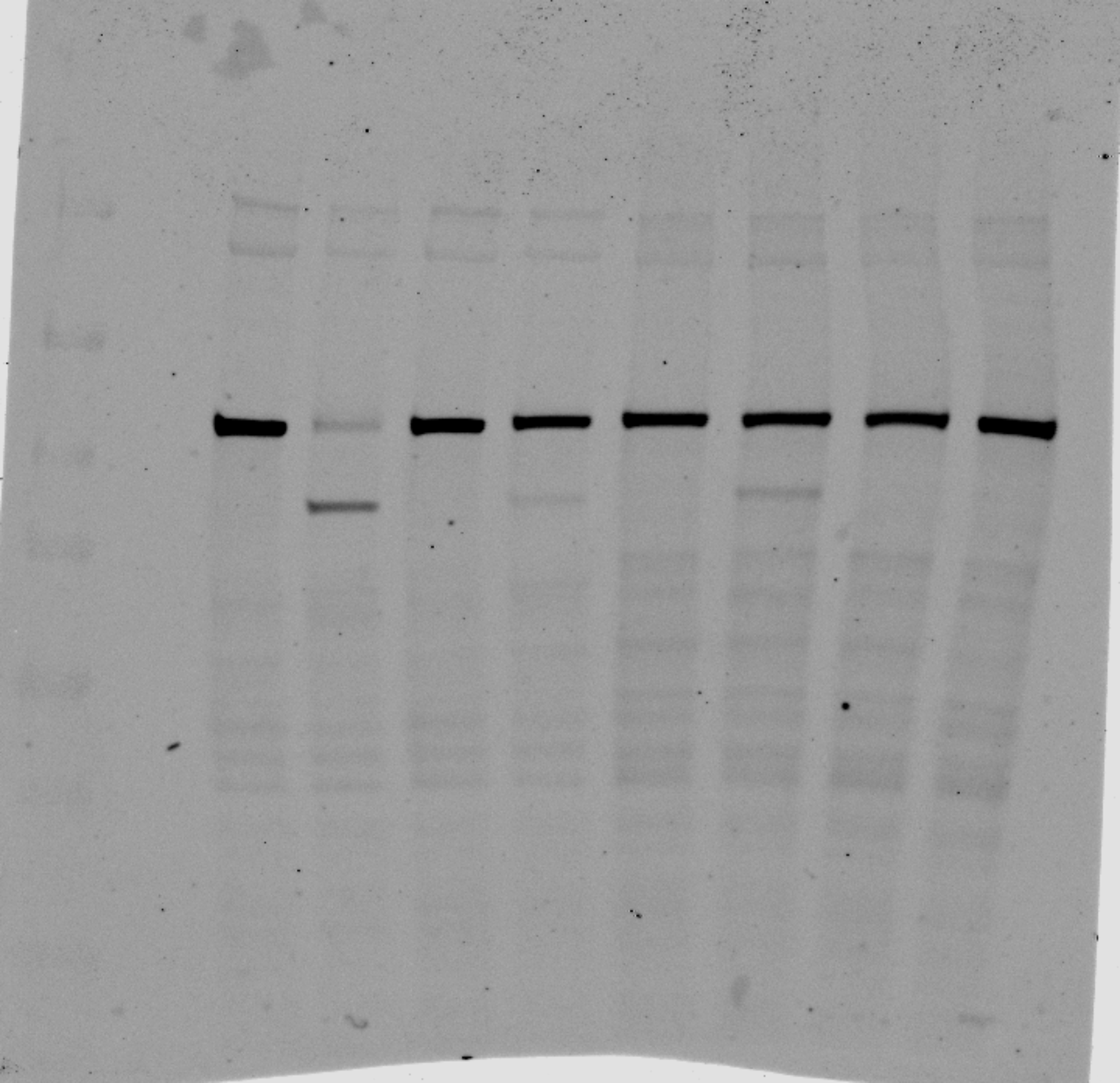

Supplement: Figure 3—source data 1. [file elife-82860-fig3-data1.zip › elife_Figure 3 source data/elife_Fig 3 source data 2/Fig_3F_Source_Data_Unlabled/Fig_3F_Source_Data_PARP_unlabeled.tif]

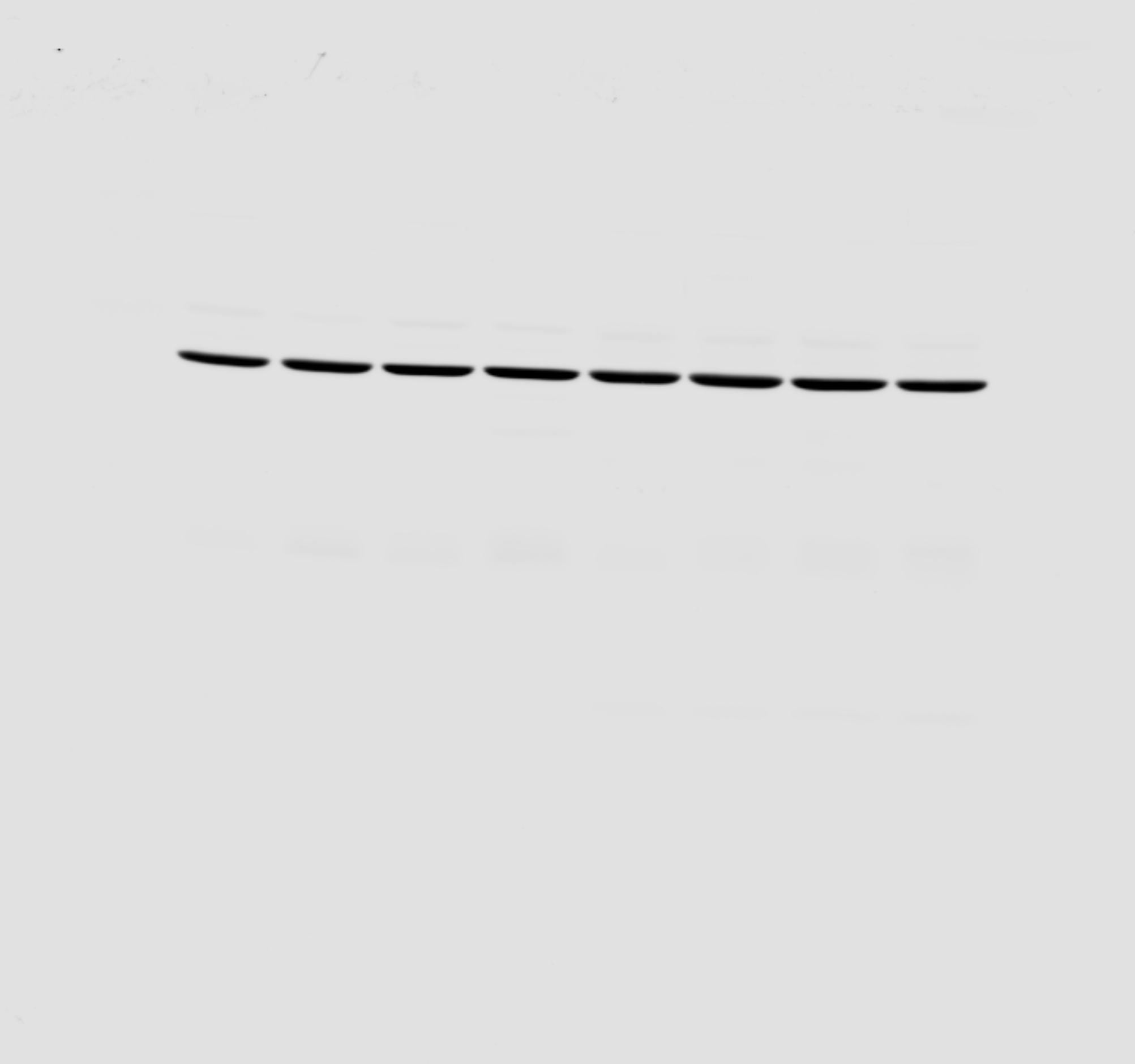

Supplement: Figure 3—source data 1. [file elife-82860-fig3-data1.zip › elife_Figure 3 source data/elife_Fig 3 source data 2/Fig_3F_Source_Data_Unlabled/Fig_3F_Source_Data_actin_Unlabeled.tif]

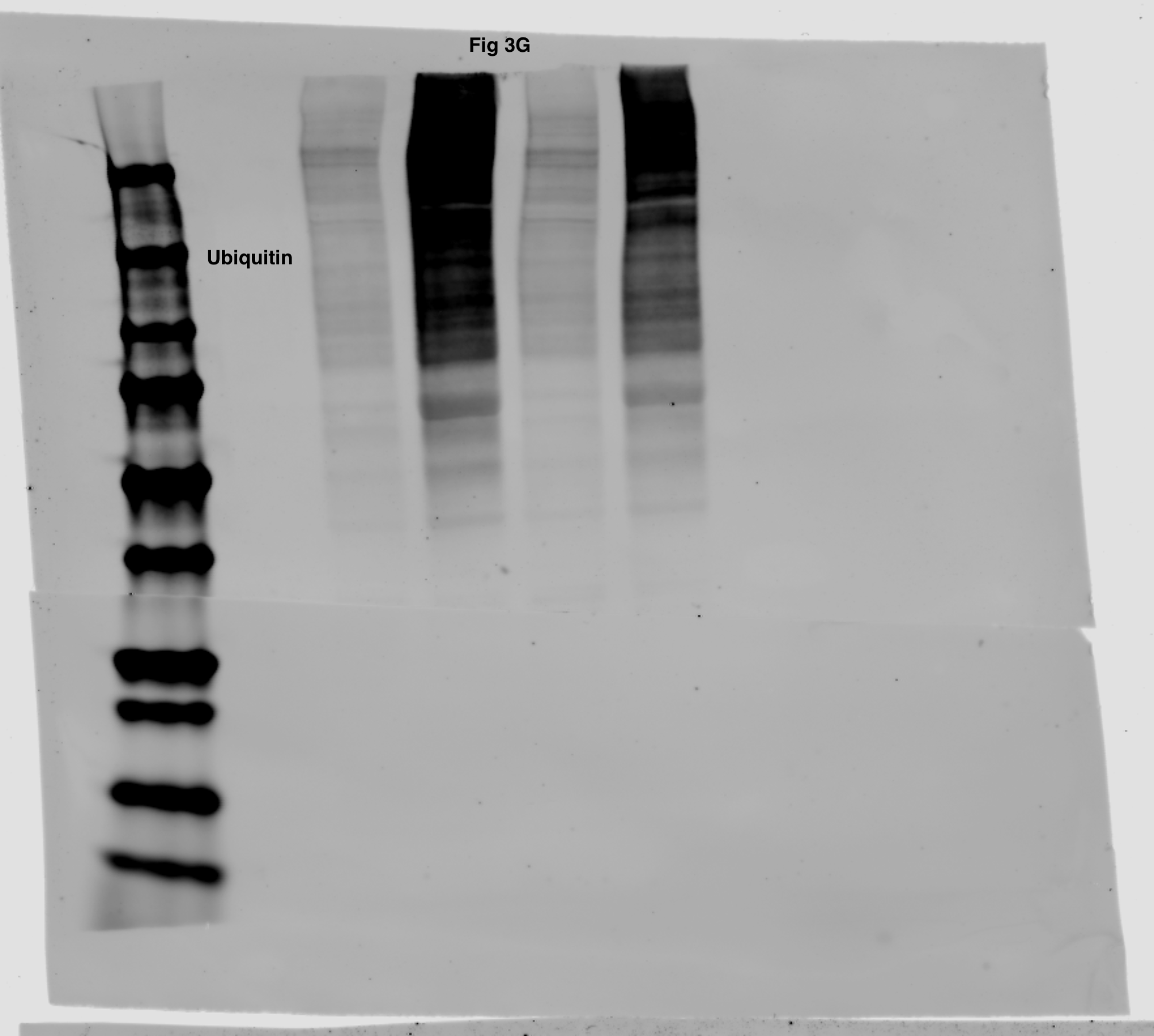

Supplement: Figure 3—source data 1. [file elife-82860-fig3-data1.zip › elife_Figure 3 source data/elife_Fig 3 source data 3/Fig_3G_Source_Data_Labeled/Fig_3G_Source_Data_Ub_labeled.tif]

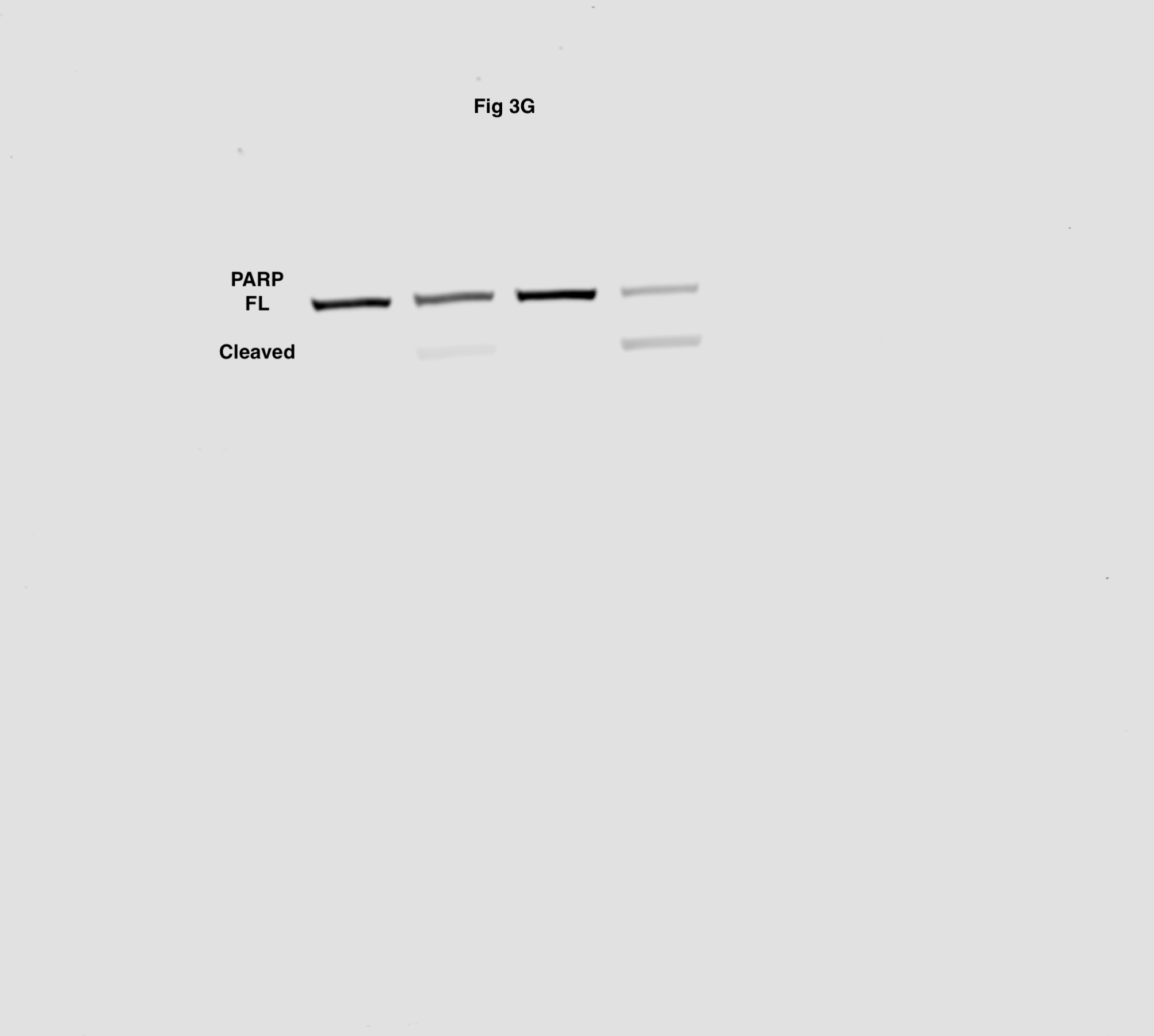

Supplement: Figure 3—source data 1. [file elife-82860-fig3-data1.zip › elife_Figure 3 source data/elife_Fig 3 source data 3/Fig_3G_Source_Data_Labeled/Fig_3G_Source_Data_PARP_labeled.tif]

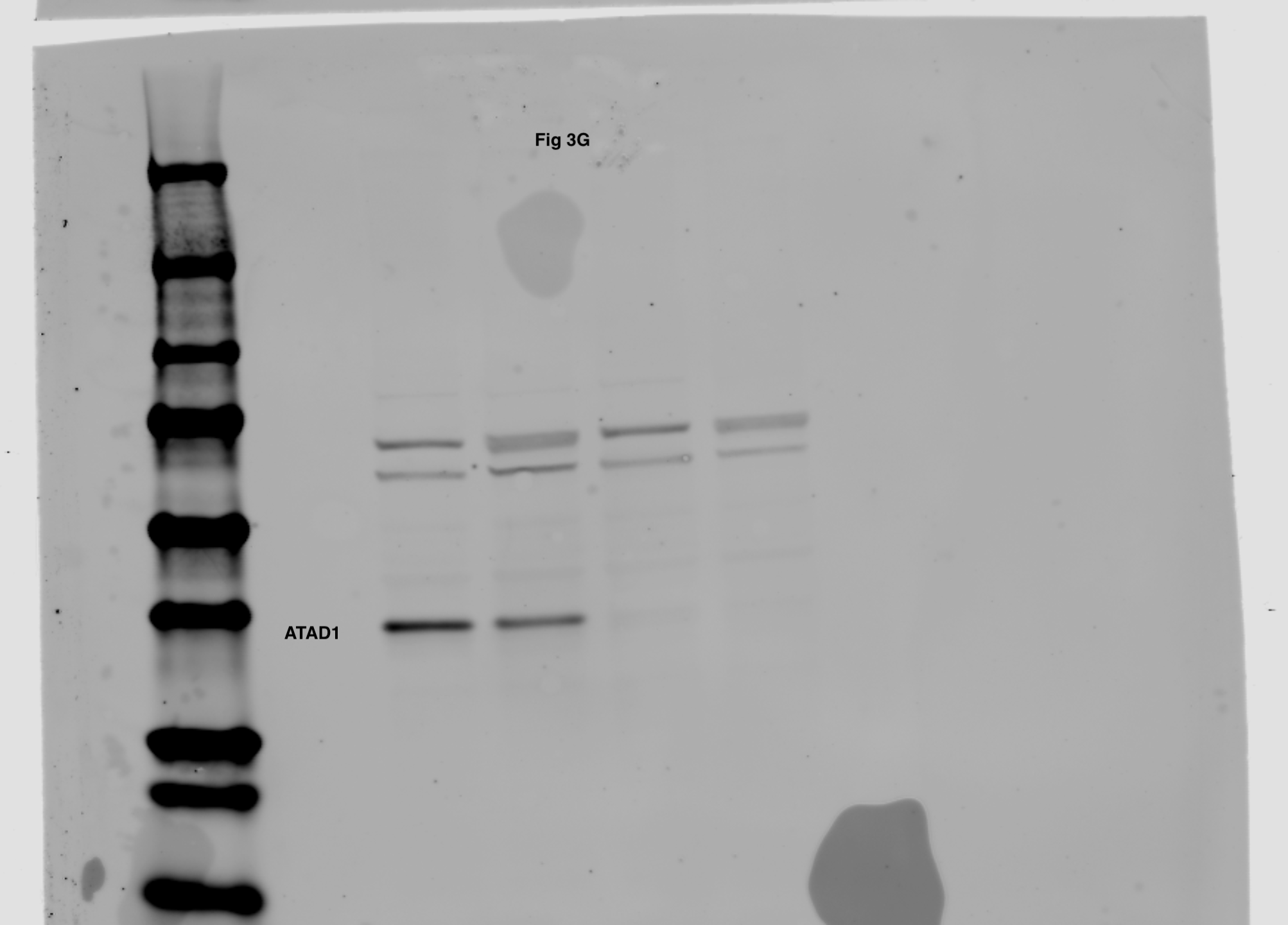

Supplement: Figure 3—source data 1. [file elife-82860-fig3-data1.zip › elife_Figure 3 source data/elife_Fig 3 source data 3/Fig_3G_Source_Data_Labeled/Fig_3G_Source_Data_ATAD1_labeled.tif]

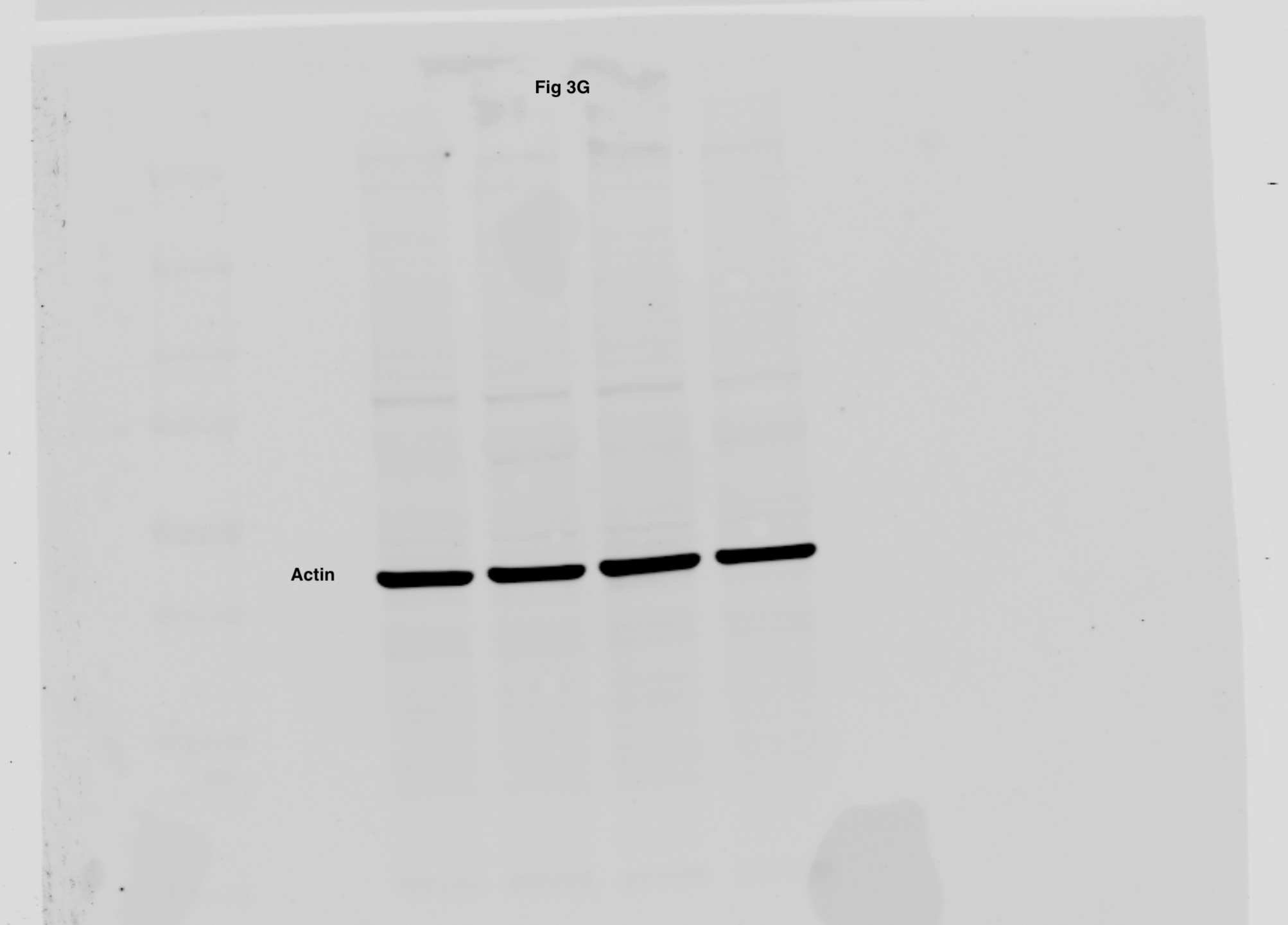

Supplement: Figure 3—source data 1. [file elife-82860-fig3-data1.zip › elife_Figure 3 source data/elife_Fig 3 source data 3/Fig_3G_Source_Data_Labeled/Fig_3G_Source_Data_Actin_labeled.tif]

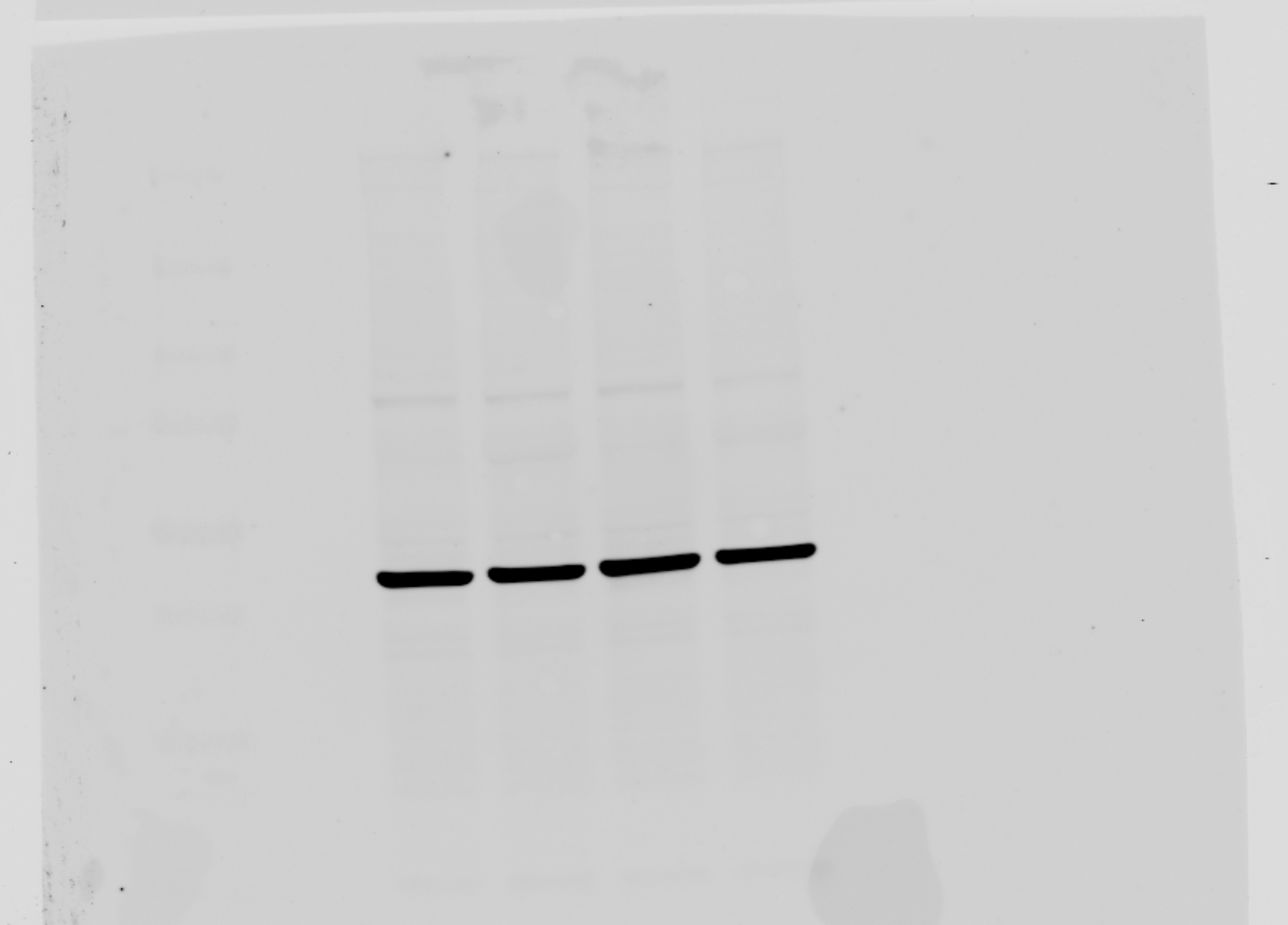

Supplement: Figure 3—source data 1. [file elife-82860-fig3-data1.zip › elife_Figure 3 source data/elife_Fig 3 source data 3/Fig_3G_Source_Data_Unlabeled/Fig_3G_Source_Data_Actin_Unlabeled.tif]

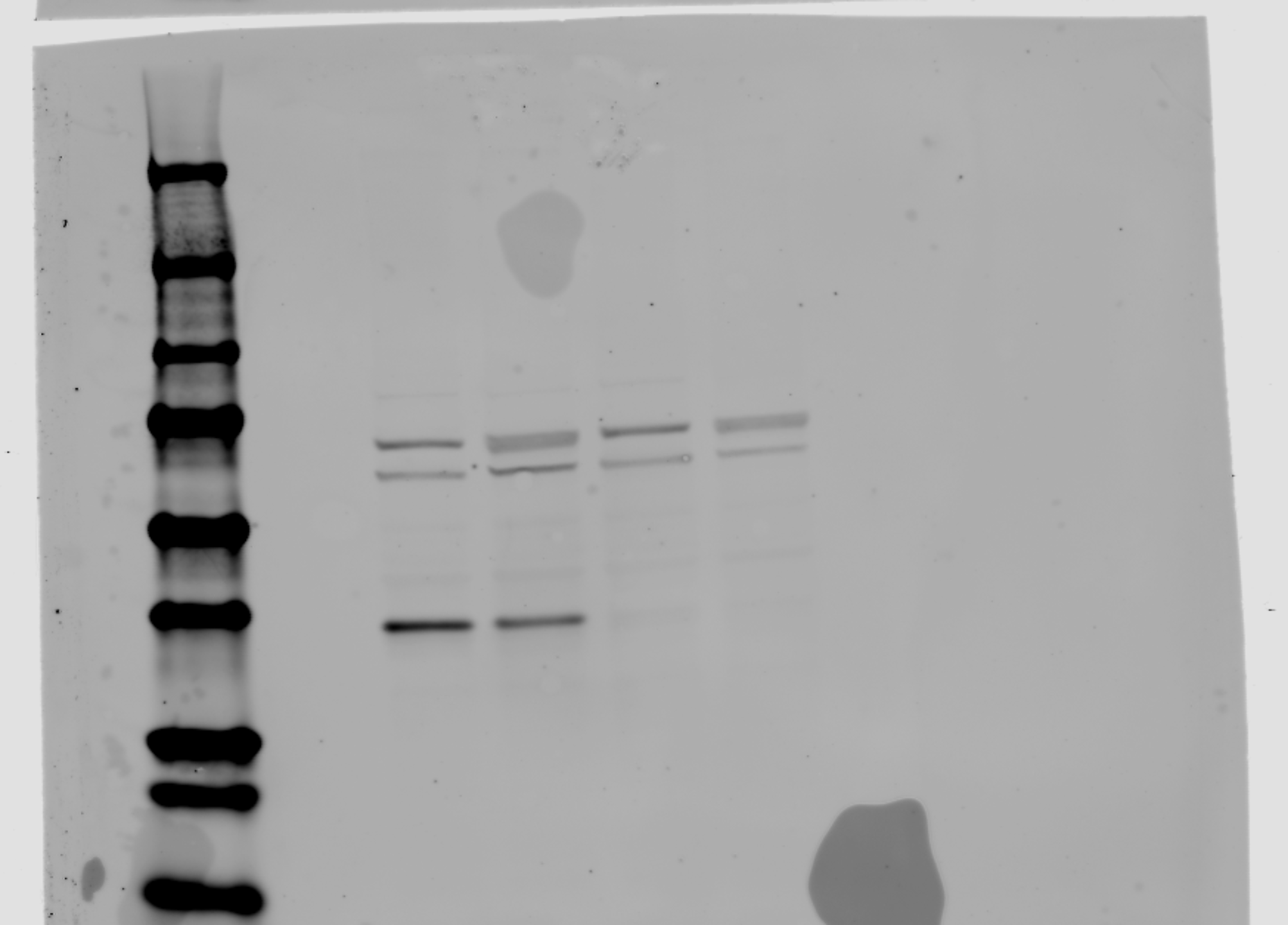

Supplement: Figure 3—source data 1. [file elife-82860-fig3-data1.zip › elife_Figure 3 source data/elife_Fig 3 source data 3/Fig_3G_Source_Data_Unlabeled/Fig_3G_Source_Data_ATAD1_Unlabeled.tif]

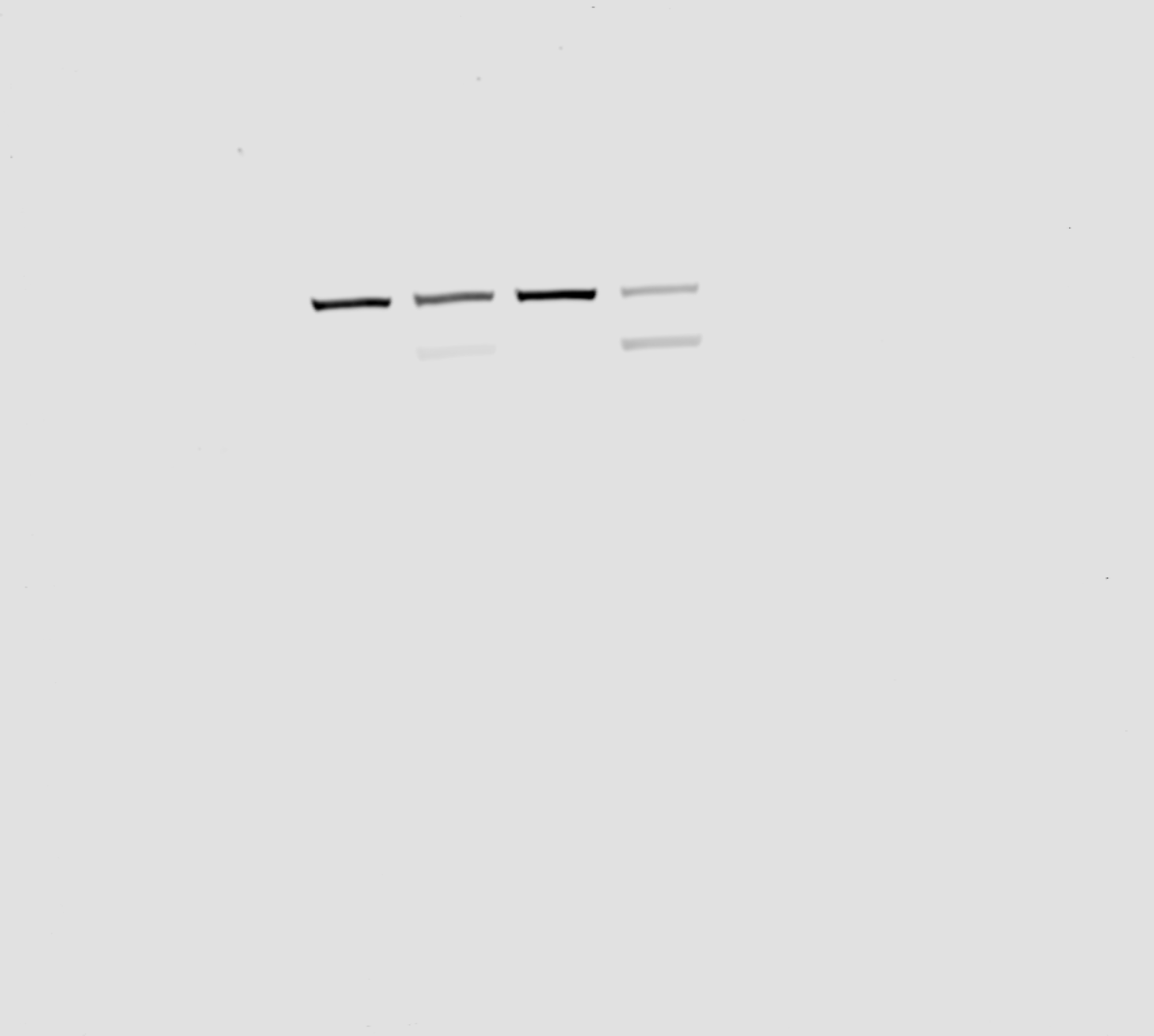

Supplement: Figure 3—source data 1. [file elife-82860-fig3-data1.zip › elife_Figure 3 source data/elife_Fig 3 source data 3/Fig_3G_Source_Data_Unlabeled/Fig_3G_Source_Data_PARP_Unlabeled.tif]

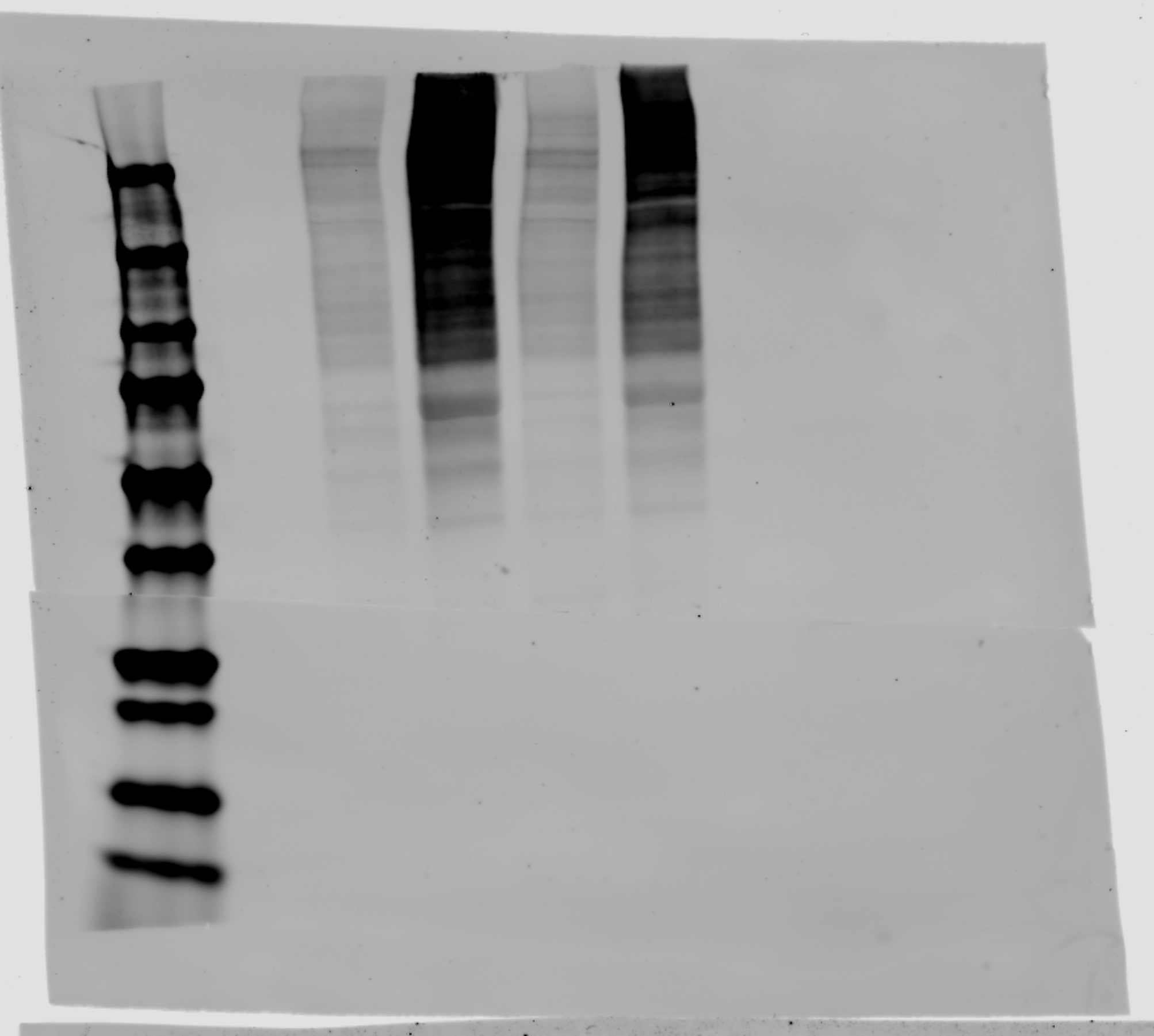

Supplement: Figure 3—source data 1. [file elife-82860-fig3-data1.zip › elife_Figure 3 source data/elife_Fig 3 source data 3/Fig_3G_Source_Data_Unlabeled/Fig_3G_Source_Data_Ub_Unlabeled.tif]

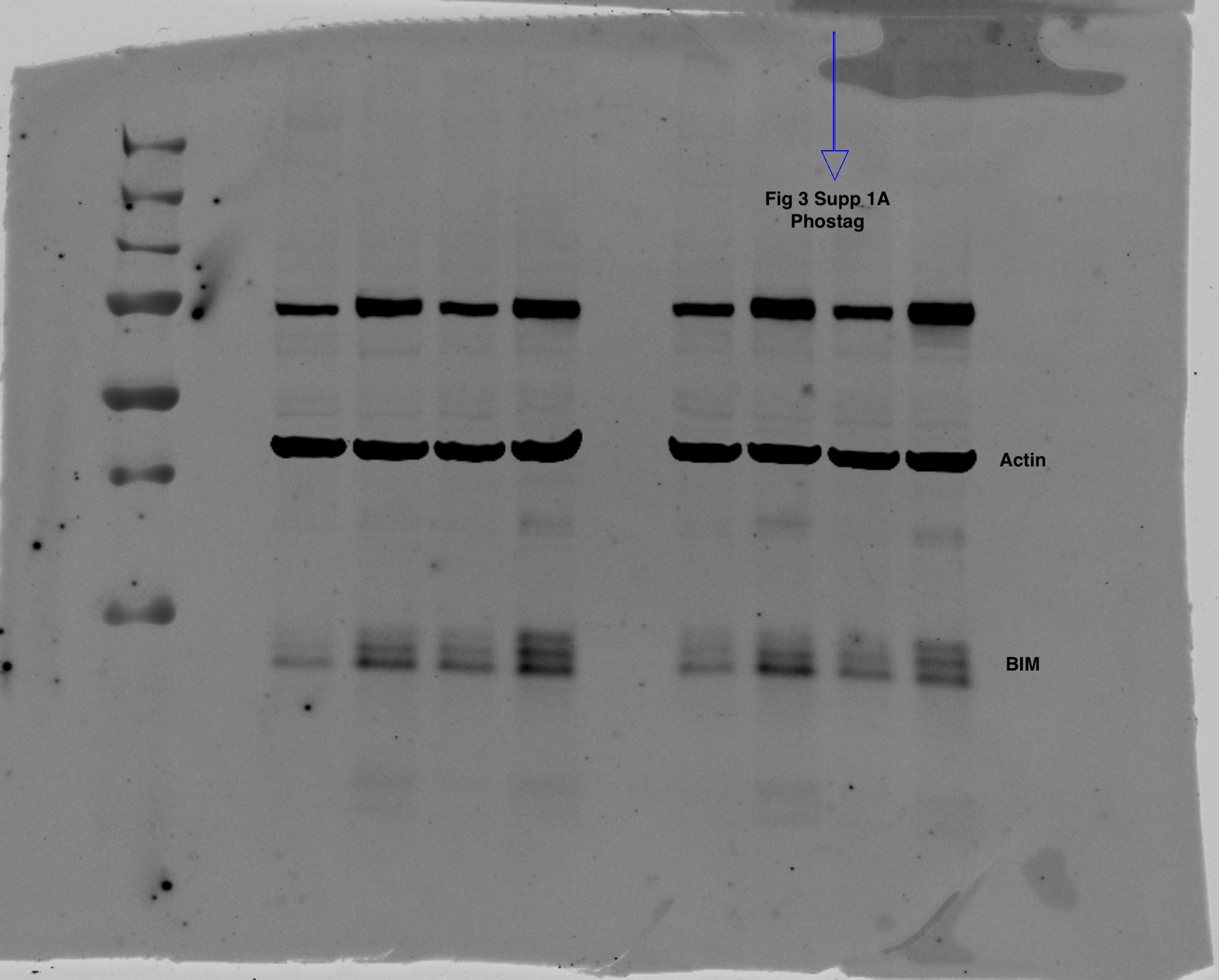

Supplement: Figure 3—figure supplement 1—source data 1. [file elife-82860-fig3-figsupp1-data1.zip › elife_Figure 3 Supp 1 source data/elife_Fig 3 Supp 1 source data 1/Fig_3_Supp_1A_Source_Data_Labeled/Fig_3_Supp_1_ptag_BIM_Actin_labeled.tif]

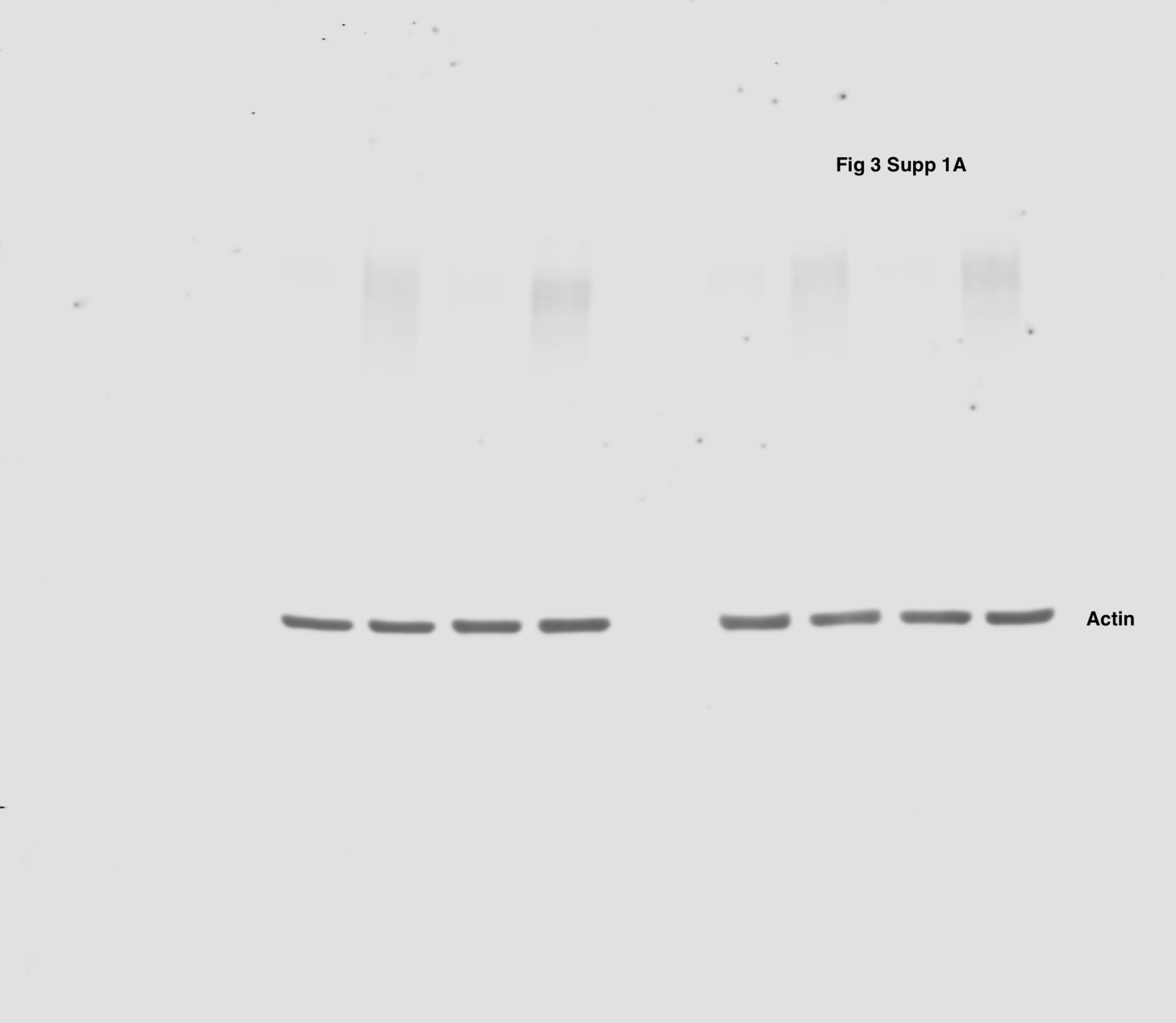

Supplement: Figure 3—figure supplement 1—source data 1. [file elife-82860-fig3-figsupp1-data1.zip › elife_Figure 3 Supp 1 source data/elife_Fig 3 Supp 1 source data 1/Fig_3_Supp_1A_Source_Data_Labeled/Fig_3_Supp_1A_Actin_labeled.tif]

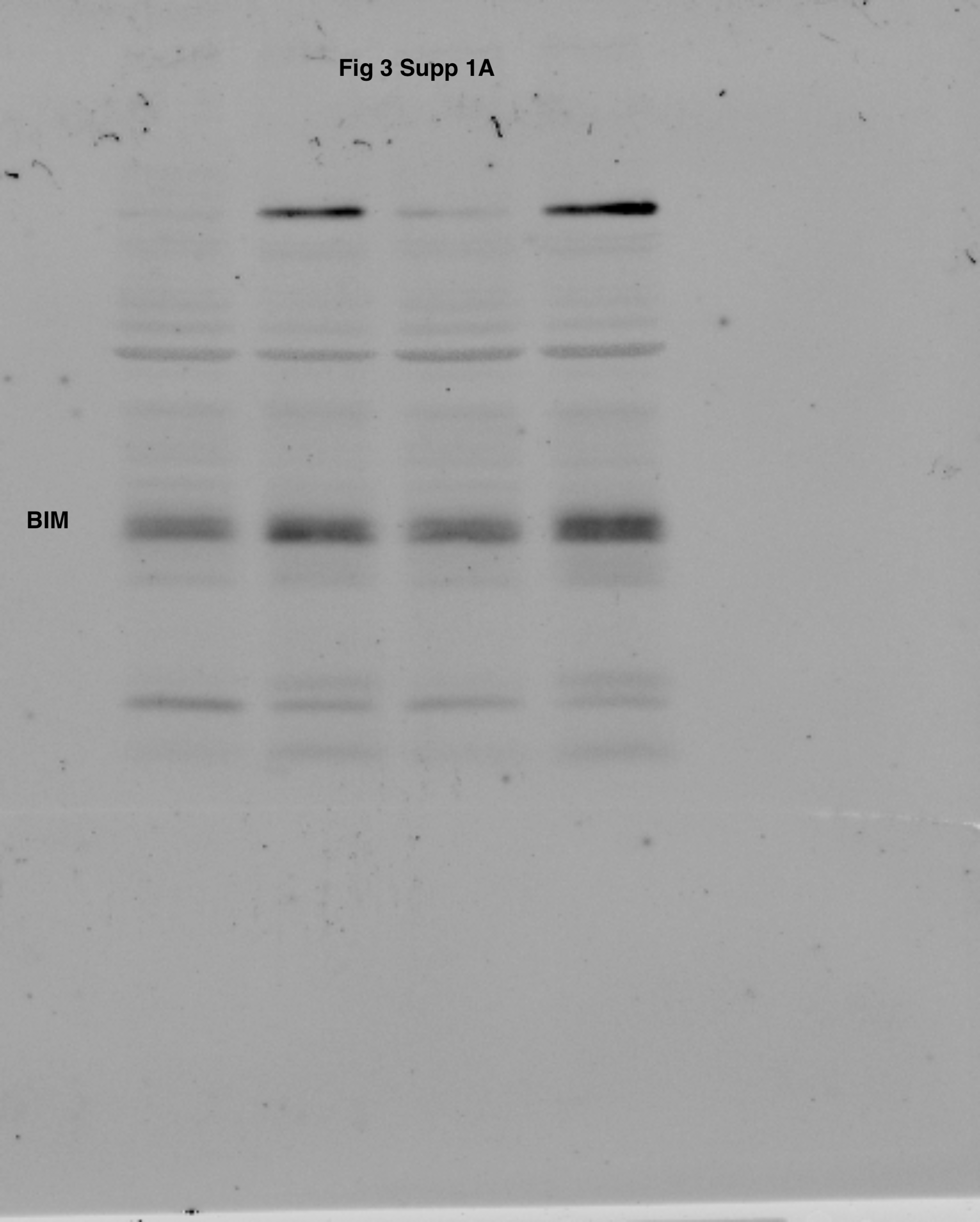

Supplement: Figure 3—figure supplement 1—source data 1. [file elife-82860-fig3-figsupp1-data1.zip › elife_Figure 3 Supp 1 source data/elife_Fig 3 Supp 1 source data 1/Fig_3_Supp_1A_Source_Data_Labeled/Fig_3_Supp_1_BIM_labeled.tif]

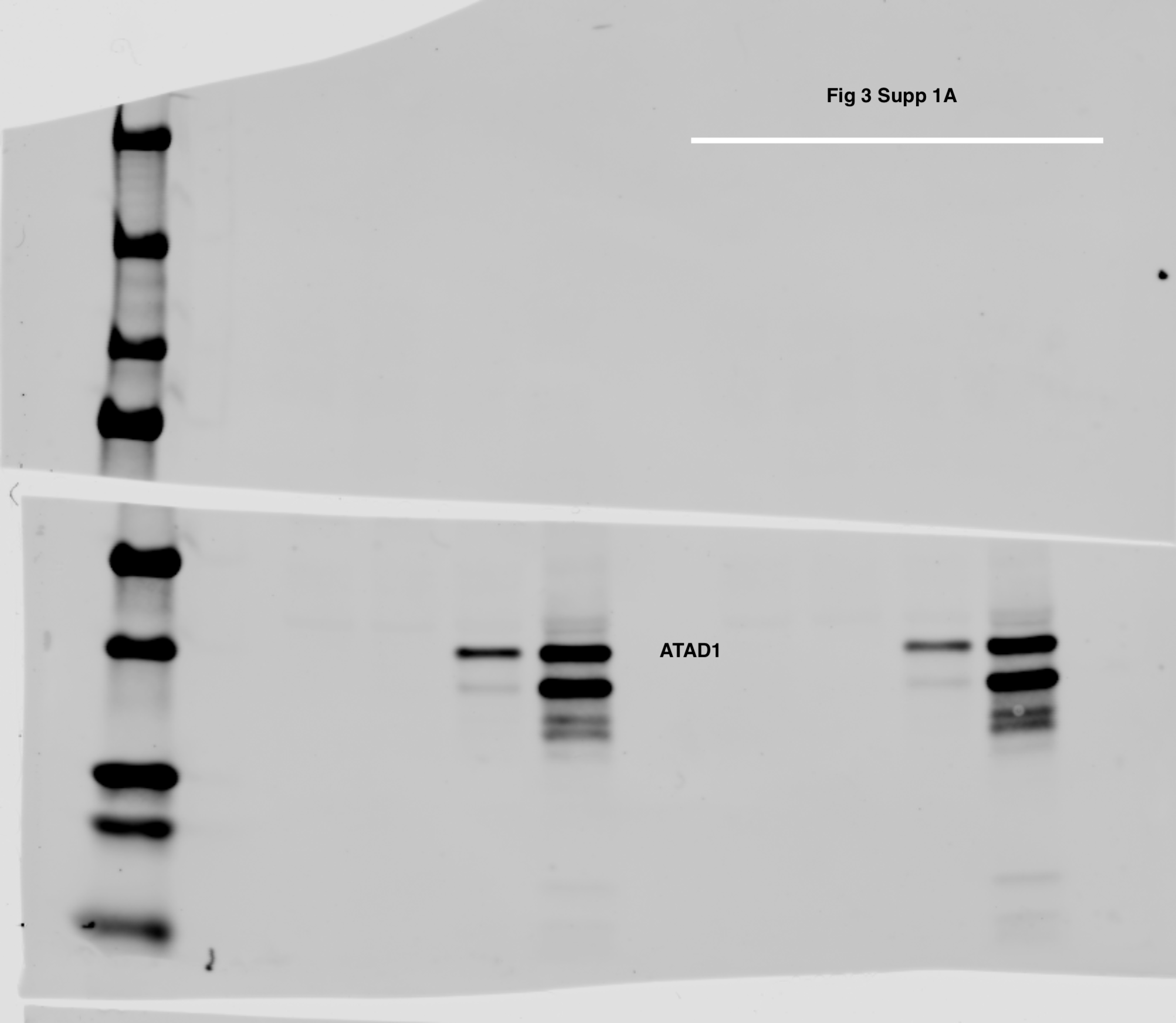

Supplement: Figure 3—figure supplement 1—source data 1. [file elife-82860-fig3-figsupp1-data1.zip › elife_Figure 3 Supp 1 source data/elife_Fig 3 Supp 1 source data 1/Fig_3_Supp_1A_Source_Data_Labeled/Fig_3_Supp_1_ATAD1_labeled.tiff]

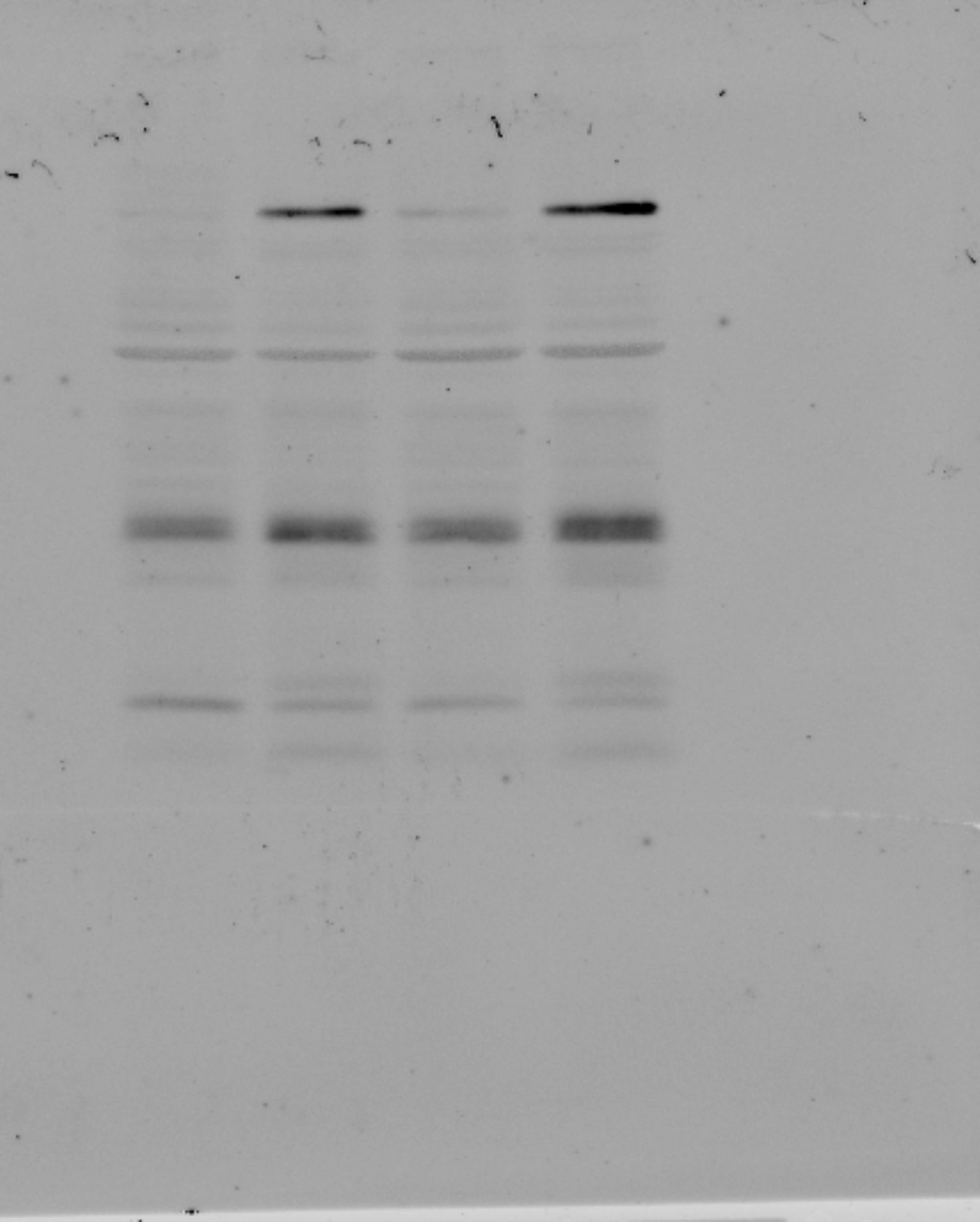

Supplement: Figure 3—figure supplement 1—source data 1. [file elife-82860-fig3-figsupp1-data1.zip › elife_Figure 3 Supp 1 source data/elife_Fig 3 Supp 1 source data 1/Fig_3_Supp_1A_Source_Data_Unlabeled/Fig_3_Supp_1_BIM_Unlabeled.tif]

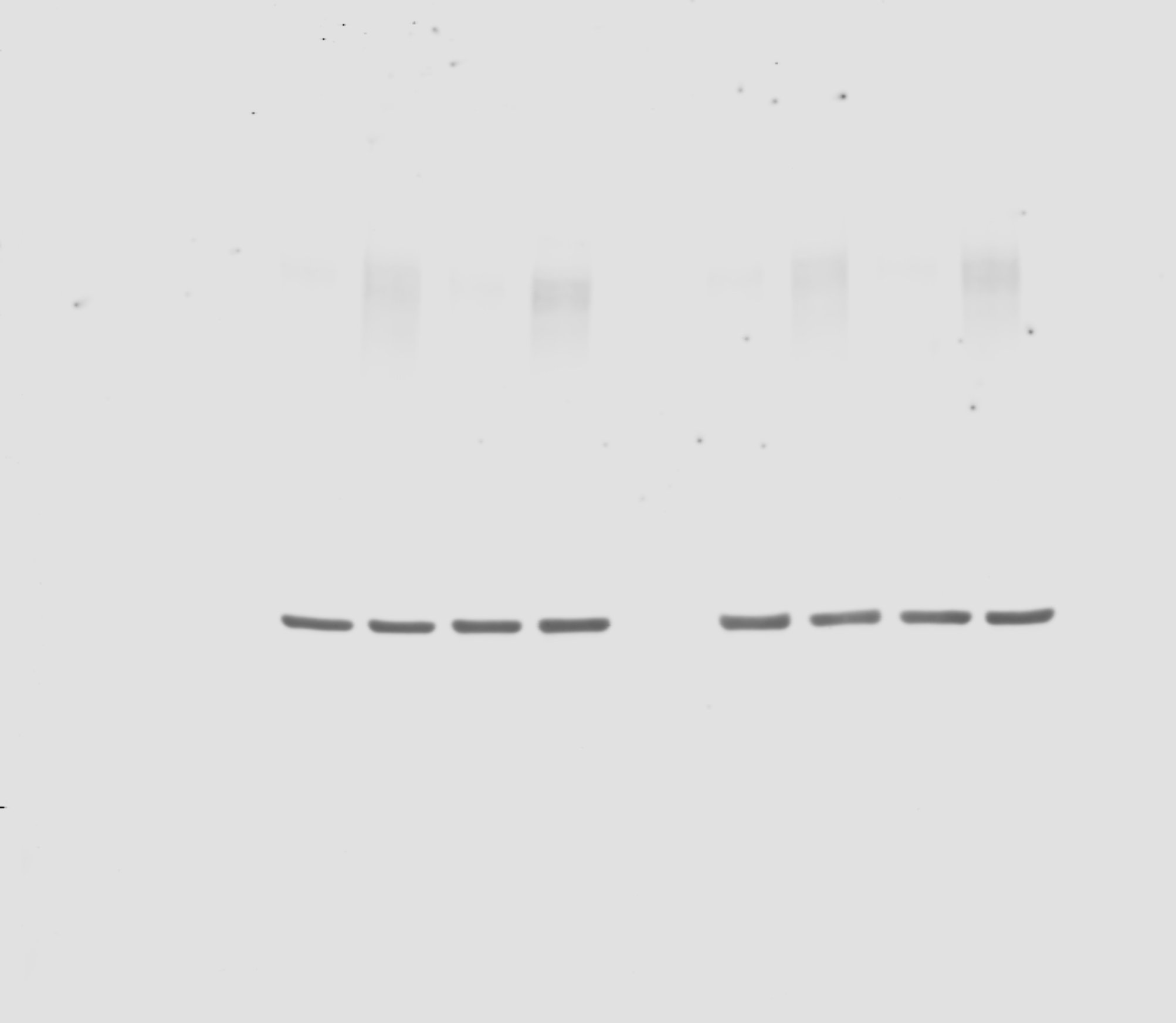

Supplement: Figure 3—figure supplement 1—source data 1. [file elife-82860-fig3-figsupp1-data1.zip › elife_Figure 3 Supp 1 source data/elife_Fig 3 Supp 1 source data 1/Fig_3_Supp_1A_Source_Data_Unlabeled/Fig_3_Supp_1A_Actin_Unlabeled.tif]

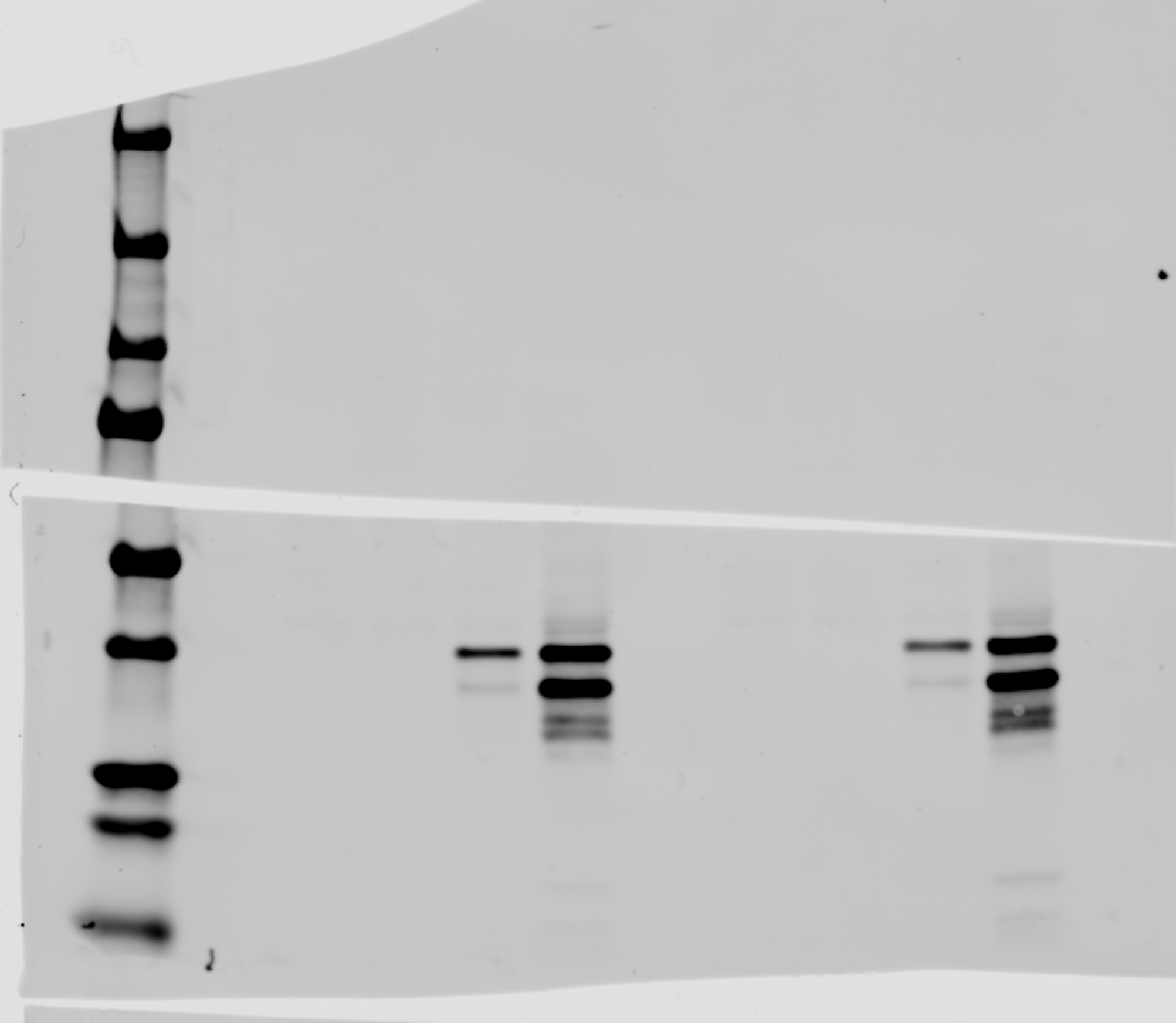

Supplement: Figure 3—figure supplement 1—source data 1. [file elife-82860-fig3-figsupp1-data1.zip › elife_Figure 3 Supp 1 source data/elife_Fig 3 Supp 1 source data 1/Fig_3_Supp_1A_Source_Data_Unlabeled/Fig_3_Supp_1_ATAD1_unlabeled.tiff]

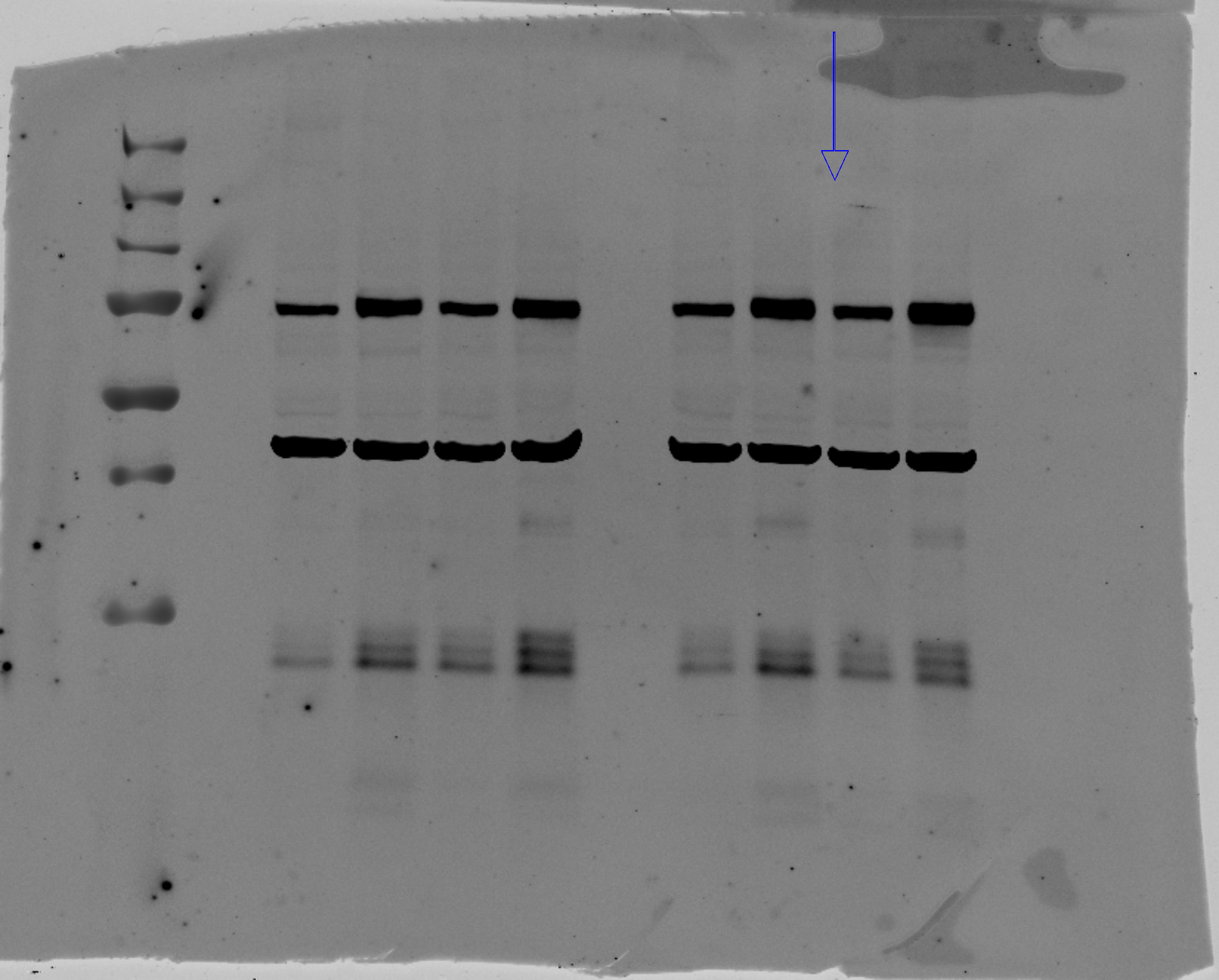

Supplement: Figure 3—figure supplement 1—source data 1. [file elife-82860-fig3-figsupp1-data1.zip › elife_Figure 3 Supp 1 source data/elife_Fig 3 Supp 1 source data 1/Fig_3_Supp_1A_Source_Data_Unlabeled/Fig_3_Supp_1_ptag_BIM_Actin_unlabeled.tif]

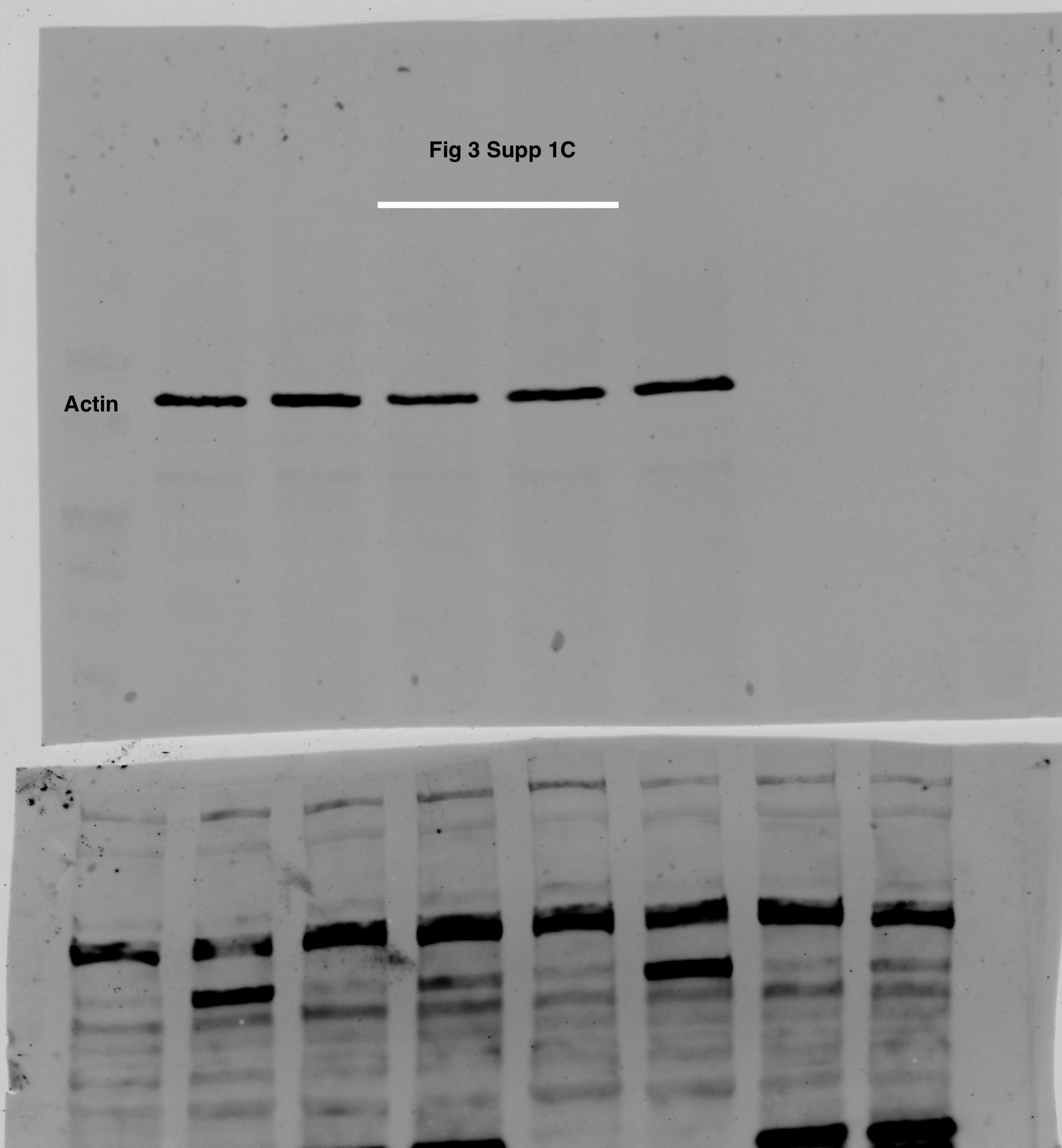

Supplement: Figure 3—figure supplement 1—source data 1. [file elife-82860-fig3-figsupp1-data1.zip › elife_Figure 3 Supp 1 source data/elife_Fig 3 Supp 1 source data 2/Fig_3_Supp_1C_Source_Data_Labeled/Fig_3_Supp_1C_Actin_labeled.tif]

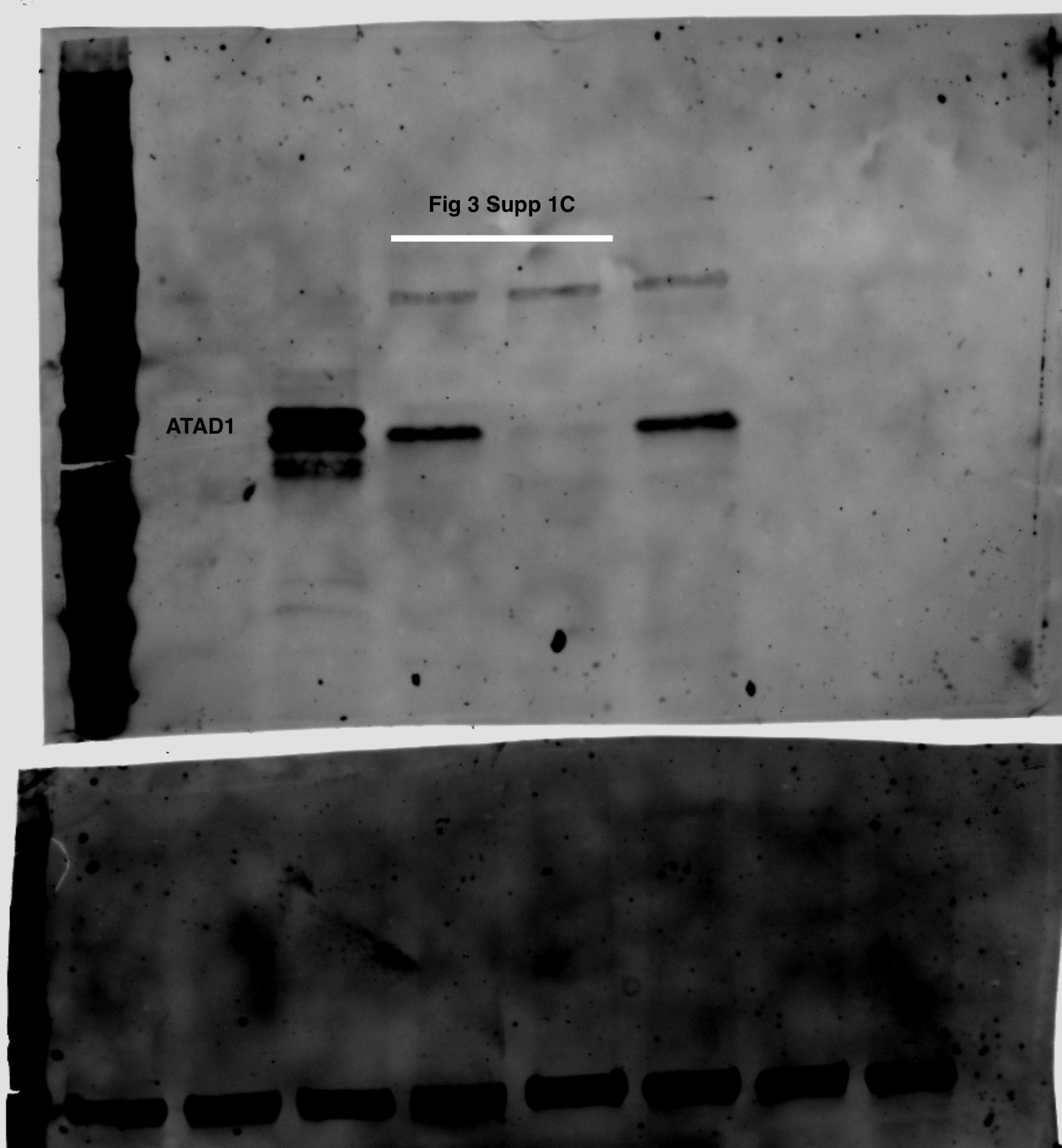

Supplement: Figure 3—figure supplement 1—source data 1. [file elife-82860-fig3-figsupp1-data1.zip › elife_Figure 3 Supp 1 source data/elife_Fig 3 Supp 1 source data 2/Fig_3_Supp_1C_Source_Data_Labeled/Fig_3_Supp_1C_ATAD1_labeled.tif]

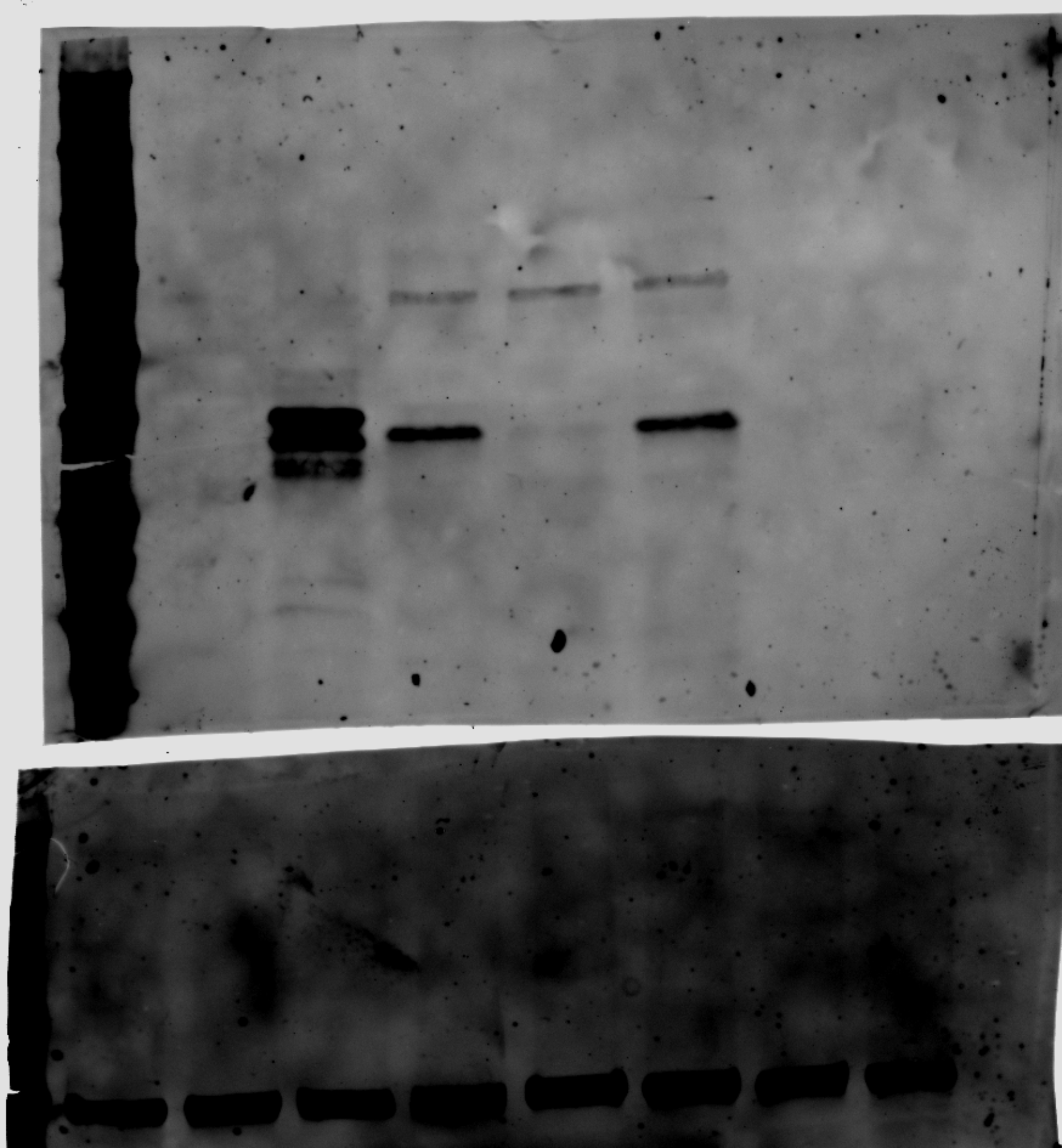

Supplement: Figure 3—figure supplement 1—source data 1. [file elife-82860-fig3-figsupp1-data1.zip › elife_Figure 3 Supp 1 source data/elife_Fig 3 Supp 1 source data 2/Fig_3_Supp_1C_Source_Data_Unlabeled/Fig_3_Supp_1C_ATAD1_Unlabeled.tif]

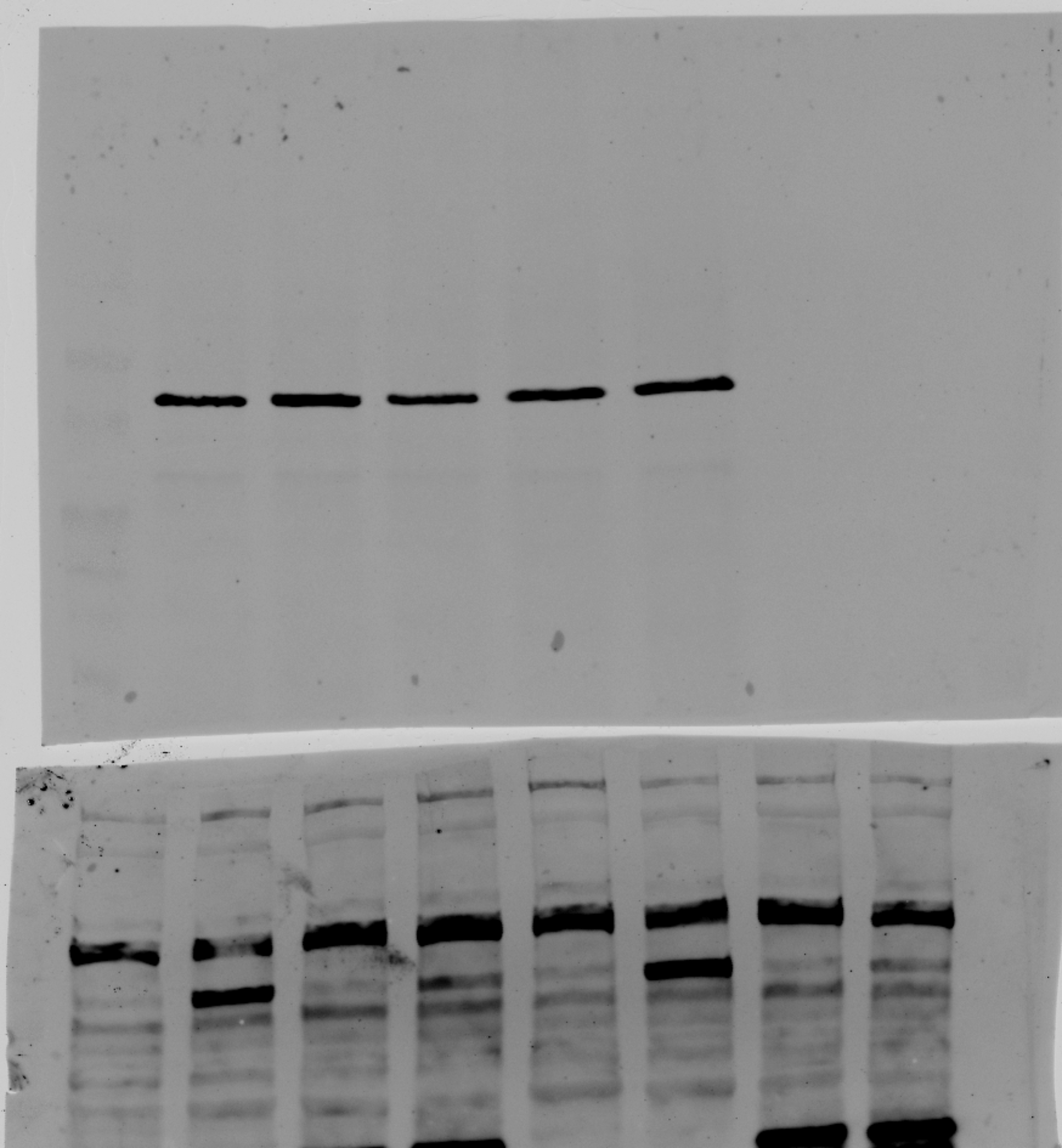

Supplement: Figure 3—figure supplement 1—source data 1. [file elife-82860-fig3-figsupp1-data1.zip › elife_Figure 3 Supp 1 source data/elife_Fig 3 Supp 1 source data 2/Fig_3_Supp_1C_Source_Data_Unlabeled/Fig_3_Supp_1C_Actin_Unlabeled.tif]

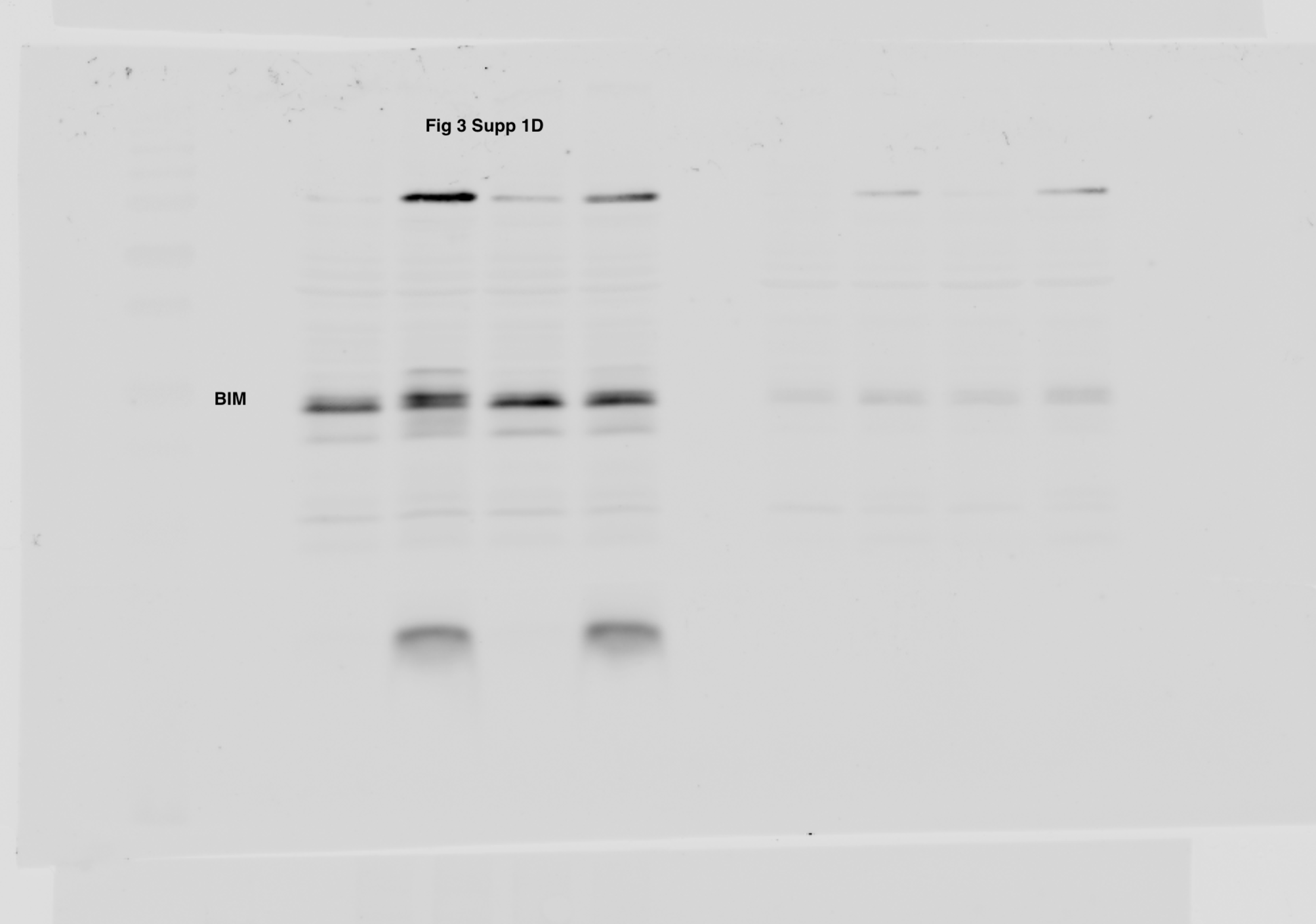

Supplement: Figure 3—figure supplement 1—source data 1. [file elife-82860-fig3-figsupp1-data1.zip › elife_Figure 3 Supp 1 source data/elife_Fig 3 Supp 1 source data 3/Fig_3_Supp_1D_Labeled/Fig_3_Supp_1D_BIM_labeled.tif]

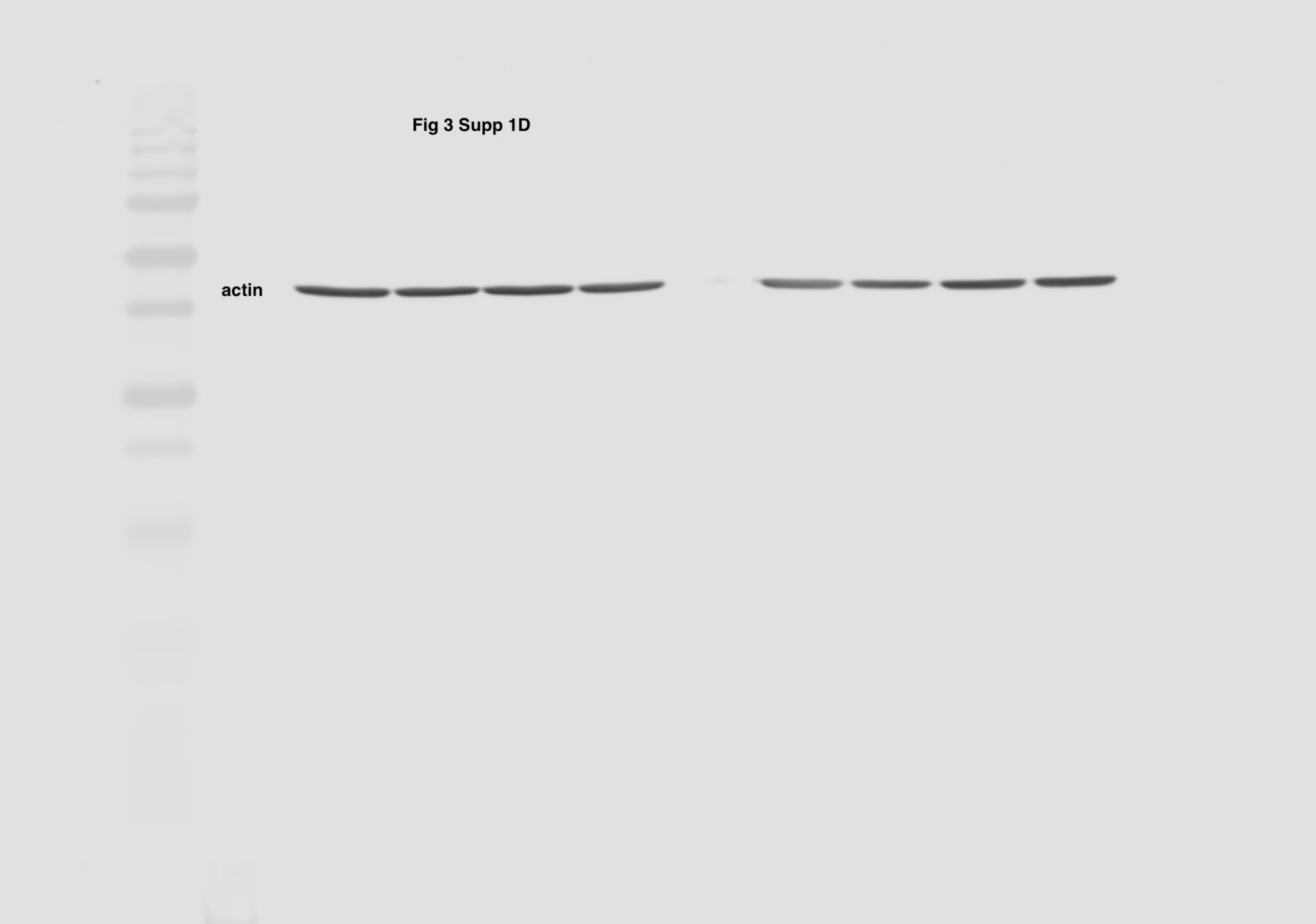

Supplement: Figure 3—figure supplement 1—source data 1. [file elife-82860-fig3-figsupp1-data1.zip › elife_Figure 3 Supp 1 source data/elife_Fig 3 Supp 1 source data 3/Fig_3_Supp_1D_Labeled/Fig_3_Supp_1C_actin_labeled.tif]

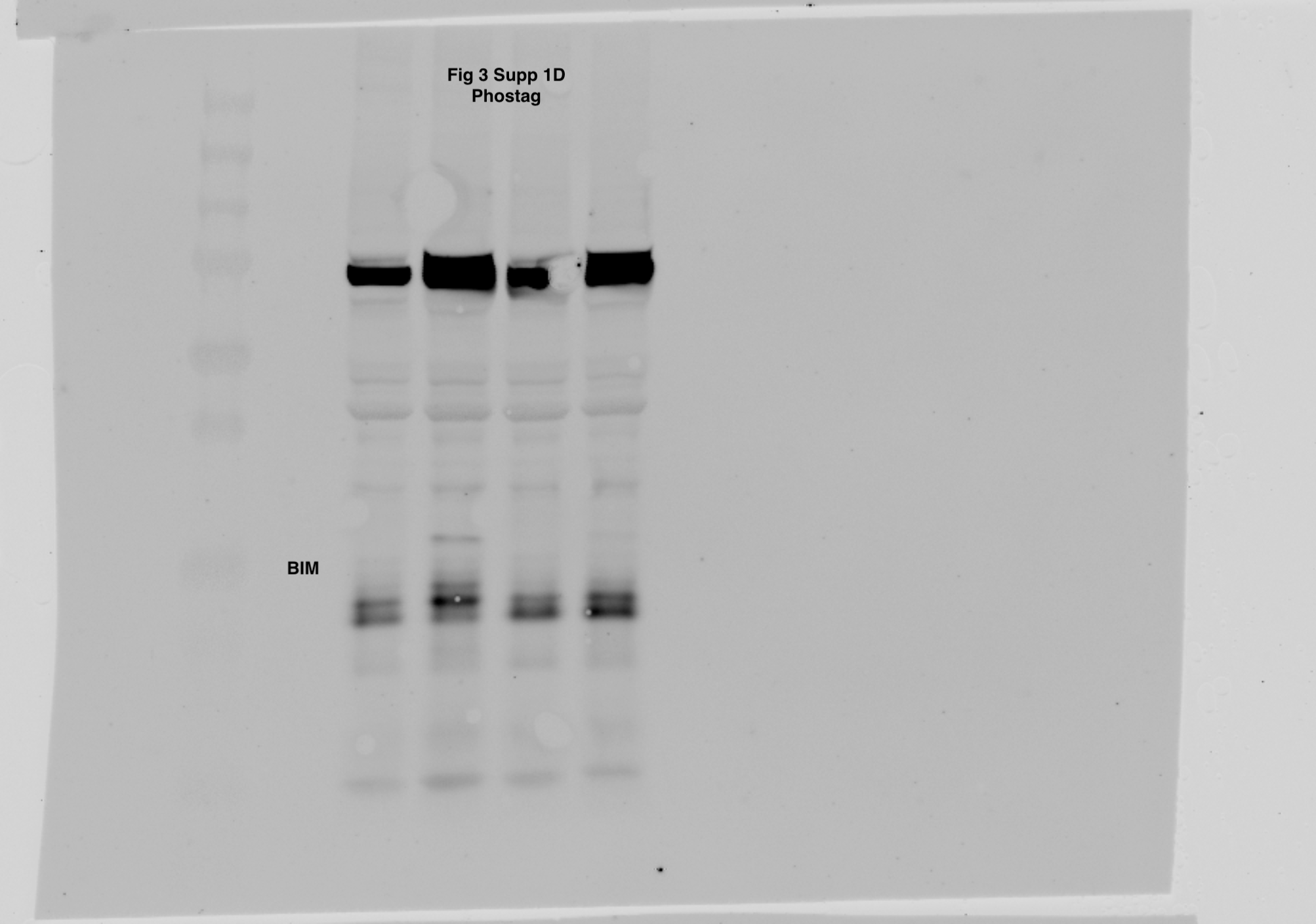

Supplement: Figure 3—figure supplement 1—source data 1. [file elife-82860-fig3-figsupp1-data1.zip › elife_Figure 3 Supp 1 source data/elife_Fig 3 Supp 1 source data 3/Fig_3_Supp_1D_Labeled/Fig_3_Supp_1C_Ptag_BIM_labeled.tif]

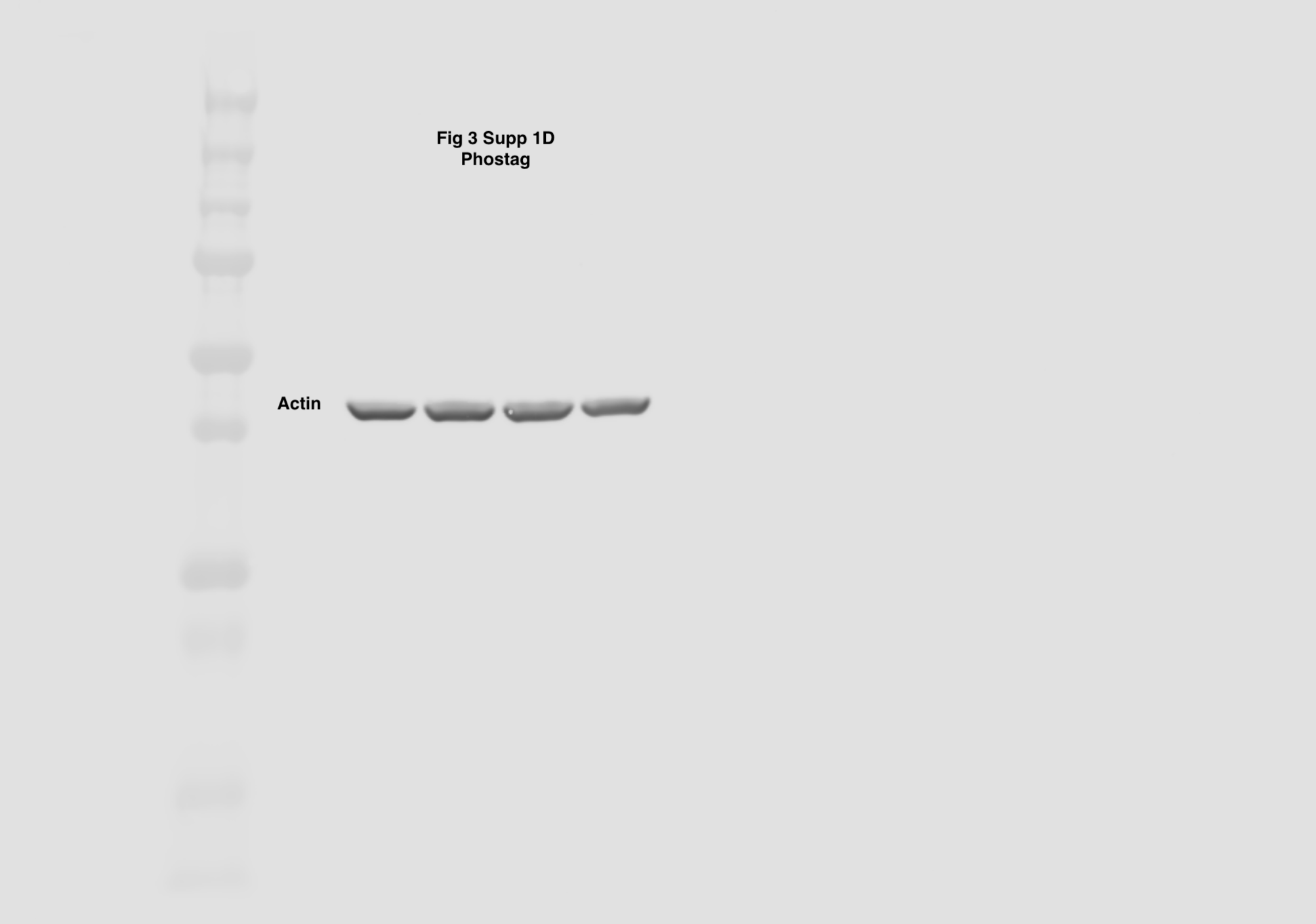

Supplement: Figure 3—figure supplement 1—source data 1. [file elife-82860-fig3-figsupp1-data1.zip › elife_Figure 3 Supp 1 source data/elife_Fig 3 Supp 1 source data 3/Fig_3_Supp_1D_Labeled/Fig_3_Supp_1D_Ptag_Actin_labeled.tif]

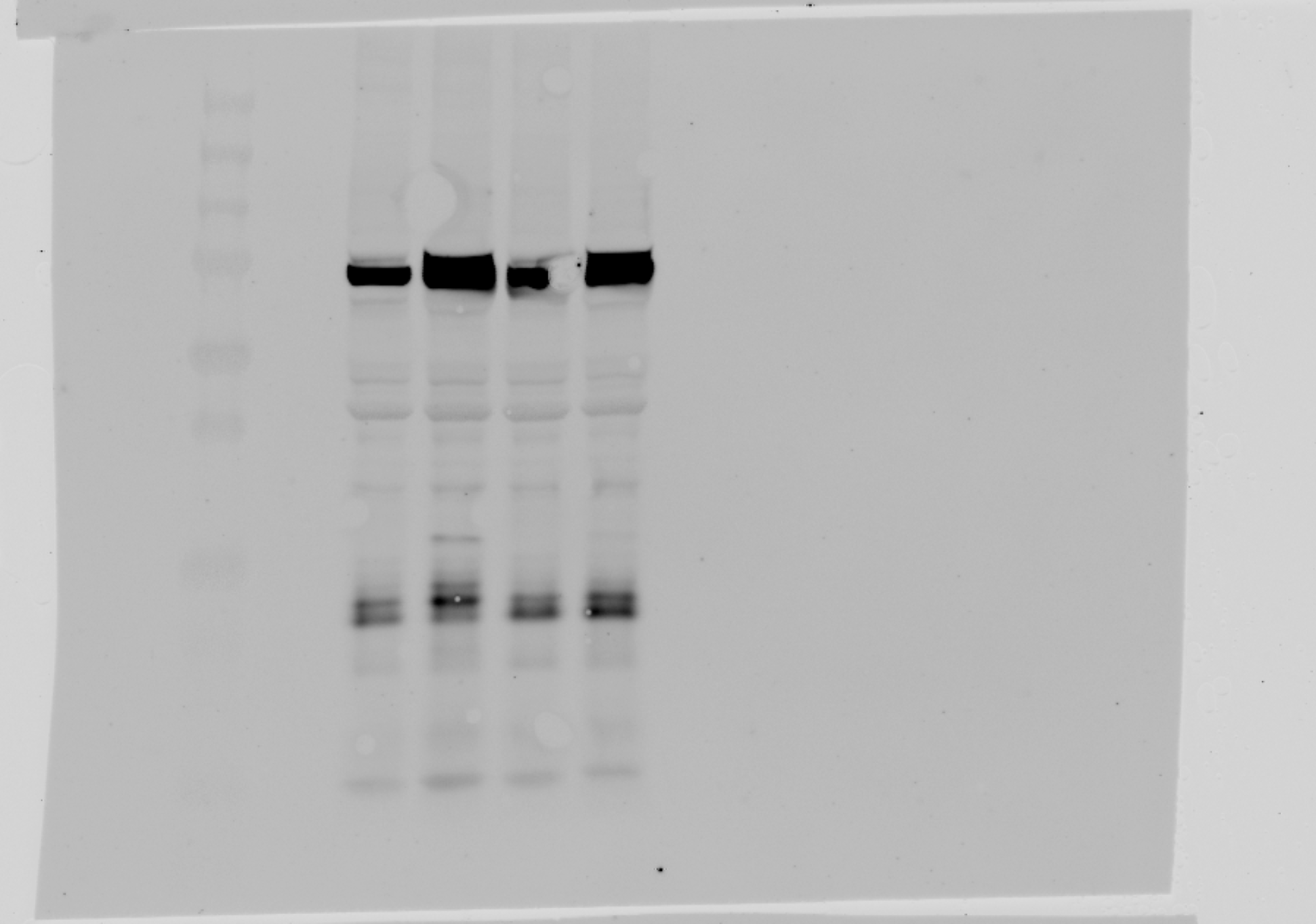

Supplement: Figure 3—figure supplement 1—source data 1. [file elife-82860-fig3-figsupp1-data1.zip › elife_Figure 3 Supp 1 source data/elife_Fig 3 Supp 1 source data 3/Fig_3_Supp_1D_Source_Data_Unlabeled/Fig_3_Supp_1C_Ptag_BIM_Unlabeled.tif]

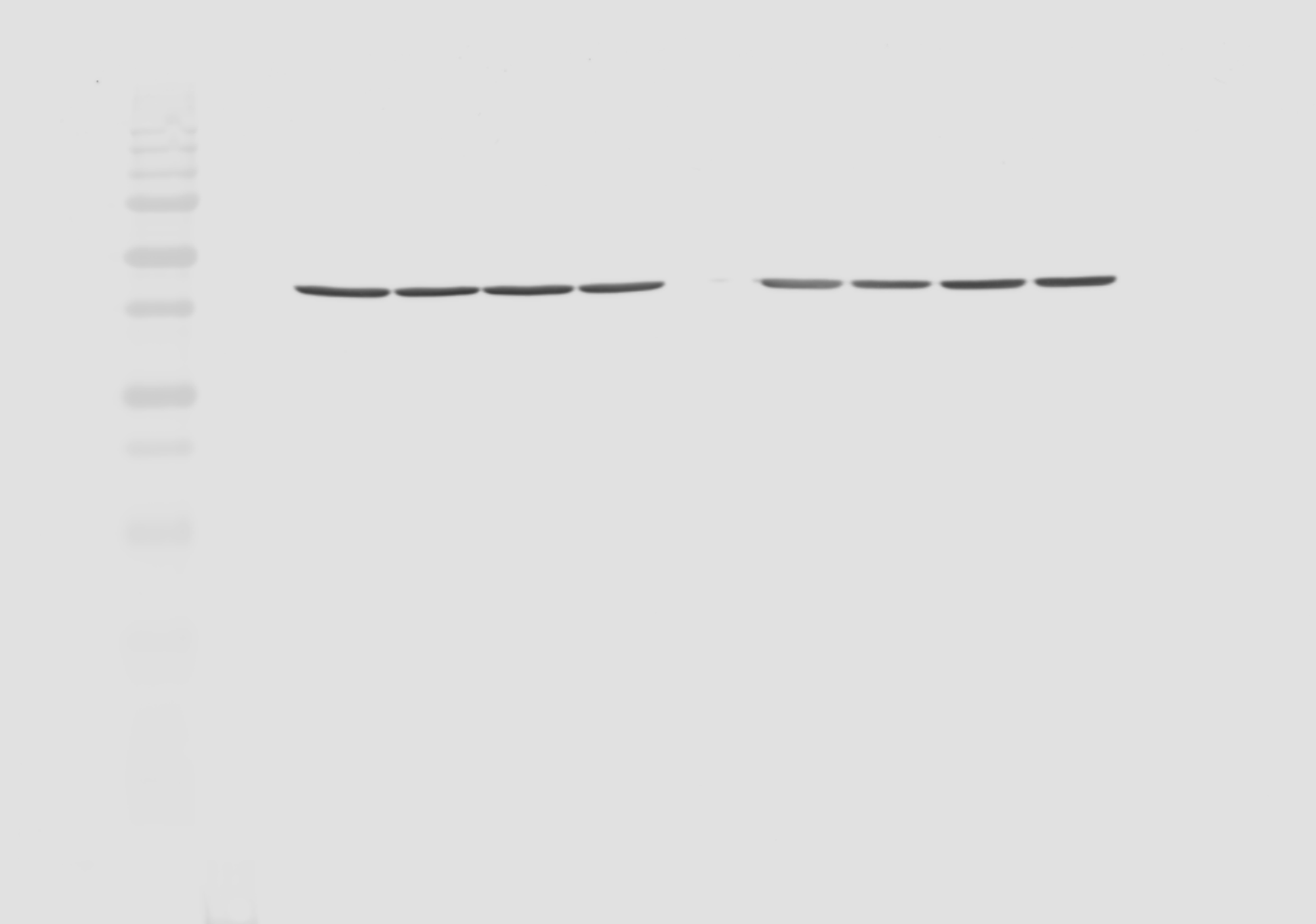

Supplement: Figure 3—figure supplement 1—source data 1. [file elife-82860-fig3-figsupp1-data1.zip › elife_Figure 3 Supp 1 source data/elife_Fig 3 Supp 1 source data 3/Fig_3_Supp_1D_Source_Data_Unlabeled/Fig_3_Supp_1C_actin_Unlabeled.tif]

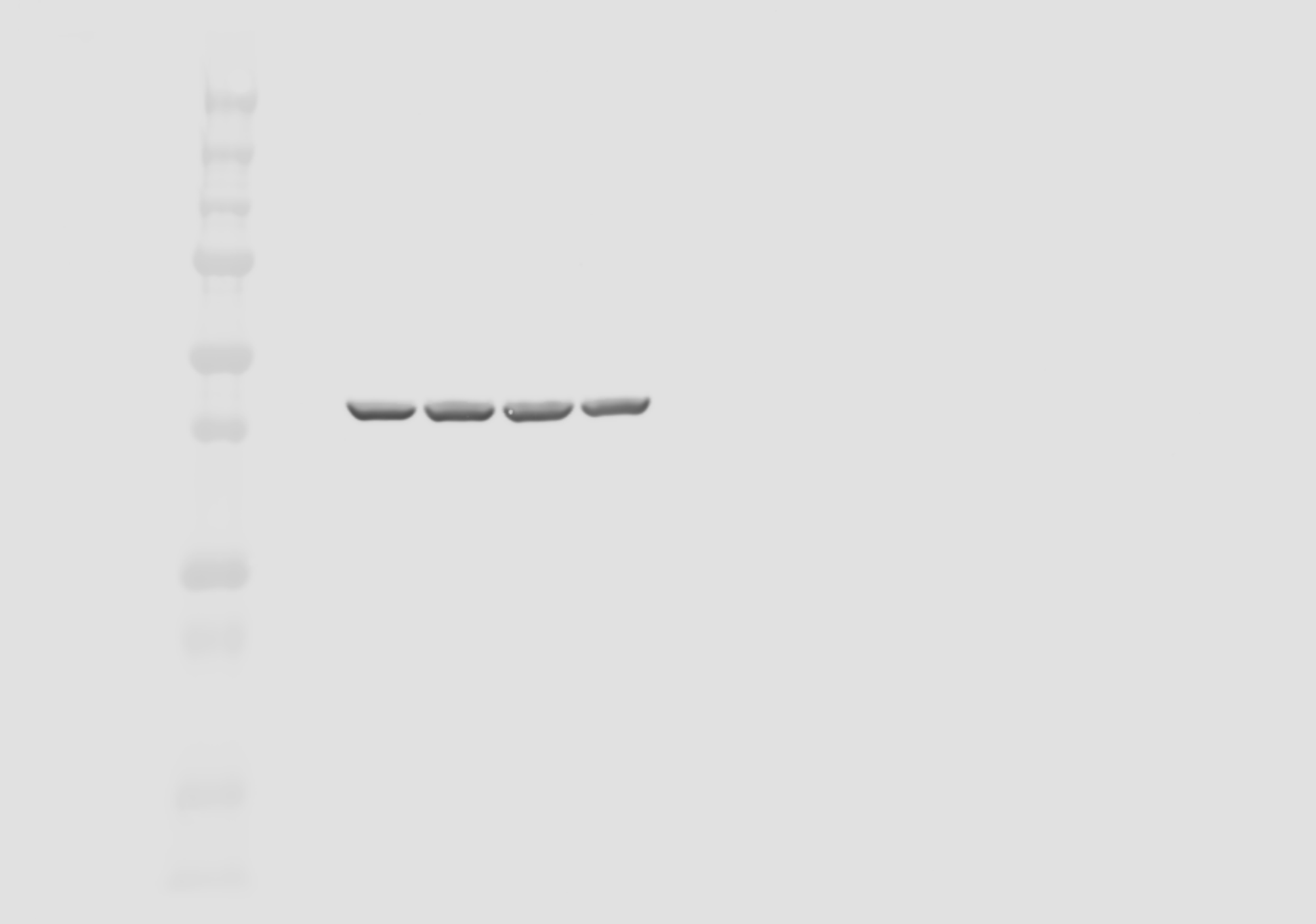

Supplement: Figure 3—figure supplement 1—source data 1. [file elife-82860-fig3-figsupp1-data1.zip › elife_Figure 3 Supp 1 source data/elife_Fig 3 Supp 1 source data 3/Fig_3_Supp_1D_Source_Data_Unlabeled/Fig_3_Supp_1D_Ptag_Actin_Unlabeled.tif]

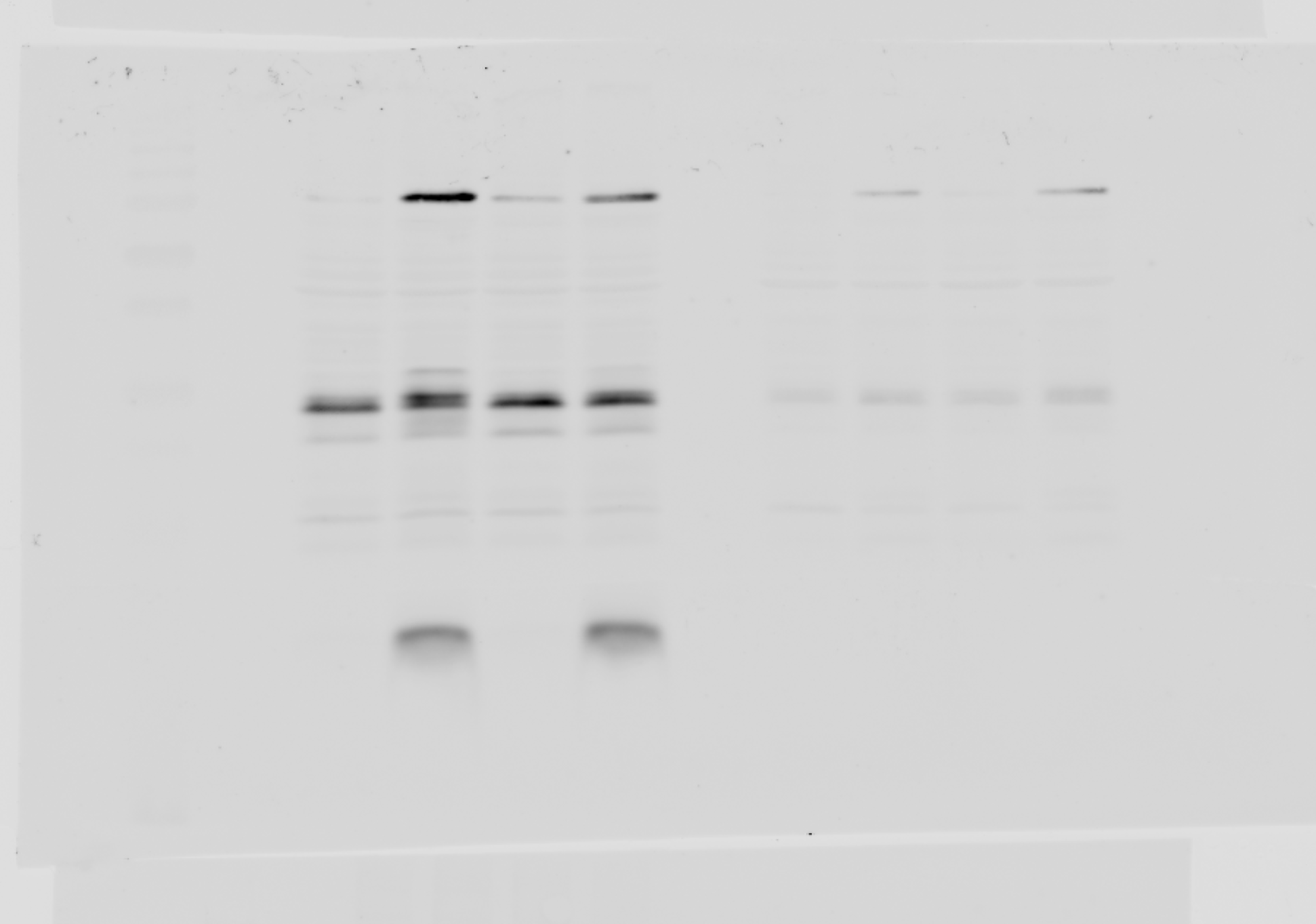

Supplement: Figure 3—figure supplement 1—source data 1. [file elife-82860-fig3-figsupp1-data1.zip › elife_Figure 3 Supp 1 source data/elife_Fig 3 Supp 1 source data 3/Fig_3_Supp_1D_Source_Data_Unlabeled/Fig_3_Supp_1D_BIM_Unlabeled.tif]

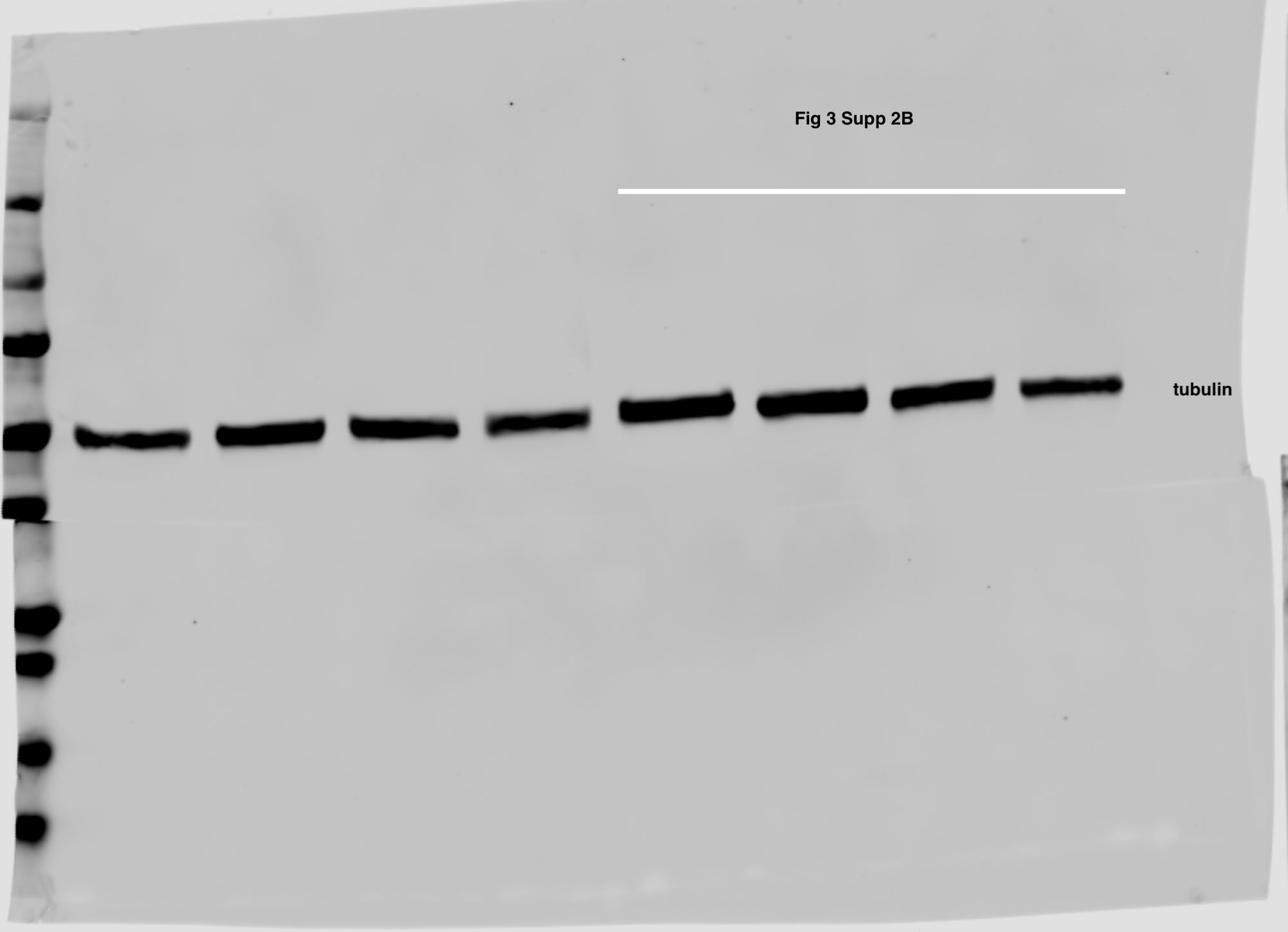

Supplement: Figure 3—figure supplement 3—source data 1. [file elife-82860-fig3-figsupp3-data1.zip › elife_Figure 3 Supp 2 source data/elife_Figure 3 Supp 2 source data 1/Fig_3_Supp_2_Source_Data_labeled/Fig_3_Supp_2B_Tubulin_labeled.tif]

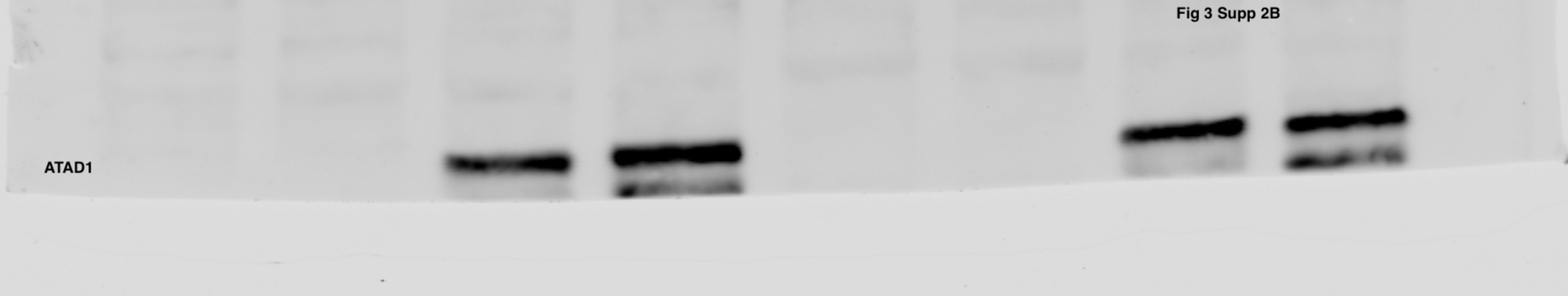

Supplement: Figure 3—figure supplement 3—source data 1. [file elife-82860-fig3-figsupp3-data1.zip › elife_Figure 3 Supp 2 source data/elife_Figure 3 Supp 2 source data 1/Fig_3_Supp_2_Source_Data_labeled/Fig_3_Supp_2B_ATAD1_labeled.tif]

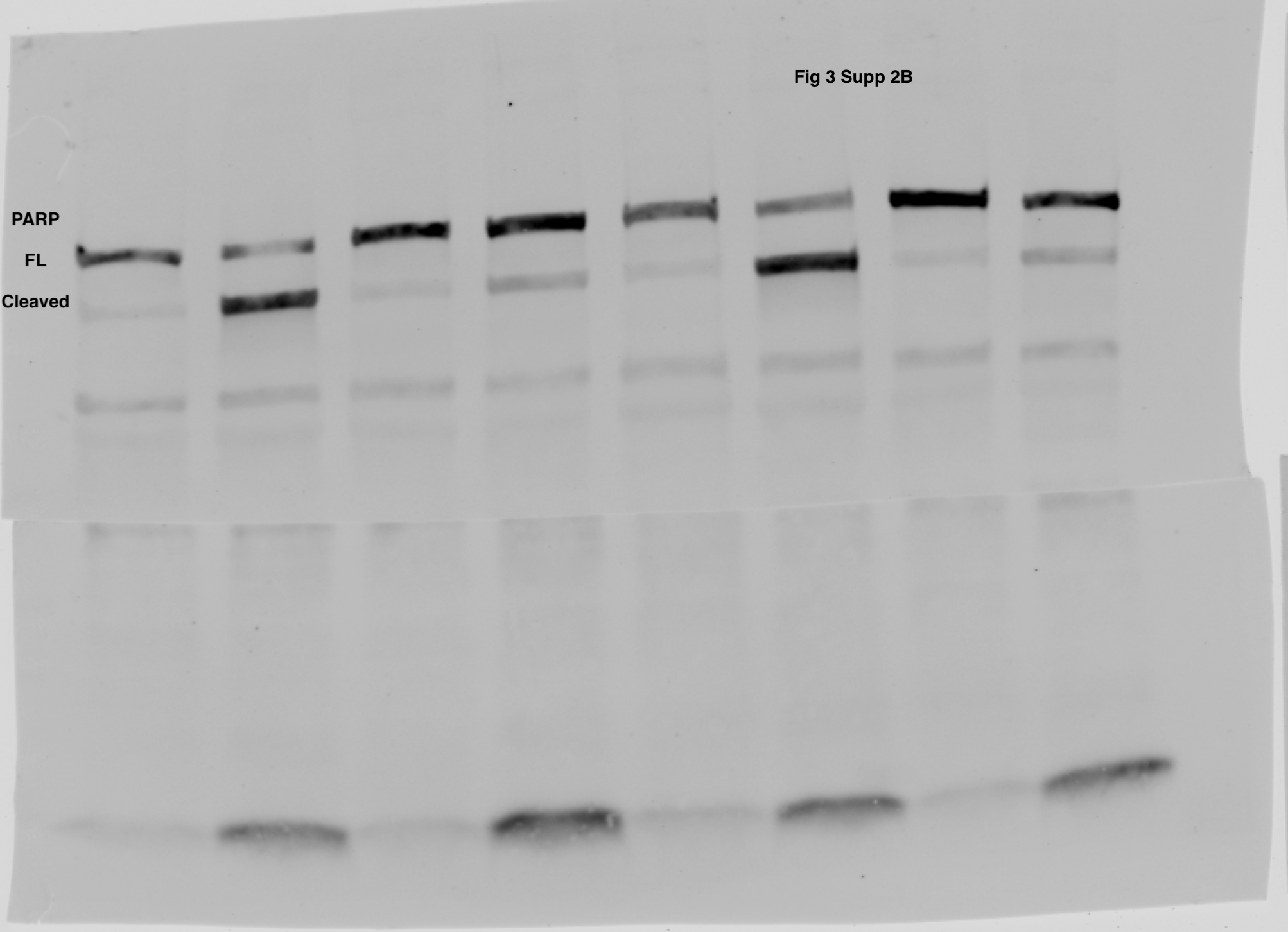

Supplement: Figure 3—figure supplement 3—source data 1. [file elife-82860-fig3-figsupp3-data1.zip › elife_Figure 3 Supp 2 source data/elife_Figure 3 Supp 2 source data 1/Fig_3_Supp_2_Source_Data_labeled/Fig_3_Supp_2B_PARP_labeled.tif]

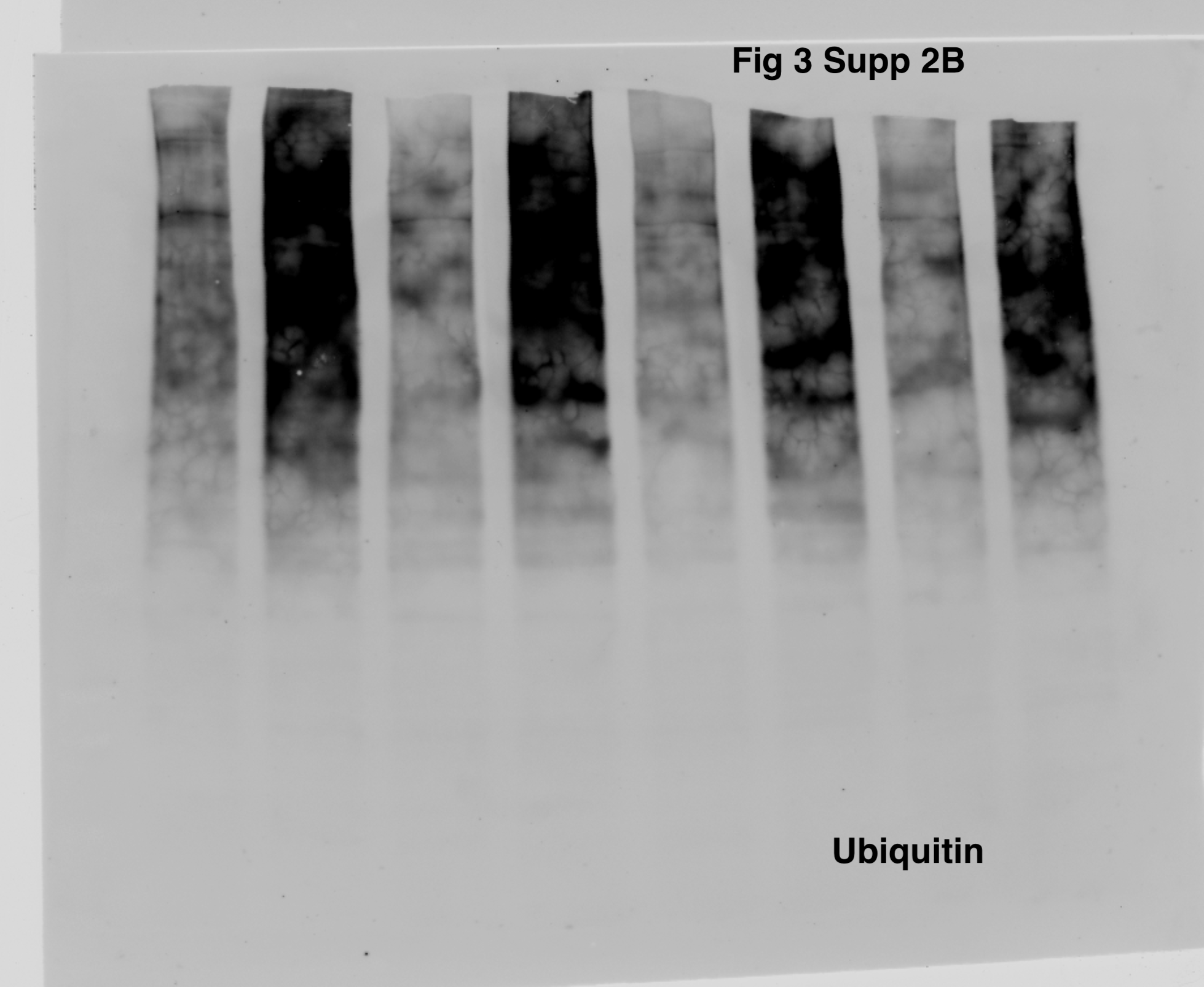

Supplement: Figure 3—figure supplement 3—source data 1. [file elife-82860-fig3-figsupp3-data1.zip › elife_Figure 3 Supp 2 source data/elife_Figure 3 Supp 2 source data 1/Fig_3_Supp_2_Source_Data_labeled/Fig_3_Supp_2B_Ub_labeled.tif]

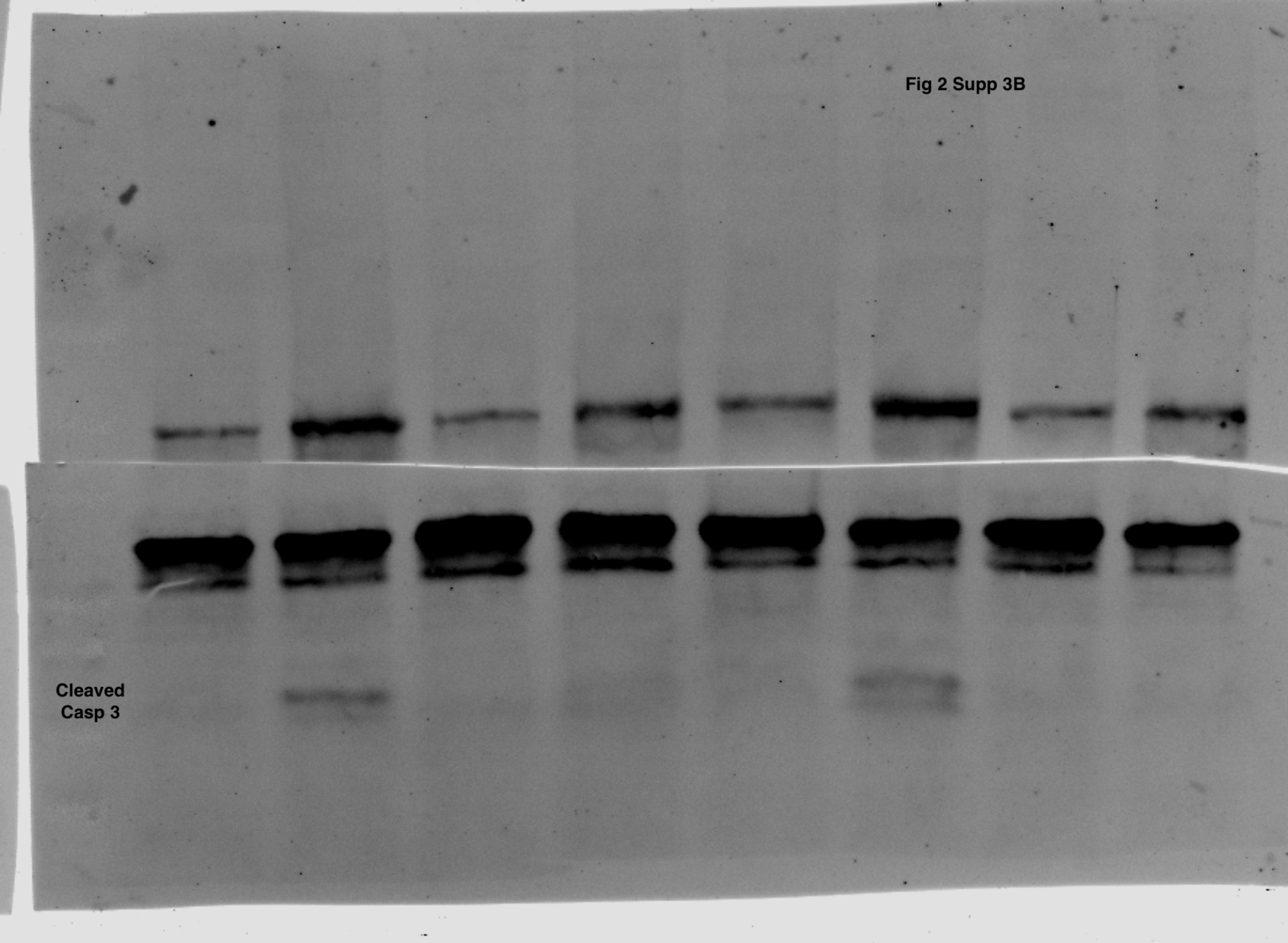

Supplement: Figure 3—figure supplement 3—source data 1. [file elife-82860-fig3-figsupp3-data1.zip › elife_Figure 3 Supp 2 source data/elife_Figure 3 Supp 2 source data 1/Fig_3_Supp_2_Source_Data_labeled/Fig_3_Supp_2B_Casp3_labeled.tif]

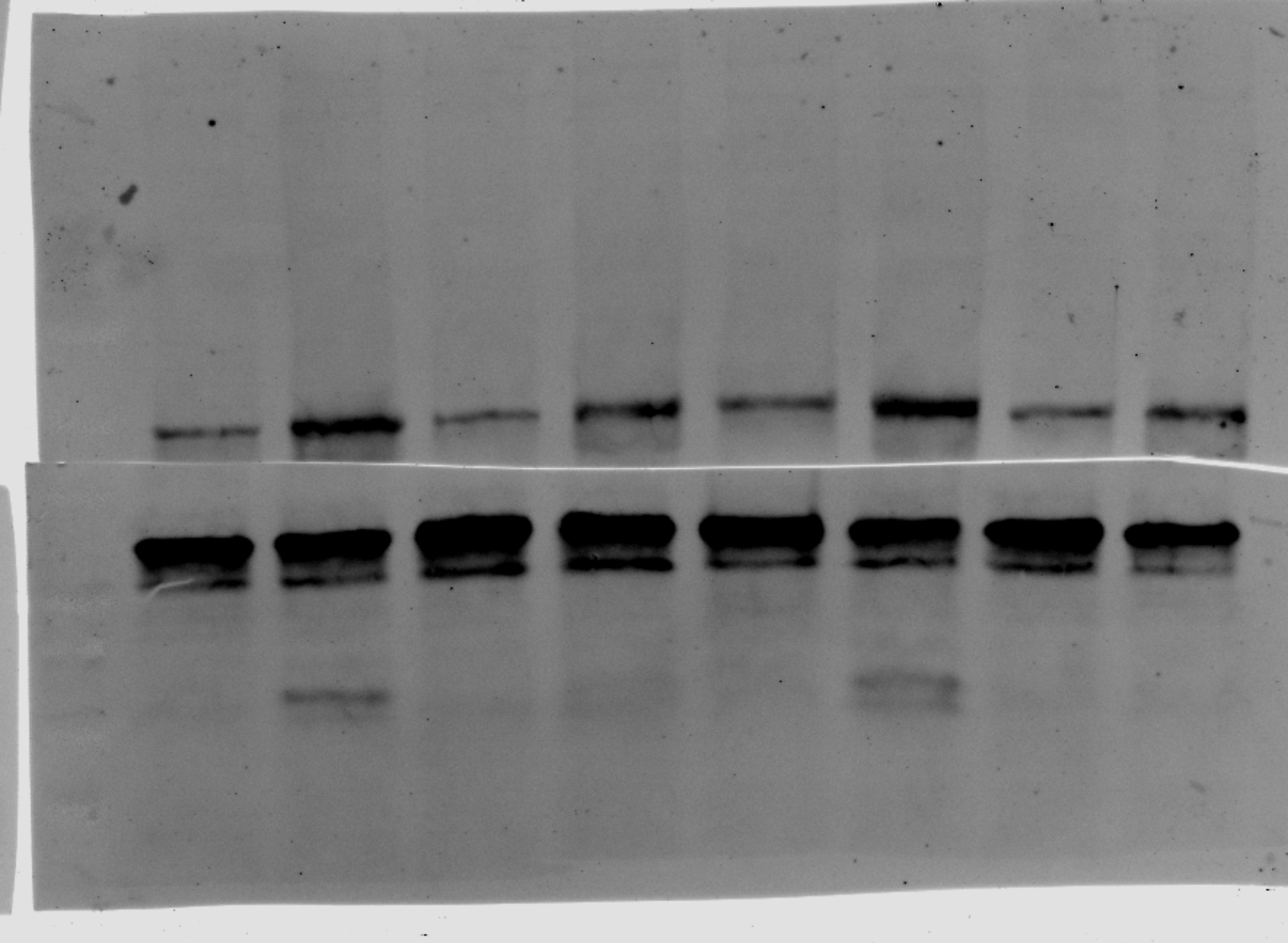

Supplement: Figure 3—figure supplement 3—source data 1. [file elife-82860-fig3-figsupp3-data1.zip › elife_Figure 3 Supp 2 source data/elife_Figure 3 Supp 2 source data 1/Fig_3_Supp_2_Source_Data_Unlabeled/Fig_3_Supp_2B_Casp3_Unlabeled.tif.tif]

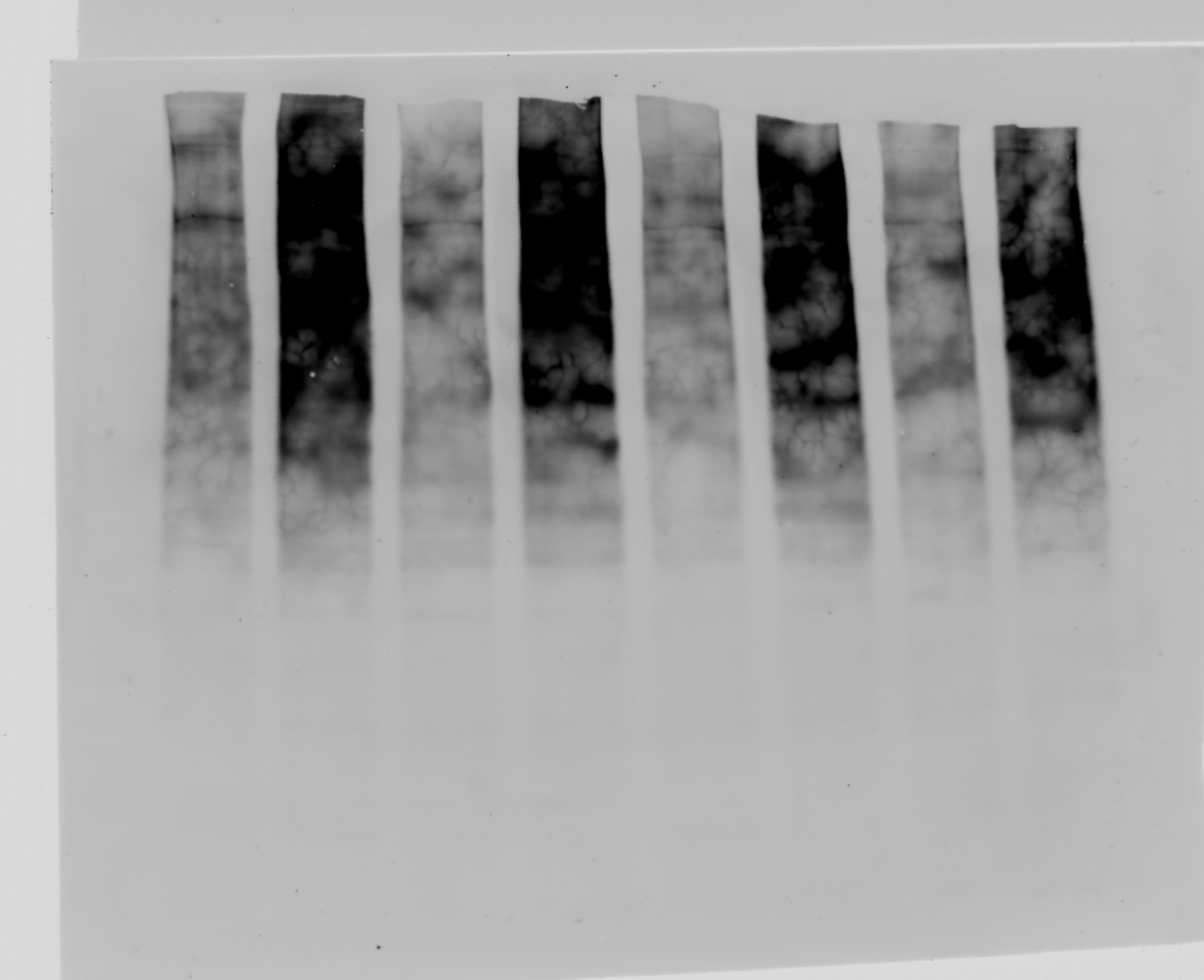

Supplement: Figure 3—figure supplement 3—source data 1. [file elife-82860-fig3-figsupp3-data1.zip › elife_Figure 3 Supp 2 source data/elife_Figure 3 Supp 2 source data 1/Fig_3_Supp_2_Source_Data_Unlabeled/Fig_3_Supp_2B_Ub_Unlabeled.tif]

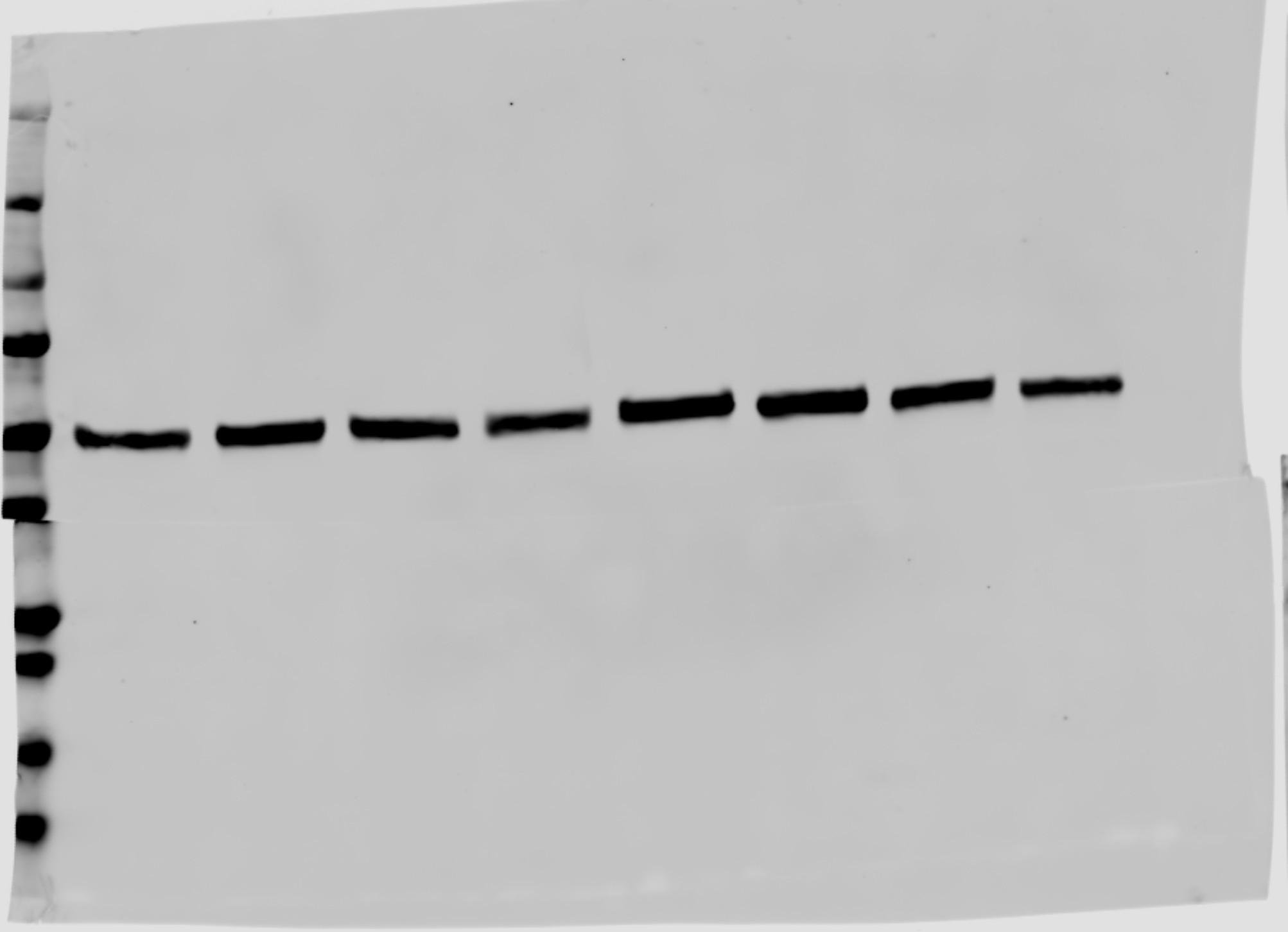

Supplement: Figure 3—figure supplement 3—source data 1. [file elife-82860-fig3-figsupp3-data1.zip › elife_Figure 3 Supp 2 source data/elife_Figure 3 Supp 2 source data 1/Fig_3_Supp_2_Source_Data_Unlabeled/Fig_3_Supp_2B_Tubulin_Unlabeled.tif]

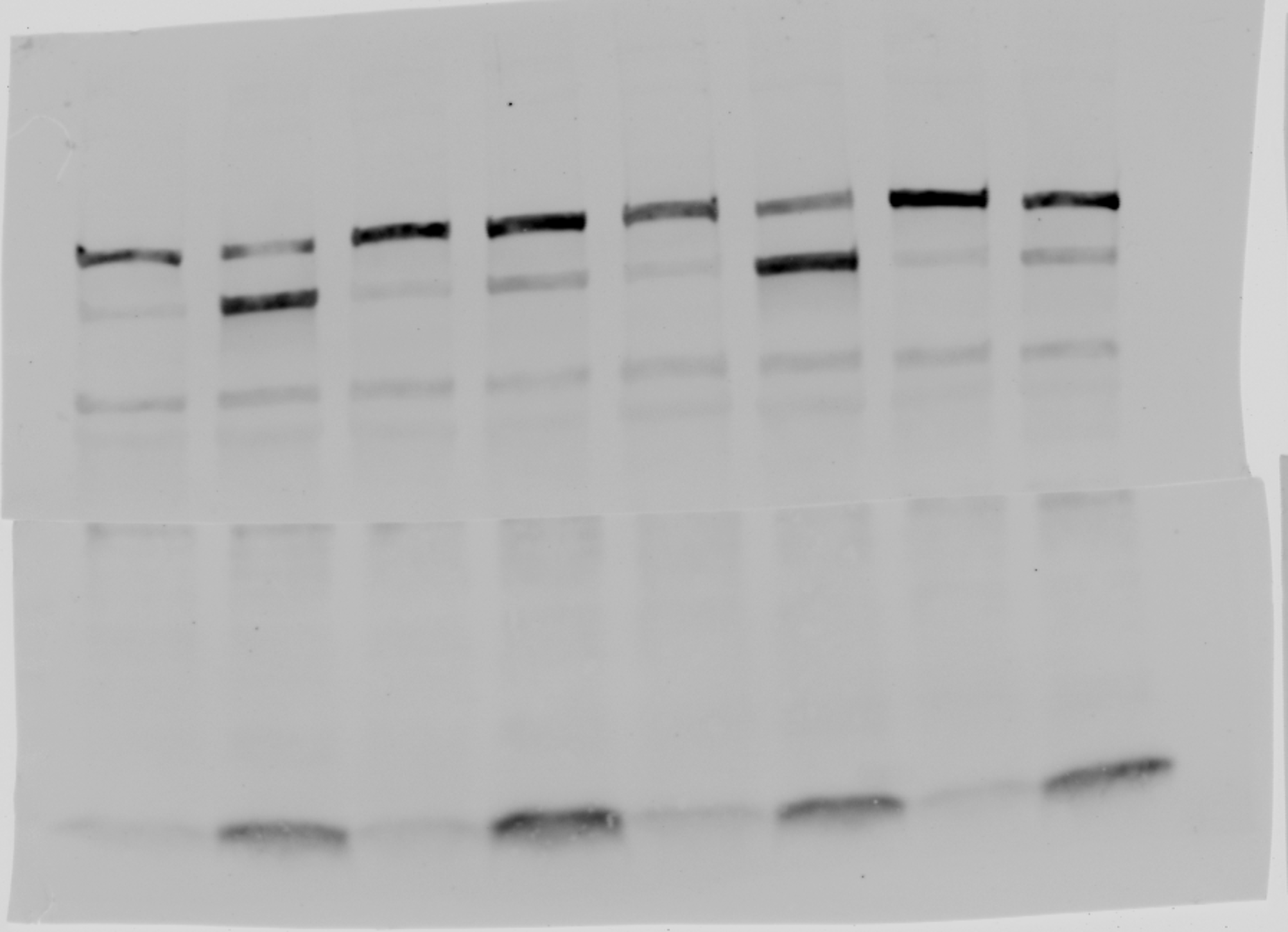

Supplement: Figure 3—figure supplement 3—source data 1. [file elife-82860-fig3-figsupp3-data1.zip › elife_Figure 3 Supp 2 source data/elife_Figure 3 Supp 2 source data 1/Fig_3_Supp_2_Source_Data_Unlabeled/Fig_3_Supp_2B_PARP_Unlabeled.tif]

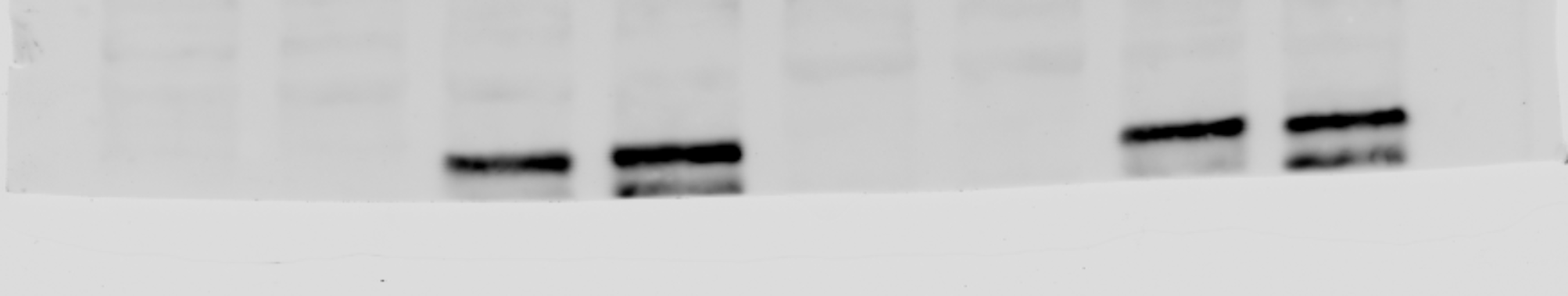

Supplement: Figure 3—figure supplement 3—source data 1. [file elife-82860-fig3-figsupp3-data1.zip › elife_Figure 3 Supp 2 source data/elife_Figure 3 Supp 2 source data 1/Fig_3_Supp_2_Source_Data_Unlabeled/Fig_3_Supp_2B_ATAD1_unlabeled.tif]

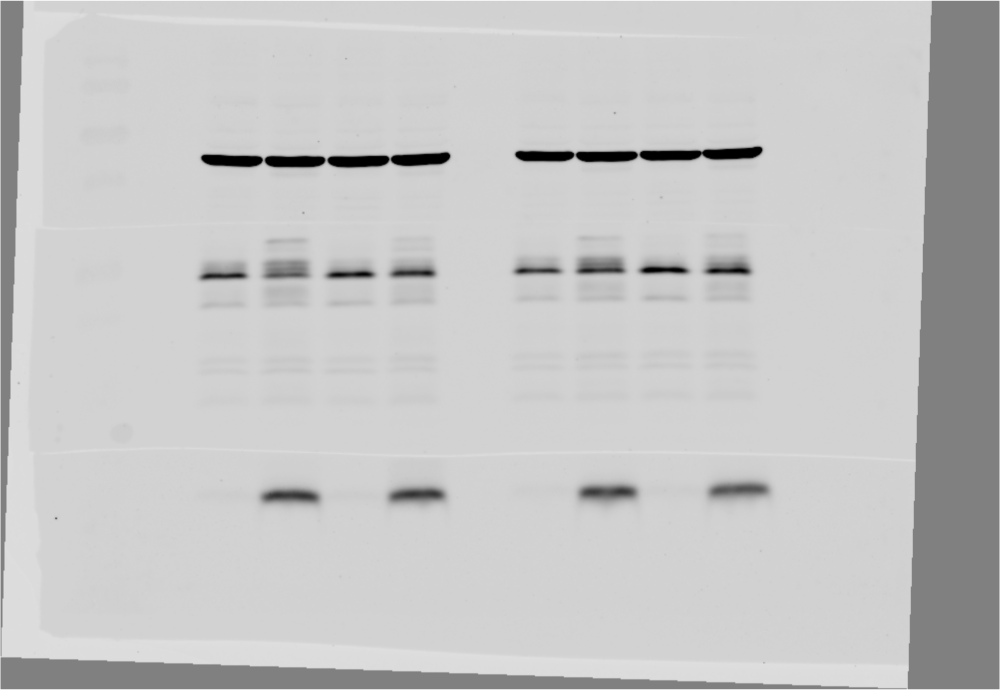

Supplement: Figure 3—figure supplement 4—source data 1. [file elife-82860-fig3-figsupp4-data1.zip › elife_Figure 3 Supp 3 source data/elife_Fig 3 Supp 3 source data 2/Fig_3_Supp_3C_Unlabeled/Fig_3_Supp_3C_BIM_NOXA_Unlabeled.tif]

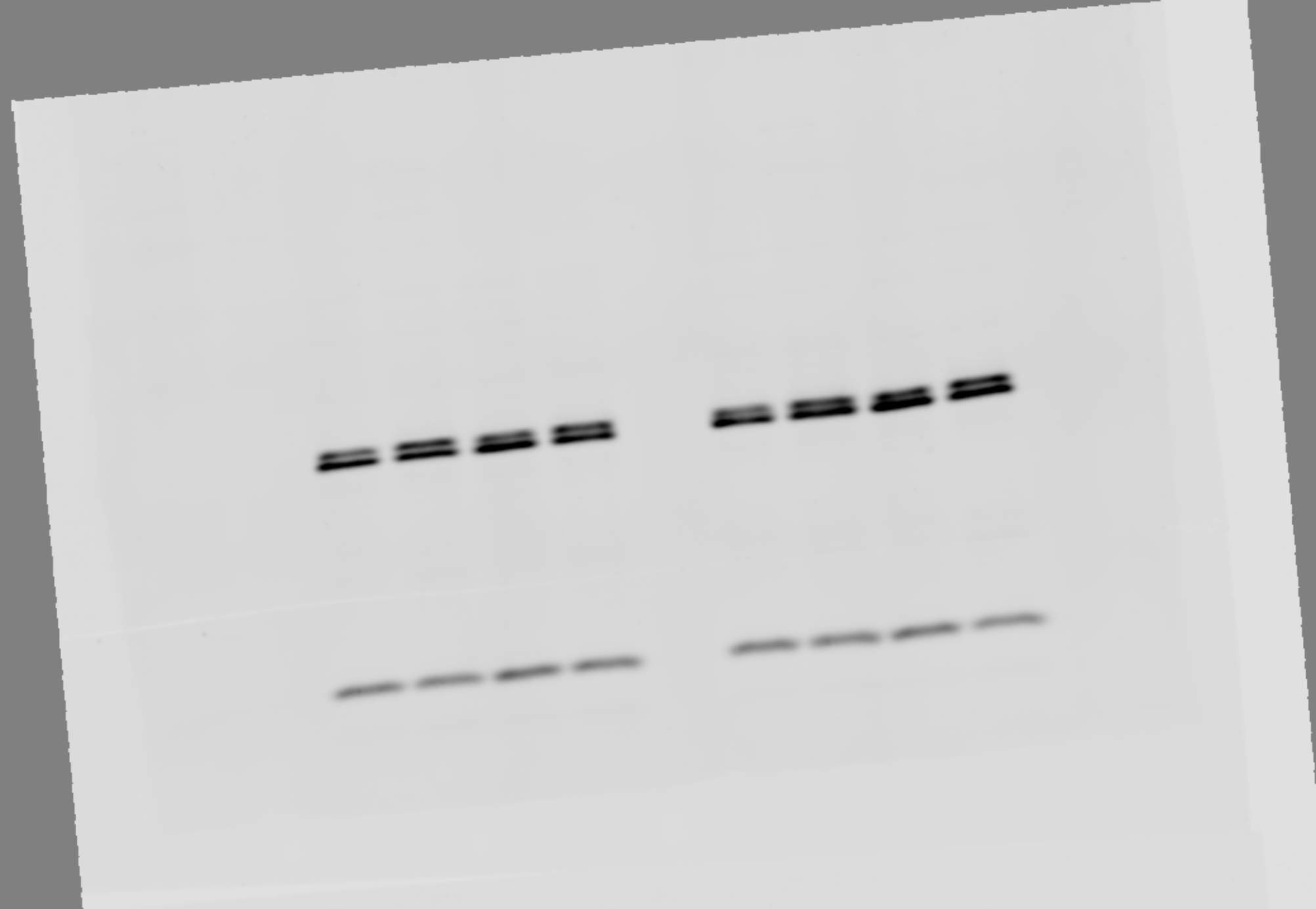

Supplement: Figure 3—figure supplement 4—source data 1. [file elife-82860-fig3-figsupp4-data1.zip › elife_Figure 3 Supp 3 source data/elife_Fig 3 Supp 3 source data 2/Fig_3_Supp_3C_Unlabeled/Fig_3_Supp_3C_BCLXL_FIS1_Unlabeled.tif]

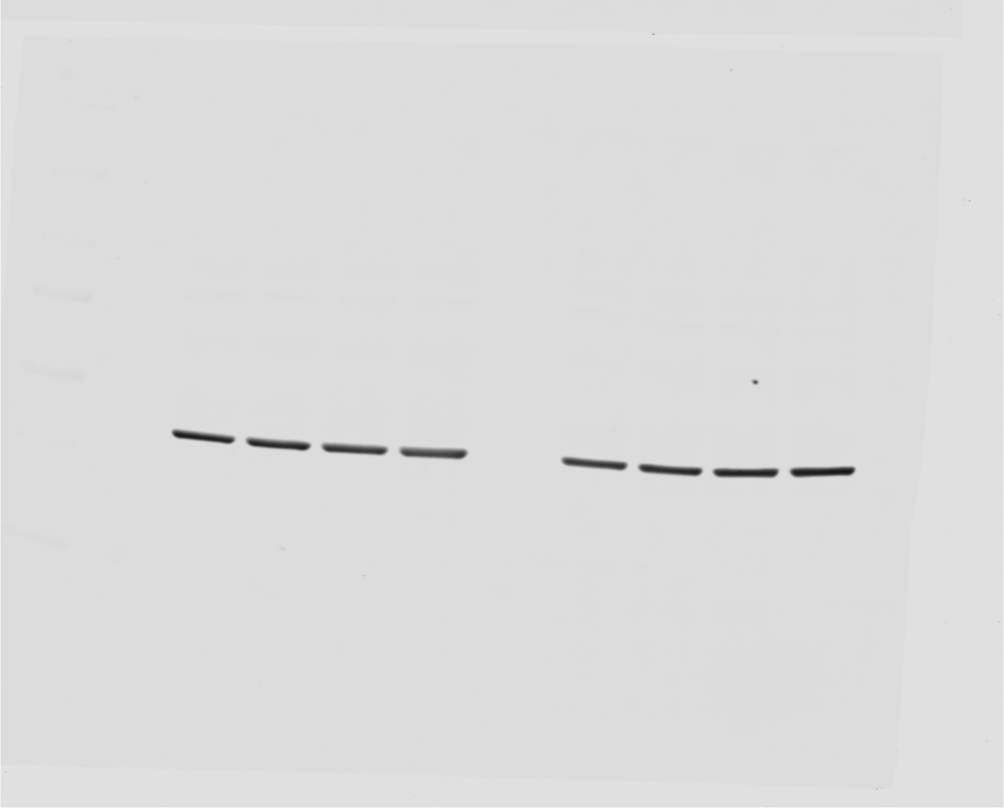

Supplement: Figure 3—figure supplement 4—source data 1. [file elife-82860-fig3-figsupp4-data1.zip › elife_Figure 3 Supp 3 source data/elife_Fig 3 Supp 3 source data 2/Fig_3_Supp_3C_Unlabeled/Fig_3_Supp_3C_Actin_Unlabeled.tif]

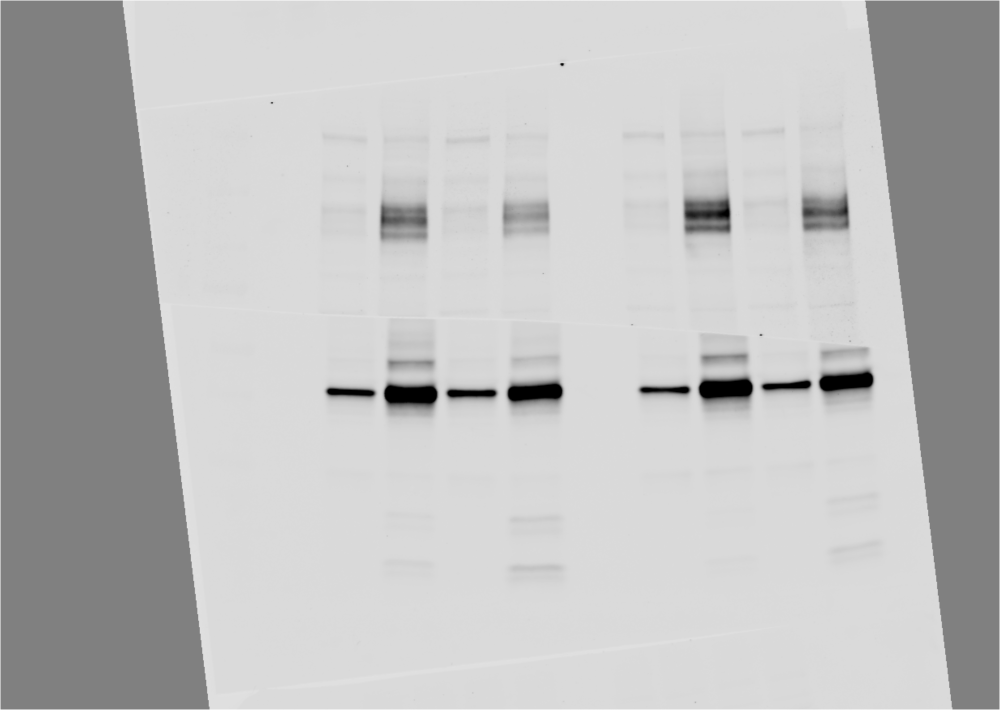

Supplement: Figure 3—figure supplement 4—source data 1. [file elife-82860-fig3-figsupp4-data1.zip › elife_Figure 3 Supp 3 source data/elife_Fig 3 Supp 3 source data 2/Fig_3_Supp_3C_Unlabeled/Fig_3_Supp_3C_NRF1_MCL1_Unlabeled.tif]

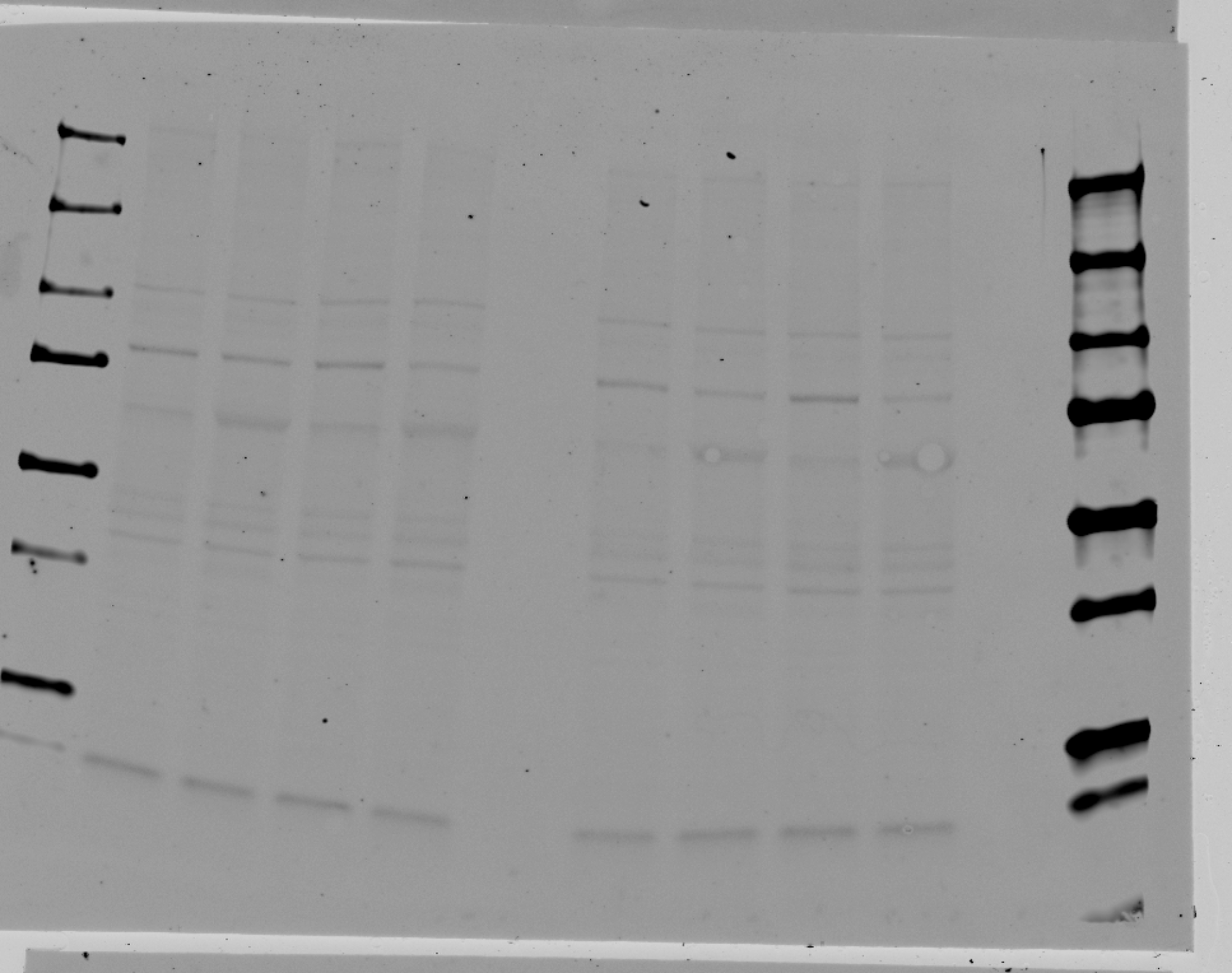

Supplement: Figure 3—figure supplement 4—source data 1. [file elife-82860-fig3-figsupp4-data1.zip › elife_Figure 3 Supp 3 source data/elife_Fig 3 Supp 3 source data 2/Fig_3_Supp_3C_Unlabeled/Fig_3_Supp_3C_BID_Unlabeled.tif.tif]

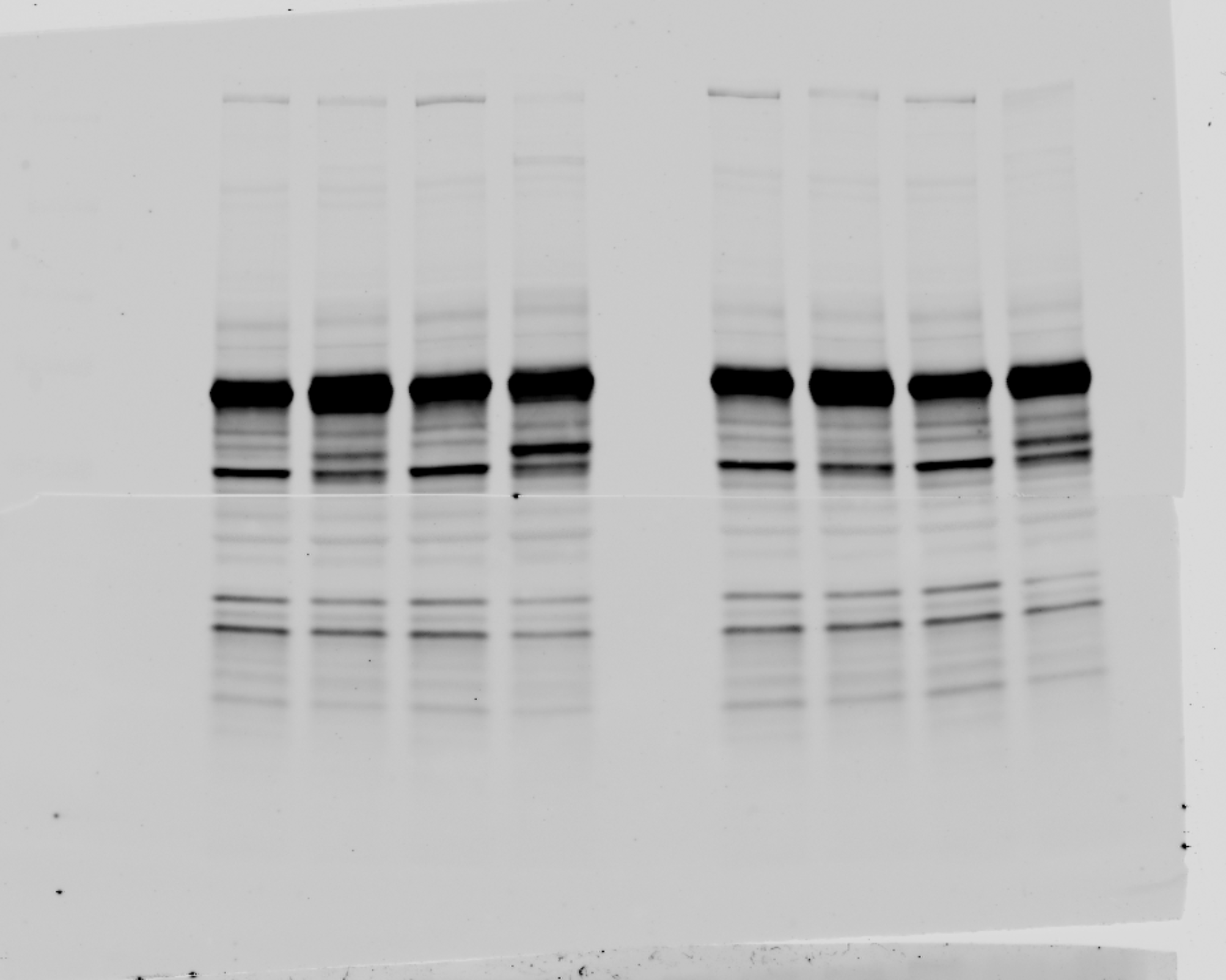

Supplement: Figure 3—figure supplement 4—source data 1. [file elife-82860-fig3-figsupp4-data1.zip › elife_Figure 3 Supp 3 source data/elife_Fig 3 Supp 3 source data 2/Fig_3_Supp_3C_Unlabeled/Fig_3_Supp_3C_MAVS_MFF_Unlabeled.tif]

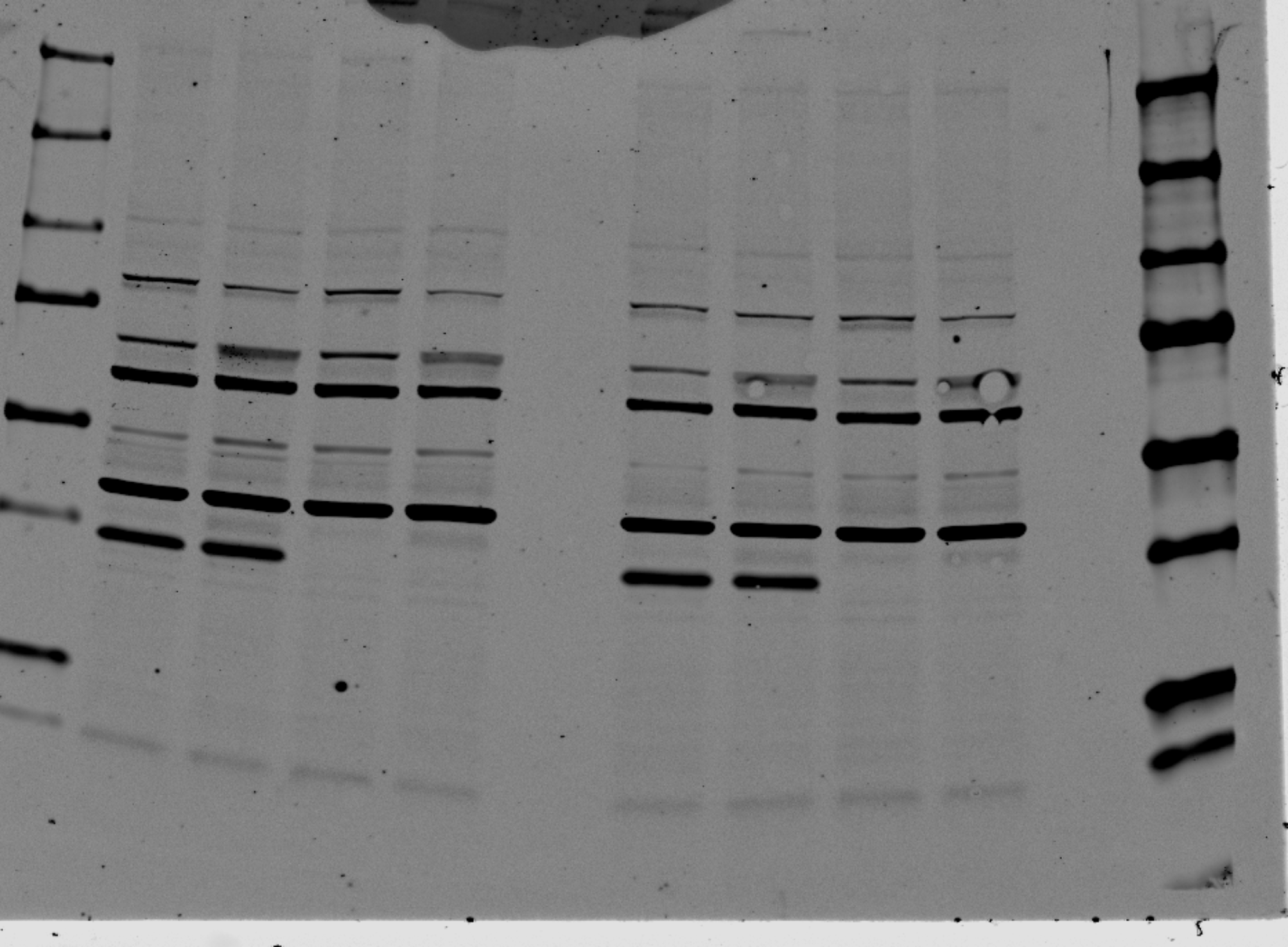

Supplement: Figure 3—figure supplement 4—source data 1. [file elife-82860-fig3-figsupp4-data1.zip › elife_Figure 3 Supp 3 source data/elife_Fig 3 Supp 3 source data 2/Fig_3_Supp_3C_Unlabeled/Fig_3_Supp_3C_ATAD1_Unlabeled.tif]

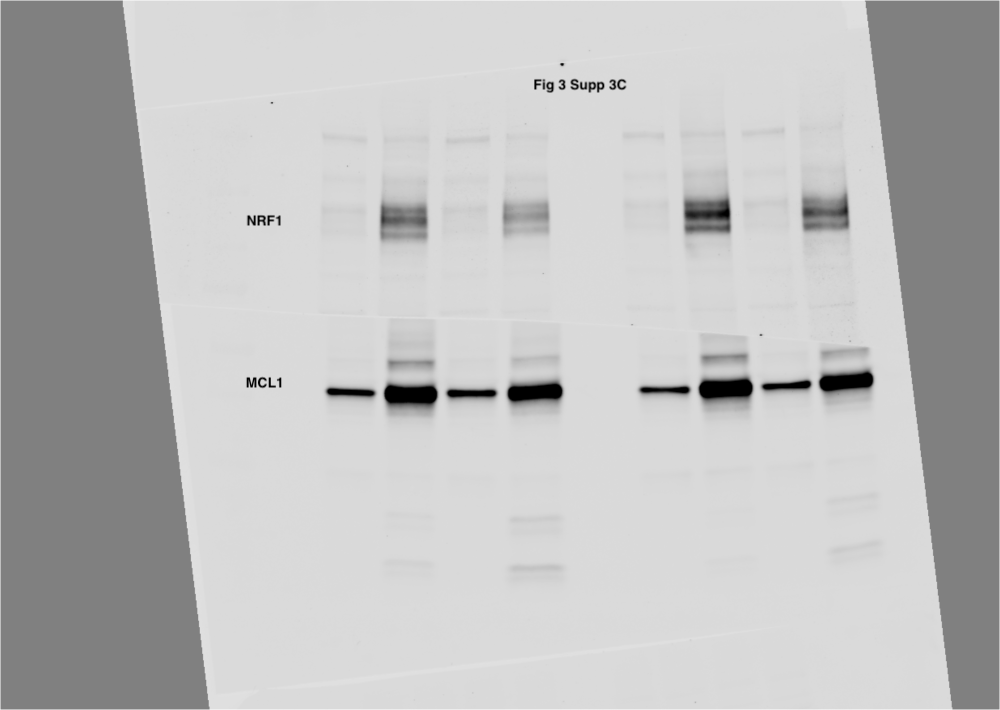

Supplement: Figure 3—figure supplement 4—source data 1. [file elife-82860-fig3-figsupp4-data1.zip › elife_Figure 3 Supp 3 source data/elife_Fig 3 Supp 3 source data 2/Fig_3_Supp_3C_labeled/Fig_3_Supp_3C_NRF1_MCL1_labeled.tif]

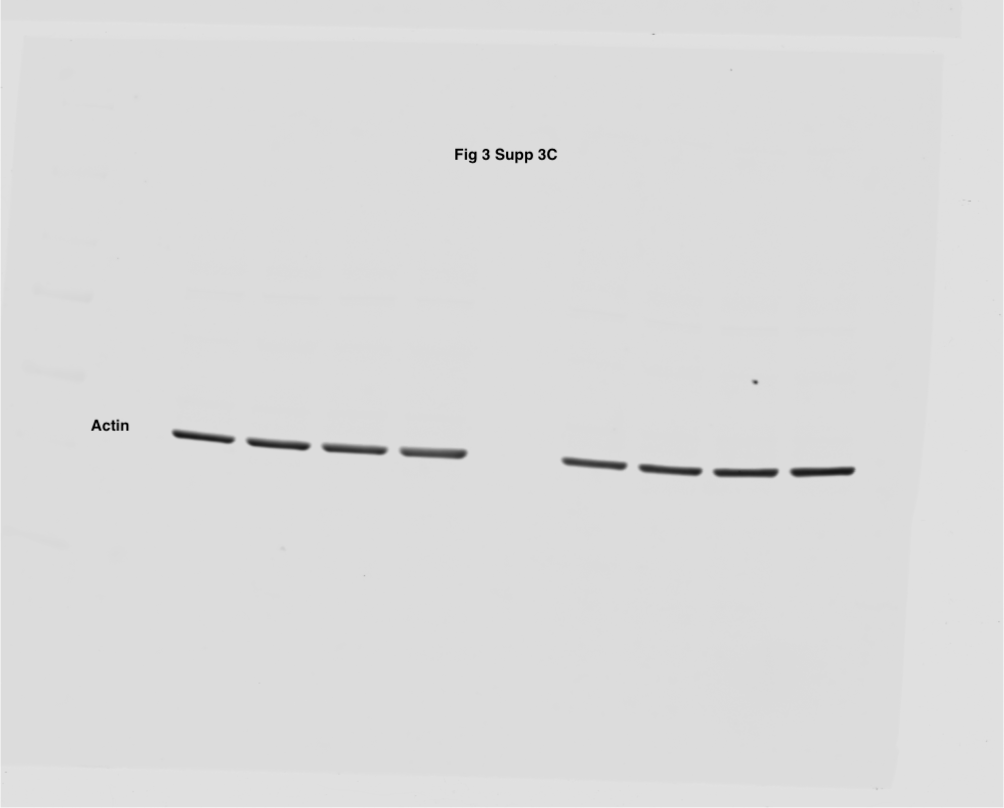

Supplement: Figure 3—figure supplement 4—source data 1. [file elife-82860-fig3-figsupp4-data1.zip › elife_Figure 3 Supp 3 source data/elife_Fig 3 Supp 3 source data 2/Fig_3_Supp_3C_labeled/Fig_3_Supp_3C_Actin_labeled.tif]

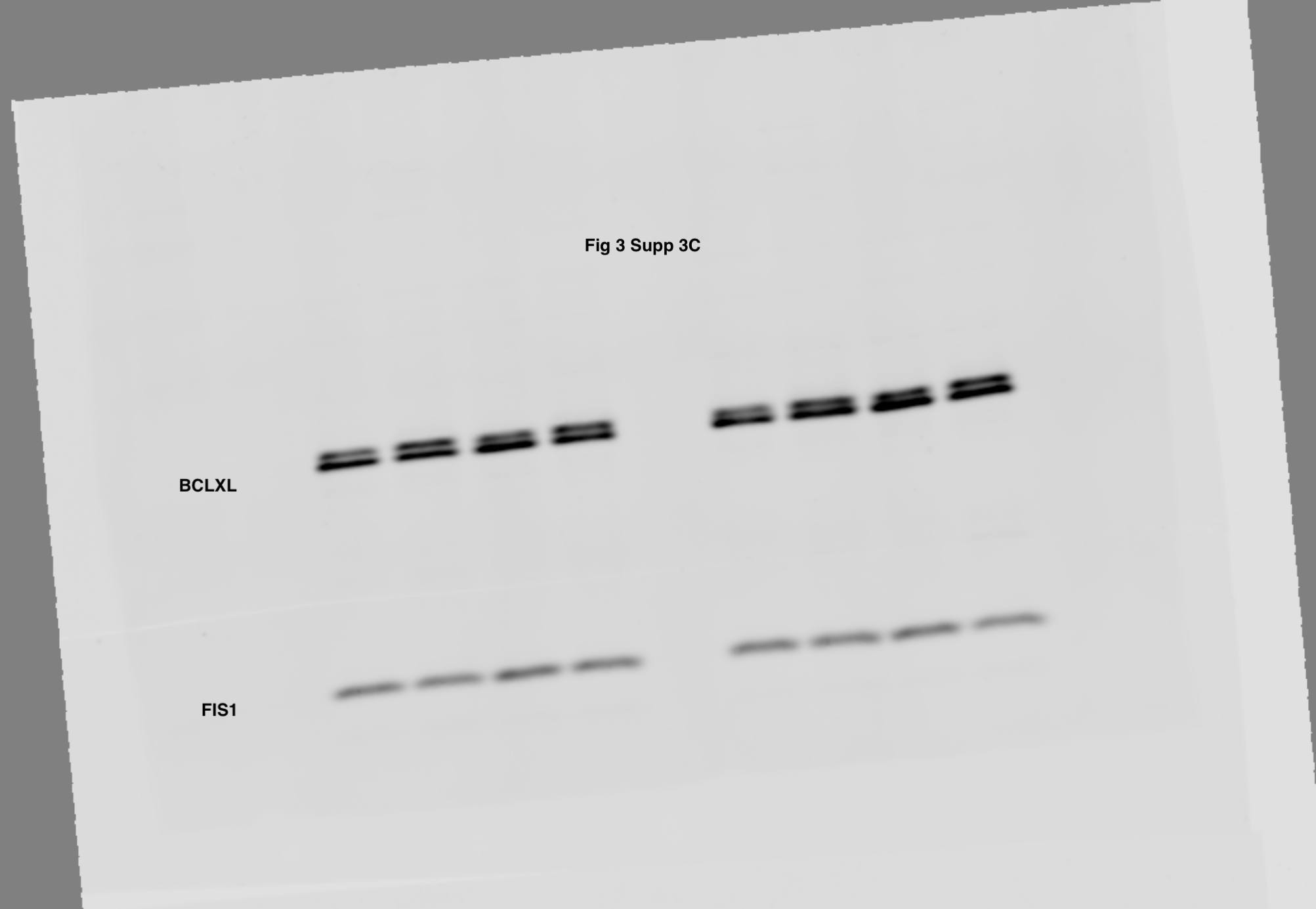

Supplement: Figure 3—figure supplement 4—source data 1. [file elife-82860-fig3-figsupp4-data1.zip › elife_Figure 3 Supp 3 source data/elife_Fig 3 Supp 3 source data 2/Fig_3_Supp_3C_labeled/Fig_3_Supp_3C_BCLXL_FIS1_labeled.tif]

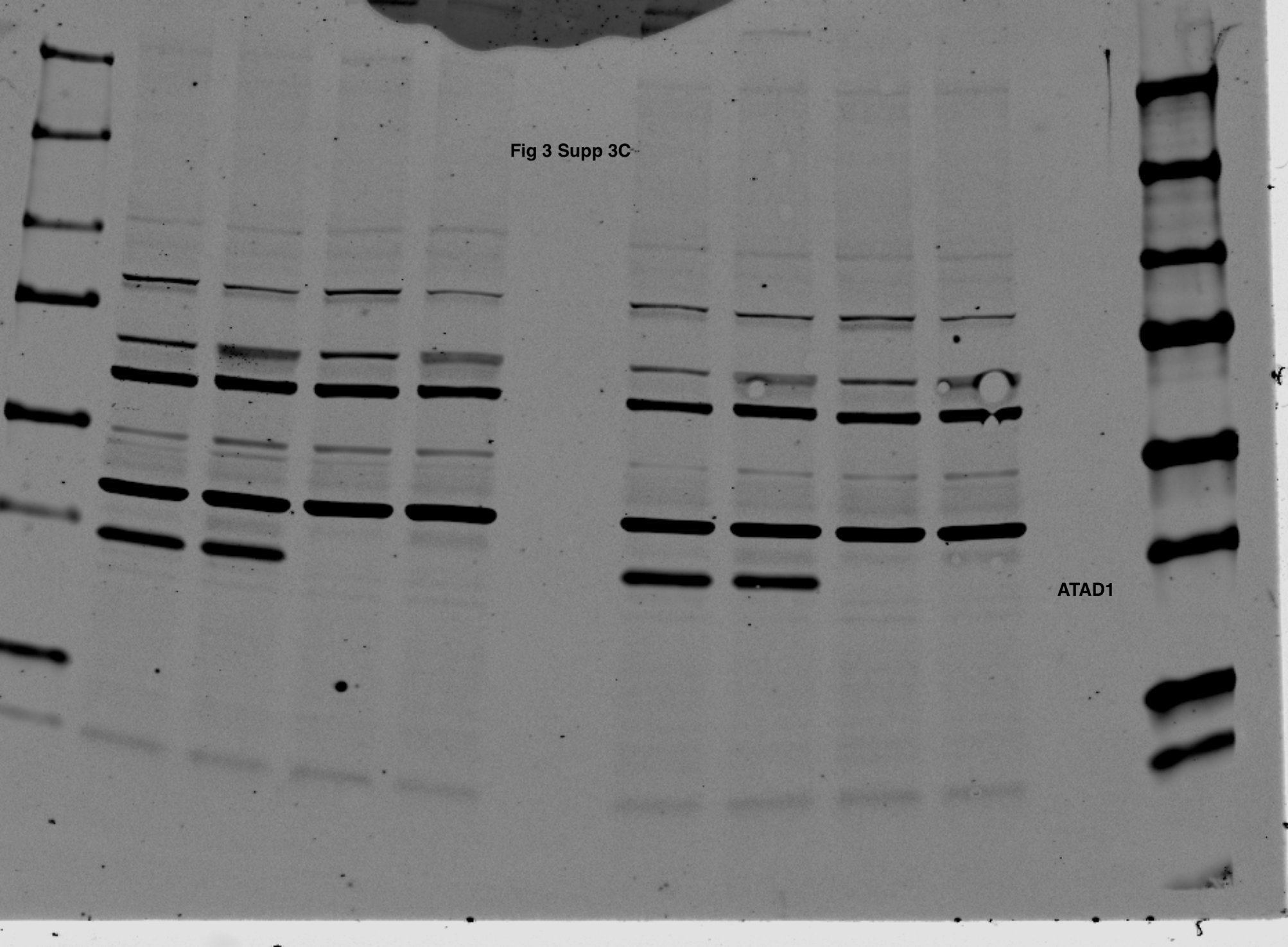

Supplement: Figure 3—figure supplement 4—source data 1. [file elife-82860-fig3-figsupp4-data1.zip › elife_Figure 3 Supp 3 source data/elife_Fig 3 Supp 3 source data 2/Fig_3_Supp_3C_labeled/Fig_3_Supp_3C_ATAD1_labeled.tif]

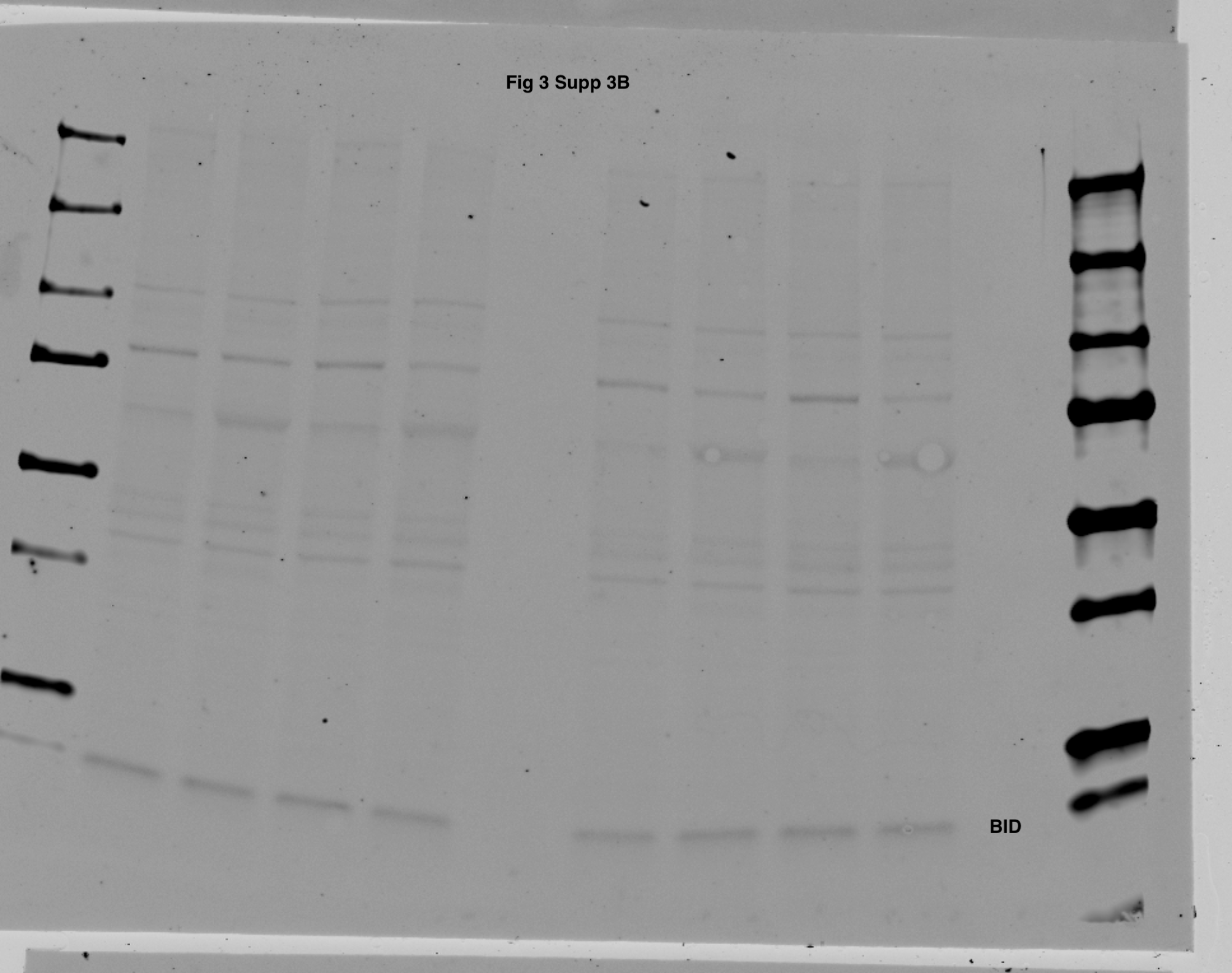

Supplement: Figure 3—figure supplement 4—source data 1. [file elife-82860-fig3-figsupp4-data1.zip › elife_Figure 3 Supp 3 source data/elife_Fig 3 Supp 3 source data 2/Fig_3_Supp_3C_labeled/Fig_3_Supp_3C_BID_labeled.tif]

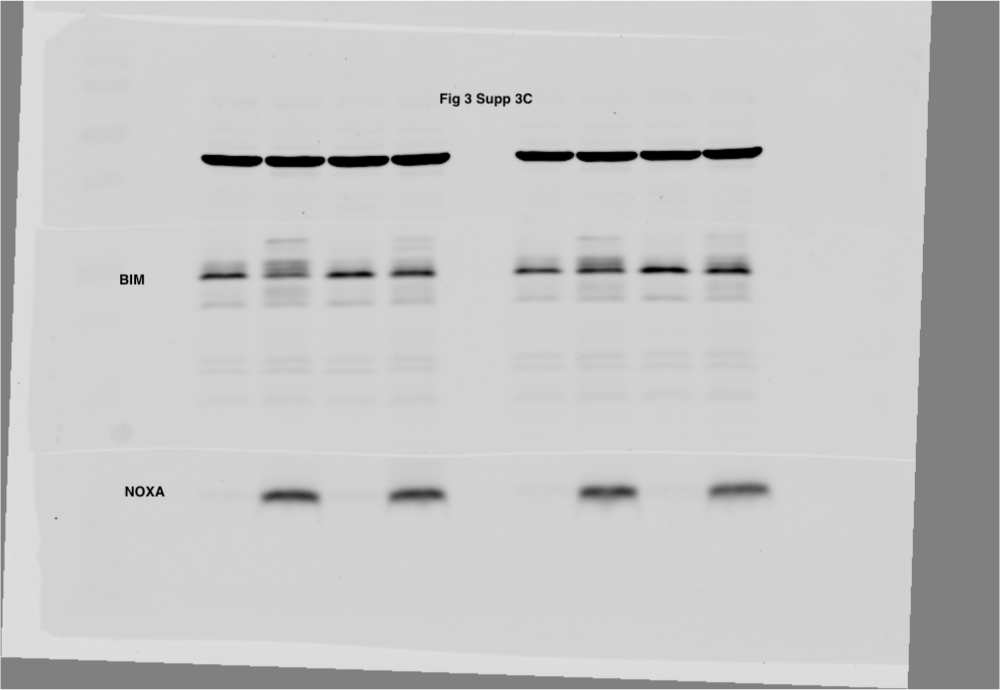

Supplement: Figure 3—figure supplement 4—source data 1. [file elife-82860-fig3-figsupp4-data1.zip › elife_Figure 3 Supp 3 source data/elife_Fig 3 Supp 3 source data 2/Fig_3_Supp_3C_labeled/Fig_3_Supp_3C_BIM_NOXA_labeled.tif]

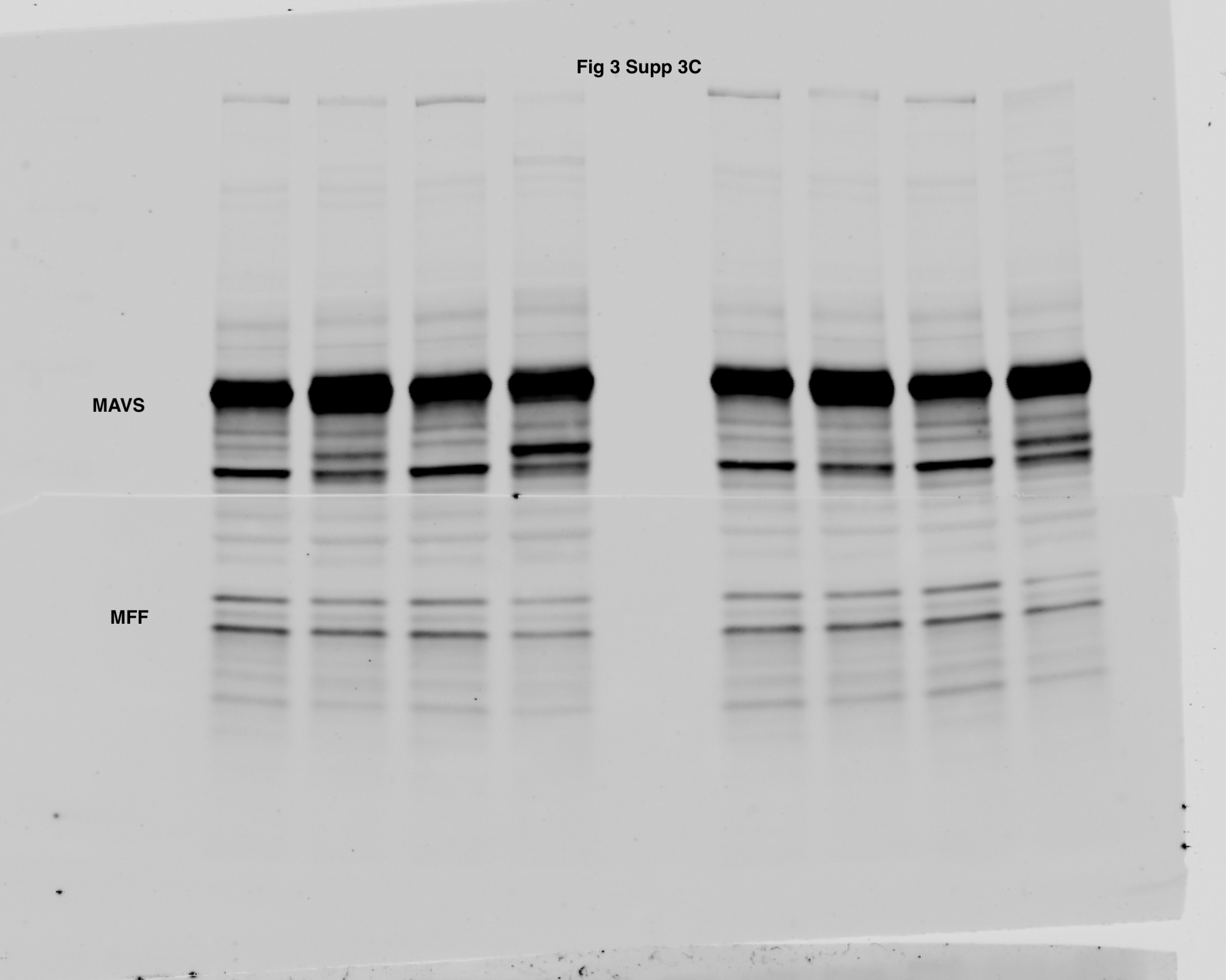

Supplement: Figure 3—figure supplement 4—source data 1. [file elife-82860-fig3-figsupp4-data1.zip › elife_Figure 3 Supp 3 source data/elife_Fig 3 Supp 3 source data 2/Fig_3_Supp_3C_labeled/Fig_3_Supp_3C_MAVS_MFF_labeled.tif]

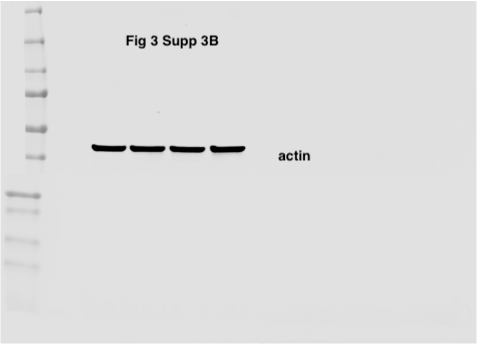

Supplement: Figure 3—figure supplement 4—source data 1. [file elife-82860-fig3-figsupp4-data1.zip › elife_Figure 3 Supp 3 source data/elife_Fig 3 Supp 3 source data 1/Fig_3_Supp_3B_Labeled/Fig_3_Supp_3B_Actin_labeled.tif]

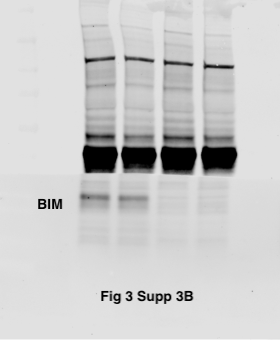

Supplement: Figure 3—figure supplement 4—source data 1. [file elife-82860-fig3-figsupp4-data1.zip › elife_Figure 3 Supp 3 source data/elife_Fig 3 Supp 3 source data 1/Fig_3_Supp_3B_Labeled/Fig_3_Supp_3B_BIM_labeled.tif]

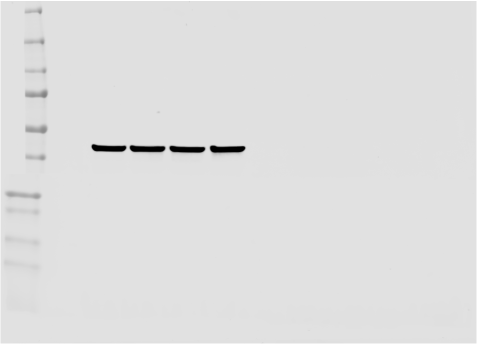

Supplement: Figure 3—figure supplement 4—source data 1. [file elife-82860-fig3-figsupp4-data1.zip › elife_Figure 3 Supp 3 source data/elife_Fig 3 Supp 3 source data 1/Fig_3_Supp_3B_Unlabeled/Fig_3_Supp_3B_Actin_Unlabeled.tif]

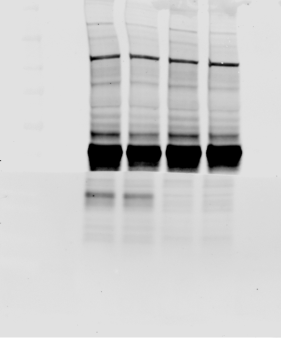

Supplement: Figure 3—figure supplement 4—source data 1. [file elife-82860-fig3-figsupp4-data1.zip › elife_Figure 3 Supp 3 source data/elife_Fig 3 Supp 3 source data 1/Fig_3_Supp_3B_Unlabeled/Fig_3_Supp_3B_BIM_Unlabeled.tif]

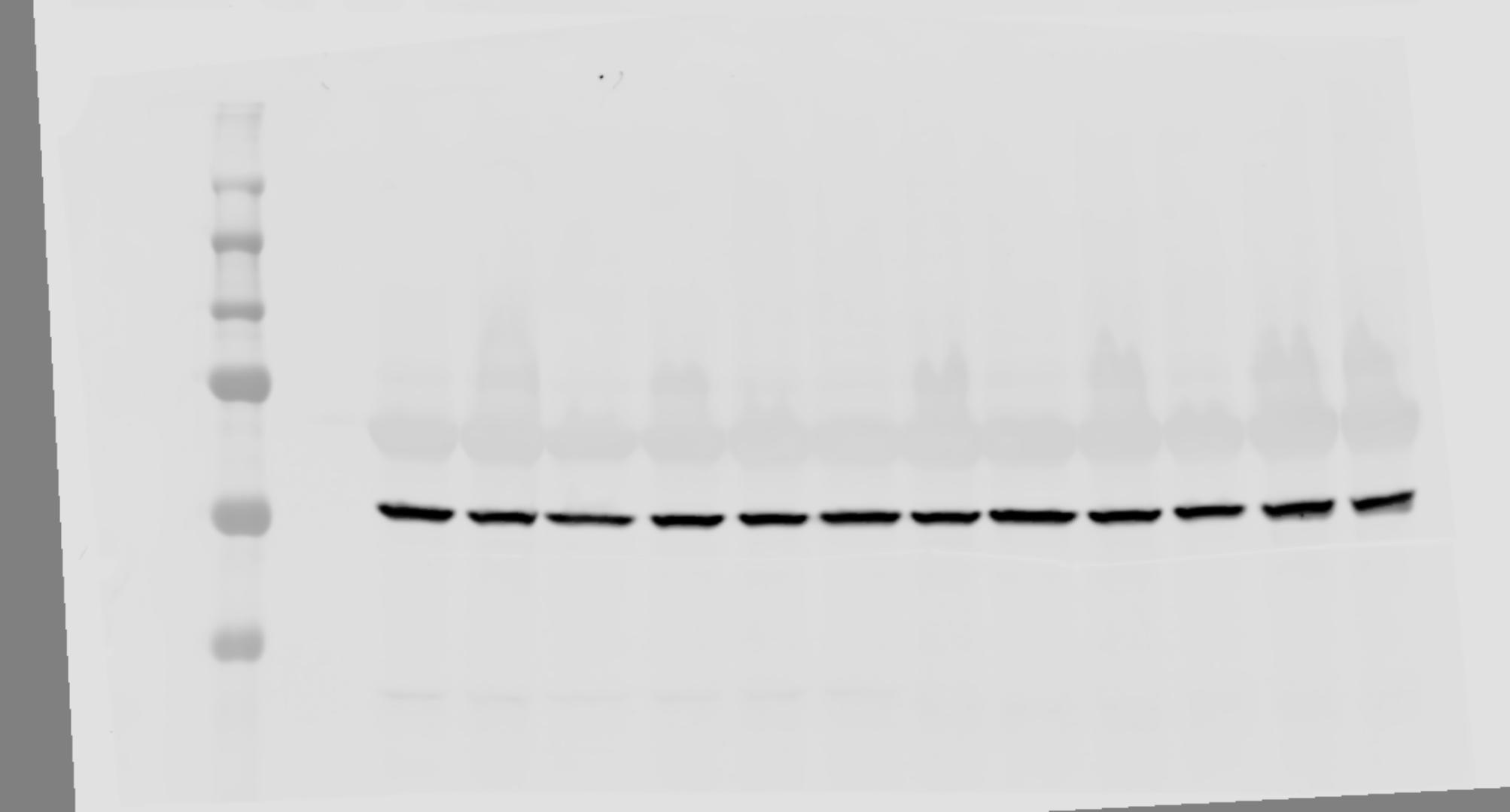

Supplement: Figure 4—source data 1. [file elife-82860-fig4-data1.zip › elife_Figure 4 source data 1/Fig_4C_Labeled/Fig_4C_Tubulin_Unlabeled.tif]

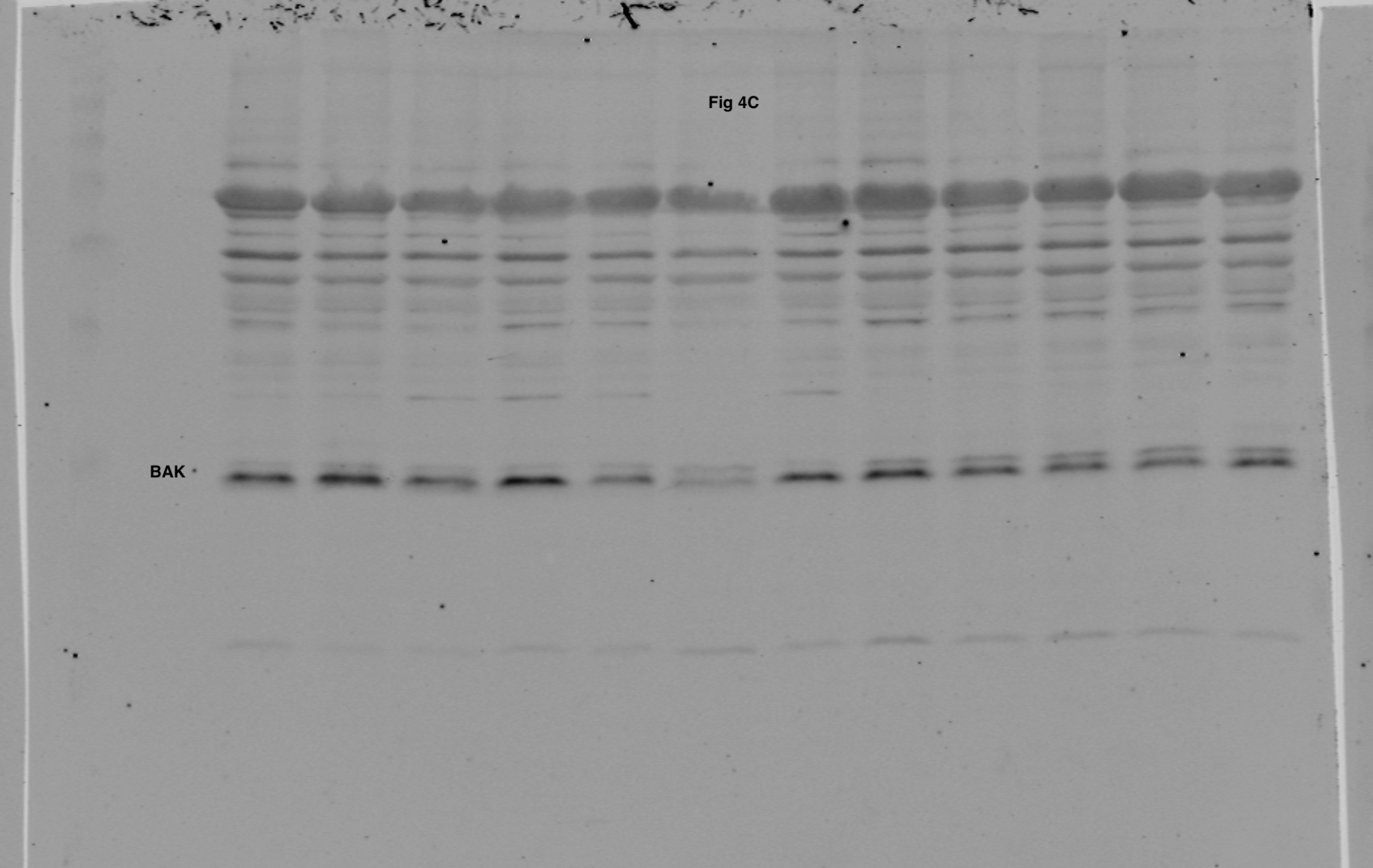

Supplement: Figure 4—source data 1. [file elife-82860-fig4-data1.zip › elife_Figure 4 source data 1/Fig_4C_Labeled/Fig_4C_BAK_labeled.tif]

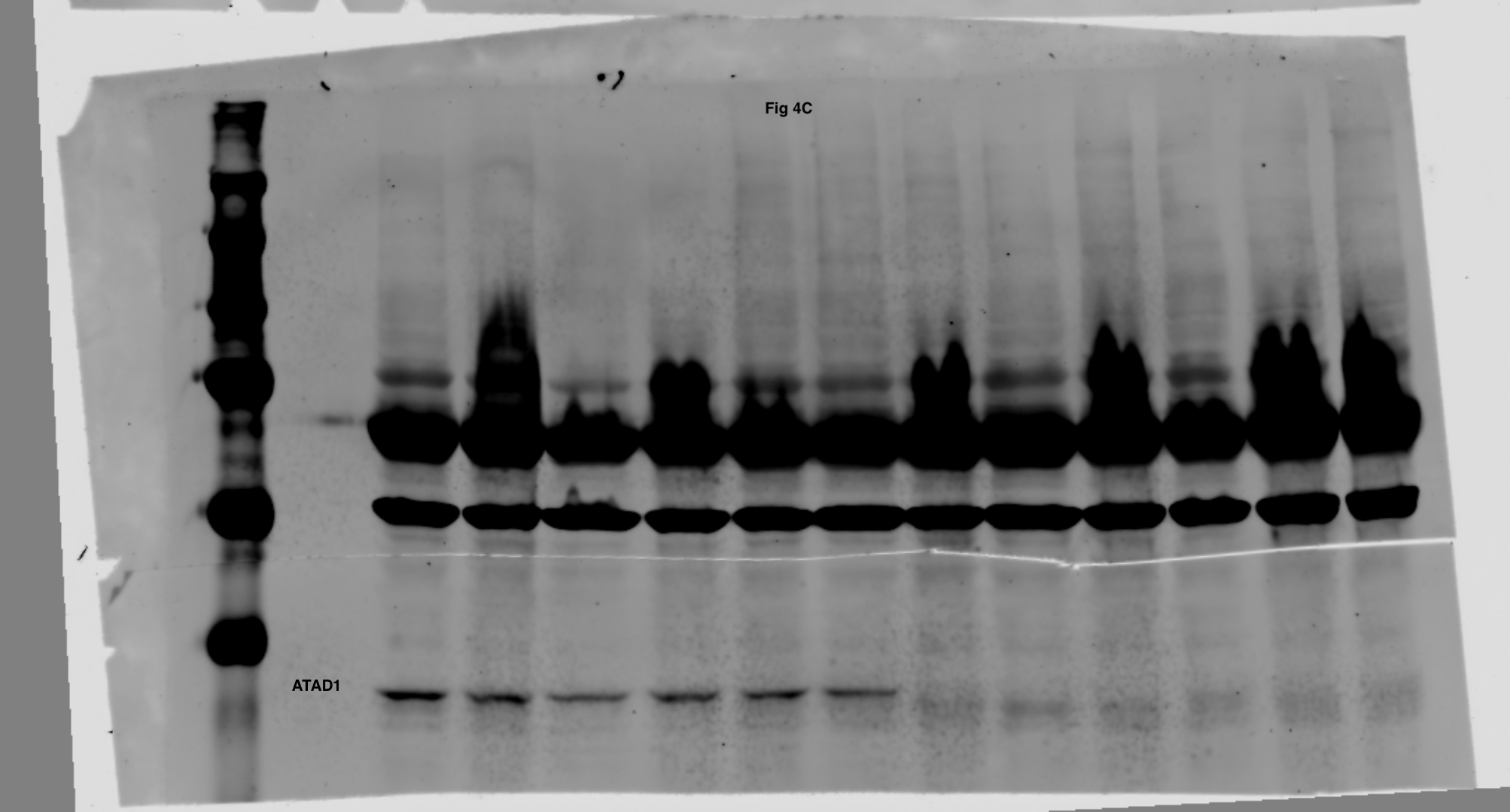

Supplement: Figure 4—source data 1. [file elife-82860-fig4-data1.zip › elife_Figure 4 source data 1/Fig_4C_Labeled/Fig_4C_ATAD1_labeled.tif]

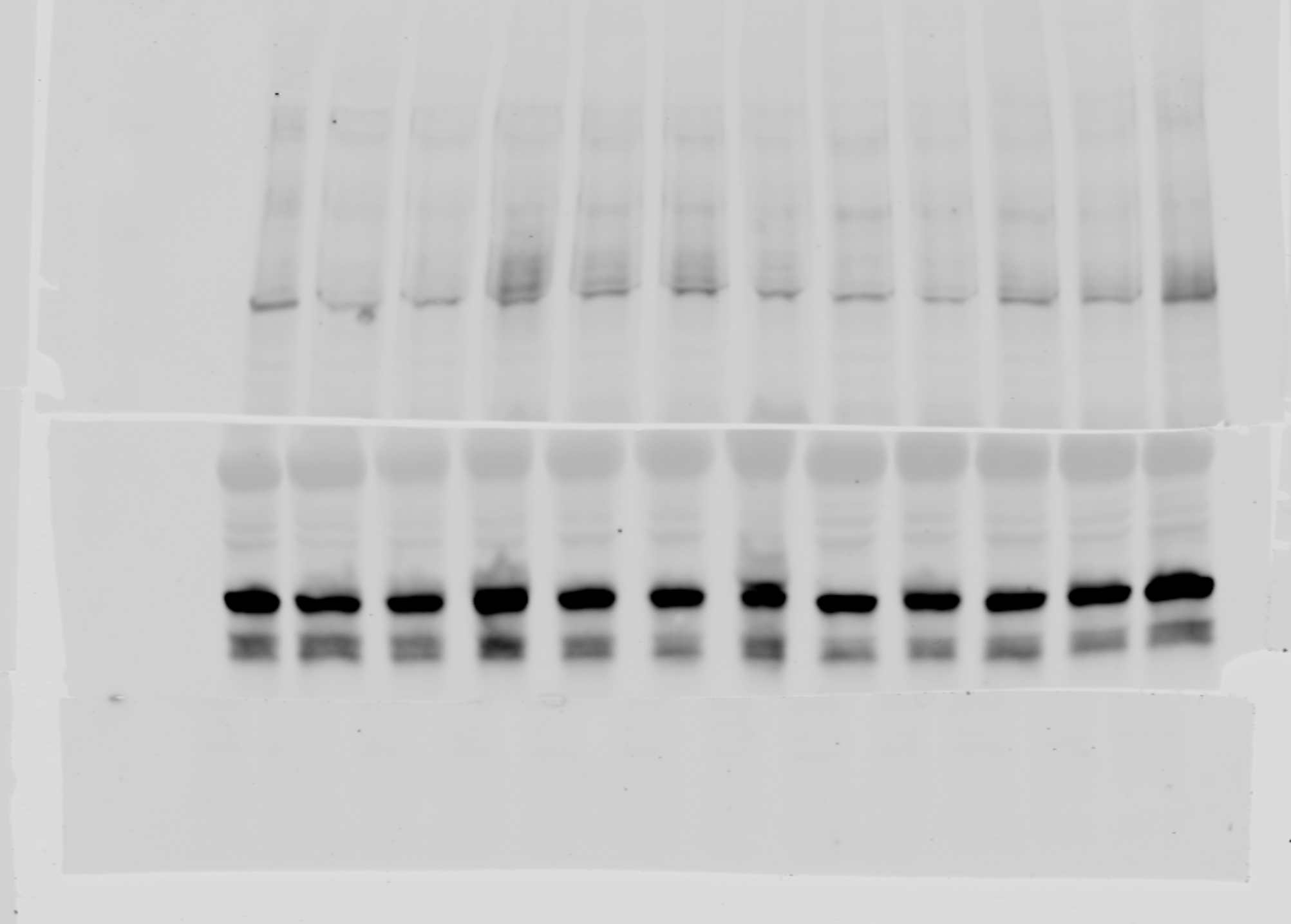

Supplement: Figure 4—source data 1. [file elife-82860-fig4-data1.zip › elife_Figure 4 source data 1/Fig_4C_Labeled/Fig_4C_NRF1_MCL1_Unlabeled.tif]

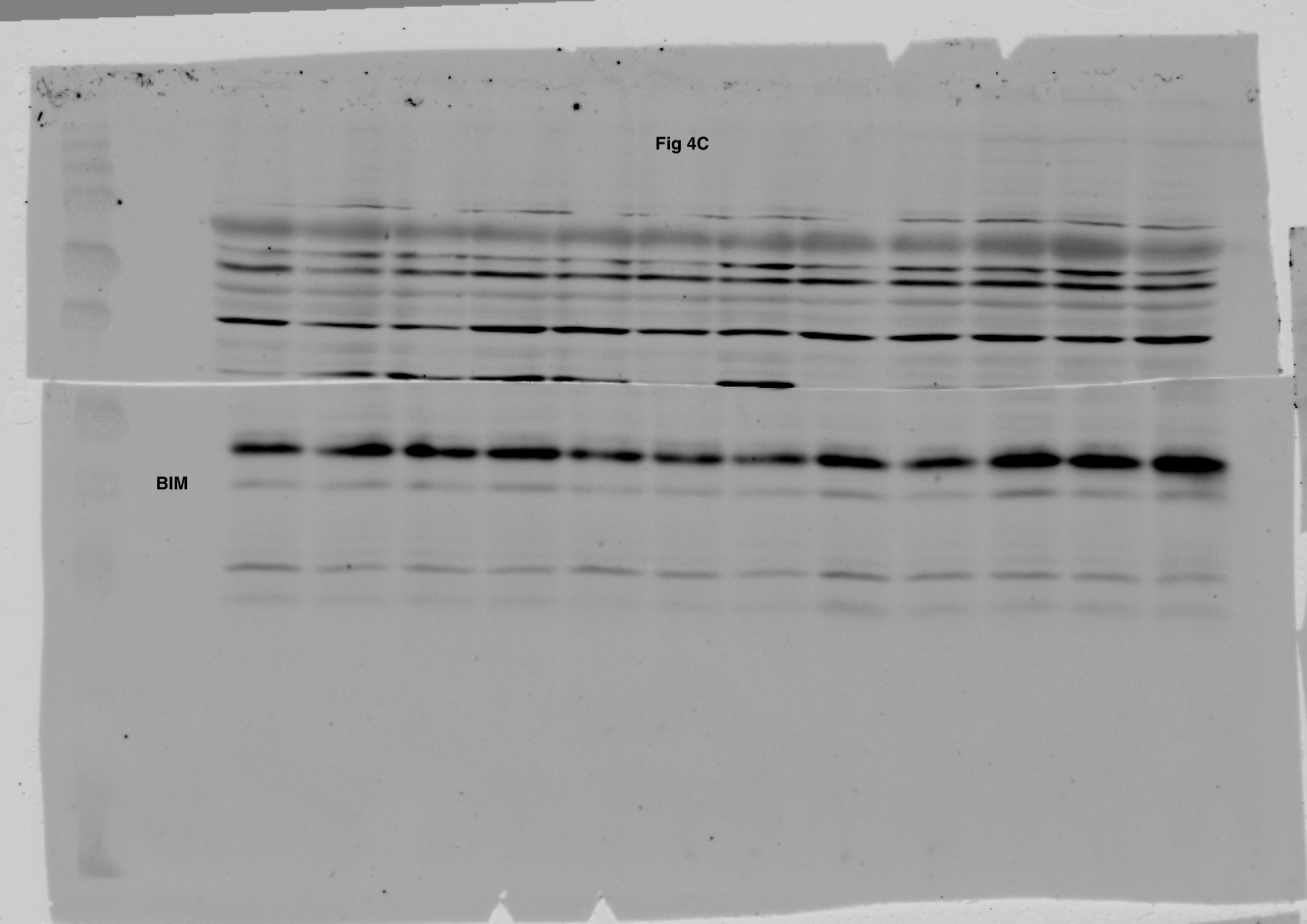

Supplement: Figure 4—source data 1. [file elife-82860-fig4-data1.zip › elife_Figure 4 source data 1/Fig_4C_Labeled/Fig_4C_BIM_labeled.tif]

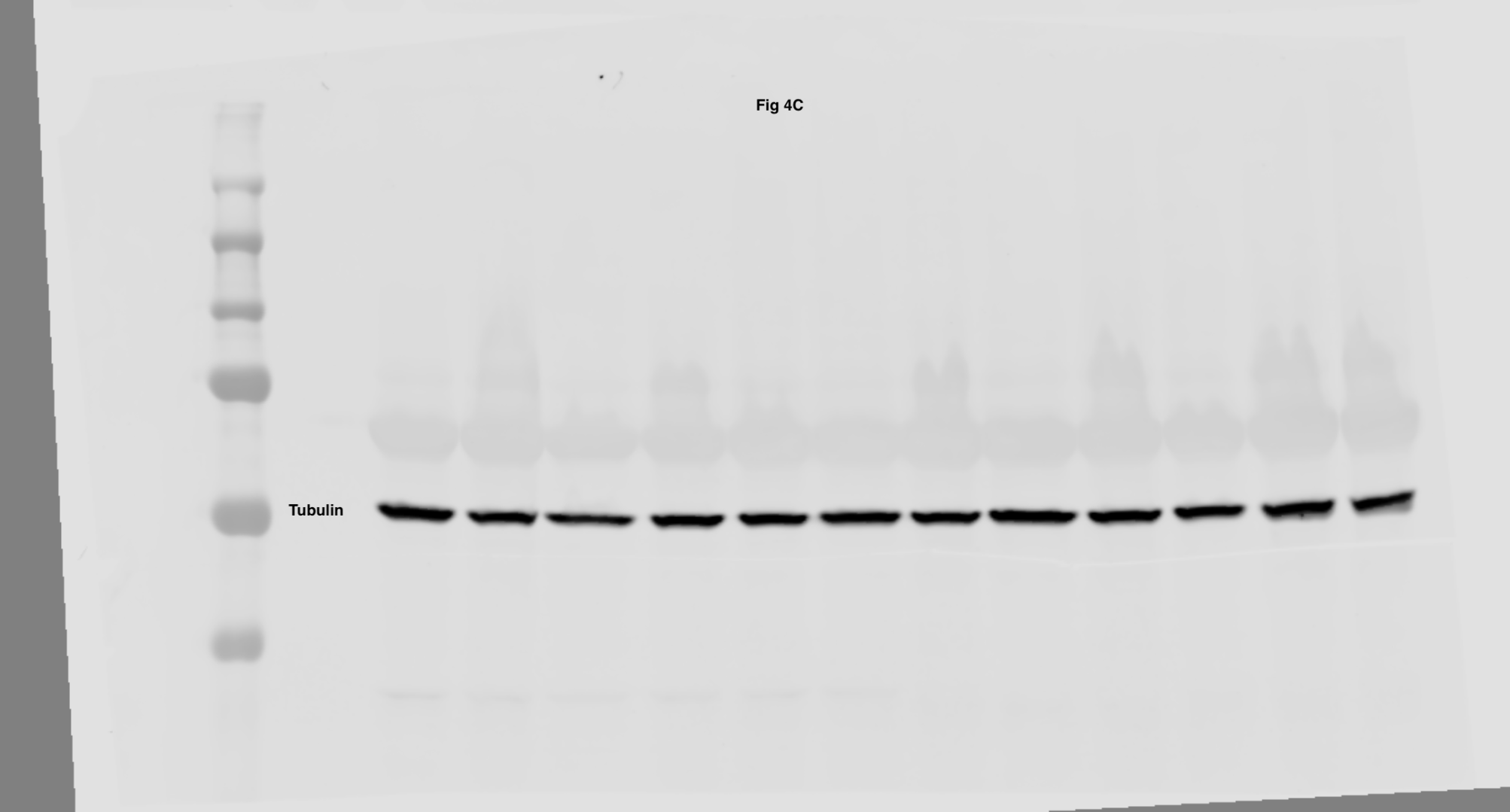

Supplement: Figure 4—source data 1. [file elife-82860-fig4-data1.zip › elife_Figure 4 source data 1/Fig_4C_Unlabeled/Fig_4C_Tubulin_labeled.tif]

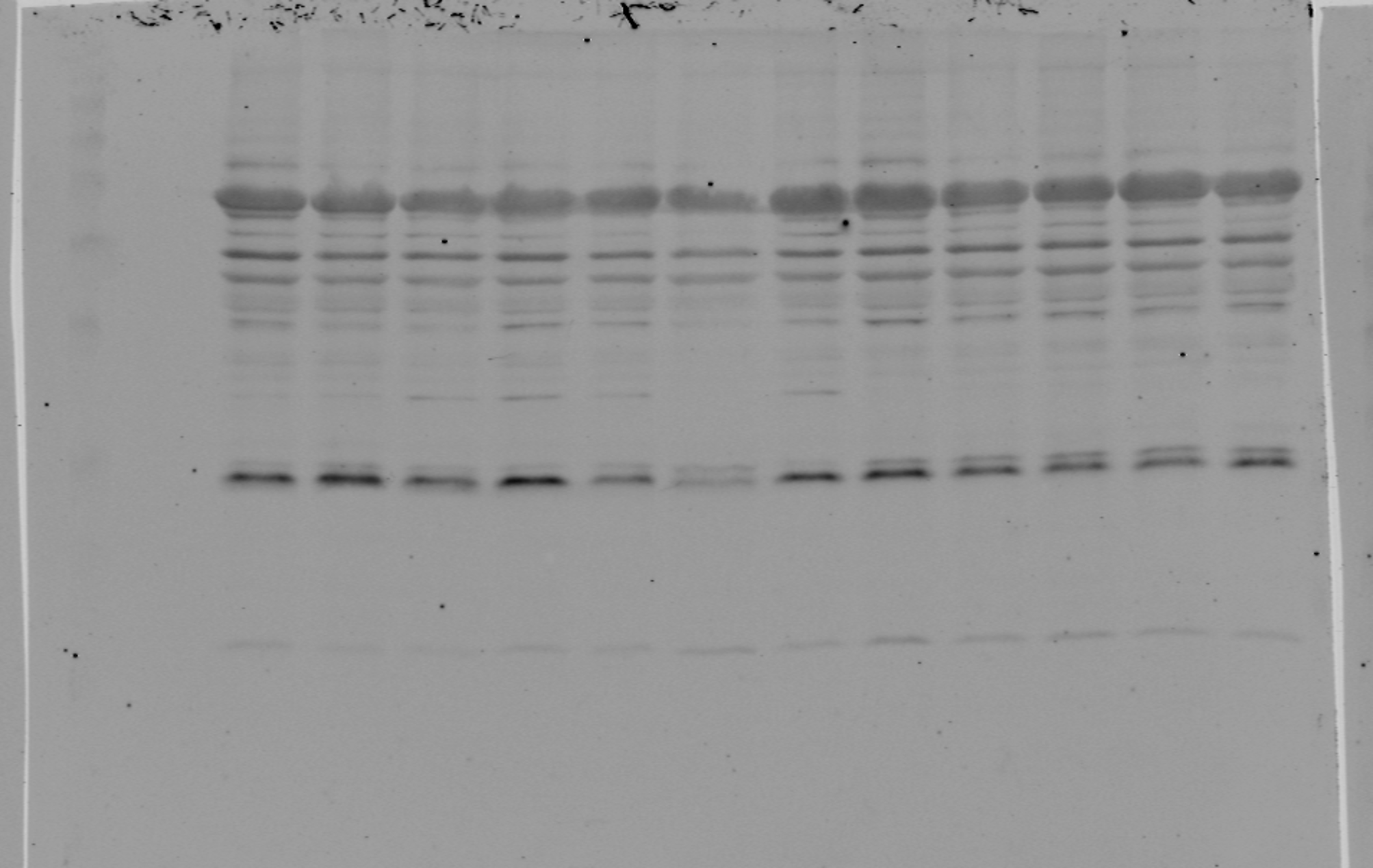

Supplement: Figure 4—source data 1. [file elife-82860-fig4-data1.zip › elife_Figure 4 source data 1/Fig_4C_Unlabeled/Fig_4C_BAK_Unlabeled.tif]

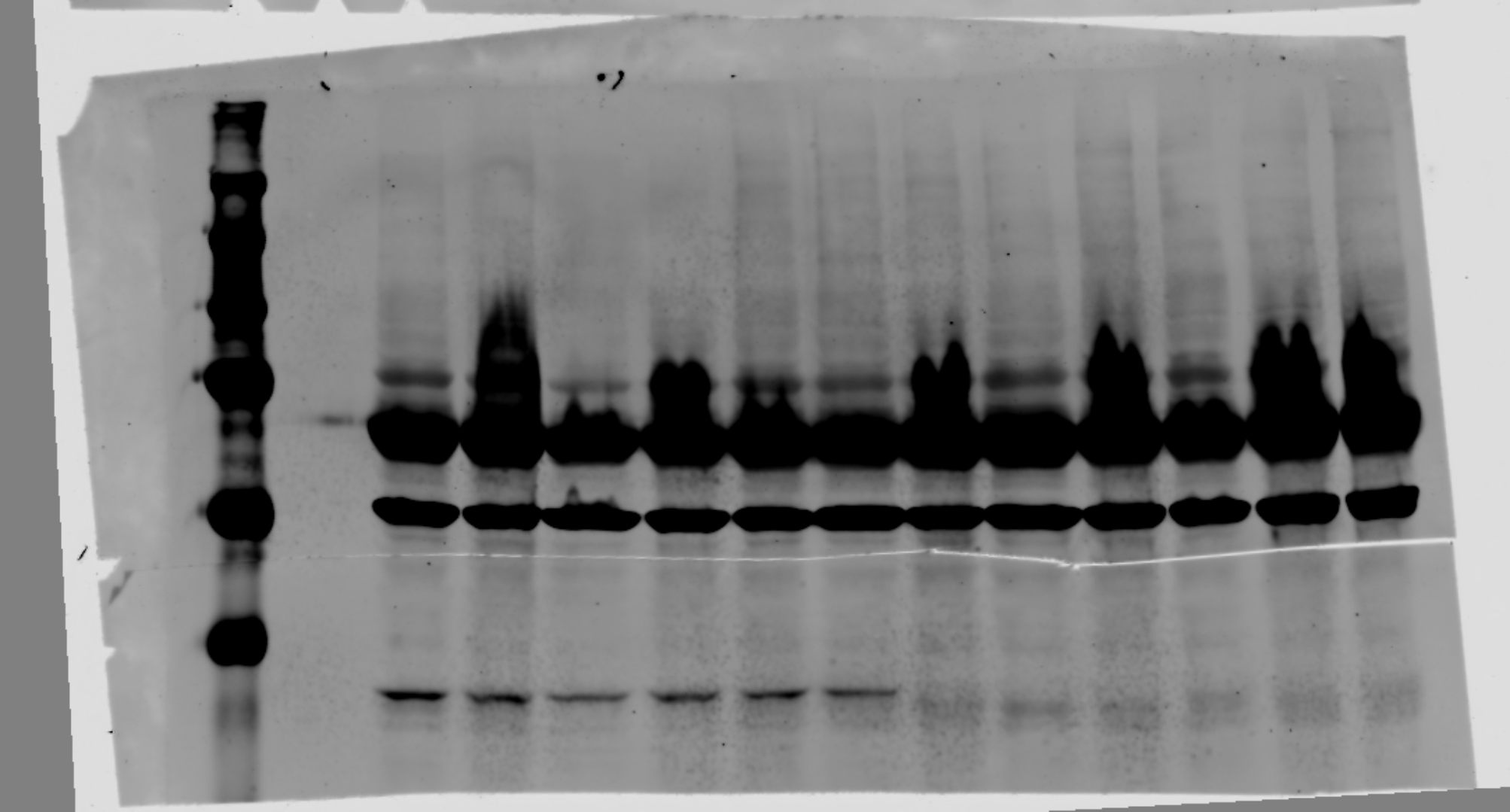

Supplement: Figure 4—source data 1. [file elife-82860-fig4-data1.zip › elife_Figure 4 source data 1/Fig_4C_Unlabeled/Fig_4C_ATAD1_Unlabeled.tif]

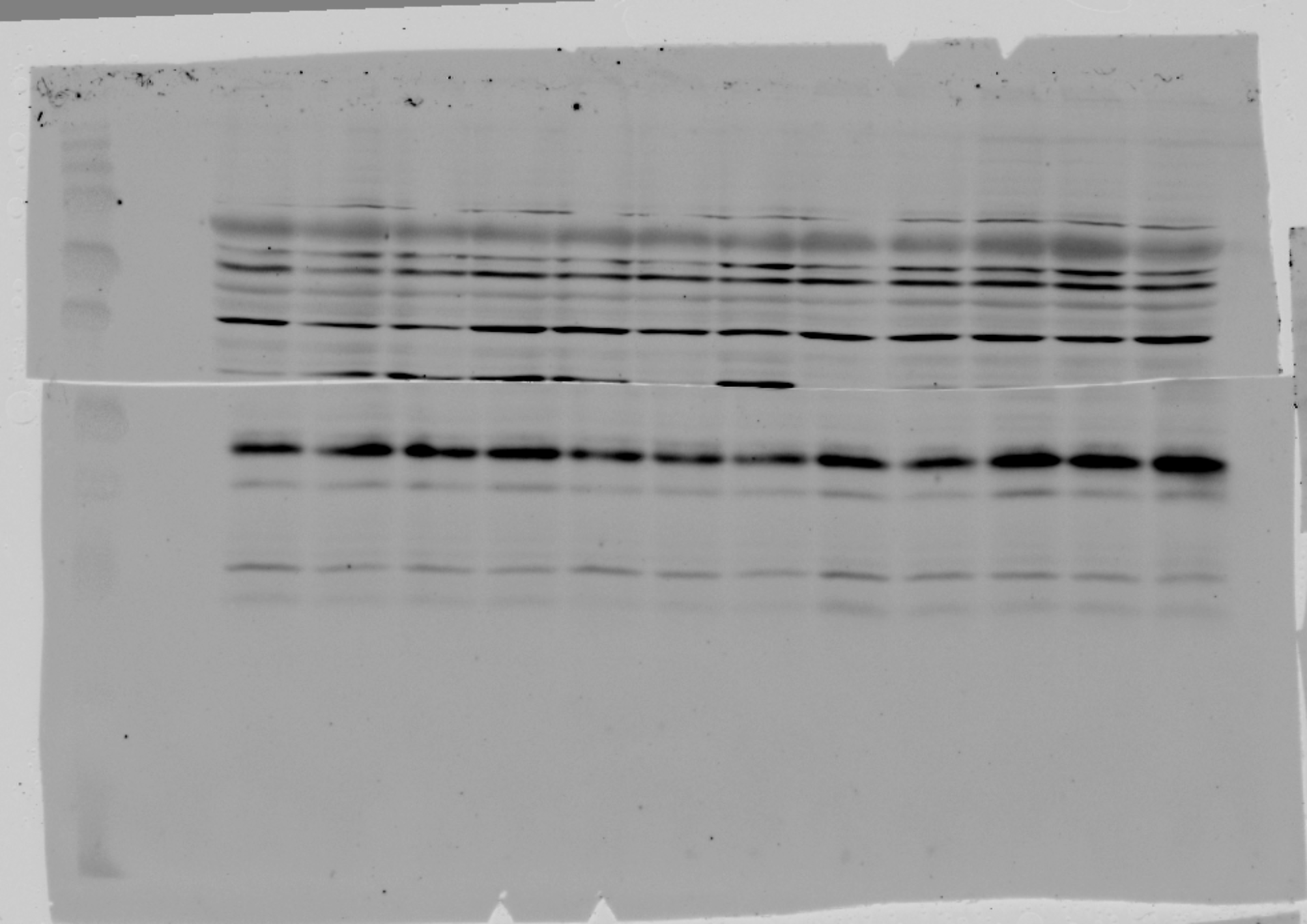

Supplement: Figure 4—source data 1. [file elife-82860-fig4-data1.zip › elife_Figure 4 source data 1/Fig_4C_Unlabeled/Fig_4C_BIM_Unlabeled.tif]

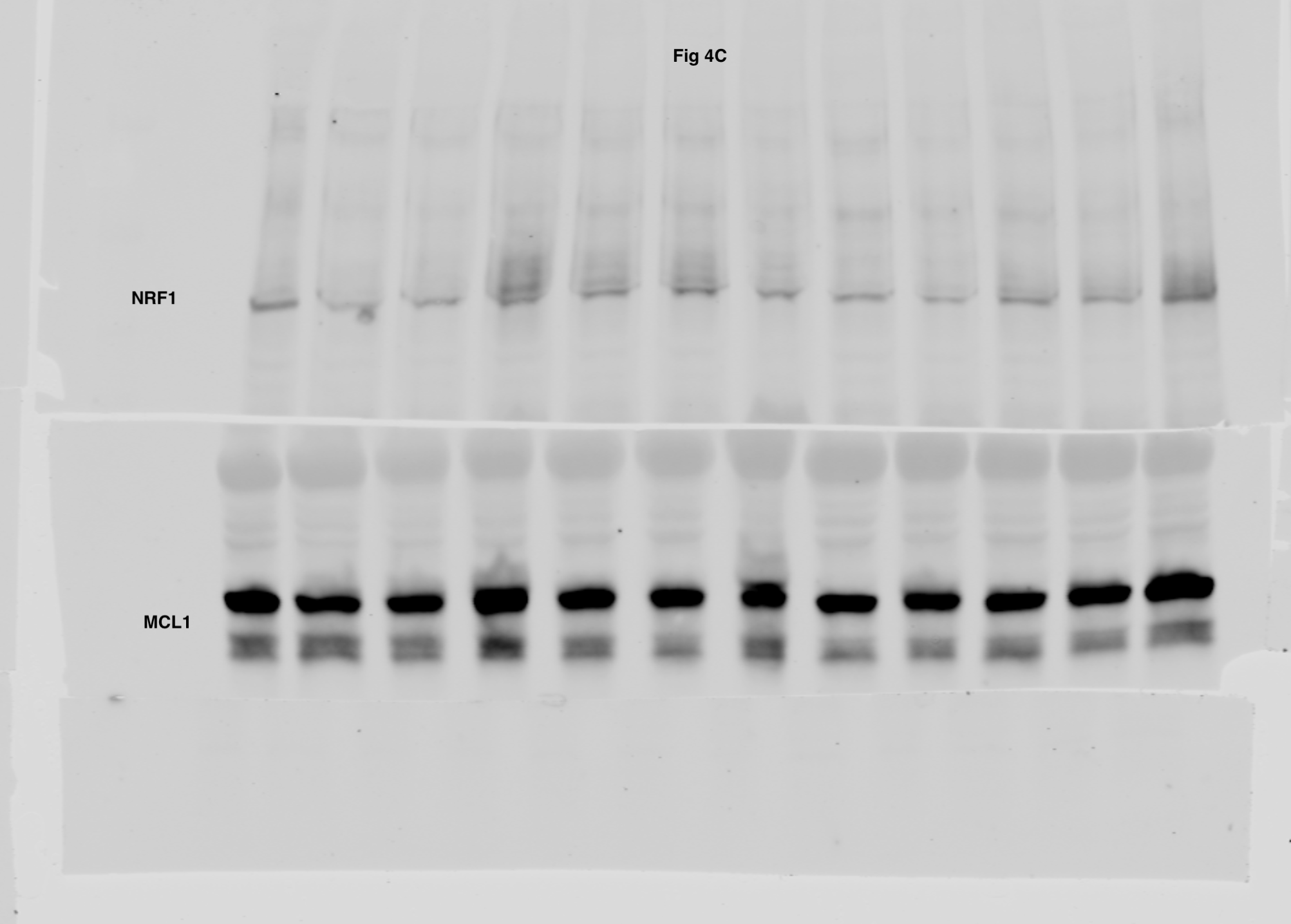

Supplement: Figure 4—source data 1. [file elife-82860-fig4-data1.zip › elife_Figure 4 source data 1/Fig_4C_Unlabeled/Fig_4C_NRF1_MCL1_labeled.tif]
